# High-Energetic Salts and Metal Complexes: Comprehensive Overview with a Focus on Use in Homemade Explosives (HME)

**DOI:** 10.3390/molecules29235588

**Published:** 2024-11-26

**Authors:** Błażej Gierczyk, Maciej Zalas, Tomasz Otłowski

**Affiliations:** Faculty of Chemistry, Adam Mickiewicz University, Uniwersytetu Poznańskiego 8, 61-614 Poznań, Poland; maciej.zalas@amu.edu.pl (M.Z.); tomek.hazmat@gmail.com (T.O.)

**Keywords:** metal-containing explosives, homemade explosives, acetylide, azide, chlorate, perchlorate, fulminate, nitrate, nitrophenolate, tetrazole

## Abstract

Metal-containing compounds form a large and rapidly expanding group of high-energy materials. Many compounds in this class attract the attention of non-professionals, who may attempt the illegal production of explosives. Several of these substances have been commercially available and pose significant danger if used by terrorists or for criminal purposes. Others are experimental compounds, kinds of curiosities, often created by pyrotechnics enthusiasts, which can present serious risks to both the creators and their immediate surroundings. The internet hosts a vast amount of information, including recipes and discussions on forums, private websites, social media, and more. This paper aims to review the variety of metal-containing explosives and discuss their appeal and potential accessibility to unauthorized individuals.

## 1. Introduction

The fascination with fire and explosions appears to be inherent in human nature. Its origins are not fully recognized, but many historians and psychologists agree that it developed over a span from 1 million to about 300,000 years ago, when humans integrated fire as a consistent element of their lives [1]. In addition to their practical uses in the military, mining, explosive demolition, etc., explosives have been applied in entertainment (primarily as fireworks) and in criminal and/or terrorist activities. These latter three are the most common reasons for preparing explosives in a home setting. Homemade explosives (HMEs) are explosives produced by non-authorized and/or non-professional individuals. HME production significantly impacts the security and stability of many regions. Besides the obvious threats of terrorism and crime, the preparation and use of HME lead to numerous severe injuries among “homegrown producers” and cause serious damage to property and material goods. Despite these clear dangers, combined with the illegality of HME preparation and ongoing legal restrictions, the unauthorized production of explosives has grown rapidly in recent years [2].

The vast majority of non-professionals prepare simple HME materials using easily accessible ingredients and involving straightforward preparation procedures. The fundamental reasons for the popularity of these simple methods differ depending on the group interested in preparing the material. For criminal and terrorist use, it is advantageous to prepare explosives discreetly, using regular substances that will not attract attention. Additionally, the relative stability and easy handling of HMEs are of high importance to these two groups in particular. This explains the significant popularity of a small selection of inorganic compounds (e.g., nitrates, chlorates) and organic compounds (e.g., TATP, HMTD) in criminal and terrorist applications [3,4].

Most amateur fireworks producers also rely on uncomplicated procedures and accessible materials. This group primarily seeks fast and spectacular explosion effects to impress themselves and/or their audience. Among homegrown fireworks makers, there is a small group that seeks to develop its skills and experiment with more demanding, advanced, and sophisticated materials. Such individuals often do not publicize their activities; they produce HMEs for personal satisfaction, aiming for products with high efficiency, high destructive power, and/or high sound intensity. Accidental explosions are most common among this small group of enthusiastic HME amateurs.

**Figure 1:**
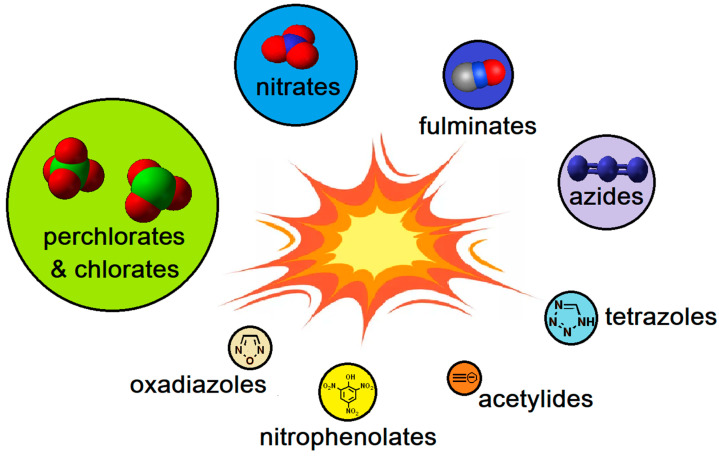
Major classes of metal ion-containing explosives (circle area is proportional to the number of documents in the Google Scholar database).

This paper compiles properties and technical data on explosive materials containing metal salts or complexes used in HME (Figure 1). Additionally, this work encompasses materials that could attract the attention of non-professional producers in the future. We review data on the most popular, as well as rare, chemical compounds used in HMEs. The data were collected from online platforms frequented by amateur explosives makers and compared with scientific data published in international journals.

Across the world, security agencies face the problem of illegally produced explosive materials and devices, which are the instruments of terrorist and partisan fighter actions, as well as tools for ordinary criminals (e.g., for ATM break-ins, mafia showdowns, or victim intimidation). Another aspect is the security of rescue services (e.g., firefighters or chemical hazard boards) in cases of accidental or intentional explosions or fires in illegal laboratories run by pyrotechnics enthusiasts. Although agency officers are well-prepared and trained to handle hazards arising from conventional explosives (e.g., PETN or TNT), chemical formulations used in HMEs create a greater challenge.

Firstly, many of these formulations are new to the agents, who have not had the opportunity to encounter them during training or work. Moreover, illegally produced materials may be more sensitive or even self-reactive due to impurities, improper crystal forms, the absence of phlegmatizing agents, or incorrect storage. On the other hand, many hobbyists tend to produce substances exhibiting extreme sensitivity due to their desire for spectacular effects and their unhealthy fascination.

Intensive research on novel high-energy materials, stimulated by the search for greener explosives or substances with unique application-related properties (e.g., laser-impulse sensitivity), has resulted in numerous scientific publications and technical reports that often inspire the illegal production of HMEs. The vast diversity of materials used in homemade explosives can be seen in 21st-century examples of incidents and accidents summarized in Table 1.

## 2. General Considerations

1. This paper aims to gather information about metal salts and complexes used as explosives. As “energetic materials” are currently a highly discussed topic, it is often difficult to determine if certain materials meet our criteria, as numerous compounds are described as “potentially explosive” or “promising energetic materials” based solely on DCS measurements and thermodynamic calculations, often without any experimental initiation tests. By contrast, it is much easier to identify primary explosives; in simple terms, these are materials that explode or deflagrate upon stimulation.
2. Tables with basic parameters, including impact, friction, and electric-discharge sensitivity, material density, heat of formation, detonation velocity, detonation/deflagration energy, detonation pressure, and behavior in hot plate, hot needle, and laser tests are provided as Supplementary Materials. Contemporary data are summarized in Table S6, while data for certain older materials not re-evaluated are found in Tables S1–S5.
3. In the case of numerous compounds, especially those published in the 19th and 20th centuries, available data are difficult to compare with current findings because there are no unified testing methods. Additionally, new data are often unreliable due to a lack of detailed material or testing information; for example, sensitivity depends significantly on the degree of substance hydration and the grain size used for sensitivity determination. If a compound was not analyzed for elemental composition or granulometric data are absent, comparisons and generalizations become challenging. The characterization scheme applied by Klapötke’s group could serve as a “gold standard” in high-energy material chemistry. Similar issues arise with patents, as complete physicochemical data for patented explosives are often unavailable. Where possible, data reported in other units have been converted to BAM recommendations and are shown in italics; in other cases, the original data are provided.
4. Values provided in source publications in [cal] were converted to [J] using the conversion factor 1 cal = 4.186 J.
5. Abbreviations used:
  *DV*—detonation velocity, *ESD*—electrostatic discharge, *FS*—friction sensitivity, *HOF*—the heat of formation, *IS*—impact sensitivity;
  ECC—energetic coordination compound, EMOF—energetic MOF, HME—homemade explosive, MOF—metal–organic framework;
  LA—lead azide, LSt—lead styphnate, MF—mercury fulminate, PETN—pentaerythritol tetranitrate (pentrite), RDX—1,3,5-trinitro-1,3,5-triazinane (hexogen), TNT—2,4,6-trinitrotoluene.
  Abbreviations for other compounds are provided in figures with compound structures. Please note that numerous compounds may exist in neutral form (HA; e.g., tetrazole: HTz, CHN_4_) or anions (A^−^; e.g., tetrazolate: Tz, CN_4_^−^).
6. Sensitivity scale according to U.N. Recommendations on the Transport of Dangerous Goods:
  Impact sensitivity: insensitive > 40 J, less sensitive > 35 J, sensitive > 4 J, very sensitive > 3 J;
  Friction sensitivity: insensitive > 360 N, less sensitive = 360 N, sensitive < 360, > 80 N, very sensitive < 80 N, extreme sensitive < 10 N.
7. For the readers’ convenience, the reference list and numbering are consistent in both the main text and the Supplementary Materials.
8. For each class of compounds, remarks regarding their illegal synthesis by HME producers were added based on information published on internet sites, social media, discussion boards, and government agency reports.

## 3. Systematic Survey of Metal Ion-Containing Explosives

### 3.1. Azides

Azides are the salts of the weak hydrazoic acid (HN_3_). This free acid is volatile (b.p. 37 °C), highly toxic, and explosive. Several reports describe its spontaneous detonation in liquid, gaseous, or dissolved form [5,6,7]. It has limited practical use in organic and inorganic synthesis. Inorganic azides contain the azide ion (N_3_^−^), a linear pseudohalogen explosophore. The azide ion is colorless, so azides may appear colored only if the cation absorbs visible light. All azides are toxic, and the soluble forms are as toxic as potassium cyanide. When in contact with acids, they release hydrazoic acid, which may spontaneously explode [8]. Azides decompose, releasing a large amount of nitrogen. The basic properties of several explosive inorganic azide salts and complexes are summarized in Tables S1 and S6.

Sodium azide is the most accessible salt of hydrazoic acid. It is not explosive but decomposes upon heating, releasing a large volume of N_2_. Due to this property, it is used in car airbags. NaN_3_ is an essential precursor for other inorganic and organic azides, making it of interest to HME producers. Mixtures of sodium azide with other high-energy materials (e.g., RDX, ammonium nitrate) increase the nitrogen content and result in enhanced explosive efficiency [9].

Among other metal azides, lead(II) azide (LA) is particularly important, as it is commercially produced and used as a primary explosive in primers, fuses, detonators, shells, percussion caps, and cartridges. It exists in four polymorphic forms: α (orthorhombic) is the most stable and the only one used in industry; the β (monoclinic), γ (monoclinic), and δ (triclinic) forms are less stable and slightly more sensitive. Therefore, these forms are avoided during Pb(N_3_)_2_ production. Commercial LA is mainly stabilized by polymers such as poly(vinyl alcohol), dextrin, gelatin, or carboxymethyl cellulose. The use of these compounds results in a less sensitive and more stable product. Moreover, their presence during crystallization leads to the formation of smaller crystals [10]. Some authors have reported that large crystals (approx. 1 mm or more) of Pb(N_3_)_2_, especially the β polymorph, tend to spontaneously explode due to internal strains. However, further studies indicate that this is a myth. Instead, a strong correlation between impact and heat sensitivity and crystal size has been confirmed [11]. LA can explode in its wet form. It is highly sensitive to various initiating factors, such as heat, mechanical shock (0.7–4 J), friction (0.5 N), or static discharge (6–12 mJ), and it exhibits an immediate deflagration-to-detonation transition [12]. In contact with acids, LA may release HN_3_, while contact with many metals and alloys can lead to the formation of more sensitive metal azides. Storing LA in contact with air promotes its reactivity with metals, hydrolysis, and the formation of basic lead(II) azide. The structure of basic lead(II) azides, previously thought to contain the OH^−^ anion, has been revised and is now recognized as an oxosalt [13,14].

Other metal azides are less popular and not used in commercial products. Silver(I) azide, which has similar sensitivity and initiation properties (1.2–3.8 J for mechanical shock, 1.3 mJ for electric discharge), has been used in some solid, small-size detonators. However, its production costs have limited its use, and it is no longer produced [11,15]. Conflicting information on its properties has been reported—ranging from extreme sensitivity and explosivity (even when crystals are broken underwater) to low sensitivity to shock or impact [11,16]. The colloidal form of AgN_3_ is less sensitive than the crystalline form. It has higher sensitivity to friction than LA but is less sensitive to impact. Its critical diameter is very small, with even crystals of only 25 µm in diameter capable of exploding [17]. Non-professionals often produce it for use in HME.

Copper(II) azide has also been reported as a potential detonator component but was never implemented in production. Cadmium azide has limited applications as a component in high-temperature stable detonators [11].

**Figure 2:**
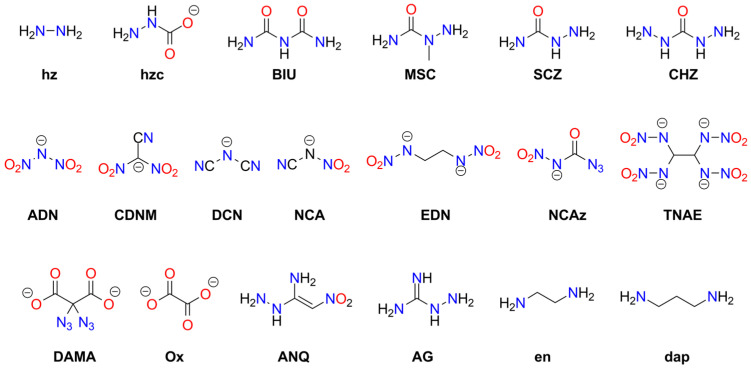
Acyclic ligands and anions used in metal-containing explosives.

Numerous metal complexes containing the azido ligand or azide anion have been synthesized. Some explosive ammine complexes of Cu and Cr are listed in Table S1. Many other derivatives containing organic amine ligands or ammonium cations do not tend to explode or detonate [16]. Notable explosive coordination compounds containing azide include complexes of copper(II) azide with triazole or tetrazole ligands [18,19,20] and with imidazole [21] (see Table S6). These compounds are more stable than Cu(N_3_)_2_ and exhibit promising properties: detonation velocities (DV) of 7.8–8.3 [km s^−1^], detonation upon heating or mechanical shock, and the ability to initiate conventional materials such as PETN. Wurzenberg et al. also synthesized and characterized several phlegmatized complexes stabilized with dextrin, polysorbate, carboxymethyl cellulose, and polyvinyl butyral [22]. Joyner et al. reported an interesting series of cobalt ammine complexes containing azide as a counterion or ligand. All of these compounds detonate upon ignition or impact [23]. Several other acyclic ligands have been studied for the complexation of metal azides: hydrazine (**hz**; Co(II), Ni(II), Zn(II)) [24,25,26], ethylenediamine (**en**; Cd) [27], and 3-amino-1-nitroguanidine (**ANQ**; Co(II), Ni(II)) [28]. Additionally, explosive complexes of mercury(II) azide and cadmium azide have been prepared [29,30]. Lund et al. reported an interesting example of an azide of Millon’s ion, [Hg_2_N]N_3_, which also exhibits explosive properties [31]. The structures of acyclic ligands and anions used in metal-containing explosives are presented in Figure 2.

The search for new monomeric and polymeric coordination compounds containing azide has become part of the trend in developing “green” (lead- and mercury-free) primary explosives for commercial applications. Cobalt, copper, and even cadmium azide derivatives, which are more stable than their parent salts, appear to be a promising route for such materials [32,33,34,35,36,37].

Metal azides and their complexes have been tested in various composite primer formulations, e.g., with K_2_BNATz [38] or incorporated into nanomaterials such as porous carbon [34,39,40]. The complex of copper(I) azide with 2-aminopyrazine (**2APr**) does not exhibit explosive properties on its own, showing only ignition in friction tests. However, it has been used as a 1% admixture with octogen to accelerate HMX decomposition [41].

Besides ionic azides, numerous covalent organic and inorganic derivatives have been reported, such as Al(N_3_)_3_, As(N_3_)_3_, and Si(N_3_)_4_. These compounds are beyond the scope of the current review. For further details, see the monographs by Fair and Walker [16], Urbański [15], Matyáš and Pachman [11], and the review [42].

A separate class of azido-derived explosives includes Werner-type complexes. Lindart and Flygare described detonation upon heating (195–200 °C) of azidopentaamminecobalt(III) azide [43]. Diazidotetraamminecobalt(III) salts are more sensitive; both the azide and iodide salts of this cation explode upon heating (around 180 °C). The formation of nitrogen triiodide prior to detonation has been postulated for [Co(NH_3_)_4_(N_3_)_2_]I. There is no difference in reactivity between the *cis*- and *trans*-[Co(NH_3_)_4_(N_3_)_2_]^+^ salts. Nitrates and perchlorates of diazidotetraamminecobalt(III) deflagrate at 150–170 °C and 200–220 °C, respectively, with the *trans* complexes being more stable. Both [Co(NH_3_)_4_(N_3_)_2_](N_3_) and [Co(NH_3_)_4_(N_3_)_2_](ClO_4_) detonate upon impact in hammer tests, with the behavior of *cis* and *trans* isomers remaining similar [44]. The neutral triazido-triamminecobalt(III) compound is also explosive when heated, impacted, or subjected to friction [45]. The *mer*-triazido-ethylenediamine-pyridinecobalt(III) complex is extremely sensitive, exploding even in a wet state [46]. Siebert and Macht synthesized salts containing the [Co(N_3_)_6_]^3−^ anion. All compounds studied were explosive upon heating or rubbing, or they even exploded spontaneously. The formation of a highly sensitive, unidentified by-product in alkaline solution was also reported [47]. A similar complex, containing eight azido ligands bound to a Co^3+^ atom, [Co(N_3_)_6_][Co(NH_3_)_4_(N_3_)_2_], was reported as explosive by Druding et al. [48].

Sodium azide can be legally purchased and possessed by non-professionals in many countries, but it is listed on the Interpol Explosive Precursor Chemicals list [49]. However, numerous procedures for synthesizing it from more widely available chemicals (such as hydrazine salts and sodium nitrite) are also published online. Moreover, sodium azide is readily accessible from car emergency-inflation systems. Additionally, there are numerous tutorials and videos available online for producing LA (lead azide). The ease of producing Pb(N_3_)_2_ makes this compound a significant risk to public security, as it may be used in criminal and terrorist acts. For example, Pb(N_3_)_2_ was used by al-Qaeda in a parcel bomb discovered in Dubai in 2010 [50] and was produced by pyrotechnics enthusiasts in Chicago (2021) [51] and Longmont (2009) [52]. Among non-professional pyrotechnic makers, other metal azides, such as copper, cobalt, zinc, strontium, and barium derivatives, are also popular. All these salts are extremely dangerous, posing serious risks to both the experimenter and their surroundings. Old samples of metal azides or failed improvised explosive devices containing these compounds can become even more unpredictable if unexpected reactions occur between the azide compound and metallic elements or containers.

### 3.2. Fulminates

**Figure 3:**
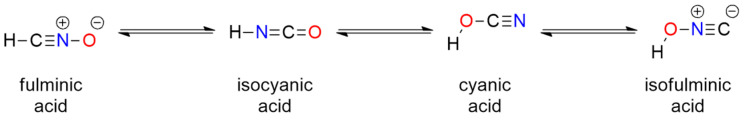
Tautomerism of fulminic acid.

Fulminic acid (HCNO) is a weak, unstable (prone to polymerization), toxic, and gaseous compound. The currently accepted structure is a zwitterionic form, formonitrile N-oxide: H−C≡N^+^−O^−^ (Figure 3). Its salts, fulminates, exhibit a high degree of covalent bond character between metal and carbon atoms; for example, mercury fulminate is covalent, while thallium fulminate is ionic [53]. The free acid can be prepared by reacting sodium fulminate with diluted sulfuric acid and isolating it by extraction with diethyl ether or flushing it with a stream of inert gas. Another method involves HI elimination from the oxime of formic acid iodide (HIC=NOH). The free acid readily polymerizes to form oligomers and polymers [54,55]. Fulminic acid and its salts are highly toxic. The basic properties of the acid and its salts are summarized in Table S2.

The most important salt of this acid is mercury(II) fulminate (MF). It was first prepared in the early 17th century, with its explosive properties discovered at the same time [53]. Hg(CNO)_2_ is easily prepared by adding ethanol to a solution of mercury in concentrated nitric acid. The mechanism of its formation is multistep and complex, beginning with the formation of acetaldehyde [11]. The use of MF as an initiator began in the 18th century [56]. The compound’s color and basic properties vary depending on the synthetic procedure and the solvent used for recrystallization [10]. It is sensitive to acids and many other reagents, such as sulfides, thiosulfates, and thiocyanates. It also reacts with copper to form a more friction-sensitive basic copper(II) fulminate. MF is highly toxic and ecotoxic. Generally, it is more sensitive than LA to mechanical shock (0.3–2 J) and discharge (0.5–0.6 mJ) but less sensitive to friction (6.5–7.5 N) [10]. Unlike Pb(N_3_)_2_, MF burns in flame and does not undergo a deflagration-to-detonation transition. During the explosion, metallic mercury, carbon monoxide, and nitrogen are primarily formed. MF is relatively insensitive when wet. Hg(CNO)_2_ was widely used in detonators, primers, detonating cords, and similar applications but is now abandoned due to its toxicity.

Silver fulminate was synthesized around the same time as the mercury(II) analogue. It is more sensitive to stimuli than MF, with sunlight-irradiated samples capable of exploding even upon contact with a feather. It was used in some detonators, as well as in show and toy pyrotechnics. However, its high sensitivity and production costs led to its discontinuation in most commercial applications (except in so-called “bang snaps” produced for Chinese New Year celebrations).

Other fulminates are extremely unstable due to their high sensitivity and tendency to hydrolyze and decompose, so they have not been studied in detail. Some complexes without practical applications have been reported, such as methanolates of Ca^2+^, Sr^2+^, and Ba^2+^ or pyridine complexes of Ag^+^, Cu^+^, Zn^2+^, Cd^2+^, and Hg^2+^ fulminates [57]. Gold forms an interesting, explosive anionic complex, Na[Au(CNO)_2_], but does not form a typical “fulminate” [58] (the so-called “gold fulminate” is actually an azide covalent compound [59]). Tetrazole-derived complexes of silver fulminate, proposed as potential green primary explosives, show greater stability than the parent salt [60] (Table S6). It appears that these complexes have not attracted the interest of non-professionals.

Easy preparation and high sensitivity make MF popular among pyrotechnics enthusiasts and terrorists. However, its production and possession are prohibited in many countries. In 1988, an attack involving an MF-containing bomb was thwarted in New Jersey [61]. Silver fulminate is also very popular among HME producers. The fulminates of copper, cadmium, and other metals are frequently mentioned on DIY pyrotechnics discussion boards.

### 3.3. Polynitrophenolates

**Figure 4:**
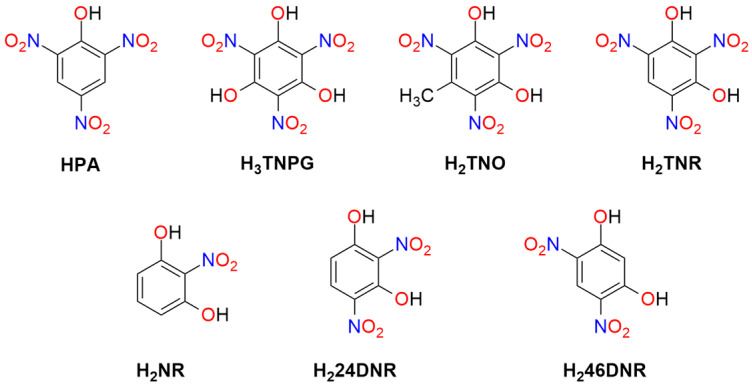
Nitrophenols used in high-energetic materials.

The structures of polynitrophenols used as explosive materials are presented in Figure 4.

#### 3.3.1. Picrates

Commonly known as picric acid (**HPA**), 2,4,6-trinitrophenol is a member of the nitroarene explosives family. It is a yellow, crystalline compound and strong acid (*pKa* = 0.38) [62,63]. Picric acid is easily synthesized by nitration of phenol or 2,4-dinitrophenol [64]. It is a relatively powerful explosive (DV: 7.5 [km s^−1^], Trauzl test: 250–350 mL), slightly more sensitive than 2,4,6-trinitrotoluene (TNT) [15]. It is flammable and does not exhibit a deflagration-to-detonation transition in an unconfined space [15]. Picric acid was first prepared in the 18th century and adopted for military applications at the end of the 19th century. It decomposes, forming carbon oxides, methane, acetylene, nitrogen, hydrogen, water, hydrogen cyanide, and ammonium hydrogen carbonate. Picrates, the salts of picric acid, are generally more sensitive than the parent compound. Several spectacular accidents occurred in the past due to the unforeseen formation of picrates during the industrial production of 2,4,6-trinitrophenol. The basic properties of picric acid and its metal salts are presented in Table S3 (data for anhydrous compounds are provided).

Picric acid forms numerous salts with metal ions, ammonia, and organic bases. Some of these salts are more sensitive than the free acid, making them potential primary explosives. Picrates tend to form hydrates with varying degrees of hydration, which significantly affects their explosive properties compared to anhydrous salts. Hydrates are generally less sensitive or insensitive to friction, impact, and heat. Contaminants in picric acid or picrates are strictly avoided due to the increased risk of explosive accidents. The data on picrates and their explosive properties are somewhat inconsistent. Although hydrated forms exist for all picrates, many reports do not specify the hydration degree of the materials studied, making comparisons difficult. Moreover, even basic physicochemical data, such as HOF or solubility, are unavailable for many picrates. Only ammonium and lead picrates have practical importance in the explosives industry. Ammonium picrate (dunnite) has been used since the early 20th century. Slightly more sensitive than TNT, it has been used in bombs and armor-piercing applications [15]. Dunnite is also a component in pyrotechnic mixtures, e.g., with ammonium or potassium nitrate or ammonium dichromate. Anhydrous lead(II) picrate is highly sensitive to mechanical or thermal stimuli (even more so than MF). Therefore, its hydrates, primarily the monohydrate, are preferred [65]. The primary application of this salt is in the production of electrical squibs and fuseheads. In addition to lead picrate, basic lead picrate is also known, which exhibits lower sensitivity than the standard salt.

Similar to azides and fulminates, copper picrates were synthesized in the form of nitrogen-rich azole complexes and confirmed as explosive compounds [20,66,67,68,69,70,71,72,73]. Picrate salts of Mn(II) and Co(II) complexes have also been studied [74]. Another ligand examined in the metal-picrate and metal-styphnate systems was methylsemicarbazide (**MSC**), with the highest impact sensitivity (IS) detected for the cobalt(II) picrate complex [75] (see Table S6).

Picrates were also prepared in the form of salts with Werner-type cations containing energy-rich ligands, such as –NO_2_ and –N_3_ [46].

**Figure 5:**
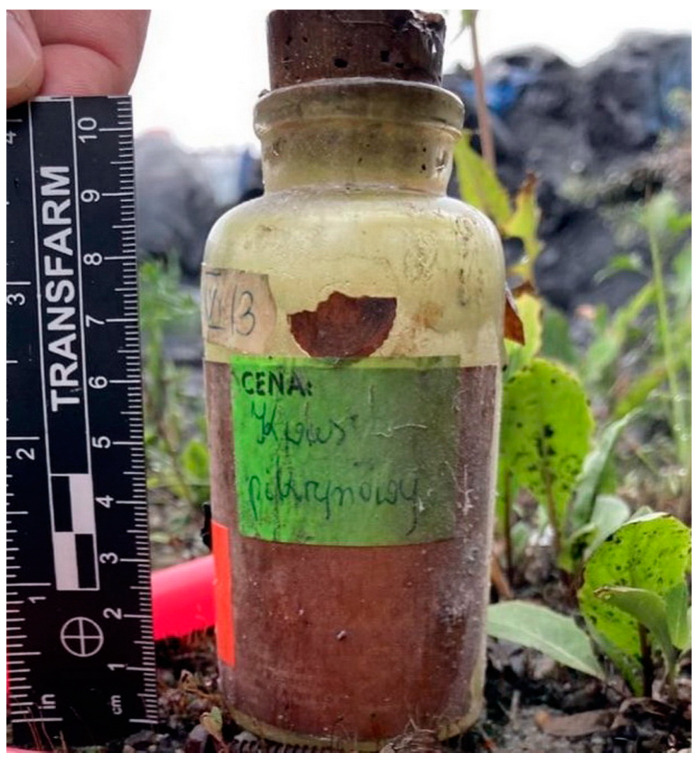
The sample of picric acid found in HME laboratory.

Picrates remain an area of interest for pyrotechnic enthusiasts (Figure 5). Picric acid and some of its salts can be easily obtained from widely available precursors, such as aspirin [76]. Reports on discussion boards and social media reveal that the illegal production of picrates poses a genuine problem and hazard, highlighting the associated risks in HME applications. The most popular picrate-based HMEs are the lead, hydroxylead, cesium, potassium, copper, magnesium, and sodium salts.

#### 3.3.2. Styphnates

Commonly known as styphnic acid (**H_2_TNR**), 2,4,6-trinitro-1,3-dihydroxybenzene (2,4,6-trinitroresorcinol), is a weaker acid than picric acid (*pK_a_* = 1.74) [63]. It can be easily prepared by nitration of 1,3-dihydroxybenzene (resorcinol). Styphnates have been less extensively studied than picrates. Similar to these compounds, styphnic acid salts form numerous hydrates. Additionally, the two acidic hydrogen atoms in the styphnic acid molecule allow for the formation of derivatives with various stoichiometries and mixed salts. Essential information on representative styphnates is presented in Table S3. Among styphnic acid derivatives, only lead and barium salts have practical importance and are well-characterized. Data on other compounds are relatively sparse. Lead(II) styphnate exists in the form of a monohydrate [77,78,79]. This salt is extremely sensitive to flame or electric discharge, with a tendency to explode upon these stimuli considered higher than that of LA or MF. To reduce the risk of explosion during handling, lead(II) styphnate is often desensitized with polymers, waxes, or graphite. While lead(II) styphnate is less sensitive to impact than other lead-containing explosives, its sensitivity increases if the salt is stored at elevated temperatures. Its explosive power, measured with the Trauzl block test, is reported as slightly higher than that of LA, but opinions vary on whether it is higher or lower than MF. It is a weak explosion initiator and is generally considered a poor primary explosive. Lead(II) styphnate is used to initiate other primary explosives, such as LA, in cascade-initiating systems. It is utilized in various blasting caps, pyrotechnic mixtures, and fuseheads.

Several basic lead styphnates are known, but only the monobasic derivative has practical significance [77,79]. This compound forms three polymorphs, which differ in explosive properties. However, reported data are contradictory. The β polymorph contains [Pb_4_(OH)_4_]^4+^ clusters [80], while the structures of the α and γ forms are not known. Some sources describe a red hexagonal form as the most sensitive, while others consider it the least sensitive. All polymorphs are sensitive to electric discharge (though slightly less than the neutral salt). The impact and friction sensitivity is similar to, or even higher than, lead(II) styphnate. Like lead(II) styphnate, the basic salt is used as a primer explosive in mixtures.

Barium styphnate also forms a monohydrate, which is its usable form [81]. The trihydrate has no practical significance. The monohydrate exists in three polymorphic forms. In general, it is less sensitive to impact than the lead analogue. Barium styphnate has applications similar to lead styphnate but is less toxic. Other styphnates are poorly characterized. Hydrates of alkali metal salts (StHM and StM_2_) are explosive and detonate upon heating to 100–130 °C for Na and K, as well as 280–370 °C for Rb [82,83]. Rubidium salts do not explode upon impact or friction [83]. The monocesium derivative shows higher sensitivity to mechanical stimuli than LA. Mono- and dithallium(I) styphnate explode upon heating to approximately 242 °C [84]. Silver styphnate has favorable explosive properties and is a promising primary explosive. However, its cost limits its applications [11,85].

Double salts, such as potassium calcium styphnate, have been studied and reported as explosives [86,87]. Other areas of interest include copper styphnate complexes with triazole and tetrazole ligands, developed as green explosives [20,66,67,68,70,71,72,73,88]. Gruhne et al. synthesized and compared Cu(II), Ag(I), and Zn(II) complexes with novel ligands—nitratoethyltetrazoles (**1NOEtTz**, **2NOETz**)—in the form of various high-energy salts, such as azides, fulminates, nitrates(V), dinitroamidates, chlorates, perchlorates, picrates, styphnates, and trinitrophloroglucinates. The highest impact sensitivity was recorded for fulminate and styphnate, the highest friction sensitivity for azide and perchlorate, and the highest electrostatic discharge sensitivity for azide salts [89]. Detonation properties of semicarbazide (**SCZ**) complexes with Cd, Co, Zn, Ni, and Mn styphnates have also been investigated and confirmed [90,91,92,93].

Tetrazole-derived anions have been used to synthesize double salts containing a styphnate moiety and Pb^2+^ cation. These compounds, with nitrogen-rich ligands, exhibit excellent sensitivity properties and, unlike lead(II) styphnate, efficiently initiate secondary explosives [11,94].

The number of amateur reports and discussions on styphnates is relatively limited, focusing mainly on the easy-to-prepare and highly explosive lead(II) and silver salts. Due to their complex synthesis, styphnate-containing complexes are not commonly produced by non-professionals.

#### 3.3.3. Other Nitrophenolates

Among other nitroarenes containing a hydroxyl group, attention should be provided to 2-nitro-1,3-dihydroxybenzene (2-nitroresorcinol), 2,4-dinitro-1,3-dihydroxybenzene (2,4-dinitroresorcinol), 4,6-dinitro-1,3-dihydroxybenzene (4,6-dinitroresorcinol), 2,4,6-trinitro-1,3,5-trihydroxybenzene (trinitrophloroglucinol), and 2,4,6-trinitro-1,3-dihydroxy-5-methylbenzene (trinitroorcinol). Except for nitroresorcinol salts, these compounds should be regarded as primary explosives.

First, 2-nitroresorcinol (**H_2_NR**) forms a salt with lead(II), which has been used in electric fuseheads in combination with other primary explosives.

Second, 2,4-dinitroresorcinol (**H_2_24DNR**) forms various salts with lead (data on other cations are unavailable). These include normal and basic salts with different degrees of hydration. They are weaker explosives than lead(II) styphnate, with impact sensitivity nearly twice as low (*IS* = 3 vs. 1.7 J) and a brisance of approximately 80% of that of lead styphnate. The advantage of H_2_24DNR salts lies in their safety during handling. These salts are used in similar applications as lead(II) styphnate [95].

Also, 4,6-dinitroresorcinol (**H_2_46DNR**) is the parent acid for a family of lead(II) salts with various compositions. In addition to normal and basic forms, it also forms an acidic salt. Similar to the Pb^2+^ derivatives of 2,4-DNR, the 4,6-DNR salts are less explosive than styphnates [11,95,96].

Trinitrophloroglucinol (**H_3_TNPG**) forms explosive monopotassium, dipotassium, and tripotassium salts, of which the most sensitive is the monopotassium derivative (detonation upon impact at 1.12 J) [97]. Explosions upon friction or impact have also been reported for the sodium and cesium salts [98,99]. Additionally, salts of this acid with copper azole complexes have been reported as explosives [66].

The only reports on trinitroorcinol (**H_2_TNO**) salts describe the explosive nature of its salts with Zn, Cs, Pb, and azole complexes of Zn and Cu. Simple salts show high impact sensitivity (<1 J). Lead trinitroorcinol (PbTNO) is extremely sensitive to friction (<0.1 N, compared to 0.45 N for lead(II) styphnate) and spark discharge (0.54 mJ) [100,101].

### 3.4. Acetylides

Ethyne (acetylene) forms numerous salts with various cations, with the parent hydrocarbon acting as a weak acid. Many of these salts (e.g., alkali or alkaline metal derivatives) decompose in water. The explosive properties are confirmed for heavy-metal acetylides, which are easily prepared by reacting acetylene (e.g., obtained from the reaction of calcium carbide, CaC_2_, with water) with metal salts [102]. Available data on acetylene-derived explosives are rather sparse; most of these compounds are merely noted as dangerous, often without detailed information on their sensitivity or thermodynamic properties.

Silver acetylide (Ag_2_C_2_) forms adducts with silver(I) nitrate(V) and other Ag^+^ salts [103,104,105,106]. The composition of the resulting products can be controlled by adjusting the concentration of the starting solution, silver salt excess, and the pH of the reaction mixture [107]. Ag_2_C_2_ was first synthesized by Berthelot in 1866 [108]. Both silver acetylide and its 1:1 adduct with AgNO_3_ are explosive [109]. Simple salt is more sensitive than the complexes and undergoes violent explosion upon impact and friction [10,110]. Compared to MF, silver acetylide is equally sensitive to impact but more easily initiated by friction [10,110]. The sensitivity of the solid salt increases with storage. Some reports indicate that the energetic properties of Ag_2_C_2_ depend on the synthesis conditions. Upon heating, it ignites at 140–200 °C.

The 1:1 silver(I) acetylide-silver(I) nitrate(V) adduct is reported to be equally or slightly less sensitive than LA [10,12,110]. Similar to Ag_2_C_2_, this compound shows a distinct relationship between its properties and synthesis conditions. Its ignition efficiency for initiating tetryl or TNT is comparable to that of LA. Ag_2_C_2_·AgNO_3_ has been proposed as a light-sensitive explosive [111,112,113,114]. Among the other complexes studied, the 1:6 adduct is non-explosive, while the 1:5.5 complex decomposes violently upon friction [104,107,115].

Copper(I) acetylide crystallizes as either a monohydrate or in an anhydrous form, though the monohydrate has practical applications [116,117]. It is relatively insensitive to impact (similar to PETN) but highly responsive to sparks [10,109,118]. Copper(I) acetylide is used in electric fuseheads [119]. In contrast, copper(II) acetylide is extremely sensitive to stimuli, making it crucial to avoid contamination of Cu_2_C_2_ with the Cu(II) analogue [118]. The formation of copper acetylides in industrial plants and laboratory apparatus has led to numerous accidents in the past [120,121,122].

Other acetylides (Au(I), Hg(I), Hg(II)) have been poorly investigated. An interesting example is mercury(II) diacetylide, which detonates upon heating (DV: 10.5 [km s^−1^], explosion temperature: 287 °C, HOF: 408.5 [kJ mol^−1^]). However, no detailed data on its mechanical sensitivity have been provided [123].

Poly(acetylene) salts of several cations are also reported to be unstable, and some may explode [124].

An interesting series of compounds have been synthesized from 1- and 2-propargyltetrazole (**1PryTz**, **2PryTz**). These compounds act as ligands and coordinate with metal ions via the *N*^5^ position in various perchlorates, polynitrophenolates, and dicyanamides, or they serve as counter-ions, such as acetylide [125].

Easily accessible acetylides are widely used in homemade pyrotechnics and explosives. Numerous syntheses of silver, copper(I), and copper(II) acetylides are discussed on online forums, social media, and in samizdat publications [126] and are regarded as attractive demonstrations for chemistry lectures [127,128].

### 3.5. Furoxans and Furazans Salts

**Figure 6:**
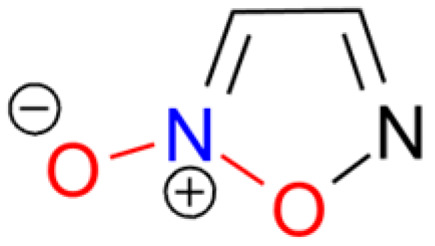
Furoxan—“hidden” nitro group as a part of a ring.

Furoxan (1,2,5-oxadiazole 2-oxide; HOF 226 [kJ mol^−1^]) derivatives are known explosive compounds, and this moiety can be considered a “hidden” nitro group (Figure 6).

**Figure 7:**
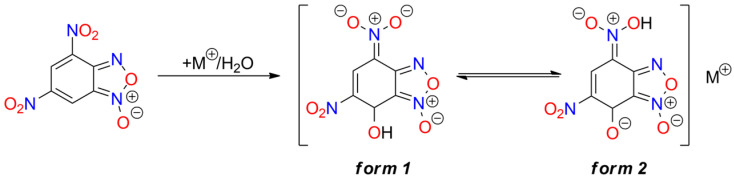
Tautomerism in Meisenheimer complex of DNBF molecule.

Among the studied furoxans, some metal salts are also recognized as high-energy materials. Generally, these salts exhibit higher sensitivity than the parent furoxans. Also, 4,6-Dinitrobenzofuroxan (**DNBF**) is the most important and most extensively studied furoxan explosive. It is difficult to initiate by various stimuli (its sensitivity is comparable to RDX and higher than PETN), and, therefore, it is classified as a secondary explosive [129]. The synthesis of DNBF is straightforward; for example, using 2,4,6-trinitrochlorobenzene as the starting material and NaN_3_ as a reagent [130,131]. Numerous salts of this compound have been synthesized, but only the potassium salt is used in ammunition as a primary explosive—a green alternative to lead- or mercury-based primers [132,133,134]. Cation binding by DNBF is possible due to the formation of a Meisenheimer complex with water, alcohol, or amine (Figure 7) [135,136,137]. This reaction occurs in slightly alkaline solutions, such as those containing metal carbonate or bicarbonate. The resulting anion exists in two resonance structures, and the presence of both tautomers has been confirmed in the solid form of DNBF metal adducts.

Analysis of IR spectra indicates that the predominant anion tautomer depends on the counterion: *form 1* is found in salts with alkali and alkaline earth metals, Mn(II), Co(II), Ni(II), Zn(II), and Cd(II), while *form 2* is present in complexes with Pb(II), Cu(II), and trivalent cations of Fe and Cr [138].

The potassium salt of DNBF has intermediate sensitivity between LA and MF (KDNBF explodes upon an impact of 7 J, friction < 5 N, discharge sensitivity at 6 mJ, or when heated to 200–220 °C) [10,139,140,141,142,143]. However, Jones et al. indicate that more studies are required for definitive conclusions [139]. Its properties clearly depend on crystal morphology, which can be modified by adding surfactants to the reaction mixture [144,145] or by altering reaction conditions [146]. KDNBF is a weaker initiator than MF. The sodium, rubidium, and cesium salts exhibit similar impact and discharge sensitivities but decompose explosively at lower temperatures (160–190 °C compared to 200–220 °C for KDNBF) [142]. The Rb and Cs derivatives are more sensitive to friction, while the Na salt is less sensitive [140,147,148,149].

Data on transition metal adducts are limited. Shinde et al. reported that Cr(III), Fe(III), and Cu(II) salts are less sensitive to friction, impact, heat, and discharge than KDNBF [148,150]. Silver and barium DNBF derivatives are less sensitive to electric discharge than the K and Na salts. AgDNBF initiates upon impact (with sensitivity similar to its potassium analogue) but has poor propagation properties, making it unsuitable as a primary explosive. Barium salt is significantly less sensitive [151].

**Figure 8:**
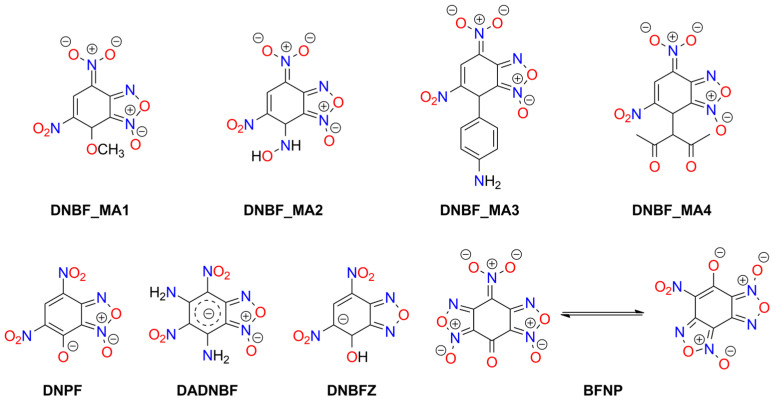
The structures of explosive bicyclic and tricyclic furoxanes.

Meisenheimer 4,5-dinitrobenzofuroxan adducts, such as those with methanol (**DNBF_MA1**) [152,153,154], hydroxylamine (**DNBF_MA2**) [154,155], aniline (**DNBF_MA3**) [156,157], dimethylaniline [157], phenol [157], or acetylacetone (**DNBF_MA4**) [158], form salts exhibiting explosive properties (see Figure 8). Available physicochemical data for these compounds are very limited. Thermogravimetric (TG) studies indicate lower thermal stability than DNBF, except for the aniline-derived compound. The hydroxylamine analogue is more easily initiated by impact than KDNBF (2.7 J) [11].

Other benzofuroxan explosives mentioned in the literature include potassium salts of 7-hydroxy-4,6-dinitrobenzofuroxan (**HDNPF**), bis(furoxano)-2-nitrophenol (**BFNP**), Meisenheimer adducts of 4,6-dinitrobenzofurazan (**DNBFZ**), and 5,7-diamino-4,6-dinitrobenzofuroxan (CL-14; **DADNBF**). KDNPF is more stable than KDNBF. Its impact sensitivity is comparable to or higher than LA (depending on the KDNP crystal form), but it is distinctly less sensitive to friction [159,160]. Its Na^+^ analogue has also been reported [161].

The potassium salt of BFNP is notable because two isomeric forms of the anion exist in the solid state, whereas in the crystals of Na^+^, Rb^+^, and Cs^+^ salts, only one tautomer of BFNP^−^ occurs. NMR studies of all these salts in solution confirm an equilibrium between both structures. KBFNP demonstrates superior heat, impact, and friction sensitivity compared to lead styphnate [162,163,164,165].

CL-14 salts of alkali metals are less sensitive to thermal and mechanical stimuli than analogous DNBF derivatives. Within the series of CL-14-derived materials, the Cs^+^ adduct shows the highest mechanical sensitivity, while the Na^+^ adduct is the most thermally unstable [149]. KDNBFZ is explosive, but it has a low ignition temperature (179 °C), making it unsuitable as a commercial explosive material [132,153]. Incidentally, the explosivity of some related compounds, studied for other purposes, has also been reported [166].

**Figure 9:**
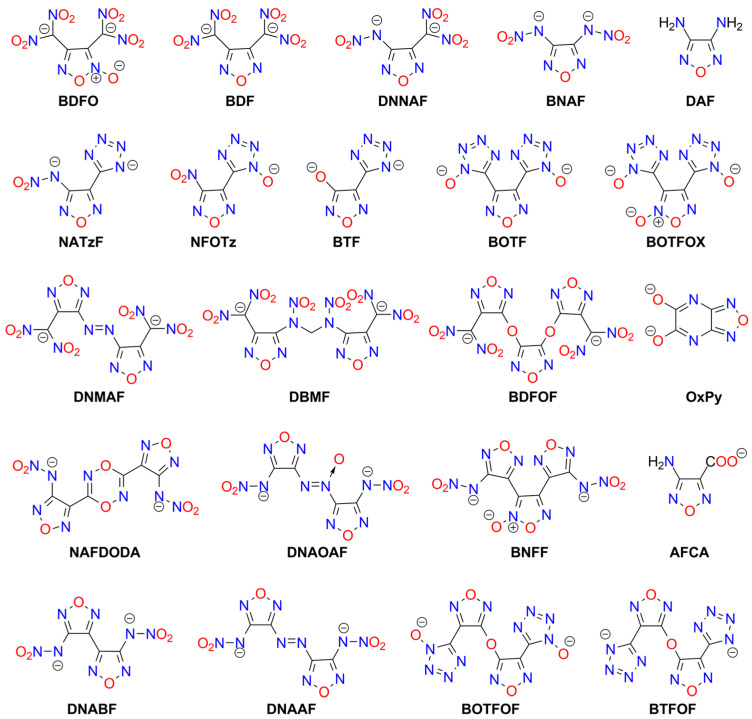
Furoxans and furazans used as anions in metal-containing explosives.

Other classes of furoxan- and furazan-derived explosives include compounds containing the 3,4-disubstituted 1,2,5-furazan ring and dinitromethyl moieties (Figure 9). The simplest example is potassium 3,4-bis(dinitromethyl)furoxanate (**K_2_BDFO**), synthesized by He and Shreeve. The impact sensitivity and detonation velocity of this salt are higher than those of LA, but it is less sensitive to friction [167]. A similar derivative containing a furazan ring is less sensitive than **K_2_BDF** [168].

Other compounds containing two or more 1,2,5-oxadiazole rings have also been prepared. The furoxan/furazan units can be coupled via diazo (e.g., [169,170]) or ether bridge [171]. Other possible acidic centers include nitroamino [172] or tetrazole/N-hydroxytetrazole groups [172,173,174,175,176,177]. Additionally, a mixed furoxan/furazan 1:2 silver/potassium 2:4 salt has been synthesized and characterized [178].

The **BTFOF** anion offers a unique opportunity to construct highly energetic metal–organic framework (MOF) polymers. An Ag^+^-containing construct based on this anion has been reported as a material less sensitive than RDX (IS > 40 J, FS > 360 N) but with energetic properties similar to those of other tetrazole complexes. Notably, it features an exceptionally high detonation velocity of 11.8 [km s^−1^] [179]. In the presence of Pb^2+^ ions, BTFOF decomposes, forming **BTF** molecules. MOFs based on this anion have been synthesized and characterized [180]. A similar high-energy MOF was obtained using **DNMAF** [181].

Also, 3-aminofurazan-4-carboxylic acid (**AFCA**) has been utilized to prepare furazan-containing transition metal MOFs (Co, Ni, Mn, Fe, Cu) [182]. Sun et al. reported explosive alkali metal salts with furoxan rings linked by an *N*,*N′*-dinitromethylenediamine bridge (**H_2_DBMF**). For these compounds, sensitivity to all types of stimuli increases with the cation diameter (Cs^+^ > Na^+^), while DV exhibits the opposite trend [183]. Nitroamine-modified furazans (**DNABF**, **DNAAF**, **DNAOAF**, **BNFF**) have been studied in the form of various salts by Fischer et al. [184] and Gospodinov et al. [185].

In addition, 1,2,5-oxadiazolo[3,4-d]pyrimidine (**H_2_OxPy**) polymers with Na^+^, K^+^, and Cs^+^ have been reported as insensitive compounds with good detonation properties [186].

For further data on furoxan and furazan explosives, see the comprehensive reviews by Fershtat and Makhova [187] and Wang et al. [188].

Due to the multi-step syntheses involved, furoxan and furazan derivatives are not popular among HME producers. Only sparse discussions on these compounds can be found on forums, such as the ScienceMadness discussion board (e.g., https://www.sciencemadness.org/whisper/viewthread.php?tid=439 (accessed on 12 September 2024).

### 3.6. Tetrazole Salts and Complexes

**Figure 10:**
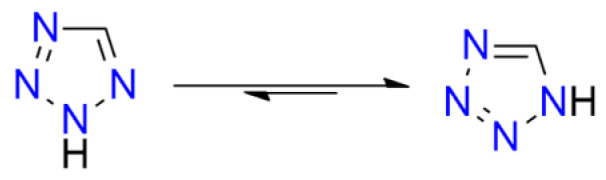
Tautomerism of tetrazole (**HTz**).

Tetrazole (**HTz**; HOF: 236 kJ/mol) is an energy-rich molecule (Figure 10). It can explode upon heating above its melting point [189].

Tetrazole is the parent molecule of numerous explosives. This diversity is possible due to several modes of tetrazole binding:

- Tetrazole is a weak N-acid (*pK_a_* = 4.90) and can form various high-energy salts;
- The tetrazole molecule can be modified by substitution at three different positions;
- Tetrazole is a good ligand, capable of binding to metal cations via the *N*^2^ or *N*^3^ atoms.

**Figure 11:**
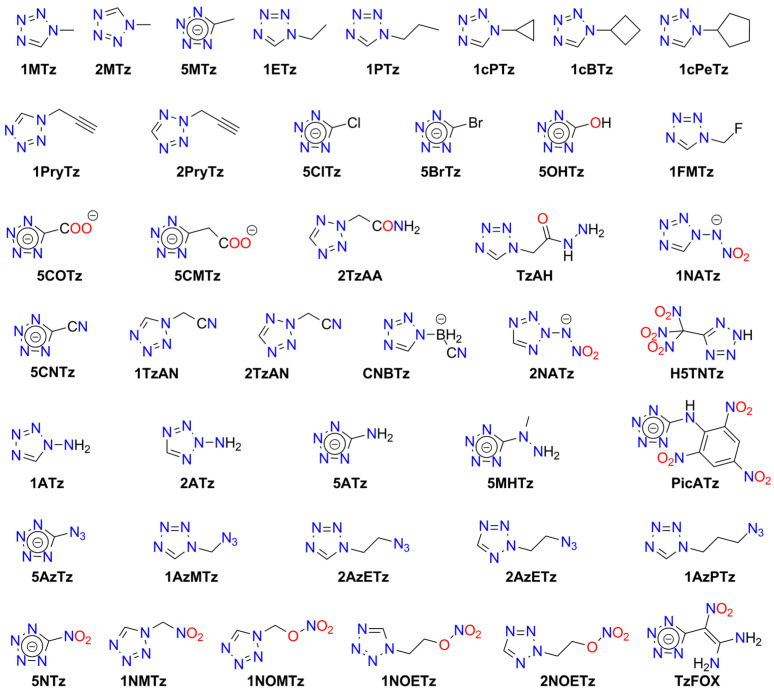
Monosubstituted tetrazoles used in metal-containing explosives.

Explosive properties are reported for organic tetrazole derivatives, salts, and complexes (Figure 11). Even salts of “simple” 1*H*-tetrazole (**HTz**) can explode upon heating and/or mechanical stimuli, such as silver(I) tetrazolate [190], barium tetrazolate [191], mercury(II) 1-methyl-5*H*-tetrazolate (**1MTz**; a metalloorganic compound with an Hg-*C*^5^ bond) [192], copper(II) 5-bromo-1*H*-tetrazolate (**H5BrTz**), copper(II) 5-chloro-1*H*-tetrazolate (**H5ClTz**) [193,194], and dimercury(I) 5-hydroxy-1*H*-tetrazolate (**H5HOTz**) [194]. Also, 5-methyl-1*H*-tetrazole (**H5MTz**) forms a 3D MOF compound with Cu(I) ions, which explodes upon impact (36 J) [195]. In contrast, some adducts of 1*H*-tetrazole are non-explosive, such as alkali metal tetrazolates [196].

More powerful and extensively studied are salts containing “nitrogen-enriched” tetrazole molecules, i.e., derivatives with amino, nitro, diazo, azido, and similar moieties.

For example, 5-amino-1H-tetrazole (**5ATz**) forms numerous explosive salts with metal cations. Impact sensitivity has been confirmed for salts with Ag^+^, Hg_2_^2+^, and Cu^2+^, with sensitivity levels ranging from 2.5 kg at 22–68 cm. Other salts ignite when heated and may explode upon ignition (e.g., Co^2+^, Ni^2+^). No sensitivity to mechanical stimuli or discharge has been observed for alkali (except the Li^+^ derivative) and alkaline earth 5ATz salts [191,197,198].

Also, 5-nitro-1*H*-tetrazole (**5NTz**) is an extremely sensitive material; the electron-withdrawing nitro substituent increases its acidity (*pK_a_* = −0.80) [199]. The alkali metal salts of 5NTz are also sensitive, although their sensitivity is lower than that of the parent azole. The highest sensitivity is observed in Rb5NTz (*IS* = 5 J, *FS* < 5 N), while the lowest sensitivity is found in the sodium salt, which crystallizes as a dihydrate (*IS* > 30 J, *FS* ≈ 360 N). These complexes are potential replacements for toxic primary explosives [200]. The alkaline earth salts of 5NTz are also sensitive but less so than the alkali metal salts (e.g., Ba: *IS* = 2.5–5 J, *FS* < 20 N; Mg: *IS* ≥ 40 J, *FS* = 240 N). These derivatives typically form as penta- and hexahydrates and are highly explosive if dehydrated, with reported instances of uncontrolled detonations [201].

Other metal salts of 5NTz have been studied less systematically. Detailed data are available for mercury(II) [202,203,204,205,206], dimercury(I) [202], silver(I) [132,202,203,204,207], copper(I) [133,208,209], and copper(II) [203,207,209]. These salts are considered promising primary explosives, with sensitivity similar to that of MF and greater power than LA, showing good initiating properties. Von Herz also reported Co^2+^ and Ni^2+^ adducts, though their structures were not detailed [203]. Cu5NTz, marketed as DBX-1, is recognized as an environmentally friendly primary explosive. Its properties make it a viable substitute for lead-containing initiators (such as LA and LSt) and MF [208,210,211]. The energetic properties of Cu5NTz depend on the quality of its precursor, sodium 5-nitrotetrazolate [212]. Some double and basic salts and derivatives with complex cations have also been reported. Lead(II) forms basic salts with the stoichiometry Pb(5NTz)_2_·Pb(OH)_2_, which is less sensitive than MF [203]. The basic salt of Cu^2+^ (Cu(5NTz)_2_‧2Cu(OH)_2_) is a poor explosive [213]. Copper(II) also forms an adduct with the 5NTz molecule: Cu(5NTz)_2_·H5NTz, which is reported as either less sensitive than Cu(5NTz)_2_ and other simple 5-nitro-1*H*-tetrazolates [7,132] or as extremely sensitive [207].

Klapötke and Sabaté proposed the synthesis of 5NTz derivatives with ammonia and amine Ag^+^ and Cu^2+^ complexes: [Cu(en)_2_](5NTz)_2_, [Ag(en)](5NTz), [Cu(NH_3_)_3_](5NTz)_2_ (where en = 1,2-ethylenediamine). The copper complexes are extremely sensitive to impact (<7 J), while the silver complex is less hazardous (*IS* > 40 J). All these compounds are less sensitive to friction than LA [207]. Additionally, 1,2-ethylenediamine and 1,3-diaminopropane (dap) Cu(II) complexes were reported by Bates and Jenkins [202].

Sodium and ammonium salts of tetrakis(5-nitro-1*H*-tetrazolato)diaquaferrate(II) ([Fe(5NTz)_4_(H_2_O)_2_)]^2−^) or tetrakis(5-nitro-1*H*-tetrazolato)diaquacopprate(II) ([Cu(5NTz)_4_(H_2_O)_2_)]^2−^) have also been synthesized. These complexes explode upon impact (2.5 kg/12 cm), friction, and spark; their overall sensitivity is lower than that of LA or LSt [214]. A series of iron(II) complex salts, [Fe(5NTz)_n_(H_2_O)_6-n_] where *n* = 3–6, were also studied. The *IS* and *FS* values increase with the number of 5NTz ligands (from 15 to 8 J and 4.2 to 0.8 N, respectively) [215].

Hiskey et al. patented ammonium and alkali metal salts of anionic complexes with divalent cations: [Co(5NTz)_3_(H_2_O)_3_]^−^, [Ni(5NTz)_4_(H_2_O)_2_]^2−^, [Cu(5NTz)_4_(H_2_O)_2_]^2−^, [Fe(5NTz)_4_(H_2_O)_2_]^2−^. Among them, the highest *IS* was reported for the ammonium salt of the Ni complex (approximately twice that of LA), and the highest *FS* for the ammonium copper derivative (one-tenth that of PETN) [216].

Nitrile derivative, 5-cyjano-1*H*-tetrazole (**H5CNTz**) has been less extensively studied. Cesium, potassium, and sodium salts have been reported as stable [217,218], and they serve as precursors for salts with nitrogen-rich cations. The silver(I) salt is reported to be explosive, with an impact sensitivity of less than 1 J [219].

An acid, 5-carboxy-1*H*-tetrazole (**H5CATz**) forms hydrated salts and coordination compounds with various metals. Among the studied compounds, only the Cu(II) and Mn(II) complexes are slightly sensitive to friction but show no impact sensitivity. Other tested derivatives (Sr, Ba, Ag) are insensitive [220].

Also, 5-azido-1*H*-tetrazole (**H5AzTz**) is explosive but, according to many authors, less sensitive than its salts [7]. The 5AzTz anion has the highest nitrogen content among tetrazole derivatives. All reported salts are extremely sensitive to various types of stimuli and are effective initiators of secondary explosives. However, they are considered too sensitive for technical applications. The parent compounds and their salts can be synthesized through various routes, such as from 5-amino- or 5-hydrazino-1*H*-tetrazole or via reaction with cyanogen bromide and azides. Many of the metal salts form hydrates, which remain extremely explosive. Reported *IS* values for alkali metal salts are <1 J, with *FS* ranging from 11 N (Li salt) to <5 N (Na); K and Cs derivatives are considered too sensitive to handle. For the Ca complex, [Ca(5AzTz)_2_(H_2_O)_10_]·{[Ca(H_2_O)_6_](5AzTz)_2_} the authors report *IS* and *FS* values of 2 J and 25 N, respectively [221]. Additionally, Ag5AzTz is highly explosive and can detonate in warm water or upon handling, even when wet [222].

**Figure 12:**
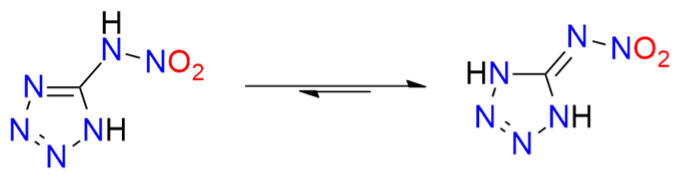
Tautomerism of 5-nitroamino-1*H*-tetrazole (**H_2_5NATz**).

**Figure 13:**
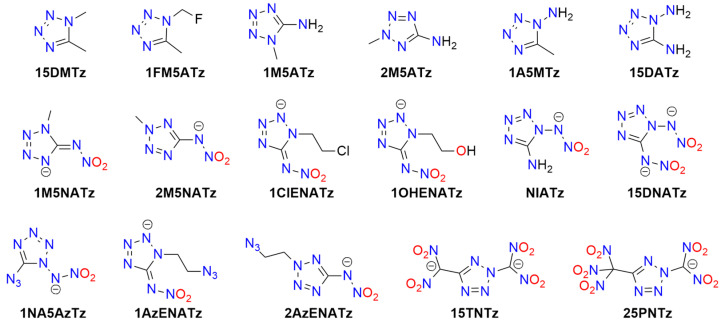
Disubstituted tetrazoles used in metal-containing explosives.

Another tetrazole derivative used in high-energy materials is 5-nitroamino-1*H*-tetrazole (5-nitrimino-1,4*H*-tetrazole; **H_2_5NATz**); it can form a dianion due to the presence of two acidic N-H protons (Figure 12). Surprisingly, the salts of H_2_5NATz have been moderately studied in the context of their suitability in pyrotechnics. Sodium salts have been reported as explosive [223,224]. Its nickel complex, [Ni(NH_3_)_3_(5NATz)]_2_, is also reported to be explosive upon impact and heating [225]. More detailed information is available for Ba^2+^ and Ca^2+^ salts. Both Ba5NATz and Ba(5NATz)_2_ are explosive, with their sensitivity depending on the degree of hydration. The most sensitive form is Ba(5NATz)_2_ tetrahydrate (*IS* = 7 J; *FS* = 250 N). A 2-methyl-substituted Ba(5NATz)_2_ analogue is also explosive [191]. Anhydrous Ba(5NATz)_2_ is highly sensitive to impact and friction, with stimulation parameters similar to LA [226]. Strontium salts are less sensitive; for example, Sr(5NATz)_2_·4H_2_O has *IS* = 20 J, *FS* = 288 N [227]. Both hydrated and anhydrous forms of calcium salt, Ca5NATz, explode upon impact (5 J for the anhydrous form), friction (112 N), and spark, as well as deflagrate upon heating, showing good initiating properties [228]. Klapötke et al. proposed a series of copper complexes of 5NATz and its 1-methyl and 2-methyl analogues (**1M5NATz**, **2M5NATz**). These compounds represent a large family of disubstituted tetrazole, used in ECC (Figure 13). Among the synthesized compounds, three—[Cu(2M5NATz)_2_(H2M5NATz)_2_], Cu(2M5NATz)_2_, and Cu(1M5NATz)_2_—exhibit high impact sensitivity (0.7–2 J). Other complexes detonate after an impact of 15 J or more [229,230].

Silver and alkali metal salts of 1-methyl-5-nitroaminotetrazole (**1M5ATz**) are almost insensitive (*IS* > 50 J) [231,232]. Benz et al. proposed a modified nitraminotetrazole substituted with a 2-azidoethyl group (**2AzENATz**) as a melt-cast material. The parent N-acid and its potassium and silver salts were characterized; both salts are significantly more sensitive to mechanical stimuli and spark but exhibit greater thermal stability [233]. Another compound synthesized by Benz and co-workers is 1-nitramino-5-azidotetrazole (**1NA5AzTz**). The potassium salt of this compound was prepared, although its characteristics were not presented [234].

Other tetrazoles have been studied less comprehensively. For example, 5-Carboxymethyl-1*H*-tetrazole (**H5CMTz**) forms an explosive MOF network with Ag^+^ cations; this compound has low sensitivity but high detonation energy, similar to RDX [235]. Another example is 1,1-diamino-2-nitro-2-(1*H*-tetrazol-5-yl)ethene (**TzFOX**), which forms MOF polymers with copper(I) (*IS* = 10 J). This complex is thermally stable (up to 300 °C) and demonstrates good initiating properties [236]. The potassium salt of TzFOX shows higher sensitivity [237].

Also, 1,5-Bis(nitramino)tetrazole (**H_2_15DNATz**) forms a highly explosive and sensitive potassium salt (*IS* < 1 J, *FS* < 5 N) [238]. The same sensitivity was reported for silver(I) 5-amino-1-nitriminotetrazolate (**NIATz**) [239]. Mixed salts of H_2_15DNATz containing transition metal cations were studied by Li et al. All the compounds prepared exhibit high sensitivity, and the authors recommend them as “green primary explosives” [240].

Potassium 1-nitriminotetrazolate (**1NATz**) and potassium 5-amino-1-nitriminotetrazolate are highly sensitive, detonating after impacts of <1 J and under friction of <0.1 and 2 N, respectively [241]; the 1-substituted NATz isomer is more sensitive than the 2-isomer (1 vs. 5 J) [242]. The copper(II) complex of 5-(1-methylhydrazynyl)-1*H*-tetrazole (**H5MHTz**), {[CuCl(µ_3_-Cl)(H5MHTz)]·H_2_O}, detonates after impact (40 J) but is less sensitive than the perchlorate and nitrate salts of Cu(II) complexes with this ligand [243]. The same ligand was studied by Tao et al., who characterized simple salts with Cu^2+^ and Ag^+^, both of which are insensitive to impact (>40 J) [244].

Explosion-related physicochemical data have also been published for the potassium salt of 1-(2-azidoethyl)-5-nitroaminotetrazole (**1AzENATz**), the sodium salt of 1-(2-chloroethyl)-5-nitroaminotetrazole (**1ClENATz**), and copper(II) complexes of these ligands, as well as with 1-(2-hydroxyethyl)-5-nitroaminotetrazole (**1OHENATz**) [245].

**Figure 14:**
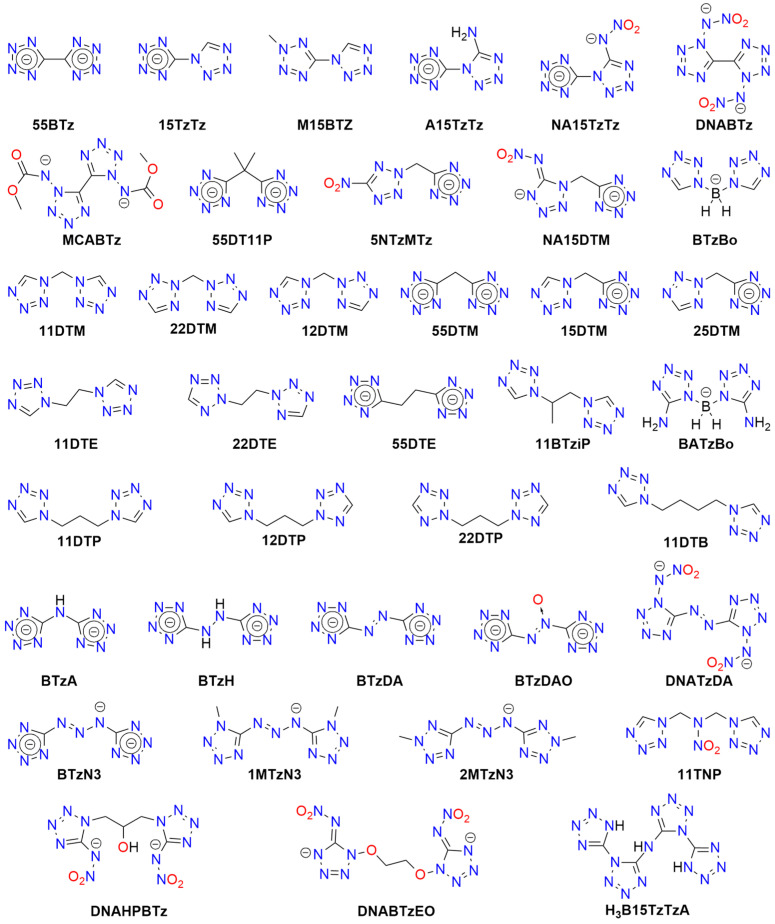
Dimeric and bridged tetrazoles used in metal-containing explosives.

A separate group of compounds includes dimeric tetrazoles, composed of two CN_4_ rings connected via a bridging moiety (Figure 14). For 5,5′-bitetrazole (**H_2_BTz**), data are available on hydrated earth metal salts [246], sodium, and diaquacopper(II) [247]; the latter forms a polymeric structure. Only BaBTz and the Cu^2+^ complex are considered impact-sensitive, with the latter also being friction-sensitive. Fischer et al. also reported an isomeric dimer, 5-(tetrazol-1-yl)-2*H*-tetrazole (**H15TzTz**) and its 2-methyl derivative (**M15BTZ**). Reported Cu^+^ and Ag^+^ salts are extremely sensitive, while CuCl_2_ coordination compounds and hydrated sodium and potassium salts are almost insensitive [248].

Wang et al. synthesized the 5-nitroamino derivative of H15TzTz (**H_2_NA15TzTz**) and its potassium salt. In the same study, they reported the K^+^ derivative of 5-amino-H15TzTz (**HA15TzTz**); both compounds are impact- and heat-sensitive [249]. The family of bridged tetrazole compounds also include amine (**H_2_BTzA**) [250,251,252,253], hydrazine (**H_2_BTzH**), diazo (**H_2_BTzDA**) [254,255,256] (numerous salts reported as insensitive), diazo-*N*-oxide (**H_2_BTzDAO**) [257], triazine (**HBTzT**) [258], and tetrazine (**H_2_BTzTt**) [259] linker-containing acids, as well as the salts derived from them.

Several Werner-type complexes were prepared by Deblitz et al., who studied diazo-bridged bis-tetrazole (**BTzDA**) salts with mixed nitrito (and azido) amminacobalt(III). These compounds show impact sensitivity (3.5–10 J) and friction sensitivity (>216 N) [46]. Diazo-N-oxide derivatives are less thermally stable than the parent diazotetrazole. A major issue observed in these salts is the formation of highly hydrated crystals, resulting in low sensitivity. However, dehydration is often difficult and may lead to decomposition.

H_2_BTzA was intensively studied as a ligand for synthesizing MOF materials with energetic properties. Some are less sensitive to impact (e.g., [Co_9_(BTzA)_10_(HBTzA)_2_(H_2_O)_10_]_n_, PbCu(BTzA)_2_), while others are classified as insensitive, such as [Cd(BTzA)(H_2_O)]_n_ or [Pb(BTzA)(H_2_O)]_n_ [260,261,262,263]. An anionic BTzA complex with Zn^2+^, [Zn(BTzA)_2_(H_2_O)]^2−^, has been combined with Li^+^ and various nitrogen-rich cations, including ammonium, hydrazinium, hydroxyammonium, guanidinium, and *N*-(mono-, di- or tri-)aminoguanidinium. All studied materials are insensitive to impact (>40 J), friction (>360 N), and electric discharge and decompose thermally above 200 °C [264].

Detailed data have been published for various salts (alkali and alkaline earth metals, Ag(I)), and amminacomplexes of Ni(II), Cu(II), Zn(II)) for the 1,1′-di(nitramino)-5,5′-bitetrazole dianion (DNABTz). Unfortunately, alkaline earth metal salts are unsuitable for explosivity tests. The alkali salts exhibit high sensitivity, increasing with cation size, and all show a rapid deflagration-to-detonation transition. Sodium forms two polymorphs with different coordination modes and physicochemical properties. Among studied derivatives, the potassium salt is the most promising as a primary explosive due to its high thermal stability and excellent explosive properties [265,266]. The parent acid is less impact-sensitive but has a lower decomposition temperature [267].

Other studied salts include a 1-nitroaminotetrazole unit bridged with 2-hydroxypropylene (**H_2_DNAHPBTz**) [268] and diazo linker (**H_2_DNATzDA**) [267]. He et al. reported a diimide sodium salt derived from 5,5′-bis(1-methoxycarbamido-5*H*-tetrazole) (**MCABTz**). However, it is insensitive, in contrast to the parent acid [269]. Additionally, triazene-linked 1-methyl- or 2-methyl-5*H*-tetrazole (**H1MTzN3**, **H2MTzN3**) salts with sodium cations are surprisingly stable and insensitive, especially compared with protonated analogs [270].

Also, 5-(5-nitro-5*H*-tetrazol-2-ylmethyl)-5*H*-tetrazole (**H5NTzMTz**) forms highly explosive alkali metal salts, a silver salt, and copper(II) complexes, with the highest sensitivity observed in the lithium and silver derivatives [271]. Interestingly, another methylene-bridged compound, the K^+^ salt of 1-(5*H*-tetrazo-5-ylmethyl)-2-nitroimino-1*H*,5*H*-tetrazole (**H_2_NA15DTM**), is insensitive [272], while the corresponding N-acid is explosive. A dimeric 5-nitroiminotetrazole containing ethylenedioxy linker (**H_2_DNABTzEO**) forms an explosive silver salt, though details of its sensitivity were not provided [273].

A series of bismuth-containing explosives was synthesized from Na_2_BTzDA, Na_2_BTz, Na_2_BTzH, and Na_2_BTzN_3_ and compared with those derived from KNATz and NaNTz. All studied materials contain various oxobismuth(III) or hydroxybismuth(III) cations and clusters. However, exact crystallographic structures were not determined. They explode upon heating, with the dimeric tetrazole-derived compounds being friction-sensitive [274].

**Figure 15:**
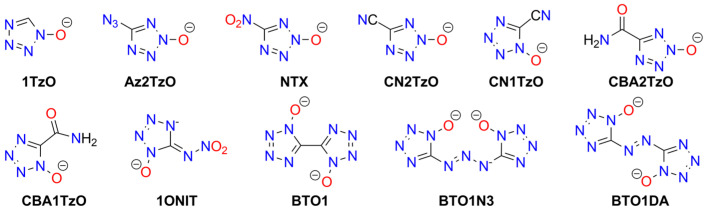
Tetrazole *N*-oxides used in metal-containing explosives.

Besides tetrazoles, their *N*-oxides and *N*-hydroxides have also been synthesized and studied (Figure 15). The simple 1-*N*-hydroxide anion (**1TzO**) was examined in the form of Ag^+^, Na^+^ (two polymorphs), and K^+^ adducts; Ag1TzO is the most sensitive and may be used as a primary explosive due to its high initiation ability against common secondary explosives [275]. Data on analogous 2-N-hydroxy derivatives are not available.

Among other monomeric N-oxides or N-hydroxides, Klapötke et al. reported salts of 2-hydroxy-5-azido-2*H*-tetrazole (**HAz2TzO**; Na^+^, K^+^, Ag^+^) [276], 2-hydroxy-5-cyjano-2*H*-tetrazole (**HCN2TzO**; Na^+^, Ag^+^), 1-hydroxy-5-cyjano-2*H*-tetrazole (**HCN1TzO**; Ag^+^), 2-hydroxy-5-carboxamido-2*H*-tetrazole (**HCBA2TzO**; Cu^2+^), and 1-hydroxy-5-carboxamido-2*H*-tetrazole (**HCBA1TzO**; Cu^2+^). The salts of the carboxamide-derived ligands are insensitive [277,278]. They also reported 2-hydroxy-5-nitro-2*H*-tetrazole (**HNTX**; Ag^+^, Na^+^-Cs^+^, Ca^2+^-Ba^2+^) [279] and 1-hydroxy-5-nitroiminotetrazole (**H_2_1ONIT**; K^+^, which is insensitive) [280].

Among bridged and dimeric tetrazole molecules, metal salts were reported for 5,5′-bis(tetrazole-1-hydroxide) (**H_2_BTO1**; Li^+^, Na^+^, K^+^, Cs^+^, Ca^2+^-Ba^2+^). All hydrates formed are low-to-moderately sensitive to mechanical stimuli, while anhydrous salts are highly sensitive [281]. Additionally, coordination polymers of BTO1, derived from Mn(II), Zn(II), Co(II), Ni(II), and Cu(II) cations, explode after mechanical stimuli and are classified as “less sensitive” (except for the Zn(II) complex, which is insensitive) [282].

Another studied dimeric tetrazole N-acid is bis(1-hydroxytetrazol-5-yl)-triazene (**H_3_BTO1N3**) and its salts with alkali metals and a Cu(II) complex. As with many other compounds discussed, highly hydrated alkali metal salts are insensitive [275]. The potassium salt of 1,1′-dihydroxy-5,5′-azobistetrazole (**H_2_BTO1DA**) is impact- and discharge-sensitive (20 J and 350 mJ, respectively), though substantially less so than the protonated form [283].

Zhang et al. synthesized and characterized MOFs containing the BTO1DZ anion. The Ni^2+^ and Cu^2+^ complexes are “less sensitive” to impact, while Co^2+^ and Cd^2+^ complexes are insensitive. These compounds are suitable catalysts for the thermal decomposition of other high-energy materials, such as ammonium perchlorate [284]. The sensitivity of MgBTO1DA (*IS* = 39.2 J) places it between insensitive and less sensitive compounds [285].

**Figure 16:**
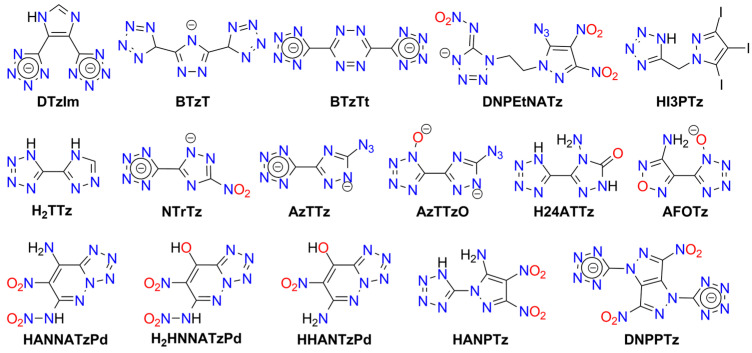
Mixed heterocycles containing a tetrazole unit used in ECC.

Another approach to designing high-energy compounds is the fusion of two different explosophores (Figure 16). The tetrazole ring has been bridged with 3,4-dinitro-1*H*-pyrazol-5-amine (**HANPTz**; Ag^+^, K^+^ and Na^+^ salts) [286] and 4-amino-1,2,4-triazol-3-on (**H_2_4ATTz**; Na^+^ salt) [287] and 3,6-dinitropyrazolo[4,3-c]-pyrazole (**H_2_DNPPTz**; K^+^ salt) [288]. A Cu(II) complex derived from H_2_4ATTz is insensitive [289]. The tetrazole ring has also been modified with dinitromethyl or trinitromethyl groups. Potassium salts of 2-dinitromethyl-5-trinitromethyl-5*H*-tetrazole (**H25PNTz**) and 2,5-bis(dinitromethyl)-5*H*-tetrazole (**H_2_25TNTz**) are highly sensitive [290]. The dipotassium salt of 5-dinitromethyl-5*H*-tetrazole (**H_2_5DNTz**) is less sensitive [291], while the monopotassium salt explodes more readily upon shock or heat [292]. Additionally, Na^+^ and Sr^2+^ salts of H_2_5DNTz have been prepared. NaH5DNTz and Na_2_5DNTz are highly sensitive to impact (<1 J), with the former also sensitive to friction and discharge. Salts of 5-trinitromethyl-2*H*-tetrazole (**H5TNTz**) are also sensitive explosive materials. Among those studied, the smallest stimuli induced detonation in the rubidium and cesium derivatives (*IS* = 0.5 J, *FS* < 1 N) [292]. Strontium salts require a stronger impact (7 J) to detonate [293].

An interesting example of a highly sensitive bridged system containing 5-nitroiminotetrazole and 5-azido-3,4-dinitropyrazole moieties (**HDNPEtNATz**) was synthesized by Kumar et al. [294]. High sensitivity to various stimuli has been reported for salts of 5-(5-azido-1*H*-1,2,4-triazol-3-yl)tetrazole (**H_2_AzTTz**) and 5-(5-azido-1H-1,2,4-triazol-3-yl)tetrazol-1-ol (**H_2_AzTTzO**). The highest sensitivity was observed for silver(I) and potassium derivatives, with the lowest for copper(I) derivatives [295,296]. Compounds containing linked tetrazole and 1,2,3-oxazole rings are discussed in the previous section.

Further, 3-(5-Tetrazoyl)-1,2,4-traizole (**H_2_TTz**) has been used as a ligand for the synthesis of MOF-type materials. Complexes with copper(II) and lead(II) have been mentioned as insensitive to friction and impact, but their calculated detonation properties have been reported [297,298,299]. The copper(I) derivative shows low sensitivity to impact (32 J) [299]. An iodinated pyrazole-tetrazole ligand (5-((3,4,5-triiodo-1*H*-pyrazol-1-yl)methyl)-1*H*-tetrazole; **HI3PTz**) was used for synthesizing a high-energy coordination polymer with zinc(II) ions, which detonates upon impact (80.3 J) [300].

Bis(1-(5-tetrazoylo)-5-tetrazoyl)amine (**H_3_BTzTzA**) forms salts with guanidinium and sodium cations. These MOF-like materials are almost insensitive in tests [301]. A MOF with mixed explosive and energetic ligands was prepared by Mei et al., who used 1-methyl-1,2,4-triazole (**1MeTr**) and the cyano(1*H*-tetrazo-1-yl)dihydroborate anion (**CNBTz**) to form a supramolecular polymer with Cu^2+^ cations. This complex is hypergolic with fuming nitric acid and insensitive to friction and impact [302].

Numerous tetrazole-derived lithium, potassium, cesium, strontium, and barium salts have been proposed as colorants for pyrotechnical mixtures [246,268,281,303,304,305].

The nitrotetrazole explosives and their explosive salts (including ammonium, hydrazine, hydroxylamine, guanidine, and organic cation salts) have been extensively reviewed by many authors [306,307,308,309,310,311]. Various aspects of copper tetrazolates and copper–tetrazole complexes have also been recently elaborated [312].

Interest in tetrazole-related explosives among non-professionals is high—these compounds’ simple synthetic routes, easily obtainable precursors, high sensitivity, and explosive energy make them widely discussed on discussion boards (for example, see a tetrazole and tetrazole-derivative monograph on sciencemadness.org: https://sciencemadness.org/scipics/tetrazoles(english).pdf, accessed on 3 November 2024) and extensively reported on social media. The extraordinary diversity of this group of explosives increases their appeal due to the “hunt and collect” effect for experience in preparing exotic, highly energetic compounds. The most commonly prepared compounds include salts and complexes of 5-nitrotetrazole, 5,5′-azobistetrazole, and 5,5′-azoxybistetrazole.

### 3.7. Nitrates

Nitrates (nitrates(V)) are salts of the strong nitric(V) acid. Both the acid and its salts are strong oxidizers. The enthalpy of formation (HOF) for gaseous HNO_3_ is [kJ mol^−1^] (NIST database). Nitrates are components of the oldest known explosive and high-energy material: black powder (gunpowder), which was invented in China in the 9th century, or possibly even earlier in the 2nd century [313,314]. Romocki suggested that mixtures similar to black powder were developed in China before the Common Era [315].

Simple metal nitrates are not explosive. However, their oxidizing properties make them essential substrates in pyrotechnic mixtures. On the other hand, strong explosives can be found among nitrates of metal complexes with ligands that exhibit reducing properties. Another approach to developing new high-energy materials involves preparing complexes of metal nitrates with energy-rich ligands (e.g., tetrazoles). Ammonium nitrate and nitrate salts of nitrogen-rich cations are explosive and are used as commercial explosives in various applications [15].

The primary conventional applications of metal nitrates in pyrotechnics and explosives production fall into three major categories: as an oxidizer in pyrotechnic compositions, as a nitration agent, or as a counterion in high-energy complexes.

#### 3.7.1. Oxidizers in Pyrotechnical Mixtures

Potassium, sodium, and calcium nitrates are commonly known as “saltpetres” (Indian (or ordinary), Chilean, and Norwegian saltpetre, respectively). They can be used as oxidizers in a wide range of high-energy mixtures.

The conventional gunpowder recipe includes potassium nitrate (75%), charcoal (15%), and sulfur (10%). Numerous variations of this formula have been published and used. Some variations adjust proportions, while others incorporate additional components, such as metal powders, manganese(IV) oxide, lead(II, IV) oxide, oil, rosin, naphthylamine, gum arabic, or even alternative fuels like graphite, sodium benzoate, cellulose, erythorbic acid, etc. [316]. Potassium nitrate may be wholly or partially substituted by other nitrates, such as ammonium nitrate (in “ammonium” or “amide” powders), lead(II) nitrate, or sodium nitrate (in mixtures known as “explosive saltpetre”) [15,316]. NaNO_3_ has some advantages over KNO_3_ (higher oxygen content), but its hygroscopic nature (due to impurities) limits its use. Other metal salt additives will be discussed in later sections.

Gunpowder has applications as a propellant for firearms (gunfire weapons), rocketry, pyrotechnics (display and consumer), and blasting agents. Although gunpowder’s industrial use is limited due to its replacement by other materials, it remains a primary choice and an introductory energetic material for non-professionals, including young chemists, weapon enthusiasts, rocket modelers, and others.

Potassium nitrate and sulfur (and, optionally, potassium carbonate) form a mixture known as “yellow powder”, which explodes violently upon heating [317].

Calcium nitrate is proposed as an oxidizer in various explosive blasting mixtures. It forms a hydrate and, therefore, is not typically used as the sole oxidizer in formulations. Instead, it serves as one of several oxidizing ingredients, often in combination with ammonium nitrate or sodium nitrate [318,319]. A similar material is calcium ammonium nitrate (CAN), a mixed salt used as fertilizer. Its composition and hydration degree vary. CAN can be used by HME producers to prepare pure ammonium nitrate or for improvised explosives in its original form [320,321].

Barium nitrate is a crucial oxidizer in various pyrotechnic devices, such as sparklers, fountains, Roman candles, flares, and fireworks [316,322,323].

Although most flash powders use chlorates or perchlorates as oxidizers, potassium, barium, or strontium nitrate (combined with metal powders and sulfur) can also be used [324,325,326,327].

Barium and strontium nitrates are colorants in pyro-effects, producing green and red flames, respectively. Other nitrate-based colorants include sodium nitrate (yellow flame) and calcium nitrate (orange flame) [322,323]. Nitrates of lithium, copper, indium, rubidium, and cesium are less commonly used due to their hygroscopic nature and, in the cases of indium, cesium, and rubidium, high costs [328,329,330,331].

Smoke-generating compositions also require oxidizers, with some based on KNO_3_ or NaNO_3_, though chlorates and perchlorates are more commonly used [316,332].

Metal nitrates are also used in various ignition devices. Mixtures of Mg, Zn, or Al powder with AgNO_3_, (NH_4_)_2_Ce(NO_3_)_6_, or Cu(NO_3_)_2_ ignite or even explode (with some delay) upon contact with water [333]. Ba(NO_3_)_2_, Pb(NO_3_)_2_, and KNO_3_ are used in friction-sensitive ignition mixtures and numerous compositions to transfer ignition from primers to propellants and deflagrating explosives [316].

#### 3.7.2. Nitration Agents

Due to restrictions on the availability of certain chemicals for non-professionals, many alternative methods for synthesizing well-known compounds have been proposed. HME makers also seek synthetic routes for basic chemicals that are now unavailable. Among such chemicals, nitric acid is essential for high-energy materials but can be challenging to obtain (e.g., in the EU). The availability of its salts, widely used as fertilizers, drug components, or curing agents, is much better [334]. Consequently, numerous methods for nitrating aromatics or preparing nitrate esters using KNO_3_/H_2_SO_4_ or NH_4_NO_3_/H_2_SO_4_ mixtures have been published in official papers and monographs, as well as unofficial materials [335,336,337,338].

In place of H_2_SO_4_ (which is also often restricted), other strong acids, such as polyphosphoric(V) acid, may be used [339]. Additionally, numerous methods for HNO_3_ production in academic or home laboratories using metal nitrates have been published, making common nitrates a potential starting material for nitric acid—an essential component in explosives production [340,341].

#### 3.7.3. High-Energetic Coordination Compound

**Figure 17:**
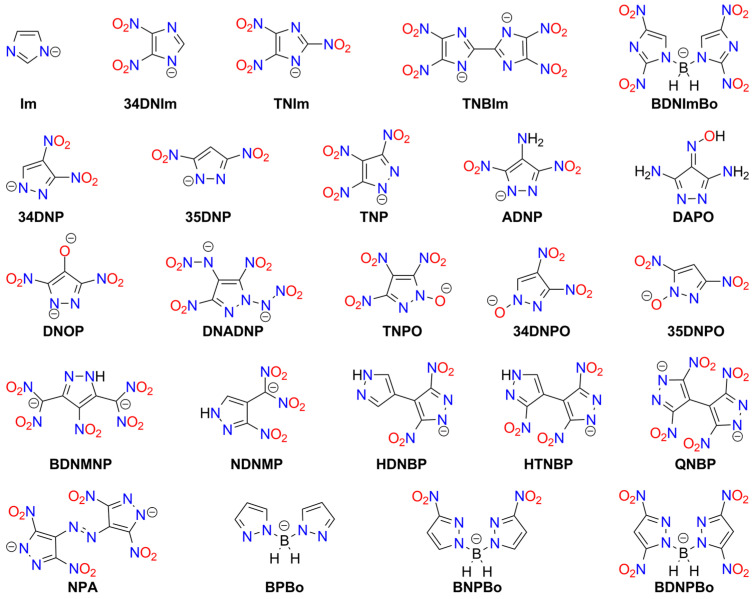
Structures of diazole (imidazole and pyrazole) ligands used in ECC.

**Figure 18:**
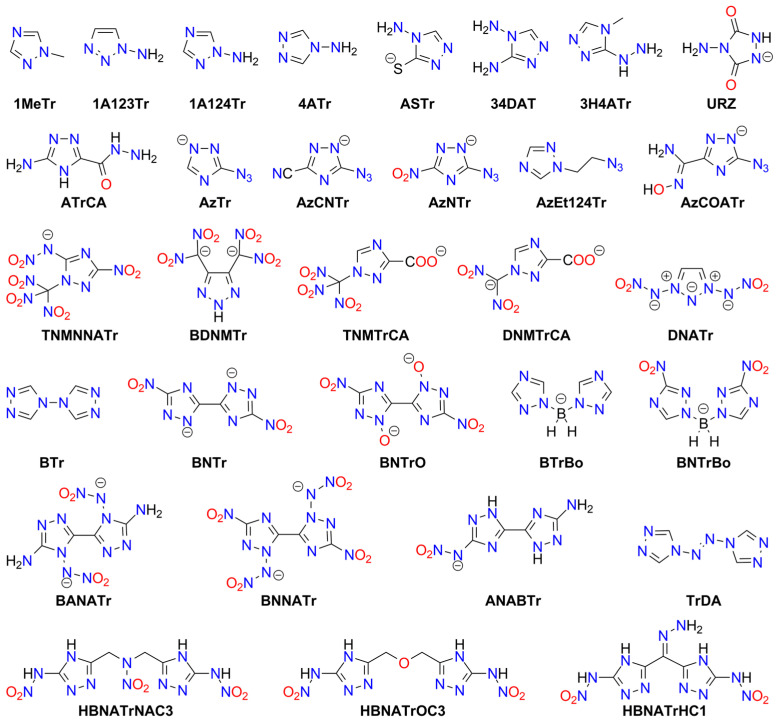
Structures of triazole ligands used in ECC.

**Figure 19:**
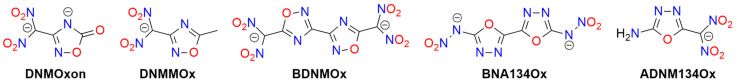
Structures of oxadiazole ligands used in ECC.

**Figure 20:**
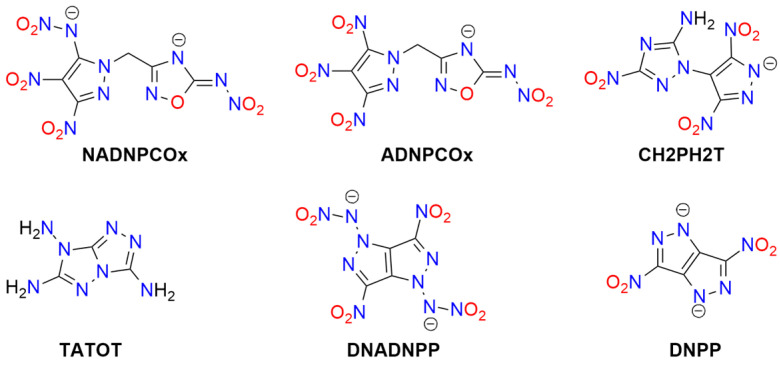
Structures of mixed and fused-ring azole ligands used in ECC.

**Figure 21:**
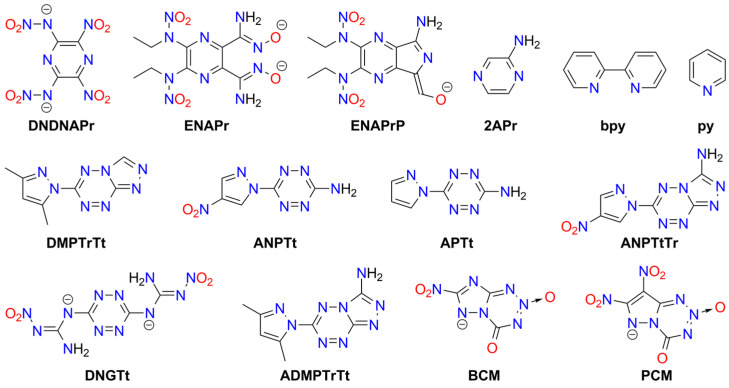
Structures of various azine ligands used in ECC.

Numerous organic energy-rich ligands were used in ECC, containing energetic anions (nitrates, chlorates, etc.). They are presented in Figure 17, Figure 18, Figure 19, Figure 20 and Figure 21. The first well-characterized examples of explosive metal nitrate complexes are salts containing poly(hydrazinato)metal cations: M(hz)_x_^n+^. Patil et al. synthesized and characterized the thermal and impact sensitivity of a series of these nitrates, with the Co(II) complex showing the highest impact sensitivity. The iron(II) derivative is unstable and ignites spontaneously during filtration at room temperature, while [Mg(hz)_2_](NO_3_)_2_ is insensitive [24].

Wojewódka and Bełzowski also studied these compounds, reporting sensitivity tests and proposing a series of mixed materials containing nitrates of hydrazine-metal complexes with other energy-rich materials (e.g., chlorates, azides, ferrocyanides) or mechanical sensitizers (e.g., glass). According to their study, the Ni(II) complex is significantly more sensitive than others [342]. Wojewódka et al. also compared nitrates of hydrazine complexes and perchlorates of hydrazine and ethylenediamine complexes with commercial explosives (RDX, PETN, TNT, HMX) in terms of maximum pressure, wave energy, and bubble energy [343].

Ni(II) and Co(II) complexes were also studied by Chhabra et al., who reported a higher sensitivity for the cobalt salt. Mixed materials containing perchlorates and ferrocyanides were also prepared [344]. Thermal stability and storage life data for these complexes were subsequently published [345]. The Ni complex was further studied by Shunguan et al., who reported on the influence of preparation conditions on its properties, noting its potential as a promising primary explosive [346]. Additional sensitivity data were presented by Talawar et al. [347,348]. Cartwright conducted detailed studies on the synthesis and stability of [Ni(hz)_3_](NO_3_)_2_, highlighting significant inconsistencies in reported sensitivity data [349]. The crystal structure of this complex was also determined [350].

Other explosive nitrates have been prepared from ammonia and ethylenediamine Co(III) complexes, which explode upon impact from a 2 kg weight dropped from heights of 60–180 cm (12–36 J) [351]. Other ammonia complexes were studied by Tomlinson et al., with the highest impact sensitivity (3.8 J) reported for [Cu(NH_3_)_4_](NO_3_)_2_ [352]. This compound was also used as a stimulant and sensitizer for ammonium nitrate [353].

Nitrates of silver–tetrazole complexes were studied by Sun et al. All obtained compounds are sensitive to impact (1–5 J), friction (5–60 N), and discharge (5–100 mJ), with the 5-nitrotetrazole derivative showing performance comparable to LA [354]. Silver nitrate–aminotetrazole systems (**H5ATz**, **1M5ATz**, **2M5ATz**) were studied by Klapötke et al., who reported the highest sensitivity for the salt containing two silver cations per ligand molecule [355]. Silver nitrate was also converted to complexes with isomeric ditetrazoylmethanes (**11DTM**, **12DTM**, **22DTM**), all of which are highly impact-sensitive and reactive to friction and discharge. The 2,2′-isomer also forms a complex with copper(II) nitrate [67].

Bis(5-tetrazoyl)methane (**H_2_55DTM**) has been used as a ligand for metal nitrate salts, with the Ni(II) derivative being the most sensitive. Analogous chloride complexes are almost insensitive [356]. Another tetrazole-containing nitrate was prepared by Wurzenberger et al., who reported that the Cu(II) complex of 1-(2-azidoethyl)-1H-tetrazole (**1AzETz**) is impact-sensitive. However, its perchlorate and chlorate analogs are easier to stimulate. An anhydrous form of the nitrate complex was obtained but not characterized [357]. Similar results were obtained for complexes with a 3-azidopropyl (1AzPTz) analog [70]. Results for 1AzETz were also compared with its 1-(2-azidoethyl)-1H-tetrazole (2AzETz) isomer, with the 2-substituted analogs generally showing greater sensitivity [358]. A copper(II) nitrate complex with an azidomethyl analog was also reported [20].

Other copper(II) complexes have been synthesized using 5-(5-nitrotetrazole-2-ylmethyl)-tetrazole (**H5NTzMTz**) [271] and 1,2-bis(1*H*-tetrazol-5-yl)ethane (**H_2_55DTE**) [359]. The compound 5-(1-Methylhydrazinyl)-1*H*-tetrazole (**H5MHTz**) forms copper(II) nitrate complexes sensitive to impact, friction, discharge, thermal, and laser stimuli. However, the perchlorate analogs of these complexes are easier to stimulate [243].

Further, 1-methyltetrazole (**1MTz**) was used to synthesize a series of complexes, including nitrates of Co(II), Ni(II), Zn(II), Cu(II), and Ag(I). All are insensitive to mechanical stimuli, except the silver salt, which explodes upon impact (40 J) and shows the highest discharge sensitivity (160 mJ) among the nitrates studied [71]. Among copper(II) nitrate complexes with 1-cycloalkyl-substituted tetrazole, only the 1-cyclobutyltetrazole (**1cBTz**) derivative is sensitive (*IS* = 9) [19].

Other tetrazoles studied in metal nitrate systems include 2,2-bis(1*H*-tetrazol-5-yl)propane (**11BTC3**; Cu, Co, Ni, Zn salts) [360], 1-amino- and 2-aminotetrazole (**1ATz** and **2ATz**; Zn, Cu salts) [361], and 1-tetrazoleacetonitrile (**1TzAN**; Cu salt) [73]. All these complexes are explosive.

Wojewódka and Bełzowski prepared 5-(2,4,6-trinitrophenylamino)-1*H*-tetrazole (**HPicATz**) and its complexes with transition metal nitrates and perchlorates, characterizing their mechanical sensitivity. Among the nitrates, Ni(II) and Zn(II) complexes show the highest impact sensitivity, while Cu(II) has the highest friction sensitivity [362].

Zinc nitrate forms an explosive (but nearly insensitive) complex with 3,6,7-triamino-7*H*-[1,2,4]triazolo[4,3-b][1,2,4]triazole (**TATOT**); its chloride complex is also insensitive [363]. Another triazole ligand, 3,4-diamino-1,2,4-triazole (**DATr**), forms complexes with nitrates of divalent cations Mn, Co, Ni, and Zn, with only Co and Ni salts being impact-sensitive and Co being friction-sensitive [364].

Detonating but low-sensitivity MOFs containing Ag(I) and Cu(II) with nitrate(V) ions were synthesized by Li et al. using bis(1,2,4-triazo-4-yl)diazine (**TrDA**) as a ligand [365]. The Cu(II) complex was further reacted with iodate ions, resulting in a series of materials differing in the NO_3_^−^/IO_3_^−^ ratio [366]. Studies on the application of [Cu(TrDA)_3_](NO_3_)_2_ as a combustion catalyst have also been reported [367].

High impact sensitivity has been reported for transition metal nitrate complexes with 3-amino-1-nitroguanidine (**ANQ**), with the highest sensitivity (IS < 1 J) observed in Co(II) and Ag(I) salts [368]. Szimhardt and Stierstorfer synthesized nitrates of methylsemicarbazide (**MSC**) transition metal complexes, comparing them with other salts (perchlorates, chlorides, sulfates(VI), azides, picrates, and styphnates), finding nitrate salts to be insensitive (for other salts, see appropriate paragraphs). Salts with non-energetic anions (Cl^−^, SO_4_^2−^) are also insensitive [75].

Zhang et al. reported the explosive properties of biuret (**BIU**) complexes with transition metal nitrates and perchlorates, classifying copper(II) and silver(I) nitrates as less sensitive and other nitrates as insensitive [369].

Other ligands studied in high-energy complexes include tetrazine derivatives (**APTz**, **ADMPTz**, **DNAzPTz**, **DNAzDMPTz**; Cu^2+^, laser-induced materials) [370], 4,5-bis(5-tetrazolyl)imidazole (**H_2_DTzIm**; Cu^2+^ and Pb^2+^, insensitive) [371,372], 3,5-diaminopyrazolone-4-oxime (**DAPO**; Cd^2+^, Co^2+^, insensitive) [373], and 3,4-diaminofurazan (**DAF**; Ag^+^, Cu^2+^, Zn^2+^) [374,375].

A complex of 1,4-diazabicyclo[2.2.2]octane (**DABCO**) nitrate with potassium nitrate has a perovskite-like structure, with energetic and combustion properties superior to black powder, making it suitable as a propellant and a promising energetic material for pyrotechnics [376].

Trihydrazinenickel(II) and trihydrazinecobalt(III) nitrates are popular, easily obtainable primary explosives made from non-restricted compounds. DIY methods for phlegmatizing the nickel complex have also been published online. Another compound, bis(ethylenediamine)copper(II) nitrate, is a simple salt accessible with reagents available in school laboratories.

### 3.8. Chlorates and Perchlorates

Chlorates (chlorates(V)) and perchlorates (chlorates(VII)) are salts of strong acids—chloric(V) acid (HClO_3_) and chloric(VII) acid (HClO_4_). Both the acids and their salts are strong oxidizers, with enthalpies of formation HOF(HClO_3_(g)) = −4.9 [kJ mol^−1^], HOF(HClO_4(g)_) = 1.8 [kJ mol^−1^] (ATcT database). Currently, chlorates and perchlorates have a broader range of applications in pyrotechnic compositions than nitrates. Mixtures based on these salts are more exergic and burn or explode more rapidly. In general, chlorate-based mixtures are more sensitive than those based on perchlorates. Thus, many commercial pyrotechnic devices and propellants use the latter as oxidizers. For example, mixtures of KClO_3_ with red phosphorus or sulfur (with the addition of Cu^2+^ salts) may explode spontaneously [377].

Chlorates and perchlorates are applied in various products, including modified gunpowder, rocket propellants, gun propellants, pyrotechnic propellants, bullet tracers, match-head compositions, smoke-generating mixtures, igniters and primers, ignition-transferring mixtures, friction-sensitive mixtures, ignition-delay compositions, flares, flash powders, fireworks, firecrackers, percussion caps, roman candles, and more. Perchlorates and chlorates of Li, Ba, Sr, and Cu are also widely used as flame colorants [316,323,378,379].

In many countries (e.g., the EU), the availability of chlorates and perchlorates is restricted, and non-professionals are prohibited from possessing these substances to reduce the risk of illegal and reckless use. However, chlorates are easily produced through the electrolysis of corresponding chlorides or from decomposed bleach (which contains hypochlorite); as a result, they are sometimes illegally made by self-taught pyrotechnicians [340].

The higher energetic properties of chlorates and perchlorates make them a favorable counterion in high-energy metal complexes. Many nitrate derivatives discussed in an earlier chapter were compared with analogous chlorate and perchlorate salts, with the latter generally showing greater sensitivity and more robust detonation characteristics.

Simple chlorates and perchlorates of ammonia or aliphatic amine complexes with Cu(II) were reported as insensitive (undergoing deflagration or detonation) by Amiel [380]. Joyner documented the explosive properties of chlorates and perchlorates of ammonia and ethylenediamine Co(III) complexes [351], with further data on this class of compounds provided by Tomlinson et al. [352].

Among the perchlorates of hydrazine–metal systems, the most intensively studied are Ni(II) and Co(II) salts. [Ni(hz)_3_](ClO_4_)_2_ was reported as a potent explosive, even when wet [381]. According to Wojewódka and Bełzowski, this salt is the most sensitive among those studied. High impact sensitivity was confirmed for the Cr(III) complex, while the Cd(II) salt is friction- and spark-sensitive [342]. [Mg(hz)_2_](ClO_4_)_2_ is reported to be insensitive [24].

Bushuyev et al. reported the structures and properties of Zn(II) and Co(II) perchlorate complexes, formed with hydrazine in the presence of CO_2_ and containing mixed hydrazine and carboxyhydrazine (**Hhzc**) ligands in the coordination sphere [382], and compared them with compounds that contain only hydrazine ligands [350]. A comprehensive review of chlorate-based HMEs was published by Horocke et al. [383].

Various perchlorate and chlorate tetrazole–metal complexes have been studied. Further, 1-Ethyl- and 1-(2-azidoethyl)-1*H*-tetrazole (**1ETz**, **1AzETz**) complexes with copper(II) were studied by the Klapötke group. In general, chlorates show lower decomposition temperatures and higher friction and discharge sensitivity, though no clear trend was observed in impact tests; the perchlorate complex [Cu(AzEtTz)_6_](ClO_4_)_2_ is the most sensitive among the salts studied. Silver(I), iron(II), and zinc complexes were also reported in the same study, with Ag(AzEtTz)ClO_4_ being more sensitive than LA [357]. This study continued with the preparation of complexes of 1-propyl- (**1PTz**) and 1-(3-azidopropyl)-1*H*-tetrazole (**1AzPTz**) with chlorates and perchlorates of Cu(II), Mn(II), Zn(II), and Fe(II). These salts are generally less sensitive than ethyl analogs. The study confirmed higher sensitivity of chlorates to friction, discharge, and heat [70]. Another study focused on 1-azidomethyl (**1AzMTz**) derivatives, which form complexes showing deflagration-to-detonation transitions, making them suitable as primary explosives. The studied chlorate and perchlorate salts are much more sensitive than their ethylene or propylene-linked analogs [20].

Other ligands studied include 5-aminotetrazoles and their monomethyl derivatives. Compared to nitrates, these compounds exhibit significantly higher sensitivity and can be classified as highly sensitive materials [355]. The same trend was observed for photosensitive Cu(II) complexes of 5-(1-methylhydrazino)tetrazole [243]. Bis(1*H*-tetrazo-5-yl)methane (**H_2_55DTM**) was studied only as a ligand for Cu(II) perchlorate, forming extremely sensitive complexes [356]. Further studies on complexes with isomeric ligands resulted in perchlorate complexes with silver(I) and copper(II) [67].

Other bridged isomeric tetrazoles studied contain 1,3-propylene linkers (**11DTP**, **12DTP**, **22DTP**), with complexes formed using Mn(II), Fe(II), Co(II), Ni(II), Cu(II), Zn(II), and Ag(I). The cobalt salt displayed the highest sensitivity [88]. The mixed perchlorate/5-nitrotetrazolate silver salt is extremely sensitive, with a sensitivity higher than that of nitrate complexes and other 5-substituted tetrazole derivatives [354]. Other mixed salts were prepared using 5-nitrotetrazolate and perchlorate anions with amminacomplexes of Cu(II), Zn(II), Co(III), and Ni(III) [384,385]. The cobalt complex was later studied as an initiator for conventional explosives like PETN or tetryl and compared with an ASA (azide/styphnate/aluminium) detonator [348].

Several nitromethyl derivatives of tetrazole were also studied. First, 1-(nitromethyl)-5*H*-tetrazole (**1NMTz**) was used as a ligand for chlorates, perchlorates, and nitrates of Co, Ni, and Fe, with the highest sensitivity (*IS* < 1 J, *FS* = 14 N) reported for the Fe(ClO_4_)_2_ complex of 1NMTz [386]. Among 1-(nitratomethyl)-5*H*-tetrazole (**1NOMTz**) ECCs, the copper(II) chlorate complex is the most sensitive to mechanical stimuli, exploding upon impact below 1 J and friction of 0.4 N. Complexes of the same ligand with copper polynitrophenolates show greater friction resistance (*FS* > 40 N) [387].

Evers et al. reported explosive copper(II) perchlorate adducts with a tetrazole dimer bridged by -CH_2_CH_2_- linker (**H_2_55DTE**) [359]. Numerous perchlorates derived from 1-methyltetrazole were obtained by Stierstorfer et al., with the iron complex being the most impact-sensitive and the Cu(II) complex being the most friction-sensitive among the salts studied [71]. The studies continued with salts of 1-amino and 2-amino-5*H*-tetrazoles (**1ATz**, **2ATz**), with many of the obtained compounds showing high sensitivity (*IS* < 1, *FS* < 0.1) [361].

Also, 5-amino-2-methyl-2*H*-tetrazole (**2M5ATz**) forms four distinct complexes with copper(II) perchlorate, three of which were characterized as explosive. This study also reported a sensitive 2-methyl-2H-tetrazole (**2MTz**) complex [388]. An isomeric compound, 1-amino-5-methyl-1*H*-tetrazole (**1A5MTz**) was examined as a ligand for Mn, Fe, Cu, and Zn perchlorates, with all salts showing high sensitivity, some exceeding that of LA [389].

Tang et al. synthesized a 1-methyl-5-aminotetrazole (**1M5ATz**) complex with Cu(ClO_4_)_2_, along with analogous picrate and 3,5-dinitrobenzoate salts. Only the perchlorate could be classified as very sensitive based on impact sensitivity, though it is friction-insensitive [390]. Among ditetrazoles with a 2,2-propylene linker (**H_2_55DT11P**), the most sensitive are iron(II) and zinc adducts [360].

Braun et al. characterized a series of 1-cycloalkyl-substituted tetrazoles (**1cPTz**, **1cBTz**, **1cPeTz**). Unlike their nitrate counterparts, chlorates and perchlorates of these complex cations are sensitive [19]. Also, 1,5-dimethyltetrazole (**15DMTz**) was complexed with several transition metals and isolated as perchlorates and polynitrophenolates, with Mn(II), Cu(II), and Zn(II) salts exhibiting sensitivity to impact and friction [69].

Perchlorates of complex cations containing a picrylamino-derivative of tetrazole (**HPicATz**) were studied and compared with analogous nitrates. Generally, they display similar mechanical sensitivities, though the most impact-sensitive was the Zn(II) complex, while the Hg(II) complex showed the highest friction sensitivity [362].

A systematic study on copper(II) chlorates containing triazole and tetrazole ligands was published by Wurzenberger et al. The compounds obtained were comprehensively studied; data on their sensitivity, behavior upon laser radiation, initiation properties against PETN, toxicity, and physicochemical properties were reported. Two complexes, those of 4-amino-1,2,4-triazole (**4ATr**) and 1,3-bis(2*H*-tetrazol-2-yl)propane, were described as superior to LA in terms of explosive properties [391]. The 4ATr copper(II) perchlorate complex was studied in detail by Cudziło et al. for its primary explosive properties [392,393].

Comprehensive research on aminotriazole complexes containing 1-amino-1,2,3-triazole (**1A123Tr**), and 1-amino-1,2,4-triazole (**1A124Tr**), with Cu, Zn, Mn, and Fe salts (perchlorates, chlorates, nitrates, chlorides, picrates, styphnates) was reported by Szimhardt et al. Some of the studied complexes exhibit extreme sensitivity to impact (<1 J), friction (2 N), and discharge (10 mJ) [68]. Wang et al. proposed a silver perchlorate complex with 3-amino-1*H*-1,2,4-triazole-5-carbohydrazide (**ATrCA**) as a laser-pulse-initiating primary explosive [394].

A series of Co(III) perchlorates containing ammonia and tetrazole-derived ligands were studied by Zhilin et al. Among the compounds prepared, the highest shock sensitivity (64% detonation after a 12.8 J impact) was noted for [Co(NH_3_)_4_(Tz)_2_]ClO_4_, while [Co(NH_3_)_4_(5NTz)_2_]ClO_4_ showed the best priming properties [395]. Some complexes containing chlorate and perchlorate anions were reported as explosive without additional data, e.g., [Co(TzAH)_2_](ClO_4_)_2_ [396] or chlorates/bromates/nitrates of 4-amino-1,2,4-triazole (4ATr) complexes with Zn, Cd, Cu, and Ni [397]. A Werner complex with a 5-cyanotetrazolate anion as a ligand was reported as a material showing a deflagration-to-detonation transition [398]. Other examples were reported by Smirnov et al. and proposed as primary explosives [399].

Other azole ligands studied in energetic complexes with chlorate and perchlorate counter-ions include 1,3-bis-1,1′-tetrazolylnitrazapropane (**11TNP**) [400], 1- and 2-tetrazoleacetonitriles (**1TzAN**, **2TzAN**), and 2-tetrazoleacetamide (**2TzAA**) [73].

Analogous to nitrates, perchlorates of transition metal/3-amino-1-nitroguanidine (**ANQ**) complexes were studied. These are more sensitive than their nitrate and chloride counterparts, with Cu(II) and Ag(I) derivatives being the most impact-sensitive (*IS* < 1), while Ni(II) and Co(II) showed the highest friction sensitivity [368]. Semicarbazide ligands were also used in metal-perchlorate materials. Szimhardt and Stierstorfer reported methylsemicarbazide (**MSC**) derivatives, classifying the copper(II) salt as very sensitive, Zn, Mn, and Ni as less sensitive, and an additional Zn salt as insensitive [75]. Carbohydrazide (**CHZ**) complexes were studied by Talawar et al., with Ni(II) perchlorate being very sensitive, while the other salts were classified as less sensitive [401]. Joas and Klapötke also studied these complexes, with differing results from Talawar’s group, as all complexes showed high impact sensitivity [402].

Biuret (**BIU**) complexes of metal perchlorates are explosive. However, their sensitivity is relatively low. The highest IS values were reported for Cu(II) and Ag(I) complexes (25 and 30 J, respectively) [369].

Other studied ligands for (per)chlorate salts include **TATOT** (Zn^2+^) [363], **H_2_4ATTz** (Co^3+^) [289], 1-fluoromethyl- (**1FMTz**) and 1-fluoromethyl-5-amino-1*H*-tetrazoles (**1FM5ATz**) (Cu^2+^ and Cu^+^; the later decomposes rapidly at room temperature and was not characterized) [403], **4ATr**, **1M5Atz**, and **15DATz** (Co(NH_3_)_5_^3+^) [404], **15DATz** (Cd^2+^) [405], and tetrazine ligands: **APTt**, **DMPTrTt**, **ADMPTrTt**, **ANPTt**, and **ANPTrTt** (Fe^2+^; laser-ignitable compounds) [406,407]. The 3-hydrazino-4-amino-1,2,4-triazole (**3H4ATr**) ligand forms complexes with Cu^2+^, Co^2+^, Ni^2+^, and Cd^2+^, which are described as photosensitive explosives, though other sensitivity data were not reported [408].

**Figure 22:**
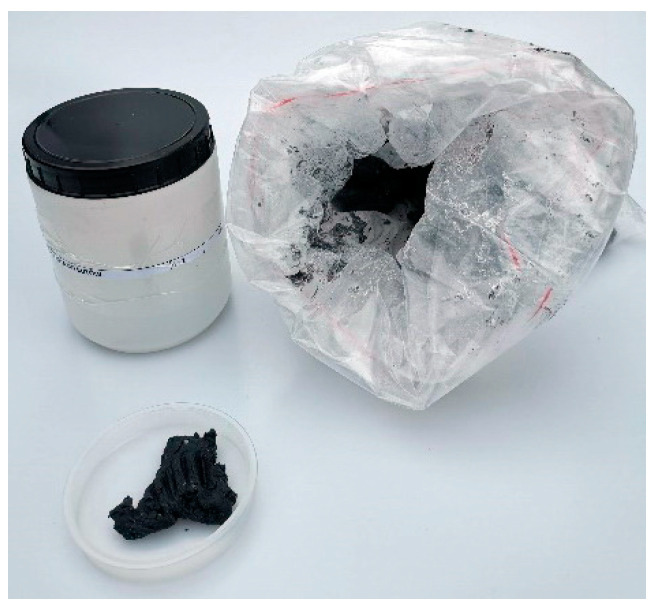
HME based on potassium chlorate and black shoe polish.

The most significant use of inorganic chlorates and perchlorates in HME is in fuel-oxidizer systems (Figure 22): flash powders (e.g., used in the 2002 Bali and 2004 Jakarta bombings [409]), so-called Poor Man’s C-4 (vaseline/KClO_3_; an incident in Merced in 2015), Armstrong’s mixture (red phosphorus/KClO_3_; accident in Kingston, 2015), and Rack-a-Rock (nitrobenzene/KClO_3_). Illegal production of some chlorate and perchlorate-based primers is also a serious challenge. The most popular are tetraamminecopper(II) perchlorate [410,411] and bis-(5-nitro-2*H*-tetrazolato-*N*^2^)tetraminecobalt(III) perchlorate. Several poorly characterized explosives, such as chlorates of copper(II) glycinate or bis(ethylenediamine)copper(II), are also reported on discussion boards.

### 3.9. Other Compounds

Data on numerous other high-energetic metal-containing compounds have been published. However, they were not studied as comprehensively as the classes listed above.

Silver and mercury oxalate (Ox) explode upon impact, friction, and heating. Their exact parameters for mechanical stimuli have not been measured [7]. Silver salt is a popular chemical for classroom and lecture demonstrations [412].

The first report on the thermal instability of copper(II) amine (or ammonia) complexes with bromates (as well as chlorates and perchlorates), which results in deflagration or explosion, was published in 1935 [380]. Bromates, iodates, nitrites (nitrates(III)), and permanganates (manganates(VII)) of Co(III) complexes with ethylenediamine and ammonia were studied by Joyner [351]. Copper(II) bromate complexes with ethylenediamine (**en**) and various 1,3,4-triazole (**4ATr**) and tetrazole ligands (**1MTz**, **2M5ATz**, **22DTP**, **12DTP**, and **11BTziP**) were prepared by Wurzenberger et al. All (except the 22DTP complex) are more impact-sensitive than LA or PbSt but exhibit lower friction and discharge sensitivity than these conventional primers. They are also laser-detonable [413].

The MnO_4_^−^ anion is a strong oxidizer, related to the strong permanganic acid (manganic(VII) acid; HMnO_4_). Permanganates, especially potassium permanganate, are important components in non-professional pyrotechnic mixtures. When mixed with powdered metals (Al, Mg, Zn, Sb, etc.), they form flash powder [414,415,416]. Some potassium permanganate compositions are used as pyrotechnic delay mixtures [417,418] or fuels [419]. KMnO_4_ can cause spontaneous, delayed ignition when mixed with glycerin, ethylene glycol, or other polyols; the delay time depends on the size of the oxidizer particles. This reaction is well-known as a spectacular chemical demonstration [420], but it is also used to initiate controlled fires in woodlands [421]. However, these mixtures may be illegally used for intentional arson. Mixtures of potassium permanganate with concentrated sulfuric acid release manganese(VII) oxide—a powerful oxidizer that explodes upon contact with various organic materials (paper, organic solvents, etc.) and can cause fires [422]. Ammonium permanganate is a moderate explosive; in its dry form, it may detonate upon mechanical stimulation, ultrasonic induction, or heating over 140 °C [7,423,424]. Other ammonium permanganates are also potentially explosive [425]. Potassium permanganate is widely used by pyrotechnics enthusiasts, particularly in flash powders and self-igniting mixtures, and is described on the internet. Its availability as a pharmaceutical ingredient makes it accessible to potential terrorists (e.g., bomb traps used in Aurora, Colorado). It is listed as a precursor in documents published by various security agencies, including AOAV [426], GICHD [427], and Interpol [49], as well as by the Committee on Reducing the Threat of Improvised Explosive Device Attacks by Restricting Access to Chemical Explosive Precursors [428].

A less-known salt that forms highly explosive mixtures is sodium hypophosphite (NaH_2_PO_2_). Its mixtures with chlorates (e.g., sodium chlorate(V)) are as powerful as glycerol trinitrate (nitroglycerine), exploding upon heating [429]. Sodium hypophosphite–sodium nitrate systems can also detonate when heated [430]. These compounds are rarely used by hobbyists for explosions, though attempts are well-documented on YouTube and discussion boards.

Potassium (or sodium) persulfate(VI) (K_2_S_2_O_8_ & Na_2_S_2_O_8_), salts containing a peroxo moiety (-O-O-), may be used in exotic flash powder compositions with magnesium or aluminum. This formulation is well-known in amateur circles, although it is not extensively studied or described in the scientific literature. Only a few patents mention persulfates as oxidizers in pyrotechnics [431]. Even more surprising is the lack of scientific information about persulfates in explosive coordination compounds, such as [Cu(NH_3_)_4_](S_2_O_8_), which is well-known among explosion enthusiasts (www.sciencemadness.org/smwiki/index.php/Tetraaminecopper(II)_persulphate, accessed on 3 November 2024). It is moderately sensitive to shock but explodes quickly upon heating.

Nitrocarbamoyl azide (**HNCAz**) is an N-acid; the acid and its salts are extremely sensitive to friction, impact, and discharge [432].

A few simple nitroamide anions have been studied as explosophores. The dinitroamide anion (**ADN**) forms energetic salts with various metals and complex cations. KADN and CsADN were studied as oxidizers in pyrotechnic mixtures containing nitrocellulose and titanium powder. These salts were classified as insensitive to mechanical forces (*IS* > 20 J, *FS* > 360 N) but spark-stimulable (*ESD* > 5.6 mJ). The mixtures studied showed increased sensitivity and combustion velocity [433]. Fischer et al. obtained and characterized adducts of ADN salts complexed with 3-amino-1-nitroguanidine ligand (**AQN**) [368]. Additionally, an ADN copper salt containing a bis-tetrazole ligand, **11DTP**, was synthesized and compared with perchlorates, styphnates, and cyjanodinitromethylenides (**CDNM**) [88]. These studies were continued, resulting in the preparation of numerous ADN salts of various triazole and tetrazole ligands [434].

Further, 1,1,2,2-ethyenedinitramide (**H_2_EDN**) was converted into numerous metal and nitrogen-rich cation salts. The metal cations studied included Ag(I), Cu(II), alkali metals, and alkaline earth metals. Additionally, several silver and copper-derived MOFs containing the EDN anion and various ligands were synthesized. Some of the materials obtained, including Na, Rb, Ag, and Cu-MOFs, are highly sensitive [435].

Tetranitroaminoethane (**H_4_TNAE**) was synthesized and studied by Born et al. Its salts are very impact-sensitive but less or insensitive to friction. The authors also discuss and cast doubt on explosion parameters published in conference proceedings, suggesting they are overestimated [436].

Urazine (**HURZ**) salts of alkali metal ions (Li^+^, Na^+^, K^+^) were found to be insensitive (*IS* > 40 J, *FS* > 360 N), in contrast to their perchlorate salt and perchlorate-containing Cu(II) complex [437].

Another energy-rich simple anion is dicyanoamide (**DCA**). Its copper(II) salt was converted into tetrazole complexes. The copper(II) dicyanoamidate is insensitive to mechanical stimuli but may explode after ESD. Complexes with nitrogen-rich ligands are sensitive [438]. Nitrocyanamide (**NCA**) metal salts were also comprehensively studied. Silver nitrocyanamide and some copper(II) NCA salts, containing tetrazole-derived ligands, show high sensitivity (*IS* < 5 J) [439].

Further, 2,2-diazidomalonic acid (**H_2_DAMA**) was used for the preparation of Na^+^ and K^+^ salts. The former forms an insensitive hydrate, while K_2_DAMA is highly sensitive (*IS* = 5) [440].

Nitroimidazole derivatives are known as energetic compounds. Further, 3,4-dinitroimidazolates (**34DNIm**) have been studied as potential dopants for pyrotechnic mixtures to enhance flame coloration. Their sensitivity is moderate to low. However, the calcium salt shows surprisingly high impact sensitivity (3 J), with the next in line, the sodium salt, showing an *IS* = 25 J [441]. On the other hand, the potassium salt of 4,4,5,5-tetranitro-2,2-bisimidazole (**H_2_TNBIm**) was found to be insensitive (*IS* > 40 J, FS > 240 N, ESD > 1 J), unlike its guanidine adducts [442]. Copper(II) complexes with TNBIm were obtained by Lewczuk et al., who reported that these complexes are sensitive to impact but insensitive to friction [443].

Drukenmüller et al. studied other 3,5-dinitro- and 3,4,5-trinitropyrazoles (**H35DNP**, **HTNP**), 2,4,5-trinitroimidazole (**HTNIm**), and their salts with s-block elements and Cu(II). No clear correlation was observed between the number of nitro groups and sensitivity. The highest *IS* values were reported for hydrated Ca35DNP (8 J) and BaTNP (5 J) [444].

Li et al. reported heat-resistant explosives based on a nitrated pyrazole-triazole dimer, 4-(5-amino-3-nitro-1*H*-1,2,4-triazol-1-yl)-3,5-dinitropyrazole (**HCPT**). Its potassium salt is impact-sensitive (*IS* = 7.5 J) but stable up to 323 °C [445]. Lei et al. synthesized a pyrazole containing two energetic moieties: nitromethyl and dinitromethyl (3-nitro-4-(dinitrometylo)pyrazole; **HNDNMP**). Their salts are explosive, with the potassium one exhibiting primary explosive properties [446]. Another example of an explosive pyrazole salt is potassium (*E*)-1,2-bis(3,5-dinitro-1H-pyrazol-4-yl)diazene (**H_2_NPA**). While the acid is insensitive (*IS* >40 J, *FS* = 240 N), K_2_NPA explodes upon weak stimuli (1.5 J, 60 N) [447]. Two isomeric nitropyrazoles, 3,4-dinitro- (**H34DNP**) and 3,5-dinitropyrazole (**H35DNP**), and their salts were prepared. Hydrated forms, such as Na34DNP‧2H_2_O, K34DNP‧2H_2_O, and Na35DNP‧2H_2_O, are insensitive (*IS* = 40 J, *FS* = 360 N, *ESD* = 1500 mJ), while anhydrous crystals of K35DNP explode upon impact (8.5 J), friction (240 N), and discharge (400 mJ) [448]. Hydrated and anhydrous silver(I) 34DNP polymers were sensitive to friction and impact [449].

Additionally, salts of dinitropyrazole N-oxide show sensitivity. Bölter et al. synthesized and studied these compounds. Among derivatives characterized, the potassium salts of 3,4- and 3,5-dinitropyrazol-1-oxides (**H34DNPO**, **H35DNPO**) show the highest tendency for initiation [450].

Further, 3,5-dinitro-4,4′-bipyrazole (**HDNBP**) and 3,3′,5-trinitro-4,4′-bipyrazole (**HTNBP**) were used to prepare potassium and cesium salts. Hydrates of dinitroderivatives are classified as less sensitive (*IS* = 30), while KTNBP is classified as sensitive (*IS* = 7); the sensitivity of CsTNBP was not determined [451]. For 3,3′,5,5′-tetranitro-4,4′-bipyrazole (**QNBP**), only dipotassium salts were characterized. It is more sensitive than the trinitro analogue (*IS* = 1.5) [452].

A fused-ring dimeric pyrazole, 3,6-dinitropyrazolo[4,3-c]pyrazole (**H_2_DNPP**), was studied by Zhang et al. They obtained its salts with monovalent cations (K^+^, Na^+^, Ag^+^). Potassium salt, the most sensitive, shows high thermal stability (decomposition at 395 °C) [453]. The potassium salt of 1,4-dinitroamino-3,6-dinitropyrazolo[4,3-c]pyrazole (**H_2_DNADNPP**) also exhibited high sensitivity [454].

Other pyrazoles studied include 2,4-dinitroamino-3,5-dinitropyrazole (**H_2_DNADNP**) and 1-hydroxy-3,4,5-trinitropyrazole (**HTNPO**). The potassium salts of both compounds are mechanically sensitive [455]. Singh et al. described a method for bonding five nitro groups to the pyrazole ring, yielding 3,5-bis(dinitromethyl)-4-nitropyrazole (**H_2_BDNMNP**), which was characterized in the form of potassium, ammonium, and hydrazinium salts. K_2_BDNMNP is easily detonable [456].

Salts (Na, K) of 3,5-dinitro-4-hydroxypyrazole (**H_2_DNOP**) and 4-amino-3,5-dinitropyrazole (**H_2_ADNP**) were also tested as potential explosives. Their sensitivity is moderate, but detonation properties are superior to TNT [457].

Also, 1,2,3-triazoles are known explosophores and are often modified with other high-energy groups. The double nitroamine-substituted 1,2,3-triazole, 1,3-bis(nitroamino)-1,2,3-triazole (**HDNATr**), is an N-acid, and its potassium and silver salts are both highly sensitive [458]. Additionally, the potassium salt of 4,5-bis(dinitromethyl)-1,2,3-triazole (**H_2_BDNMTr**) is highly endo-energetic and explosive, reacting to stimuli at less than 1 J impact [459]. Another triazole, 5,5′-bis(3-amino-4-nitroamino-1,2,4-triazole) (**H_2_BANATr**), has been proposed in the form of its Li^+^ salt as a “green” red pyrotechnic colorant [460]. K_2_BANATr shows the highest sensitivity among salts of this trizole [461]. This acid’s salts were studied more comprehensively, and additional research provides the properties of alkali and other metal salts. The anhydrous salts are impact-sensitive, while the hydrates (Na^+^, divalent cations) are classified as insensitive. All were tested as flame colorants [462].

Another colorant, cesium salt of *N*-(5′-amino-1*H*,1′*H*-[3,3′-bi(1,2,4-triazol)]-5-yl)nitramine (**HANABTr**), was proposed by Wang et al. Due to its friction sensitivity (360 N), it is classified as less sensitive [463]. The potassium salt of 5,5′-bis(3-nitro-1-nitroamino-1,2,4-triazole) (**H_2_BNNATr**) is more sensitive than K_2_BANATr [464]. Izsák et al. reported highly sensitive silver salts of 5-azido-1*H*-1,2,4-triazole-3-carbonitrile (**HAzCNTr**) and its carboxamidoxime analog (**HAzCOATr**) and tested them as primary explosives. However, their slow deflagration-to-detonation transition is a drawback for this application [465]. A similar compound, 5-azido-3-nitro-1*H*-1,2,4-triazole (**HAzNTr**), was also converted into various salts of alkali metals (Na^+^-Cs^+^), Ag^+^, and Pb^2+^, with Cs^+^ and Ag^+^ salts showing the highest sensitivity [466]. In both cases, parent acids were far less sensitive.

Bis(3-nitroamino-1,2,4-triazo-5-yl)methane (**H_2_BNATrC**) forms salts with alkaline earth metals, which are insensitive. Its salts with transition metals are also insensitive, while H_2_BNATrC itself is highly sensitive (*IS* < 1 J) [467,468]. The monohydrate of its potassium salt, as well as certain onium derivatives, were derived from 1-trinitromethyl-3-nitro-5-nitroamino-1,2,4-triazole (**HTNMNNATr**) and found to be highly sensitive [469]. A Zn^2+^ complex with 4-amino-3-mercaptotriazole (**HASTr**) was proposed as a low-sensitivity energetic material [470].

Further, 1-trinitromethyl-1,2,4-triazole-3-carboxylate served as the parent compound for the K^+^ and Ag^+^ carboxylate salts (anion: **TNMTrCA**), which, when reacted with metal iodides (K, Rb, Cs), formed dinitromethylenide salts (anion: **DNMTrCAH**; in Cs salt, one molecule undergoes both deprotonation and denitration, forming dianion **DNMTrCA**). The resulting EMOFs were characterized as primary explosives [471]. Harter et al. synthesized a series of bridged triazole salts (**HBNATrNAC3**, **HBNATrHC1**, and **HBNATzOC3**) with sodium, potassium, and onium cations. The metal salts were less sensitive than the parent compounds and analogous ammonium or hydroxylammonium derivatives [472].

A MOF containing copper(II), iodate ions, and a triazole ligand (TrDA) was synthesized by Zhang et al., showing lower impact sensitivity than the nitrate salt (the data cited for [Cu(TrDA)_3_](NO_3_)_2_ in this paper differ from those in other literature) [366].

**Figure 23:**
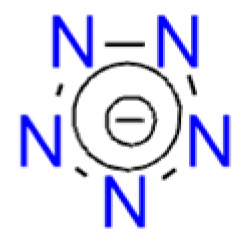
Structure of pentazole anion (Pz^−^).

Pentazole (Figure 23; **HPz**) forms explosive salts with high nitrogen content. However, published reports on their synthesis and characteristics are scant. Sodium, manganese(II), iron(II), iron(III), cobalt(II), aluminum, and magnesium aquacomplexes have been reported as explosive, easily initiated, and prone to spontaneous detonation, though without detailed sensitivity parameters. Only TG/DSC curves and data confirming their rapid, exergonic decomposition have been published [473,474]. Zhang et al. also reported a violent explosion of the Co(II) salt during TG analysis [475]. Impact and friction sensitivities have been reported for silver(I), lithium, potassium, basic lead(II) salts, and a silver-containing complex [476,477,478,479].

Other high-energy materials include 1,2,4-oxadiazoles. This compound is less energetic than its 1,2,5- (furazan) and 1,2,3- isomers (HOF: 216, 158, and 100 kJ mol^−1^, respectively) but remains a promising building block for explosives. Hermann et al. reported salts of 2-(dinitromethyl)-1,2,4-oxadiazoles, including K^+^ and Ba^2+^ derivatives of 2-(dinitromethyl)-1,2,4-oxadiazol-5-one (**H_2_DNMOxon**) and the K^+^ salt of 2-(dinitromethyl)-5-methyl-1,2,4-oxadiazole (**HDNMMOx**). Potassium salts are highly sensitive to impact, while the barium salts can be classified as sensitive [480]. The dimeric 1,2,4-oxadiazole with dinitromethyl groups, **H_2_BDNMOx**, was converted to various salts, including a silver(I) salt, and exhibits high sensitivity in terms of *IS*, *FS*, and *ESD* [481]. A related structure, bis(5-dinitromethyl-1,2,4-oxadiazol-3-yl)methane (**H_2_BDNMOxC**), which contains a methylene bridge, was synthesized as silver and nitrogen–cation salts. Ag_2_BDNMOxC is significantly less sensitive than the methylene-free homolog [482]. Two pyrazole-1,2,4-oxadiazole-linked systems (**HADNPCOx** and **H_2_NADPOx**) containing nitroamine groups were also synthesized and transformed into potassium salts, with the latter, containing more energy-rich groups, showing high sensitivity [483].

Additionally, 1,3,4-oxadiazole has been used in designing high-energy salts [484]. Molecules modified with dinitromethyl or nitroamine groups were converted into potassium salts. Two such materials were synthesized and characterized: 2-dinitrometylo-5-amino-1,3,4-oxadiazole (**HADNM134Ox**) [485], and 5,5′-bis(2-nitroamino-1,3,4-oxadiazole) (**H_2_BNA134Ox**) [486].

**Figure 24:**
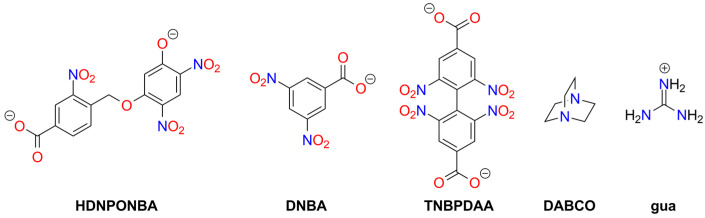
Additional organic anions and ligands used in ECC.

The 3,5-dinitrobenzoic acid (Figure 24; **HDNBA**) salts of transition metals (Co, Ni, and Zn) and their complexes with semicarbazide (**SCZ**) and 1,5-diaminotetrazole (**15DATz**) were synthesized and characterized by Li et al. Their impact sensitivities are between 10 and 40 J, with the highest values obtained for the DNBA-15DATz-Ni system and DNBA-Zn salt. The friction sensitivities of all compounds are low (>360 N) [487]. Additionally, HDNBA complexes with Cu^2+^ and 2,2′-bipyridine (**bpy**) were identified as impact-sensitive, with an IS of 20.7 J [488].

Several MOFs have been proposed using various high-energy ligands. Chen et al. demonstrated the formation of layered materials with variable structures, dependent on crystallization conditions, containing the 1,4-bis(nitroguanidynyl)tetrazine (**H_2_DNGTt**) molecule. These MOFs are classified as less sensitive to impact [489]. Two copper(II)-containing EMOFs were constructed using 5,5′-dinitro-2*H*,2′*H*-3,3′-bi-1,2,4-triazole (**H_2_BNTr**) or its *N*-oxide (**H_2_BNTrO**) and 4,4′-azo-1,2,4-triazole (**TrDA**); the MOF with the N-oxide ligand is impact-sensitive [490]. Additional polymers using the TrDA ligand were studied by Su et al. [491]. They obtained complexes containing sulfate and tetrafluoroborate anions in the form of films on copper plates and compared them with a nitrate-containing MOF. The 4-((5-hydroxy-2,4-dinitrophenoxy)methyl)-3-nitrobenzoic acid (**H_2_HDNPONBA**) forms MOFs with several cations; while these polymers are impact-insensitive, the complex [Cd(HDNPONBA)(H_2_O)(DMF)]‧DMF‧0.5H_2_O undergoes sharp exothermic decomposition at 295 °C, qualifying it as a potential secondary explosive [492]. Similar behavior was reported for polymers of 2,2,6,6-tetranitro-4,4-biphenyl dicarboxylic acid (**H_2_TNBPDAA**) with Ni(II) and Mn(II) cations [493].

Klapötke’s group synthesized and studied an intriguing class of high-energy materials based on bis(azoyl)borates. They characterized the energetic and flame-colorant properties of metal salts derived from dihydrobis(pyrazol-1-yl)borate (**BPBo**), dihydrobis(1,2,4-triazol-1-yl)borate (**BTrBo**), dihydrobis(tetrazol-1-yl)borate (**BTzBo**), dihydrobis(3-nitropyrazol-1-yl)borate (**BNPBo**), dihydrobis(5-aminotetrazol-1-yl)borate (**BATzBo**), dihydrobis(3-nitro-1,2,4-triazol-1-yl)borate (**BNTrBo**), dihydrobis(3,5-dnitropyrazol-1-yl)borate (**BDNPBo**), and dihydrobis(2,4-dinitroimidazol-1-yl)borate (**BDNImBo**). These salts were tested as components in pyrotechnic compositions [494,495,496,497].

Numerous other classes of ligands have been proposed. However, reports on their properties remain relatively scarce. Also, 3,5-Dinitro-2,6-bis(nitroamino)pyrazine (**H_2_DNDNAPr**) has been studied as a potassium salt [498]. Another pyrazine-derived ligand, 5,6-bis(ethylnitroamino)-*N*′2,*N*′3-dihydroxypyrazine-2,3-bis(carboximidamide) (**H_2_ENAPr**), was utilized for synthesizing laser- and mechanically sensitive complexes containing Cu^2+^ or Ni^2+^. Reaction with copper perchlorate induces the cyclization of carboxyimide moieties, leading to the formation of pyrrolo[3,4-*b*]pyrazine formation (**HENAPrP**). The nickel complex forms in the presence of imidazole (**HIm**) and acetonitrile; the latter molecule undergoes hydration and deprotonation, resulting in the anion CH_3_C(NH_2_)(O^−^)_2_ [499].

Bian et al. reported sodium and silver(I) salts of 7-nitro-4-oxo-4,8-dihydro-[1,2,4]triazolo[5,1-d][1,2,3,5]tetrazine 2-oxide (**HMBC**). The sodium derivative was identified as explosive, though its sensitivity to mechanical stimuli is very low (*IS* > 40 N, *FS* > 360 N). Ammonium and hydroxylamine salts of this anion are slightly more sensitive to friction (324 N; DV: 8.3 and 9.1 [km s^−1^]) [500].

Zao et al. continued the studies on tetrazine-2-oxides, publishing data on derivatives of 7,8-dinitro-4-oxo-4,6-dihydropyrazolo[5,1-d][1,2,3,5]tetrazine 2-oxide (**HPCM**), including ammonium, hydroxylamine, hydrazine, and potassium salts. The reported compounds show *IS* = 6.5–10 N, comparable to RDX or HMX (6.5 N), but lower *FS* (170–360 N vs. 120 N) [501].

Fused tetrazole–pyridazine ligands, including 6-nitramino-7-nitro-8-aminotetrazolo[1,5-b]pyridazine (**HANNATzPd**), 6-nitramino-7-nitro-8-hydroxy-tetrazolo[1,5-b]pyridazine (**H_2_HNNATzPd**), and 6-amino-7-nitro-8-hydroxy-tetrazolo[1,5-b]pyridazine (**HHANTzPd**), along with their alkali metal salts, were synthesized and characterized by Chen et al. All salts exhibited impact insensitivity (>20 J) and demonstrated resistance to frictional stimulation (64–108 N) [502].

An interesting material, modified graphene oxide decorated with copper(II) 3-amino-1,3,4-triazolate via alkoxide anions (C-O^−^ Cu^2+^ N^−^), was obtained by Yang et al. The material is classified as insensitive (*IS* = 40 J). However, initiation following an impact at this energy was observed [503]. Another example of a graphene-modified explosive was reported by Liu et al., who characterized a copper(II) complex with 5-(4-amino-5-furazanyl)-1*H*-tetrazol-1-olate (**AFOTz**) in its pure form and as a deposit on graphene oxide and amino-functionalized graphene oxide. The native complex demonstrated higher impact and electric discharge sensitivity but lower friction sensitivity than the graphene-based materials [504].

## 4. Conclusions

Despite our best efforts, we acknowledge that some examples, papers, and data might have been inadvertently omitted, particularly those from older articles, patents, declassified reports, and governmental and laboratory archives. Additionally, certain sources were deliberately excluded: papers and reports focused solely on technical aspects of explosive application, preparation, and production; synthesis documents lacking data on energetic or explosive properties; and redundant materials (such as conference and journal duplicates). For further exploration, we refer readers to recent reviews covering various energetic material topics: nitrogen-rich explosives [505], fused heterocycles [506], nitrated pyrazoles [507], imidazoles [508], 1,2,5-oxadiazoles [187], pentazoles [509], N-oxides [510], catenated nitrogen systems [511], energetic material crystal engineering [511,512], metal-free primary explosives [513], designing high-energetic structures [514], EMOFs [515,516], and ECC’s with acyclic ligands [517]. Also noteworthy are predictive studies on the properties of metal-containing explosives [518]. Comprehensive background, data reviews, and in-depth discussions on energetic materials can also be found in both classic [7,15] and more recent monographs [11,322,323,519,520,521,522,523,524,525,526,527,528,529,530] monographs.

The scale of illegal production of explosive materials by chemistry and pyrotechnics enthusiasts may be illustrated by the number of videos published on social media platforms (Table 2).

**Figure 25:**
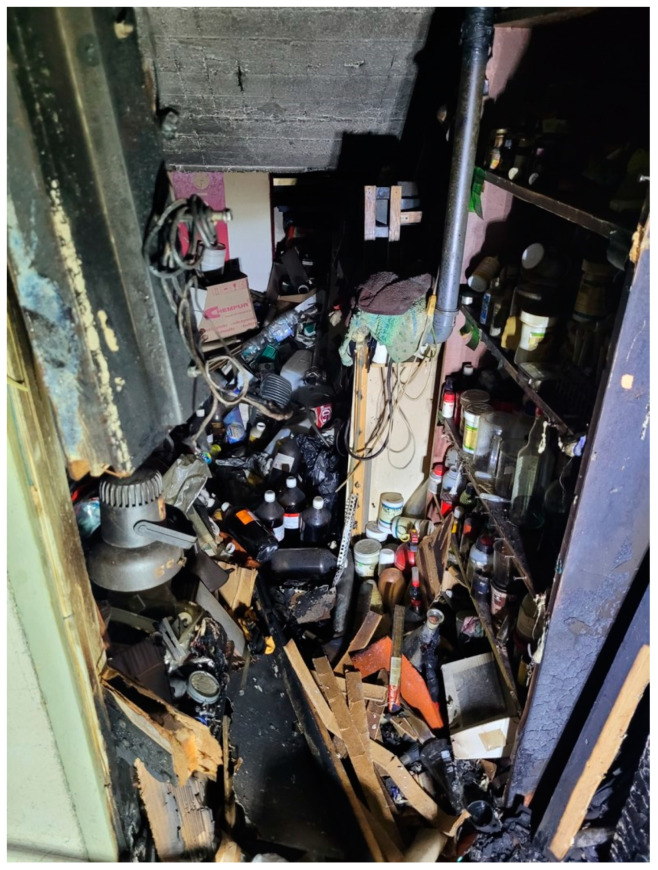
Illegal laboratory containing HMEs discovered after a fire in the basement.

The number of examples provided in specialized discussion boards and websites is even higher. One could expect that the increasing number of published examples will result in greater diversity in the types of HME produced, especially for hobbyist purposes. Security and rescue services should be prepared for accidents involving the explosion of HME and the hazards posed by such materials in cases of other, seemingly non-explosive events (Figure 25).

Currently, the most extensively studied areas of metal compound-based explosives include novel tetrazole derivatives and their applications as green high-energy materials, primers, laser-induced explosives, and components (colorants) for pyrotechnical mixtures. Other key topics include energetic metal–organic frameworks, which are being explored as potential explosives, fuel-burning or decomposition catalysts, and additives for conventional explosives. A specific research aspect, crucial from a security perspective, involves in situ analysis of unknown materials, as well as post-blast studies of explosion residues and HME mixture reactivity [531,532,533,534,535,536,537].

Some overarching conclusions can be drawn:

- **Metal Cations: Enhancing and Stabilizing Explosives:** Metal cations play a significant role in many explosive compounds, sometimes enhancing properties, other times providing molecular stabilization. While some metal-based explosives are in active use or advancing toward commercialization, others remain scientific curiosities or experimental dead-ends.
- **A New Challenge for Security and Identification Technologies:** The vast range of metal-containing explosives presents a considerable challenge for public security agencies. Although exotic compounds may not be of immediate interest to terrorists or typical criminals—who continue to favor readily available substances like TATP, HMDT, LA, and MF—chemistry and pyrotechnics enthusiasts are likely to pursue these compounds, spurring a cycle similar to that seen with designer drugs. As substances are banned, new ones are synthesized, complicating on-site identification for agencies. To address this, Raman and ATR IR devices require updated, extensive spectral libraries. Ensuring accessibility to “positive” spectral data (primarily IR and Raman) in compatible formats could allow portable device libraries to stay current and functional. The extent of illegal metal-based HME production can be observed across open-access platforms like sciencemadness.org and youtube.com.
- **Bringing Historical Explosives into the Modern Era:** Many “legacy” explosives require reevaluation with modern techniques, as older data on their structures and properties may be outdated or uncertain. Historical studies offer valuable inspiration for further research and continued development.
- **Emphasis on Safety and Regulation:** Due to the rapid development of metal-containing explosives and their accessibility to non-professional audiences, heightened safety and regulatory measures may be necessary. As with designer drugs, a proactive regulatory approach could mitigate risks before certain compounds become widely misused. Enhanced international cooperation could also strengthen efforts to monitor and control the circulation of precursor materials.
- **Future Research Directions:** While the study of metal-based explosives has made remarkable progress, further exploration of the environmental impact, stability, and long-term handling safety of these compounds remains essential. Research focused on eco-friendly materials, alongside innovations in detection technologies, could help ensure both environmental responsibility and the safety of practitioners and enforcement personnel.

## Figures and Tables

**Table 1:** Examples of HME accidents in the 21st century.

Year	Location	Description
2001	Paris–Miami flight	PETN with detcord booster and TATP primer (shoe bomb)
2002	Bali, Indonesia	Flash powder (KClO_3_, Al, S), detcord booster
2003	Istanbul, Turkey	Ammonal (NH_4_NO_3_, Al)
2004	Amman, Jordan	HPOM mixture (H_2_O_2_, cummin), nitroglycerine booster, caps as primer
2004	Madrid, Spain	Dynamite
2006	Ontario, Canada	ANFO mixture (NH_4_NO_3_, fuel oil)
2006	Rzeszów, Poland	Explosives’ components: P_red_, Al, HNO_3_, H_2_SO_4_, KNO_3_, KClO_3_, etc.
2008	Sana’a, Yemen	TNT
2010	New York, U.S.	Mixture of NH_4_NO_3_, icing sugar and sawdust, pyrotechnics as primer
2010	UK & UAE	PETN with Hg(CNO)_2_ as primer (bombs in printers)
2011	Lubbock, U.S.	Picric acid with TATP as primer
2011	Oslo, Norway	Main charge: ANFO (NH_4_NO_3_, fuel oil), calcium-ammonium nitrate, Al and phenolic resin microsphaeres; picric acid as booster, diazodinitrophenol as primer
2012	Aurora, U.S.	Black powder (KNO_3_, C, S) with thermite as primer and KMnO_4_/glycerine firestarter
2015	Paris, France	TATP
2015	Edinburgh, UK	Illegal lab with various materials, including mercury(II) and lead(II) picrates
2017	Łódź, Poland	Armstrong’s mixture (KClO_3_, P_red_)—explosion in an illegal lab during a police and firefighters’ intervention that was not related to HME
2019	Płock, Poland	Nitroglycol, HMTD, black powder (KNO_3_, C, S)
2021	Chicago, U.S.	Illegal lab with Pb(N_3_)_2_
2022	Poland	Improvised explosive device containing elaborated pyrotechnics
>2000	Poland	Numerous ATM break-ins with gaseous explosive mixtures (oxygen, acetylene, methane, propane/butane)
2024	Poland	Explosives’ components, including substrates for energetic hydrazino- and amminocomplexes of Cu and Ni
2024	Nashville, U.S.	Poor Man’s C-4 (KClO_3_, vaseline)

**Table 2:** Number of records related to HME in social media.

Tags	Record Number ^a^
perchlorate synthesis, preparation, making, explosion	>9000
perchlorate synthesis, preparation, making, explosion	>30,000
picrate synthesis, preparation, making, explosion	ca. 800
picric acid synthesis, preparation, making, explosion	>9300
styphnate synthesis, preparation, making, explosion	ca. 150
tetrazole, tetrazolate synthesis, preparation, making, explosion	>300
acetylide synthesis, preparation, making, explosion	>2000
fulminate synthesis, preparation, making, explosion	>3500
azide synthesis, preparation, making, explosion	>7600
furoxan, furazane synthesis, preparation, making, explosion	ca. 70
tetraamine copper, tetraammine copper explosion, explosive	ca. 150
hydrazine nickel explosion, explosive	ca. 150
hypophosphite explosion, explosive	ca. 110

^a^ Data from YouTube website; total number of published materials—may include academic, technical, or informational content.

## Supplementary Materials

The following supporting information can be downloaded at: https://www.mdpi.com/article/10.3390/molecules29235588/s1, Table S1: General properties of metal azides, basic salts and some amminacomplexes; Table S2: General properties of metal fulminates; Table S3: General properties of metal pictares; Table S4: General properties of metal styphnates; Table S5: General properties of metal acetylides; Table S6: Physicochemical data of metal-containing explosives. References [538,539,540,541,542,543,544,545,546,547,548,549,550,551,552,553,554,555,556,557,558,559,560,561,562,563,564,565,566,567,568,569,570,571,572,573,574,575] are cited in the Supplementary Materials.
