# Supplementary material for: High-Energetic Salts and Metal Complexes: Comprehensive Overview with a Focus on Use in Homemade Explosives (HME)
Source: Molecules. 2024 Nov 26;29(23):5588. doi: 10.3390/molecules29235588 (PMC11643457; doi:10.3390/molecules29235588)
Supplement: Supplementary file 1 [file molecules-29-05588-s001.zip › molecules-3322890-supplementary.pdf]

High-Energetic Salts and Metal Complexes: Comprehensive Overview with a Focus on Use in Homemade Explosives (HME)

Błażej Gierczyk, Maciej Zalas and Tomasz Otłowski

Supplementary Materials

Abbreviations used:

DV – detonation velocity, *ESD* – electrostatic discharge, *FS* – friction sensitivity, *HOF* – the heat of formation, *IS* – impact sensitivity;
det. – detonation, def. – deflagration, dec. – decomposition, ign. – ignition;
b.p. – boiling point, m.p. – melting point;
(+) - react upon stimuli, (-) – do not react upon stimuli, (n.d.) or balnk field – no data;
HP – hot plate test, HN – hot needle test.
Abbreviations for compounds are provided in figures with compound structures in main text.

Table S1. General properties of metal azides, basic salts and some amminacomplexes.

Name	Formula	m.p. [°C]	HOF [kJ mol^{-1}]	Solubility in water [g/100 mL]	Other	Sensitivity	Reference
Hydrazoic acid	HN_3	-80	294.1	∞	b.p. 37°C; DV: 8.1 (anhydrous) 7.3 (70% aq.) [km s^{-1}]	**very sensitive**, induced by heat, mechanical shock, or explode spontaneously	[538]
Lithium azide	LiN_3	-	10.8	66.4 @ 16°C	dec. ~240°C	explode on thermal shock (~115°C), quite stable on slow heating	[539]
Sodium azide	NaN_3	-	21.3	41.7 @ 17°C	dec. ~350°C	stable	[539,540]
Potassium azide	KN_3	354	-1.4	~49.6 @ 17°C		may explode after heating above m.p.	[539]
Rubidium azide	RbN_3	317	-0.4	114 @ 17°C	dec. ~440°C	sensitive on mechanical shock (*IS* = *40 J*)	[539]
Caesium azide	CsN_3	326	-10.0	307.4 @ 16°C	dec. ~410°C	stable	[539]
Thalium(I) azide	TlN_3	334	234	0.3 @ 17°C		explodes upon heating (ca. 450°C) or mechanical shock (17.5 kg mm^{-2})	[540]
Calcium azide	$Ca(N_3)_2$	-	46.0	45 @ 16°C		explodes when heating (ca. 160°C)	[539]
Magnesium azide	$Mg(N_3)_2$	-	no data	readily sol.	dec. ~450°C	stable deflagrate in flame	[539]
Strontium azide	$Sr(N_3)_2$	-	7.1	45.6 @ 16°C	dec. ~230°C	moderately mechanical shock sensitive and heat sensitive (expl. ~170°C) deflagrate when heated in air	[539]
Barium azide	$Ba(N_3)_2$	-	-22.2	17.8 @ 17°C	dec. ~220°C	heat sensitive (ca. 150-200°C), sensitive on mechanical shock (explosion after *IS* = *10* J) deflagrate when heated in air	[539]
Cadmium azide	$Cd(N_3)_2$	-	451	readily sol.	DV: 4.2 [km s^{-1}]	**very sensitive**; explosion induced by friction, mechanical shock (18.5 kg mm^{-2}) or heating (~345°C), spontaneous explosion reported; explosive in solutions	[539,541]
Mercury(II) azide	$Hg(N_3)_2$	-	556.5	0.26 @ 20°C		**very sensitive**; explosion induced by friction, scratching, mechanical shock (*IS* = *0.33* J) or heating, spontaneous explosions also reported	[31,541]
Nickel(II) azide	$Ni(N_3)_2$	-	no data	readily sol. hydrolyze slowly		**extremely sensitive** on heating (~200°C) or mechanical shock	[7,11,15]
Cobalt(II) azide	$Co(N_3)_2$	-	no data	readily sol. hydrolyze slowly		**extremely sensitive** on heating (~200°C) or mechanical shock	[7,11,15]

Table S1. cont.

Name	Formula	m.p. [°C]	HOF [kJ mol^{-1}]	Solubility in water [g/100 mL]	Other	Sensitivity	Reference
Zinc azide	$Zn(N_3)_2$	α: 335-338<br>β: 327-330	212.6	hydrolyze		highly sensitive on mechanical shock, friction, heating (expl. ~290°C) or electric charges	[542]
Basic zinc azide	$Zn(OH)N_3$	-	no data	insoluble	dec. α ~210°C<br>dec. β ~210°C	less sensitive than $Zn(N_3)_2$	[539,542]
Copper(II) azide	$Cu(N_3)_2$	-	587	0.08	DV: 5-5.5 [km s^{-1}]	**extremely sensitive** on heating (205°C), friction or impact shock (<<1 J & <<1 N) and ESD (<0.28 mJ)	[22,539,543]
Copper(I) azide	CuN_3	-	281	0.008		**extremely sensitive** on heating (~220°C), friction or mechanical shock (*IS* = *0.8* J); explosive also when wet	[543,544]
Basic copper(II) azide	$Cu(N_3)_2{\cdot}n Cu(OH)_2$<br>$n$ = 1-3	-	no data	insoluble		less sensitive than $Cu(N_3)_2$ (expl. ~245°C), deflagrate on impact	[545]
Copper(II) oxyazide	$Cu(N_3)_2{\cdot}8CuO$	-	no data	insoluble		less sensitive than $Cu(N_3)_2$	[7,11,15]
Tetraamminecopper(II) azide	$[Cu(NH_3)_4](N_3)_2$	-	no data			explosive	[546]
Diamminediazidocopper(II)	$(NH_3)_2Cu(N_3)_2$	-	no data	insoluble		deflagrate on impact, explode in flame and on heating (>105°C)	[547]
Silver azide	AgN_3	251	311	0.0008	DV: 1-5 [km s^{-1}]	sensitive on heat (ca. 350°C), friction and mechanical shock (even if wet?); crystals detonate after impact of 0.5 kg weight from 28.5 cm (*IS* = 3 J; *FS* ≤ 0.1 N)	[6,12,110,548]
Chromium(III) azide	$Cr(N_3)_3$	-	no data	hydrolyze		explosive on thermal or mechanical shock	[539]
Hexaamminechromium(III) azide	$[Cr(NH_3)_6](N_3)_3$	-	no data	soluble		explosive upon heating	[7,11,15]
Triamminetriazido-chromium(III)	$Cr(NH_3)_3(N_3)_3$	-	no data	insoluble		explosive upon heating (ca. 250-300°C)	[7,11,15]
Manganese(II) azide	$Mn(N_3)_2$	-	385.9			explosive upon heating (>218°C), mechanical shock and friction	[7,11,15]
Basic manganese(II) azide	$Mn(OH)N_3$	-	no data	soluble		explode after heating, unsensitive on mechanical shock	[539]
Palladium(II) azide	$Pd(N_3)_2$	-	no data	insoluble		**very sensitive**; explode on friction, heating or impact even when wet	[7,11,15]
Potassium hexaazidoplatinate(IV)	$K_2[Pt(N_3)_6]$	-	no data	soluble		explosive spontaneously, solution explosive when heated	[7,11,15]

Table S1. cont.

Name	Formula	m.p. [°C]	HOF [kJ mol^{-1}]	Solubility in water [g/100 mL]	Other	Sensitivity	Reference
Lead(II) azide	$Pb(N_3)_2$	315	α: 483.4<br>β: 484.7	0.02 @ 25°C	DV: 4.6 [km s^{-1}]	**primary explosive**; highly heat, friction and shock sensitive (*IS* = 4 J, *FS* ≤ 1N, *ESD* = 0.007-5 mJ); detonation temperature ~320°C, may explode during crystalization	[10,12,110,540,548-550]
Lead(II) azide chloride (1:1)	$PbClN_3$	-	no data	insoluble	dec. 290°C	much less explosive than $Pb(N_3)_2$; explode at ca. 300°C	[551]
Lead(II) oxyazide	$Pb(N_3)_2{\cdot}PbO$[a]		no data	insoluble		deflagrate or explode upon heating or flame, detonation temperature ~350°C, less sensitive to mechanical impact than $Pb(N_3)_2$ but of similar explosion properties	[11,14]
Lead(IV) azide	$Pb(N_3)_4$	-	no data	soluble		explode spontaneously	[7,11,15]
Dimercury(I) azide	$Hg_2(N_3)_2$	225 (dec.)	592	0.03 @ r.t.		**very sensitive**; explosion induced by mechanical shock (0.5 kg weight dropping from 6 cm) or heating (ca. 270-290°C), spontaneous explosions also reported	[31,110,541]
mer-Triazido-ethylenediamine-pyridinecobalt(III)	$[Co(N_3)_3(en)(py)]$	n.d.	n.d.	insoluble		**very sensitive**	[46]
Azidopentaamminecobalt(III) azide	$[Co(NH_3)_5(N_3)](N_3)_2$	n.d.	n.d.	n.d.		explosive upon heating (195-200°C)	[43]
Diazidotetraamminecobalt(III) azide[b]	$[Co(NH_3)_4(N_3)_2](N_3)$	n.d.	n.d.	n.d.		explosive upon heating (182°C) and after impact	[44]
Diazidotetraamminecobalt(III) iodide[b]	$[Co(NH_3)_4(N_3)_2]I$	n.d.	n.d.	n.d.		explosive upon heating (180°C)	[44]
Diazidotetraamminecobalt(III) nitrate[b]	$[Co(NH_3)_4(N_3)_2](NO_3)$	n.d.	n.d.	n.d.		deflagrate upon heating (150-180°C), explode after impact	[44]
Diazidotetraamminecobalt(III) perchlorate[b]	$[Co(NH_3)_4(N_3)_2](ClO_4)$	n.d.	n.d.	n.d.		deflagrate upon heating (200-220°C), explode after impact	[44]
Triazido-triamminecobalt(III)	$[Co(NH_3)_3(N_3)_3]$	n.d.	n.d.	n.d.		**highly sensitive** on heating (187°C), when impactd or fricted	[45]
Hexaamminecobalt(III) hexaazidocobaltate(III)	$[Co(NH_3)_6][Co(N_3)_6]$	n.d.	n.d.	n.d.		detonate upon heating, mechanical shock or spontaneously	[47]
Hexaamminechromium(III) hexaazidocobaltate(III)	$[Cr(NH_3)_6][Co(N_3)_6]$	n.d.	n.d.	n.d.		detonate upon heating, mechanical shock or spontaneously	[47]
Hexaamminerhodium(III) hexaazidocobaltate(III)	$[Rh(NH_3)_6][Co(N_3)_6]$	n.d.	n.d.	n.d.		detonate upon heating, mechanical shock or spontaneously	[47]

Table S1. cont.

Name	Formula	m.p. [°C]	HOF [kJ mol^{-1}]	Solubility in water [g/100 mL]	Other	Sensitivity	Reference
trans-Diazidotetraamminecobalt(III) *trans*-tetraazidodiammine-cobaltate(III)	$[Co(NH_3)_4(N_3)_2][Co(N_3)_6]$	n.d.	n.d.	n.d.		reported as "strong detonator" upon heating	[48]

[a] a dozen of forms differ in stoichiometry and hydration degree [14]

[b] both *cis* and *trans* isomers [44]

Table S2. General properties of metal fulminates.

Name	Formula	m.p. [°C]	HOF [kJ mol-1]	Solubility in water [g/100 mL]	Other	Sensitivity	Reference
Fulminic acid	HCNO	-10		∞		insensitive, polymerize readily	[7,11,15]
Mercury(II) fulminate	$Hg(CNO)_2$	-	273	0.07 @ 12°C	DV: 2-5 [km s^{-1}]	**very sensitive** to mechanical shock and discharge, friction sensitive<br>*IS* = 5 J; *FS* ≤ 0.1-10 N (grains: 50-300 μm)	[10,12,548]
Silver fulminate	AgCNO	-	180	0.02 @ 30°C	DV: 1.7 [km s^{-1}]<br>dec. 196°C	**extremely sensitive** to friction and discharge, **very sensitive** to impact or heat, may explode if light-irradiated, explosive even when moist<br>*IS* = 5 J; *FS* ≤ 0.1 N; *ESD* ≤ 0.28 (grains: 100-200 μm)	[548]
Sodium fulminte	NaCNO	-	n.d.	soluble		sensitive to impact, friction and heat (210-220°C)	[7,11,15]
Potassium fulminate	KCNO	-	n.d.	soluble		sensitive to impact, friction and heat (200°C)	[7,11,15]
Rubidium fulminate	RbCNO	-	n.d.	n.d.		extremely sensitive to impact, friction and heat (195°C)	[7,11,15]
Caesium fulminate	CsCNO	-	n.d.	n.d.		extremely sensitive to impact, friction and heat (220-225°C)	[7,11,15]
Cadmium fulminate	$Cd(CNO)_2$	-	163	easily soluble, hydrolyze		sensitive to impact, friction and heat (215°C)	[7,11,15]
Copper(I) fulminate	CuCNO	-	n.d.	insoluble		sensitive to impact, friction and heat	[7,11,15]
Copper(II) fulminate		-	n.d.	n.d.		sensitive to impact, friction and heat (205°C)	[7,11,15]
Thallium(I) fulminate	TlCNO	-	n.d.	hydrolyze		sensitive to impact, friction and heat (120°C)	[7,11,15]

Table S3. General properties of metal pictares.

Name	Formula	m.p. [°C]	HOF [kJ mol^{-1}]	Solubility in water [g/100 mL]	Other	Sensitivity	Reference
Picric acid	HPA	122.5	-214	1.11 @ 20°C	DV: 4-7.6 [km s^{-1}]	sensitive on impact higher than TNT, low friction sensitivity *IS* = 16-50 J, *FS* > 360, *ESD* 8980 mJ	[519,552,553]
Lithium picrate	LiPA	301	n.d.	n.d.		impact sensitive, heat sensitive (expl. 325°C)	[554-556]
Sodium picrate	NaPA	210	n.d.	4.3 @ 20°C		impact (*19* J) and heat sensitive (expl. 313°C)	[15,553-556]
Potassium picrate	KPA	250	-499	0.51 @ 20°C	DV: 3.5-5.5 [km s^{-1}]	impact (*4.2* J) and heat sensitive (expl. 331°C)	[12,15,83,552,554-557]
Rubidium picrate	RbPA	233	n.d.	0.38 @ 20°C		friction and impact sensitive, heat sensitive (expl. 336°C)	[83,554-556]
Caesium picrate	CsPA	276	n.d.	0.31 @ 20°C		friction and impact sensitive, heat sensitive (expl. 300°C)	[554-556]
Thallium(I) picrate	TlPA	320 (dec.)	n.d.	1.04 @ 45°C		impact sensitive, heat sensitive (expl. 301°C)	[558]
Silver picrate	AgPA	296	n.d.	1.68 @ 25°C		friction, impact and heat sensitive (expl. 322°C)	[12,110,556]
Beryllium picrate	$BePA_2$	114	n.d.	readily sol.		no data	[559]
Magnesium picrate	$MgPA_2$	dec.	n.d.	10 @ 22°C		impact sensitive, heat sensitive (expl. 368°C)	[15,559,560]
Calcium picrate	$CaPA_2$	316	n.d.	50 @ 20°C		impact sensitive, heat sensitive (expl. 326°C)	[15,555,559,560]
Strontium picrate	$SrPA_2$	290	n.d.	1.4 @ 20°C		impact sensitive, heat sensitive (expl. 341°C)	[15,555,559,560]
Barium picrate	$BaPA_2$	200	n.d.	1.2 @ 20°C		impact sensitive, heat sensitive (expl. 332°C)	[15,110,555,559,560]
Lead picrate	$PbPA_2$	n.d.	n.d.	0.71 @ 25°C		**high sensitive** on impact (*0.2* J) and heat (expl. 281°C)	[12,15,65,83,110,552]
Copper(II) picrate	$CuPA_2$	305 (dec.)	n.d.	n.d.		impact (*6* J) and heat sensitive (expl. 290°C)	[15,110,553,556]
Zinc picrate	$ZnPA_2$	n.d.	n.d.	12.5		impact sensitive, heat sensitive (expl. 303°C)	[15,553,559]
Manganese(II) picrate	$MnPA_2$	n.d.	n.d.	n.d.		impact (*60* J) and heat sensitive (expl. 298°C)	[15,561]
Iron(II) picrate	$FePA_2$	275.5	n.d.	n.d.		impact sensitive, heat sensitive (expl. 296°C)	[553,561,562]
Iron(III) picrate	$FePA_3$	n.d.	n.d.	n.d.		impact (*2* J) and heat sensitive (expl. 247°C)	[15,553]
Chromium(III) picrate	$CrPA_3$	275.5	n.d.	n.d.		impact (*5.6* J) and heat sensitive (expl. 296.5°C)	[15,553,561]
Nickel(II) picrate	$NiPA_2$	n.d.	n.d.	n.d.		impact (*40* J) and heat sensitive (expl. 348°C)	[15,553,561]
Cobalt(II) picrate	$CoPA_2$	226	n.d.	n.d.		impact sensitive, heat sensitive (expl. 327.5°C)	[561,563]
Mercury(II) picrate	$HgPA_2$	290	n.d.	n.d.		impact sensitive, heat sensitive (expl. 333°C)	[559]
Cadmium picrate	$CdPA_2$	dec.	n.d.	n.d.		impact sensitive, heat sensitive (expl. 328°C)	[553,559]
Aluminum picrate	$AlPA_3$	dec.	n.d.	n.d.		impact (*3.8* J) and heat sensitive (expl. 360-395°C)	[553,558]

Table S4. General properties of metal styphnates.

Name	Formula	m.p. [°C]	HOF [kJ mol^{-1}]	Solubility in water [g/100 mL]	Other	Sensitivity	Reference
Styphnic acid	$TNRH_2$	174-175	-523	0.64 @ 25°C	DV: 7.5 [km s^{-1}]	impact sensitivity similar to picric acid, explodes at 314°C *IS* = 7.4-10.5 J, *FS* > 353 N, *ESD* 230-1230 mJ	[519]
Lead(II) styphnate monohydrate	$TNRPb{\cdot}H_2O$	n.d.	835	0.07 @ 20°C	DV: 2.1-5.6 [km s^{-1}]	**extremely sensitive** to flame and spark (0.04-0.14 mJ), sensitive to impact (2.3-5 J) and friction (1.45 N), explode upon heating (235-280°C)	[10,79,110,564]
Basic lead(II) styphnate	$TNRPb{\cdot}Pb(OH)_2$	α: 95-100 β: 60-70	ca. 980	0.0008 @ 20°C		flame, heat (expl. 250-290°C), impact, friction and discharge sensitive	[79]
Barium styphnate monohydrate	$TNRBa{\cdot}H_2O$	dec.	n.d.	slightly sol.		heat (expl. 345-360°C), flame and impact sensitive	[564]
Monothallium(I) styphnate	TNRTlH	dec.	492	slightly sol.		heat (expl. 242°C) sensitive	[84]
Dithallium(I) styphnate	$TNRTl_2$	dec.	528	slightly sol.		heat (expl. 242°C) sensitive	[84]
Silver styphnate monohydrate	$TNRAg_2{\cdot}H_2O$	n.d.	ca. 275	insoluble		heat (expl. 286°C) sensitive, sensitivity on mechanical stimuli similar to StPb	[85,110]

Table S5. General properties of metal acetylides.

Name	Formula	m.p. [°C]	HOF [kJ mol-1]	Solubility in water [g/100 mL]	Other	Sensitivity	Reference
Silver acetylide	Ag_2C_2	dec.	352	insoluble	DV: 1.2 [km s^{-1}]	**highly sensitive** to impact (0.7-3.2 J), friction (0.1-0.2 N); ignites when heated (140-200°C); explosive reacting with some reagents (e.g. Cl_2)	[10,12,110,548,565]
Silver acetylide silver nitrate(V) 1:1	$Ag_2C_2{\cdot}AgNO_3$	dec.	213	insoluble	DV: 2.2-3.5 [km s^{-1}]	sensitive to impact (2.1-3.9 J), friction (1 N); ignites when heated (200-220°C)	[10,12,110]
Silver acetylide silver nitrate(V) 1:6	$Ag_2C_2{\cdot}6AgNO_3$	dec.	n.d.	soluble		hardly explosive or even nonexplosive; decomposing when heated (308-327°C)	[104,107]
Copper(I) acetylide	Cu_2C_2	dec.	n.d.	insoluble		moderately sensitive on friction (50 N), easily explodes upon spark, ignites when heated (100-270°C)	[117,118]
Copper(II) acetylide	CuC_2	dec.	n.d.	insoluble		highly sensitive on mechanical stimuli or heating (50-120°C); explosive reacting with some reagents (e.g. acids, KCN)	[118]
Mercury(II) acetylide	HgC_2	dec.	no data	insoluble		no data	[566,567]
Dimercury(I) acetylide hydrate	$Hg_2C_2{\cdot}H_2O$	dec.	no data	insoluble		no data	[568]
Gold(I) acetylide	Au_2C_2	dec.	no data	insoluble		**highly sensitive** to friction and impact, exploding when heated; ignited at 83-157°C	[569]

Table S6. Physicochemical data of metal-containing explosives.

Compound	*IS* [J]	*FS* [N]	*ESD* [mJ]	Grain size [μm]	N content [%]	T_{dec} [°C]	*d* [g cm^{-3}]	*HOF* [kJ mol^{-1}]	ΔU_{ex} [kJ kg^{-1}]	*DV* [m s^{-1}]	p_{ex} [kbar]	HN	HP	Laser test	Ref.
**AZIDES**															
[Cu(1MTz)(N$_3$)$_2$]	<1	<0.1	0.79	<100	60.5	148	2.036	n.d.	n.d.	n.d.	n.d.	n.d.	n.d.	n.d.	[22]
[Cu(1ETz)(N$_3$)$_2$]	3	4.5	33	<100	57.0	134	1.931	n.d.	n.d.	n.d.	n.d.	n.d.	n.d.	n.d.	[22]
[Cu(1PTz)(N$_3$)$_2$]	2.5	10	112	<100	53.9	148	1.880	n.d.	n.d.	n.d.	n.d.	n.d.	n.d.	n.d.	[22]
[Cu(1cPTz)(N$_3$)$_2$]	<1	5	37	<100	54.4	138	1.863	n.d.	n.d.	n.d.	n.d.	def.	def.	n.d.	[19]
[Cu(1cBTz)(N$_3$)$_2$]	2	60	50	<100	51.6	126	1.797	n.d.	n.d.	n.d.	n.d.	def.	def.	n.d.	[19]
[Cu(1cPeTz)(N$_3$)$_2$]	5	216	76	<100	49.0	188	1.775	n.d.	n.d.	n.d.	n.d.	def.	def.	n.d.	[19]
[Cu(1A123Tr)(N$_3$)$_2$]	3	<5	5	<100	60.5	123	2.093	n.d.	n.d.	n.d.	n.d.	n.d.	n.d.	n.d.	[550]
[Cu(4ATr)$_2$(N$_3$)$_2$]	10	>360	300	<100	62.1	161	1.952	n.d.	n.d.	n.d.	n.d.	n.d.	n.d.	n.d.	[550]
[Cu$_3$(TATr)$_4$(N$_3$)$_6$]·0.5H$_2$O	15	>360	250	<100	64.8	170	1.975	n.d.	n.d.	n.d.	n.d.	n.d.	n.d.	n.d.	[550]
[Cu(5BTr)(N$_3$)$_2$]	2	≤0.1	5	<100	58.5	195	2.065	n.d.	n.d.	n.d.	n.d.	det.	det.	det.	[550]
[Cu(1AzMTz)(N$_3$)$_2$]	<1	<0.1	38	n.d.	66.8	147	1.93	n.d.	n.d.	n.d.	n.d.	det.	det.	n.d.	[20]
[Cu(1AzETz)(N$_3$)$_2$]	<1	≤0.1	1.1	<100	63.5	131	2.003	n.d.	n.d.	n.d.	n.d.	det.	det.	def.	[550]
[Cu$_2$(2AzETz)(N$_3$)$_4$]	<1	<0.1	2.5	<100	61.3	143	n.d.	n.d.	n.d.	n.d.	n.d.	det.	det.	n.d.	[358]
[Cu(1AzPTz)(N$_3$)$_2$]	<1	4.5	2.5	<100	60.6	132	1.897	n.d.	n.d.	n.d.	n.d.	n.d.	n.d.	n.d.	[550]
[Cu$_4$(2M5ATz)$_2$(N$_3$)$_8$]	<1	<5	13	<100	60.4	161	2.188	n.d.	n.d.	n.d.	n.d.	det.	det.	det.	[550]
[Cu$_4$(22DTE)(N$_3$)$_8$]	<1	<5	5	<100	58.3	190	2.200	n.d.	n.d.	n.d.	n.d.	det.	det.	det.	[550]
[Cu$_2$(12BC3)(N$_3$)$_4$]	3	<5	18	<100	58.9	146	2.136	n.d.	n.d.	n.d.	n.d.	det.	det.	det.	[550]
[Cu$_4$(22DTP)(N$_3$)$_8$]	1	≤0.1	5	<100	58.2	195	n.d.	n.d.	n.d.	n.d.	n.d.	det.	det.	det.	[550]
[Cu$_2$(11BTziP)(N$_3$)$_4$]	2	<5	5	<100	58.9	165	n.d.	n.d.	n.d.	n.d.	n.d.	det.	det.	def.	[550]
[Cu$_2$(2APr)(N$_3$)$_2$]$_n$	-	def.	n.d.	n.d.	41.3	n.d.	2.311	n.d.	n.d.	n.d.	n.d.	n.d.	n.d.	n.d.	[41]
[Co(NH$_3$)$_6$](N$_3$)$_3$	*4.4*	n.d.	n.d.	~100	73.2	n.d.	n.d.	n.d.	n.d.	n.d.	n.d.	n.d.	n.d.	n.d.	[23]
[Co(NH$_3$)$_5$(N$_3$)](N$_3$)$_2$	*3.4*	n.d.	n.d.	~100	72.6	n.d.	n.d.	n.d.	n.d.	n.d.	n.d.	n.d.	n.d.	n.d.	[23]
trans-[Co(NH$_3$)$_4$(N$_3$)$_2$]N$_3$	*2.6*	n.d.	n.d.	~100	71.9	n.d.	n.d.	n.d.	n.d.	n.d.	n.d.	n.d.	n.d.	n.d.	[23]
cis-[Co(NH$_3$)$_4$(N$_3$)$_2$]N$_3$	*1.4*	n.d.	n.d.	~100	71.9	n.d.	n.d.	n.d.	n.d.	n.d.	n.d.	n.d.	n.d.	n.d.	[23]
[Co(N$_3$)$_3$(NH$_3$)$_3$]	*0.56*	n.d.	n.d.	0.1-10	71.2	n.d.	n.d.	n.d.	n.d.	n.d.	n.d.	n.d.	n.d.	n.d.	[23]
[Co(TrDA)(N$_3$)$_2$]$_n$	1.2	5	4.1	n.d.	63.8	246	2.094	1292.9	4407	7672	285	n.d.	n.d.	n.d.	[33]
[Cd(TrDA)(N$_3$)$_2$]$_n$	1.6	12	8.9	n.d.	54.4	273	2.282	1471.2	4248	7538	287	n.d.	n.d.	n.d.	[33]
[Co(11DTE)(N$_3$)$_2$]$_n$	1.5	48	28	n.d.	63.4	253	1.950	1263.7	4390	7339	250	n.d.	n.d.	n.d.	[36]
[Cd(11DTE)(N$_3$)$_2$]$_n$	1.8	80	30	n.d.	54.1	212	2.108	1423.2	4180	7088	244	n.d.	n.d.	n.d.	[36]
[Cd$_2$(11DTE)$_2$(N$_3$)$_3$Cl]$_n$	2.0	360	20	n.d.	48.7	220	2.137	2056.6	3390	6476	205	n.d.	n.d.	n.d.	[36]
[Cd$_2$(11DTE)$_2$(N$_3$)$_2$Br$_2$]$_n$	2.0	360	18	n.d.	38.5	216	2.362	2071.9	3160	6119	193	n.d.	n.d.	n.d.	[36]
Cu(HIm)$_4$(N$_3$)$_2$	4.16	*1.23 MPa*	n.d.	n.d.	46.7	190	1.616	-2492.6	11090	n.d.	n.d.	n.d.	n.d.	n.d.	[21]
[Ni(HIm)$_4$(N$_3$)$_2$]	14.7	*3.91 MPa*	n.d.	n.d.	47.3	180	1.480	-4731.2	16710	n.d.	n.d.	n.d.	n.d.	n.d.	[21]

Table S6. cont.

Compound	*IS* [J]	*FS* [N]	*ESD* [mJ]	Grain size [µm]	N content [%]	T_{dec} [°C]	*d* [g cm^{-3}]	*HOF* [kJ mol^{-1}]	ΔU_{ex} [kJ kg^{-1}]	*DV* [m s^{-1}]	p_{ex} [kbar]	HN	HP	Laser test	Ref.
[Co(hz)$_2$(N$_3$)$_2$]	*0.45*	n.d.	n.d.	n.d.	67.6	223	n.d.	n.d.	n.d.	n.d.	n.d.	n.d.	n.d.	n.d.	[24]
[Ni(hz)$_2$(N$_3$)$_2$]	*0.5*	n.d.	n.d.	n.d.	67.7	196	n.d.	n.d.	n.d.	n.d.	n.d.	n.d.	n.d.	n.d.	[24]
[Zn(hz)$_2$(N$_3$)$_2$]	*0.6*	n.d.	n.d.	n.d.	65.6	212	n.d.	n.d.	n.d.	n.d.	n.d.	n.d.	n.d.	n.d.	[24]
	28.5	*3.92 MPa*	n.d.	n.d.		205	n.d.	n.d.	5450	n.d.	n.d.	n.d.	n.d.	n.d.	[25]
[Cd$_2$(hz)$_2$(N$_3$)$_4$]	*4.0*	*1.92 MPa*	n.d.	n.d.	49.0	222	2.638	n.d.	n.d.	n.d.	n.d.	n.d.	n.d.	n.d.	[26]
[Cd(en)(N$_3$)$_2$]	*3.3*	*1.96 MPa*	n.d.	n.d.	43.7	265	2.090	n.d.	n.d.	n.d.	n.d.	n.d.	n.d.	n.d.	[27]
[Ni(ANQ)$_2$(N$_3$)$_2$]$_n$	1.5	64	n.d.	n.d.	58.8	232	1.98	n.d.	n.d.	7500	235	def.	n.d.	n.d.	[28]
[Co(ANQ)$_2$(N$_3$)$_2$]$_n$	4	72	n.d.	n.d.	58.8	216	1.94	n.d.	n.d.	7200	209	def.	n.d.	n.d.	[28]
[Cd$_2$(34DATr)$_2$(N$_3$)$_2$]$_n$	>40	>360	n.d.	n.d.	44.4	401	2.581	1589.4	3390	n.d.	n.d.	n.d.	n.d.	n.d.	[37]
[Cd(BTr)$_2$(N$_3$)$_2$]$_n$	8	360	n.d.	n.d.	53.8	255	1.968	1476.4	3412	n.d.	n.d.	n.d.	n.d.	n.d.	[37]
[Cd(11DTP)(N$_3$)$_2$]$_n$	2.5	288	n.d.	n.d.	52.1	181	1.080	1363.7	3947	n.d.	n.d.	n.d.	n.d.	n.d.	[37]
[Cd$_2$(11DTB)(N$_3$)$_4$]$_n$	2	240	n.d.	n.d.	47.7	230	0.989	2255	4504	n.d.	n.d.	n.d.	n.d.	n.d.	[37]
[Cu(N$_3$)$_2$(1NOETz)]	3	1	14	<100	50.2	122	1.985	n.d.	n.d.	n.d.	n.d.	det.	det.	det.	[89]
[Cu(N$_3$)(15DTM)]·H$_2$O	<1	50	6	<100	56.1	208	2.017	n.d.	n.d.	n.d.	n.d.	det.	det.	det.	[73]
[Cu(N$_3$)(25DTM)]·H$_2$O	<1	7.5	13	<100	56.1	128	n.d.	n.d.	n.d.	n.d.	n.d.	det.	det.	det.	[73]
**FULMINATES**															
[Ag$_2$(CNO)$_2$(4ATr)]·H$_2$O	>40	>360	>1000	100-500	20.9	187	2.992	n.d.	n.d.	n.d.	n.d.	dec.	def.	n.d.	[60]
[Ag$_4$(CNO)$_4$(BTr)]	5	10	2.5	<100	19.0	215	3.406	n.d.	n.d.	n.d.	n.d.	def.	det.	n.d.	[60]
[Ag$_3$(CNO)$_3$(15DMTz)$_3$]·3H$_2$O	>40	>360	740	<100	26.3	138	2.204	n.d.	n.d.	n.d.	n.d.	dec.	def.	n.d.	[60]
[Ag$_4$(CNO)$_4$(1cBTz)$_2$]	20	84	7.3	100-500	19.8	133	2.454	n.d.	n.d.	n.d.	n.d.	def.	det.	n.d.	[60]
[Ag$_3$(CNO)$_3$(11DTM)$_3$]	6	72	25	100-500	41.7	152	2.329	n.d.	n.d.	n.d.	n.d.	def.	def.	n.d.	[60]
[Ag$_3$(CNO)$_3$(12DTM)$_3$]	7	64	203	100-500	41.7	144	2.259	n.d.	n.d.	n.d.	n.d.	def.	def.	n.d.	[60]
[Ag$_4$(CNO)$_4$(22DTP)]	8	1	0.7	<100	21.6	177	2.891	n.d.	n.d.	n.d.	n.d.	det.	det.	n.d.	[60]
[Ag$_2$(CNO)$_2$(1NOETz)]	≤1	20	250	>1000	21.4	123	2.718	n.d.	n.d.	n.d.	n.d.	def.	det.	det.	[89]
[Ag(CNO)(1NOETz)]	9	60	250	100-500	27.2	102	2.337	n.d.	n.d.	n.d.	n.d.	dec.	det.	-	[89]
[Ag$_4$(CNO)$_4$(1AzMTz)$_2$]	<1	<0.1	10	n.d.	29.7	109	2.71	n.d.	n.d.	n.d.	n.d.	det.	det.	n.d.	[20]
**NITROPHENOLATES**															
[Cu(PA)$_2$(MSC)$_2$]	30	>360	300	<100	21.1	179	1.885	n.d.	n.d.	n.d.	n.d.	n.d.	n.d.	n.d.	[75]
[Zn(PA)$_2$(MSC)$_2$]	10	360	1000	<100	26.6	190	n.d.	n.d.	n.d.	n.d.	n.d.	n.d.	n.d.	n.d.	[75]
[Mn(PA)$_2$(MSC)$_2$]	9	288	550	<100	24.4	190	1.825	n.d.	n.d.	n.d.	n.d.	n.d.	n.d.	n.d.	[75]
[Co(PA)$_2$(MSC)$_2$]	5	288	300	<100	24.2	211	1.866	n.d.	n.d.	n.d.	n.d.	n.d.	n.d.	n.d.	[75]
[Co(PA)$_3$(MSC)$_2$]	10	>360	750	<100	26.9	217	n.d.	n.d.	n.d.	n.d.	n.d.	n.d.	n.d.	n.d.	[75]
[Cu(MSC)$_2$(H$_2$O)$_2$](HTNR)$_2$	>40	>360	400	<100	21.9	179	1.880	n.d.	n.d.	n.d.	n.d.	n.d.	n.d.	n.d.	[75]

Table S6. cont.

Compound	*IS* [J]	*FS* [N]	*ESD* [mJ]	Grain size [µm]	N content [%]	T_{dec} [°C]	*d* [g cm^{-3}]	*HOF* [kJ mol^{-1}]	ΔU_{ex} [kJ kg^{-1}]	*DV* [m s^{-1}]	p_{ex} [kbar]	HN	HP	Laser test	Ref.
$[Zn(MSC)_2(H_2O)_2](HTNR)_2$	>40	>360	1000	<100	21.9	195	1.866	n.d.	n.d.	n.d.	n.d.	n.d.	n.d.	n.d.	[75]
$[Mn(HTNR)_2(H_2O)_2]$	25	324	600	<100	23.3	184	1.892	n.d.	n.d.	n.d.	n.d.	n.d.	n.d.	n.d.	[75]
$[Co(MSC)_2(H_2O)_2](HTNR)_2$	>40	>360	1500	<100	22.1	205	1.852	n.d.	n.d.	n.d.	n.d.	n.d.	n.d.	n.d.	[75]
$[Ni(MSC)_2(H_2O)_2](HTNR)_2$	>40	>360	1500	<100	22.1	193	1.844	n.d.	n.d.	n.d.	n.d.	n.d.	n.d.	n.d.	[75]
$[Cu(H_2O)_4(11DTM)_2](PA)_2$	6	>360	840	100-500	34.4	185	1.757	n.d.	n.d.	n.d.	n.d.	dec.	dec.	n.d.	[67]
$[Cu(TNR)(11DTM)]\cdot H_2O$	2	192	188	100-500	32.3	236	1.973	n.d.	n.d.	n.d.	n.d.	dec.	def.	n.d.	[67]
$[Cu(H_2O)_4(11DTM)_2](H_2TNPG)_2$	4	48	960	100-500	32.1	196	1.810	n.d.	n.d.	n.d.	n.d.	dec.	def.	n.d.	[67]
$[Cu(H_2O)_2(12DTM)_2](PA)_2$	1	>360	750	100-500	35.8	159	1.860	n.d.	n.d.	n.d.	n.d.	dec.	dec.	n.d.	[67]
$[Cu(H_2O)_2(12DTM)_2](HTNR)_2$	7	144	1500	100-500	34.6	156	1.872	n.d.	n.d.	n.d.	n.d.	dec.	def.	n.d.	[67]
$[Cu(H_2O)_2(11DTM)_2](H_2TNPG)_2$	1.5	128	1350	100-500	33.4	136	2.020	n.d.	n.d.	n.d.	n.d.	dec.	def.	n.d.	[67]
$[Cu(H_2O)_4(1A123Tr)_2](PA)_2$	>40	>360	1220	500-1000	25.8	188	1.825	n.d.	n.d.	n.d.	n.d.	n.d.	n.d.	n.d.	[68]
$[Cu(HTNR)_2(1A123Tr)_2]$	1.5	80	40	500-1000	27.2	177	1.842	n.d.	n.d.	n.d.	n.d.	n.d.	n.d.	n.d.	[68]
$[Cu(H_2O)_2(1A123Tr)_4](H_2TNPG)_2$	2	160	300	500-1000	32.2	153	1.840	n.d.	n.d.	n.d.	n.d.	n.d.	n.d.	n.d.	[68]
$[Cu(\mu\text{-}TNR)(1A123Tr)_2]$	3	60	50	500-1000	32.5	194	1.876	n.d.	n.d.	n.d.	n.d.	n.d.	n.d.	n.d.	[68]
$[Cu(\mu\text{-}TNR)(1A124Tr)_2]$	30	240	90	500-1000	32.5	237	1.888	n.d.	n.d.	n.d.	n.d.	n.d.	n.d.	n.d.	[68]
$[Cu(PA)_2(15DMTz)_2]$	8	>360	n.d.	n.d.	27.4	250	1.67	n.d.	n.d.	n.d.	n.d.	n.d.	n.d.	n.d.	[69]
$[Cu(HTNR)_2(15DMTz)_2]$	2	>360	n.d.	n.d.	26.2	184	n.d.	n.d.	n.d.	n.d.	n.d.	n.d.	n.d.	n.d.	[69]
$[Cu(HTNPG)\ (15DMTz)_2]$	2	360	n.d.	n.d.	29.7	192	1.72	n.d.	n.d.	n.d.	n.d.	n.d.	n.d.	n.d.	[69]
H_2TNO	>40	324	540	100-500	16.2	n.d.	1.71	-436.7	4024	6987	198	n.d.	n.d.	n.d.	[100]
H_2TNR	7.4-10.5	353	230-1230	n.d.	17.2	223	1.83	-523	3969	7522	237	n.d.	n.d.	n.d.	[519]
	n.d.	n.d.	n.d.	n.d.		n.d.	1.83	-374.3	4509	7668	252	n.d.	n.d.	n.d.	[100]
$[Cu(1ATz)_2(HTNO)_2]$	<1	96	50	100-500	29.9	185	1.904	n.d.	n.d.	n.d.	n.d.	dec.	def.	n.d.	[100]
$[Cu(2ATz)_2(HTNO)_2]$	<1	80	50	100-500	29.9	182	1.812	n.d.	n.d.	n.d.	n.d.	def.	def.	n.d.	[100]
$[Cu(1A5MTz)_2(HTNO)_2]$	<1	60	250	100-500	28.8	192	n.d.	n.d.	n.d.	n.d.	n.d.	dec.	def.	n.d.	[100]
$[Cu(1M5ATz)_2(HTNO)_2]$	2	>360	90	100-500	28.8	220	1.802	n.d.	n.d.	n.d.	n.d.	dec.	def.	n.d.	[100]
$[Cu(15DATz)_2(HTNO)_2]$	<1	360	200	100-500	32.3	220	1.848	n.d.	n.d.	n.d.	n.d.	dec.	def.	n.d.	[100]
$[Cu(1NOETz)_2(HTNO)_2]$	<1	192	90	100-500	25.0	159	1.817	n.d.	n.d.	n.d.	n.d.	dec.	def.	n.d.	[100]
$[Cu(1AzETz)_2(HTNO)_2]$	<1	>360	90	100-500	32.7	191	1.810	n.d.	n.d.	n.d.	n.d.	dec.	def.	n.d.	[100]
$[Cu(2AzETz)_2(HTNO)_2]$	<1	80	160	100-500	32.7	141	1.663	n.d.	n.d.	n.d.	n.d.	def.	def.	-	[358]
$[Cu(11DTE)(HTNO)_2]\cdot H_2O$	>40	192	200	100-500	32.3	252	n.d.	n.d.	n.d.	n.d.	n.d.	dec.	def.	n.d.	[100]
$[Cu(11DTP)(HTNO)_2]$	<1	240	200	100-500	25.7	184	1.768	n.d.	n.d.	n.d.	n.d.	dec.	def.	n.d.	[100]
$[Zn(MeTz)_2(HTNO)_2]$	2	168	33	100-500	26.1	229	1.832	n.d.	n.d.	n.d.	n.d.	dec.	def.	n.d.	[100]
CsHTNO	<1	>360	50	100-500	10.8	233	2.421	n.d.	n.d.	n.d.	n.d.	det.	def.	n.d.	[100]
PbTNO	<1	<0.1	0.54	100-500	9.1	240	n.d.	n.d.	n.d.	n.d.	n.d.	det.	det.	n.d.	[100]
$[Cu(1M5ATz)_2(HTNR)_2]$	2	>360	n.d.	100-500	29.9	249	n.d.	n.d.	n.d.	n.d.	n.d.	dec.	def.	n.d.	[100]

Table S6. cont.

Compound	*IS* [J]	*FS* [N]	*ESD* [mJ]	Grain size [µm]	N content [%]	T_{dec} [°C]	*d* [g cm^{-3}]	*HOF* [kJ mol^{-1}]	ΔU_{ex} [kJ kg^{-1}]	*DV* [m s^{-1}]	p_{ex} [kbar]	HN	HP	Laser test	Ref.
[Cu(15DATz)(TNR)]	<1	45	160	100-500	41.5	229	n.d.	n.d.	n.d.	n.d.	n.d.	dec.	def.	n.d.	[100]
[Cu(11DTE)(TNR)]·H_2O	<1	360	200	100-500	31.4	264	n.d.	n.d.	n.d.	n.d.	n.d.	def.	def.	n.d.	[100]
[Cu(PA)$_2$(1ATz)$_2$]	4	120	50	500-1000	32.5	178	1.952	n.d.	n.d.	n.d.	n.d.	def.	def.	dec.	[66]
[Cu(HTNR)$_2$(1ATz)$_2$]	1.5	48	16	500-1000	31.1	186	1.905	n.d.	n.d.	n.d.	n.d.	def.	def.	det.	[66]
[Cu(H$_2$TNPG)$_2$(1ATz)$_4$]	3.5	20	90	100-500	39.4	193	1.928	n.d.	n.d.	n.d.	n.d.	def.	def.	def.	[66]
[Cu(PA)$_2$(2ATz)$_2$]	3	128	60	100-500	32.5	203	1.905	n.d.	n.d.	n.d.	n.d.	def.	def.	dec.	[66]
[Cu(HTNR)$_2$(2ATz)$_2$]	3	48	20	100-500	31.1	206	1.864	n.d.	n.d.	n.d.	n.d.	def.	def.	def.	[66]
[Cu(H$_2$TNPG)$_2$(2ATz)$_4$]	2	24	100	100-500	39.4	176	1.909	n.d.	n.d.	n.d.	n.d.	def.	def.	det.	[66]
[Cu$_2$(PA)$_4$(1A5MTz)$_4$]·H_2O	2	168	88	100-500	30.8	190	1.786	n.d.	n.d.	n.d.	n.d.	def.	def.	dec.	[66]
[Cu(TNR)(1A5MTz)$_2$]	2	16	6.3	500-1000	36.1	212	1.887	n.d.	n.d.	n.d.	n.d.	def.	def.	dec.	[66]
[Cu(HTNPG)(1A5MTz)$_2$]	1	7	4.9	100-500	35.0	202	1.891	n.d.	n.d.	n.d.	n.d.	det.	def.	dec.	[66]
[Cu(PA)$_2$(2A5MeTz)$_2$]	2.5	108	37	100-500	31.2	176	1.860	n.d.	n.d.	n.d.	n.d.	def.	def.	dec.	[66]
[Cu(TNR)(2A5MeTz)(H_2O)]·H_2O	2.5	38	840	100-500	25.3	172	1.949	n.d.	n.d.	n.d.	n.d.	def.	def.	dec.	[66]
[Cu(H$_2$TNPG)$_2$(2A5MeTz)$_2$]	2	20	8.2	500-1000	28.7	202	1.939	n.d.	n.d.	n.d.	n.d.	def.	def.	dec.	[66]
[Cu(PA)$_2$(1AzMTz)$_2$]	<1	360	>1500	n.d.	36.4	164	1.83	n.d.	n.d.	n.d.	n.d.	dec.	def.	n.d.	[20]
[Cu(HTNR)$_2$(1AzMTz)$_2$]	<1	192	>1500	n.d.	34.9	154	n.d.	n.d.	n.d.	n.d.	n.d.	dec.	dec.	n.d.	[20]
[Cu(HTNPG)$_2$(1AzMTz)$_2$]	<1	45	1220	n.d.	41.6	151	n.d.	n.d.	n.d.	n.d.	n.d.	def.	def.	n.d.	[20]
[Cu(PA)$_2$(1AzETz)$_2$]	3	252	226	>1000	35.1	183	1.815	n.d.	n.d.	n.d.	n.d.	def.	def.	dec.	[70]
[Cu(PA)$_2$(2AzETz)$_2$]	2	192	160	100-500	35.1	169	1.783	n.d.	n.d.	n.d.	n.d.	dec.	def.	def.	[358]
[Cu(TNR)(1AzETz)$_2$]	<1	240	123	100-500	40.7	177	1.803	n.d.	n.d.	n.d.	n.d.	def.	def.	dec.	[70]
[Cu(TNR)(2AzETz)$_2$]	<1	30	200	100-500	40.7	151	1.843	n.d.	n.d.	n.d.	n.d.	det.	def.	det.	[358]
[Cu(H$_2$TNPG)$_2$(1AzETz)$_4$]	1.5	84	608	500-1000	41.8	121	1.766	n.d.	n.d.	n.d.	n.d.	def.	def.	dec.	[70]
[Cu(H$_2$TNPG)$_2$(2AzETz)$_4$]	<1	54	250	100-500	41.8	145	1.803	n.d.	n.d.	n.d.	n.d.	def.	def.	det.	[358]
[Cu(11DTP)(TNR)]·H_2O	3	>360	700	<100	51.5	153	1.711	n.d.	n.d.	n.d.	n.d.	n.d.	n.d.	def.	[88]
[Co(H_2O)$_4$(1MTz)$_2$](PA)$_2$·2H_2O	>40	>360	1000	100-500	24.8	248	1.786	n.d.	n.d.	n.d.	n.d.	n.d.	n.d.	n.d.	[71]
[Ni(H_2O)$_4$(1MTz)$_2$](PA)$_2$	>40	>360	1500	100-500	34.7	211	1.757	n.d.	n.d.	n.d.	n.d.	n.d.	n.d.	n.d.	[71]
[Cu(PA)$_2$(1MTz)$_2$]	4	>360	300	500-1000	28.5	226	1.828	n.d.	n.d.	n.d.	n.d.	n.d.	n.d.	n.d.	[71]
[Zn(PA)$_2$(1MTz)$_2$]	15	>360	250	100-500	35.9	212	1.724	n.d.	n.d.	n.d.	n.d.	n.d.	n.d.	n.d.	[71]
[Mn(HTNR)$_2$(1MTz)$_4$]	15	288	200	100-500	35.0	202	1.732	n.d.	n.d.	n.d.	n.d.	n.d.	n.d.	n.d.	[71]
[Co(H_2O)$_4$(1MTz)$_2$](HTNR)$_2$·2H_2O	25	>360	230	100-500	23.8	200	1.793	n.d.	n.d.	n.d.	n.d.	n.d.	n.d.	n.d.	[71]
[Ni(H_2O)$_4$(1MTz)$_2$](HTNR)$_2$·2H_2O	25	>360	150	<100	23.8	227	1.803	n.d.	n.d.	n.d.	n.d.	n.d.	n.d.	n.d.	[71]
[Cu(HTNR)$_2$(1MTz)$_2$]	2	160	1500	100-500	27.2	231	1.812	n.d.	n.d.	n.d.	n.d.	n.d.	n.d.	n.d.	[71]
[Zn(HTNR)$_2$(1MTz)$_2$]	4	240	800	500-1000	27.2	214	1.913	n.d.	n.d.	n.d.	n.d.	n.d.	n.d.	n.d.	[71]
K(H$_2$TNPG)·H_2O	*1.1*	+	n.d.	n.d.	13.3	189	n.d.	n.d.	n.d.	n.d.	n.d.	n.d.	n.d.	n.d.	[97]
K$_2$(HTNPG)·H_2O	*1.8*	+	n.d.	n.d.	11.8	260	n.d.	n.d.	n.d.	n.d.	n.d.	n.d.	n.d.	n.d.	[97]
K$_3$(TNPG)·2H_2O	*2.7*	+	n.d.	n.d.	10.2	295	n.d.	n.d.	n.d.	n.d.	n.d.	n.d.	n.d.	n.d.	[97]

Table S6. cont.

Compound	*IS* [J]	*FS* [N]	*ESD* [mJ]	Grain size [µm]	N content [%]	T_{dec} [°C]	*d* [g cm^{-3}]	*HOF* [kJ mol^{-1}]	ΔU_{ex} [kJ kg^{-1}]	*DV* [m s^{-1}]	p_{ex} [kbar]	HN	HP	Laser test	Ref.
$Na(H_2TNPG)\cdot 2H_2O$	*1*	+	n.d.	n.d.	13.2	208	n.d.	n.d.	n.d.	n.d.	n.d.	n.d.	n.d.	n.d.	[98]
CsHTNR	*0.7*	+	n.d.	n.d.	10.7	207	n.d.	n.d.	n.d.	n.d.	n.d.	n.d.	n.d.	n.d.	[99]
$[Cu(15DATz)_2(PA)_2]$	4.25	-	n.d.	n.d.	35.0	216	1.81	-37.5	-10160	n.d.	n.d.	n.d.	n.d.	n.d.	[72]
$[Cu(15DATz)_2(HTNR)_2]$	1.5	-	n.d.	n.d.	33.5	246	1.89	-1673	-7548	n.d.	n.d.	n.d.	n.d.	n.d.	[72]
$[Cu(1NOETz)_2(PA)_2]$	20	>360	480	<100	26.8	197	1.852	n.d.	n.d.	n.d.	n.d.	dec.	def.	ign.	[89]
$[Cu(1NOETz)_2(TNR)]$	2	96	480	<100	29.1	195	n.d.	n.d.	n.d.	n.d.	n.d.	def.	def.	ign.	[89]
$[Cu(1NOETz)_4(HTNR)_2]$	5	96	>1500	500-1000	30.7	167	1.805	n.d.	n.d.	n.d.	n.d.	def.	def.	dec.	[89]
$[Cu(1NOETz)_4(H_2TNPG)_2]$	2	96	1080	500-1000	29.9	108	1.850	n.d.	n.d.	n.d.	n.d.	dec.	def.	ign.	[89]
$[Cu(2NOETz)_2(PA)_2]$	3	192	1080	500-1000	26.8	165	1.890	n.d.	n.d.	n.d.	n.d.	def.	def.	dec.	[89]
$[Cu(2NOETz)_2(TNR)]$	≤1	80	750	<100	29.1	161	1.935	n.d.	n.d.	n.d.	n.d.	def.	def.	ign.	[89]
$[Cu(2NOETz)_2(HTNPG)]$	4	324	750	500-1000	28.4	112	1.970	n.d.	n.d.	n.d.	n.d.	def.	def.	ign.	[89]
$[Cu(1NOETz)_4(H_2TNPG)_2]$	4	324	750	500-1000	29.9	105	n.d.	n.d.	n.d.	n.d.	n.d.	dec.	def.	dec.	[89]
$[Cu(1NOETz)_4(H_2TNPG)_2]\cdot 2(2NOETz)$	4	60	750	100-500	32.8	105	1.804	n.d.	n.d.	n.d.	n.d.	def.	def.	dec.	[89]
$[Mn(HAzTr)_4(H_2O)_2](PA)_2\cdot 4H_2O$	*1.7*	-	n.d.	n.d.	39.6	202	1.685	14974.9	11054	n.d.	n.d.	n.d.	n.d.	n.d.	[74]
$[Co(HAzTr)_2(H_2O)_4](PA)_2$	*2.9*	+	n.d.	n.d.	31.2	208	1.758	10844.7	7132	n.d.	n.d.	n.d.	n.d.	n.d.	[74]
RbPA	*50*	-	n.d.	n.d.	13.4	333	2.337	n.d.	n.d.	n.d.	n.d.	n.d.	n.d.	n.d.	[83]
$Rb_2TNR\cdot H_2O$	*39*	-	n.d.	n.d.	9.7	245	2.512	n.d.	n.d.	n.d.	n.d.	n.d.	n.d.	n.d.	[83]
RbHTNR	*36*	-	n.d.	n.d.	12.7	240	2.302	n.d.	n.d.	n.d.	n.d.	n.d.	n.d.	n.d.	[83]
$[Cd_2(TNR)_2(SCZ)_4]$	>40	>360	>24.8	n.d.	24.9	266	2.78	n.d.	n.d.	n.d.	n.d.	n.d.	n.d.	n.d.	[92]
$[Cd(SCZ)_3(H_2O)](HTNR)_2(H_2O)_2$	25	>360	n.d.	n.d.	23.9	212	1.886	n.d.	n.d.	n.d.	n.d.	n.d.	n.d.	n.d.	[93]
$[Co(SCZ)_2(H_2O)_2](TNR)(H_2O)_2$	15	>360	n.d.	n.d.	24.1	200	1.921	n.d.	n.d.	n.d.	n.d.	n.d.	n.d.	n.d.	[90]
$[Ni(SCZ)_2(H_2O)_2](TNR)(H_2O)_2$	>40	>360	n.d.	n.d.	24.1	232	1.875	n.d.	n.d.	n.d.	n.d.	n.d.	n.d.	n.d.	[90]
$[Zn(SCZ)_2(H_2O)_2](TNR)(H_2O)_2$	>40	>360	n.d.	n.d.	23.8	231	1.891	n.d.	n.d.	n.d.	n.d.	n.d.	n.d.	n.d.	[90]
$[Co(NH_3)_4(NO_2)_2](PA)\cdot H_2O$	8	>216	n.d.	n.d.	27.1	285	1.869	n.d.	n.d.	n.d.	n.d.	n.d.	n.d.	n.d.	[46]
$[Co(NH_3)_5(N_3)](PA)_2$	7.5	>216	n.d.	n.d.	30.5	195	1.844	n.d.	n.d.	n.d.	n.d.	n.d.	n.d.	n.d.	[46]
$[Cu(HTNR)_2(1TzAN)_2]$	3	240	63	100-500	29.1	205	1.873	n.d.	n.d.	n.d.	n.d.	def.	dec.	dec.	[73]
$[Cu(\mu\text{-}1TzAN)(TNR)]$	2	160	63	<100	27.0	143	n.d.	n.d.	n.d.	n.d.	n.d.	dec.	dec.	dec.	[73]
$[Cu(PA)_2(1TzAN)_2]$	2	>360	106	100-500	30.4	170	1.822	n.d.	n.d.	n.d.	n.d.	ign.	dec.	dec.	[73]
$[Cu(HTNO)_2(1TzAN)_2]$	2	360	63	100-500	28.1	181	1.871	n.d.	n.d.	n.d.	n.d.	dec.	dec.	dec.	[73]
$[Cu(HTNR)_2(2TzAN)_2]$	3	160	250	100-500	29.1	192	1.828	n.d.	n.d.	n.d.	n.d.	ign.	dec.	dec.	[73]
$[Cu(2TzAN)_2(TNR)]\cdot 0.5H_2O$	2	>360	250	100-500	34.1	170	1.779	n.d.	n.d.	n.d.	n.d.	def.	dec.	dec.	[73]
$[Cu(PA)_2(2TzAN)_2]$	3	>360	480	100-500	30.4	194	n.d.	n.d.	n.d.	n.d.	n.d.	ign.	ign.	dec.	[73]
$[Cu(HTNO)_2(2TzAN)_2]$	2	288	480	100-500	28.1	174	1.829	n.d.	n.d.	n.d.	n.d.	ign.	dec.	dec.	[73]
$[Cu(H_2TNPG)_2(1PryTz)_4]\cdot 0.5H_2O$	<1	80	160	100-500	30.3	145	1.709	n.d.	n.d.	n.d.	n.d.	dec.	def.	dec.	[125]
$[Cu(HTNR)_2(1PryTz)_2]$	<1	96	160	100-500	25.5	172	1.823	n.d.	n.d.	n.d.	n.d.	dec.	def.	dec.	[125]
$[Cu(TNR)(1PryTz)_2]$	<1	40	250	100-500	29.5	150	n.d.	n.d.	n.d.	n.d.	n.d.	det.	det.	-	[125]

Table S6. cont.

Compound	*IS* [J]	*FS* [N]	*ESD* [mJ]	Grain size [µm]	N content [%]	T_{dec} [°C]	*d* [g cm^{-3}]	*HOF* [kJ mol^{-1}]	ΔU_{ex} [kJ kg^{-1}]	*DV* [m s^{-1}]	p_{ex} [kbar]	HN	HP	Laser test	Ref.
[Cu(PA)$_2$(1PryTz)$_2$]	2	160	250	100-500	26.7	157	1.810	n.d.	n.d.	n.d.	n.d.	dec.	dec.	-	[125]
[Cu(HTNO)$_2$(1PryTz)$_2$]	<1	120	90	100-500	24.6	177	1.765	n.d.	n.d.	n.d.	n.d.	def.	def.	dec.	[125]
[Cu(H$_2$TNPG)$_2$(2PryTz)$_4$]	<1	40	250	100-500	30.3	123	1.742	n.d.	n.d.	n.d.	n.d.	dec.	def.	det.	[125]
[Cu(TNR)(2PryTz)$_2$]	<1	48	200	100-500	29.5	150	1.744	n.d.	n.d.	n.d.	n.d.	det.	def.	dec.	[125]
[Cu(PA)$_2$(2PryTz)$_2$]	2	120	160	100-500	26.7	142	1.764	n.d.	n.d.	n.d.	n.d.	dec.	def.	-	[125]
[Cu(HTNO)$_2$(2PryTz)$_2$]	<1	72	200	100-500	24.6	164	1.779	n.d.	n.d.	n.d.	n.d.	dec.	def.	-	[125]
[Cu(H$_2$TNPG)$_2$(1NOMTz)$_4$]	<1	72	160	100-500	31.3	136	1.931	n.d.	n.d.	7918	n.d.	def.	def.	def.	[387]
[Cu(HTNR)$_2$(1NOMTz)$_2$]	<1	40	140	100-500	26.6	176	1.938	n.d.	n.d.	7715	n.d.	def.	def.	def.	[387]
[Cu(PA)$_2$(1NOMTz)$_2$]	<1	120	90	100-500	27.7	181	1.956	n.d.	n.d.	7767	n.d.	def.	def.	-	[387]
[Cu(HTNO)$_2$(1NOMTz)$_2$]	<1	84	901	100-500	25.8	161	1.928	n.d.	n.d.	7507	n.d.	def.	def.	dec.	[387]
**FUROXANE & FURAZANE**															
KNFOTz	17	220	n.d.	n.d.	41.3	164	2.044	307.7	n.d.	8590	326	n.d.	n.d.	n.d.	[175]
K$_2$BTFOF	9	216	200	n.d.	45.8	218	1.96	1000	5523	7295	204	n.d.	n.d.	n.d.	[176]
K$_2$DNM	2	20	n.d.	n.d.	31.1	229	2.039	110.1	n.d.	7953<br>8138	321	n.d.	n.d.	n.d.	[170]<br>[169]
K$_2$FBOTF	35	>360	1500	<100	42.4	277	1.926	n.d.	n.d.	n.d.	n.d.	n.d.	n.d.	n.d.	[173]
K$_2$BOTFOX	10	48	n.d.	<100	36.2	265	2.156	n.d.	n.d.	n.d.	n.d.	n.d.	n.d.	n.d.	[173]
Ag$_2$BOTFOX	3	16	n.d.	<100	28.8	221	n.d.	n.d.	n.d.	n.d.	n.d.	n.d.	n.d.	n.d.	[173]
KBNFP	0.009	0.3	n.d.	n.d.	26.4	209	2.11	n.d.	n.d.	n.d.	n.d.	n.d.	n.d.	n.d.	[162]
KDNPF	0.025	14	n.d.	n.d.	20.0	284	1.94	n.d.	n.d.	n.d.	n.d.	n.d.	n.d.	n.d.	[133]
KBDF	4	120	n.d.	n.d.	26.7	278	2.086	-464.5	n.d.	7971	270	n.d.	n.d.	n.d.	[168]
K$_2$BDFO	2	5	n.d.	n.d.	22.7	219	2.123<br>2.170	-421.0	n.d.	7759	273	n.d.	n.d.	n.d.	[167, 178]
KDNBF	*2.2*	<5	<6	n.d.	21.1	~200	n.d.	n.d.	n.d.	n.d.	n.d.	n.d.	n.d.	n.d.	[139]
	7	3.8	26.5	n.d.		222	n.d.	n.d.	n.d.	n.d.	n.d.	n.d.	n.d.	n.d.	[140]
NaDNBF	*6.7*	5.4	24	n.d.	22.5	163	n.d.	n.d.	n.d.	n.d.	n.d.	n.d.	n.d.	n.d.	[140]
RbDNBF	*7*	1	28.9	n.d.	18.0	188	n.d.	n.d.	n.d.	n.d.	n.d.	n.d.	n.d.	n.d.	[140]
CsDNBF	*6*	0.2	24.5	n.d.	15.6	165	n.d.	n.d.	n.d.	n.d.	n.d.	n.d.	n.d.	n.d.	[140]
	0.7	+	+	n.d.		212	n.d.	n.d.	n.d.	n.d.	n.d.	n.d.	n.d.	n.d.	[147]
KDNNAF	2	72	156	n.d.	27.1	281	2.174	-347.9	7758	272	n.d.	n.d.	n.d.	n.d.	[172]
KBNAF	1	120	156	n.d.	31.6	291	2.187	-265.1	7507	251	n.d.	n.d.	n.d.	n.d.	[172]
KNATzF	1	168	196	n.d.	40.9	311	1.949	32.6	6358	160	n.d.	n.d.	n.d.	n.d.	[172]
KNAFDODA	1	12	30	n.d.	33.5	200	2.100	179.5	8150	301	n.d.	n.d.	n.d.	n.d.	[172]
NaDADNBF	*15*	*36*	n.d.	n.d.	30.1	242	n.d.	n.d.	n.d.	n.d.	n.d.	n.d.	n.d.	n.d.	[149]
KDADNBF	*13*	*32.4*	n.d.	n.d.	28.5	269	n.d.	n.d.	n.d.	n.d.	n.d.	n.d.	n.d.	n.d.	[149]
RbDADNBF	*10*	*28.8*	n.d.	n.d.	24.6	285	n.d.	n.d.	n.d.	n.d.	n.d.	n.d.	n.d.	n.d.	[149]

Table S6. cont.

Compound	*IS* [J]	*FS* [N]	*ESD* [mJ]	Grain size [μm]	N content [%]	T_{dec} [°C]	*d* [g cm^{-3}]	*HOF* [kJ mol^{-1}]	ΔU_{ex} [kJ kg^{-1}]	*DV* [m s^{-1}]	p_{ex} [kbar]	HN	HP	Laser test	Ref.
CsDADNBF	*8.4*	*16*	n.d.	n.d.	21.6	280	n.d.	n.d.	n.d.	n.d.	n.d.	n.d.	n.d.	n.d.	[149]
$[Ag_2(DMAF)\cdot(H_2O)_2]_n$	10	160	n.d.	n.d.	22.5	230	2.545	n.d.	8159	9673	500	n.d.	n.d.	n.d.	[181]
$[Ag_2(DMAF)]_n$	8	120	n.d.	n.d.	23.8	212	2.796	n.d.	9163	10242	583	n.d.	n.d.	n.d.	[181]
$Cr(DNBF)_3$	*9*	*360*	n.d.	n.d.	23.0	270	1.40	n.d.	n.d.	n.d.	n.d.	n.d.	n.d.	n.d.	[150]
$Fe(DNBF)_3$	*8.5*	*324*	n.d.	n.d.	22.8	275	1.45	n.d.	n.d.	n.d.	n.d.	n.d.	n.d.	n.d.	[150]
$Cu(DNBF)_2$	*7.8*	*360*	n.d.	n.d.	21.7	225	1.47	n.d.	n.d.	n.d.	n.d.	n.d.	n.d.	n.d.	[150]
$K_2BTOFOF\cdot H_2O$	3	120	n.d.	n.d.	40.4	236	1.856	293.7	n.d.	7063	203	n.d.	n.d.	n.d.	[177]
Na_2DBMF	4	136	130	n.d.	32.1	130	1.91	n.d.	5816	8212	310	n.d.	n.d.	n.d.	[183]
K_2DBMF	2	110	100	n.d.	30.2	133	2.07	n.d.	8661	8227	325	n.d.	n.d.	n.d.	[183]
Rb_2DBMF	2	62	60	n.d.	25.9	122	2.23	n.d.	9330	7993	319	n.d.	n.d.	n.d.	[183]
Cs_2DBMF	1	30	90	n.d.	22.6	174	2.40	n.d.	10042	7582	298	n.d.	n.d.	n.d.	[183]
K_2BDFOF	1-2	≤1	n.d.	n.d.	26.8	219	2.09	1145	n.d.	8431	329	n.d.	n.d.	n.d.	[171]
PbBTF	25	>360	n.d.	n.d.	20.4	258	3.38	n.d.	6694	9210	509	n.d.	n.d.	n.d.	[180]
BaBTF	30	>360	n.d.	n.d.	22.2	262	2.33	n.d.	6318	8450	365	n.d.	n.d.	n.d.	[180]
$[Co(AFCA)_2(H_2O)_4]\cdot 2.5H_2O$	32	>360	n.d.	n.d.	19.5	201	1.918	n.d.	5606	8340	320	n.d.	n.d.	n.d.	[182]
$[Ni(AFCA)_2(H_2O)_4]\cdot 2.5H_2O$	30	>360	n.d.	n.d.	19.4	204	1.964	n.d.	5021	8380	328	n.d.	n.d.	n.d.	[182]
$[Fe(AFCA)_2(H_2O)_4]\cdot 2H_2O$	35	>360	n.d.	n.d.	20.0	227	1.847	n.d.	5188	7630	263	n.d.	n.d.	n.d.	[182]
$[Cu(AFCA)_2(H_2O)_4]\cdot 2H_2O$	235	>360	n.d.	n.d.	19.6	198	1.882	n.d.	4561	7740	273	n.d.	n.d.	n.d.	[182]
$[Mn(AFCA)_2(H_2O)_4]\cdot 2H_2O$	24	>360	n.d.	n.d.	20.0	273	1.799	n.d.	5607	8280	314	n.d.	n.d.	n.d.	[182]
$Li_2DNABF\cdot 2H_2O$	5	240	n.d.	500-1000	34.6	317	1.869	n.d.	n.d.	n.d.	n.d.	n.d.	n.d.	n.d.	[184]
$Na_2DNABF\cdot 6H_2O$	>40	>360	n.d.	100-500	33.1	336	1.825	n.d.	n.d.	n.d.	n.d.	n.d.	n.d.	n.d.	[184]
$K_2DNABF\cdot 2H_2O$	3	120	n.d.	100-500	30.3	315	2.048	n.d.	n.d.	n.d.	n.d.	n.d.	n.d.	n.d.	[184]
$CaDNABF\cdot 3H_2O$	6.5	360	n.d.	100-500	32.0	250	2.059	n.d.	n.d.	n.d.	n.d.	n.d.	n.d.	n.d.	[184]
$SrDNABF\cdot 3H_2O$	30	360	n.d.	100-500	27.0	248	2.255	n.d.	n.d.	n.d.	n.d.	n.d.	n.d.	n.d.	[184]
$[Cu(DNABF)(H_2O)_4]\cdot H_2O$	5	192	n.d.	100-500	28.6	148	2.052	n.d.	n.d.	n.d.	n.d.	n.d.	n.d.	n.d.	[184]
$Li_2DNAAF\cdot 5H_2O$	40	192	n.d.	100-500	36.1	104	n.d.	n.d.	n.d.	n.d.	n.d.	n.d.	n.d.	n.d.	[184]
$SrDNAAF\cdot 6H_2O$	7	360	n.d.	100-500	29.2	184	n.d.	n.d.	n.d.	n.d.	n.d.	n.d.	n.d.	n.d.	[184]
$BaDNAAF\cdot 3H_2O$	40	240	n.d.	100-500	30.6	198	n.d.	n.d.	n.d.	n.d.	n.d.	n.d.	n.d.	n.d.	[184]
$[Cu(DNAAF)(H_2O)_4]$	6	240	n.d.	100-500	33.4	200	2.262	n.d.	n.d.	n.d.	n.d.	n.d.	n.d.	n.d.	[184]
$Li_2DNAOAF\cdot 4H_2O$	25	120	n.d.	<100	36.3	74	n.d.	n.d.	n.d.	n.d.	n.d.	n.d.	n.d.	n.d.	[184]
$Na_2DNAOAF\cdot 3H_2O$	40	288	n.d.	<100	35.0	184	n.d.	n.d.	n.d.	n.d.	n.d.	n.d.	n.d.	n.d.	[184]
$SrDNAOAF\cdot 5H_2O$	40	360	n.d.	100-500	29.3	210	n.d.	n.d.	n.d.	n.d.	n.d.	n.d.	n.d.	n.d.	[184]
$BaDNAOAF\cdot 5H_2O$	40	360	n.d.	<100	26.6	200	n.d.	n.d.	n.d.	n.d.	n.d.	n.d.	n.d.	n.d.	[184]
$[Cu(DNAOAF)(H_2O)_4]$	2	144	n.d.	100-500	32.2	105	1.723	n.d.	n.d.	n.d.	n.d.	n.d.	n.d.	n.d.	[184]
K_2BNAFF	3	>72	190	100-500	33.5	245	2.07	689	5483	8263	299	n.d.	n.d.	n.d.	[185]
Ag_2BNAFF	1	60	50	100-500	23.7	82	2.497	n.d.	n.d.	n.d.	n.d.	n.d.	n.d.	n.d.	[185]

Table S6. cont.

Compound	*IS* [J]	*FS* [N]	*ESD* [mJ]	Grain size [μm]	N content [%]	T_{dec} [°C]	*d* [g cm^{-3}]	*HOF* [kJ mol^{-1}]	ΔU_{ex} [kJ kg^{-1}]	*DV* [m s^{-1}]	p_{ex} [kbar]	HN	HP	Laser test	Ref.
$[Cu(AFOTz)_2(HAFOTz)_2]\cdot 2H_2O$	2	160	20	n.d.	45.0	240	2.033	1505	n.d.	n.d.	n.d.	n.d.	n.d.	n.d.	[504]
**NITRATES**															
$[Mn(hz)_3](NO_3)_2$	*0.55*	n.d.	n.d.	n.d.	40.7	141	n.d.	n.d.	n.d.	n.d.	n.d.	n.d.	n.d.	n.d.	[24]
$[Fe(hz)_3](NO_3)_2$	*n.d.*	n.d.	n.d.	n.d.	40.6	140	n.d.	n.d.	n.d.	n.d.	n.d.	n.d.	n.d.	n.d.	[24]
$[Co(hz)_3](NO_3)_2$	*0.4*	n.d.	n.d.	n.d.	40.2	188	n.d.	n.d.	n.d.	n.d.	n.d.	n.d.	n.d.	n.d.	[24]
	11.8	>60	n.d.	n.d.		220	n.d.	n.d.	n.d.	n.d.	n.d.	n.d.	n.d.	n.d.	[344]
$[Ni(hz)_3](NO_3)_2$	*0.45*	n.d.	n.d.	n.d.	40.2	220	n.d.	n.d.	n.d.	n.d.	n.d.	n.d.	n.d.	n.d.	[24]
	0.8	*588.4 kPa*	20	n.d.		167	2.129	n.d.	4243	7000	n.d.	n.d.	n.d.	n.d.	[346]
	10	24	27	n.d.		n.d.	n.d.	n.d.	n.d.	n.d.	n.d.	n.d.	n.d.	n.d.	[342]
	16.8	10	n.d.	n.d.		185	n.d.	n.d.	n.d.	n.d.	n.d.	n.d.	n.d.	n.d.	[344]
	19.2	*10*	<1000	n.d		n.d.	n.d.	n.d.	n.d.	700	n.d.	n.d.	n.d.	n.d.	[347, 348]
	n.d.	n.d.	n.d.	n.d.			2.156	-699.9	~3300	9200	202	n.d.	n.d.	n.d.	[350]
$[Zn(hz)_3](NO_3)_2$	*0.65*	n.d.	n.d.	n.d.	39.3	212	n.d.	n.d.	n.d.	n.d.	n.d.	n.d.	n.d.	n.d.	[24]
	>25	>360	720	n.d.		n.d.	n.d.	n.d.	n.d.	n.d.	n.d.	n.d.	n.d.	n.d.	[342]
$[Cd(hz)_3](NO_3)_2$	*0.43*	n.d.	n.d.	n.d.	33.7	145	n.d.	n.d.	n.d.	n.d.	n.d.	n.d.	n.d.	n.d.	[24]
	>25	192	80	n.d.		n.d.	n.d.	n.d.	n.d.	n.d.	n.d.	n.d.	n.d.	n.d.	[342]
$[Cd(hz)_2](NO_3)_2$	*0.53*	n.d.	n.d.	n.d.	30.0	241	n.d.	n.d.	n.d.	n.d.	n.d.	n.d.	n.d.	n.d.	[24]
$[Co(hz)_3](NO_3)_3$	>25	60	2250	n.d.	36.7	n.d.	n.d.	n.d.	n.d.	n.d.	n.d.	n.d.	n.d.	n.d.	[342]
$[Cr(hz)_3](NO_3)_3$	>25	64	1167	n.d.	37.7	n.d.	n.d.	n.d.	n.d.	n.d.	n.d.	n.d.	n.d.	n.d.	[342]
$[Cr(NH_3)_6](NO_3)_3$	*6.4*	n.d.	n.d.	n.d.	37.1	265	n.d.	n.d.	n.d.	n.d.	n.d.	n.d.	n.d.	n.d.	[352]
$[Cr(H_2NCONH_2)_6](NO_3)_3$	*10*	n.d.	n.d.	n.d.	35.1	265	n.d.	n.d.	n.d.	n.d.	n.d.	n.d.	n.d.	n.d.	[352]
$[Co(NH_3)_6](NO_3)_3$	12.8	n.d.	n.d.	n.d.	41.4	n.d.	n.d.	n.d.	n.d.	n.d.	n.d.	n.d.	n.d.	n.d.	[351]
	10					360	n.d.	n.d.	n.d.	n.d.	n.d.	n.d.	n.d.	n.d.	[352]
$[Co(NH_3)_5(NO_3)](NO_3)_2$	*9.6*	n.d.	n.d.	n.d.	34.0	310	n.d.	n.d.	n.d.	n.d.	n.d.	n.d.	n.d.	n.d.	[352]
$[Cu(NH_3)_4](NO_3)_2$	*3.8*	n.d.	n.d.	n.d.	32.9	330	n.d.	n.d.	n.d.	n.d.	n.d.	n.d.	n.d.	n.d.	[352]
	11.4	214	n.d.	n.d.		271	1.91	-828	n.d.	3500	n.d.	n.d.	n.d.	n.d.	[353]
$[Co(en)_3](NO_3)_3$	>36	n.d.	n.d.	n.d.	29.6	n.d.	n.d.	n.d.	n.d.	n.d.	n.d.	n.d.	n.d.	n.d.	[351]
	12.2	n.d.	n.d.	n.d.		-	n.d.	n.d.	n.d.	n.d.	n.d.	n.d.	n.d.	n.d.	[352]
$[Co(NH_3)_5Cl](NO_3)_2$	27	n.d.	n.d.	n.d.		n.d.	n.d.	n.d.	n.d.	n.d.	n.d.	n.d.	n.d.	n.d.	[351]
$[Co(en)_2Cl_2](NO_3)$	>36	n.d.	n.d.	n.d.		n.d.	n.d.	n.d.	n.d.	n.d.	n.d.	n.d.	n.d.	n.d.	[351]
$[Ag_7(5MTz)_4(NO_3)_3]_n$	5	60	100	n.d.	20.9	n.d.	n.d.	n.d.	n.d.	n.d.	n.d.	n.d.	n.d.	n.d.	[354]
$[Ag_3(HTz)_2(NO_3)_3]_n$	2	10	40	n.d.	24.1	n.d.	n.d.	n.d.	n.d.	n.d.	n.d.	n.d.	n.d.	n.d.	[354]
$[Ag_7(ATz)_4(NO_3)_3]_n$	4	20	60	n.d.	25.2	n.d.	n.d.	n.d.	n.d.	n.d.	n.d.	n.d.	n.d.	n.d.	[354]
$[Ag_5(5NTz)_4NO_3]_n$	1	5	10	n.d.	31.6	n.d.	n.d.	n.d.	n.d.	n.d.	n.d.	n.d.	n.d.	n.d.	[354]

Table S6. cont.

Compound	*IS* [J]	*FS* [N]	*ESD* [mJ]	Grain size [µm]	N content [%]	T_{dec} [°C]	*d* [g cm^{-3}]	*HOF* [kJ mol^{-1}]	ΔU_{ex} [kJ kg^{-1}]	*DV* [m s^{-1}]	p_{ex} [kbar]	HN	HP	Laser test	Ref.
$[Cu(NO_3)_2(H_2O)(AzEtTz)_2]$	10	108	840	100-500	46.3	152	1.834	n.d.	n.d.	n.d.	n.d.	def.	def.	def.	[357]
$[Cu(1AzMTz)_6](NO_3)_2$	2	15	>1500	n.d.	65.7	123	1.66	n.d.	n.d.	n.d.	n.d.	def.	def.	n.d.	[20]
$CuNa(5NTzMTz)_2NO_3$	<6	<120	n.d.	n.d.	49.2	246	n.d.	n.d.	n.d.	n.d.	n.d.	n.d.	n.d.	n.d.	[271]
$CuNa(5NTzMTz)_2NO_3{\cdot}H_2O$	<8	<140	n.d.	n.d.	47.6	248	n.d.	n.d.	n.d.	n.d.	n.d.	n.d.	n.d.	n.d.	[271]
$[Zn(TATOT)_3(H_2O)](NO_3)_2{\cdot}2H_2O$	>40	>360	1500	500-1000	51.6	234	1.825	n.d.	n.d.	n.d.	n.d.	n.d.	n.d.	n.d.	[363]
$[Ag(11DTM)_2]NO_3$	4	144	450	100-500	50.2	136	2.06	n.d.	n.d.	n.d.	n.d.	def.	dec.	n.d.	[67]
$[Ag(12DTM)_3]NO_3$	2	108	540	100-500	55.9	124	1.834	n.d.	n.d.	n.d.	n.d.	def.	def.	n.d.	[67]
$[Ag(NO_3)(22DTM)]$	3	36	380	100-500	39.2	186	2.342	n.d.	n.d.	n.d.	n.d.	def.	def.	n.d.	[67]
$[Cu_2(NO_3)_4(22DTM)_3]$	1.5	30	>1500	100-500	47.2	198	1.945	n.d.	n.d.	n.d.	n.d.	def.	def.	n.d.	[67]
$[Cu(NO_3)_2(H_255DTE)_2]$	4	160	n.d.	<100	48.5	180	1.948	n.d.	n.d.	n.d.	n.d.	dec.	n.d.	n.d.	[359]
$[Co(ANQ)_2(H_2O)_2](NO_3)_2$	9	80	700	500-1000	36.8	139	1.993	n.d.	n.d.	n.d.	n.d.	n.d.	n.d.	n.d.	[368]
$[Ni(ANQ)_2(H_2O)_2](NO_3)_2$	4	120	80	500-1000	36.8	186	2.001	n.d.	n.d.	n.d.	n.d.	n.d.	n.d.	n.d.	[368]
$[Cu(ANQ)_2(NO_3)_2]$	<1	<5	500	100-500	39.5	77	2.171	n.d.	n.d.	n.d.	n.d.	n.d.	n.d.	n.d.	[368]
$[Zn(ANQ)_2(H_2O)_2](NO_3)_2$	5	120	500	100-500	36.3	181	1.992	n.d.	n.d.	n.d.	n.d.	n.d.	n.d.	n.d.	[368]
$[Ag(ANQ)_2]NO_3$	<1	<5	10	<100	37.8	142	2.235	n.d.	n.d.	n.d.	n.d.	n.d.	n.d.	n.d.	[368]
$[Co(H_255DTM)_2(H_2O)_2](NO_3)_2$	4	240	200	<100	48.2	223	1.918	n.d.	n.d.	n.d.	n.d.	n.d.	n.d.	n.d.	[356]
$[Ni(H_255DTM)_2(H_2O)_2](NO_3)_2$	1	>360	1500	100-500	48.2	142	n.d.	n.d.	n.d.	n.d.	n.d.	n.d.	n.d.	n.d.	[356]
$[Zn(H_255DTM)_2(H_2O)_2](NO_3)_2$	6	252	1500	100-500	47.6	158	1.928	n.d.	n.d.	n.d.	n.d.	n.d.	n.d.	n.d.	[356]
$[Cu(H_255DTM)_2(H_2O)_2](NO_3)_2$	6	>360	260	100-500	44.7	144	1.895	n.d.	n.d.	n.d.	n.d.	n.d.	n.d.	n.d.	[356]
$[Cu(H_255DTM)_2(NO_3)_2]{\cdot}2H_2O$	2	160	1500	100-500	47.8	141	1.957	n.d.	n.d.	n.d.	n.d.	n.d.	def.	n.d.	[356]
$[Cu(H_255DTM)_2(NO_3)_2]$	2	160	250	<100	49.7	148	n.d.	n.d.	n.d.	n.d.	n.d.	n.d.	def.	n.d.	[356]
$[Mn_3(DATr)_6(H_2O)_6](NO_3)_6{\cdot}2H_2O$	-	-	n.d.	n.d.	39.5	298	n.d.	n.d.	1279	n.d.	n.d.	n.d.	n.d.	n.d.	[364]
$[Co_3(DATr)_6(H_2O)_6](NO_3)_6{\cdot}2H_2O$	*2*	+	n.d.	n.d.	39.1	350	1.849	n.d.	1624	n.d.	n.d.	n.d.	n.d.	n.d.	[364]
$[Ni_3(DATr)_6(H_2O)_6](NO_3)_6{\cdot}2H_2O$	*4*	-	n.d.	n.d.	39.2	341	1.869	n.d.	1532	n.d.	n.d.	n.d.	n.d.	n.d.	[364]
$[Zn_3(DATr)_6(H_2O)_6](NO_3)_6{\cdot}2H_2O$	-	-	n.d.	n.d.	39.5	255	1.870	n.d.	836	n.d.	n.d.	n.d.	n.d.	n.d.	[364]
$[Cu(H5MHTz)_2(NO_3)_2]_n$	2	32	90	<100	47.2	206	n.d.	n.d.	n.d.	n.d.	n.d.	det.	det.	det.	[243]
$[Cu(H5MHTz)_2(H_2O)_2](NO_3)_2$	2	96	150	<100	43.4	163	1.944	n.d.	n.d.	n.d.	n.d.	def.	def.	det.	[243]
$[Cu(\mu_3\text{-}5MHTz)_2(H_2O)(NO_3)]_n$	3	108	800	<100	38.2	163	1.903	n.d.	n.d.	n.d.	n.d.	dec.	def.	-	[243]
$[Ag_25ATz(NO_3)]$	15	18	+	n.d.	23.4	298	n.d.	n.d.	n.d.	n.d.	n.d.	n.d.	det.	n.d.	[355]
$[Ag(1M5ATz)]NO_3$	10	100-360	-	n.d.	31.4	226	n.d.	n.d.	n.d.	n.d.	n.d.	n.d.	def.	n.d.	[355]
$[Ag(2M5ATz)]NO_3$	20	100-360	-	n.d.	31.4	259	n.d.	n.d.	n.d.	n.d.	n.d.	n.d.	def.	n.d.	[355]
$[Cu(TrDA)_3(NO_3)_2]_n$	22.5	-	2475	n.d.	53.4	243	1.68	n.d.	15194	9160	357	n.d.	n.d.	n.d.	[365]
$[Ag(TrDA)_{1.5}(NO_3)]_n$	30	-	>2475	n.d.	43.8	257	2.16	n.d.	5802	7773	297	n.d.	n.d.	n.d.	[365]
$[AgNO_3(1MTz)_2]$	40	160	150	<100	37.3	119	2.113	n.d.	n.d.	n.d.	n.d.	n.d.	n.d.	n.d.	[71]

Table S6. cont.

Compound	*IS* [J]	*FS* [N]	*ESD* [mJ]	Grain size [µm]	N content [%]	T_{dec} [°C]	*d* [g cm^{-3}]	*HOF* [kJ mol^{-1}]	ΔU_{ex} [kJ kg^{-1}]	*DV* [m s^{-1}]	p_{ex} [kbar]	HN	HP	Laser test	Ref.
[Cu(1cBTz)(NO$_3$)$_2$]	9	>360	90	500-1000	35.0	160	1.585	n.d.	n.d.	n.d.	n.d.	n.d.	n.d.	n.d.	[19]
[Zn(1ATz)$_6$](NO$_3$)$_2$	<1	6	10	100-500	64.1	180	1.800	n.d.	n.d.	n.d.	n.d.	def.	def.	n.d.	[361]
[Cu(1ATz)$_3$(NO$_3$)$_2$]	3	2.25	620	<100	53.8	161	1.968	n.d.	n.d.	n.d.	n.d.	def.	def.	det.	[361]
[Cu(µ-2ATz)$_2$(NO$_3$)$_2$]	<1	2	10	<100	47.0	157	2.169	n.d.	n.d.	n.d.	n.d.	def.	def.	det.	[361]
[Cu(H$_2$O)(55DT11P)$_2$](NO$_3$)$_2$	8	360	1500	100-500	44.6	293	1.808	n.d.	n.d.	n.d.	n.d.	n.d.	n.d.	n.d.	[360]
[Co(55DT11P)$_3$](NO$_3$)$_3$·3H$_2$O	10	>360	1500	100-500	45.1	267	n.d.	n.d.	n.d.	n.d.	n.d.	n.d.	n.d.	n.d.	[360]
[Ni(55DT11P)$_3$](NO$_3$)$_2$·2.5H$_2$O	5	324	1500	500-1000	47.4	203	n.d.	n.d.	n.d.	n.d.	n.d.	n.d.	n.d.	n.d.	[360]
[Ni(H$_2$O)$_2$(55DT11P)$_2$](NO$_3$)$_2$·H$_2$O	10	360	150	100-500	42.2	163	1.719	n.d.	n.d.	n.d.	n.d.	n.d.	n.d.	n.d.	[360]
[Zn(H$_2$O)$_2$(55DT11P)$_2$](NO$_3$)$_2$·2H$_2$O	40	>360	200	100-500	41.8	170	1.736	n.d.	n.d.	n.d.	n.d.	n.d.	n.d.	n.d.	[360]
[Cu(NO$_3$)$_2$(1AzPTz)$_2$(H$_2$O)]	>40	96	422	100-500	43.8	155	1.713	n.d.	n.d.	n.d.	n.d.	def.	def.	dec.	[70]
[Cu(APTz)$_2$(NO$_3$)$_2$]	>78.4	>360	62.5	n.d.	43.6	145	n.d.	n.d.	n.d.	n.d.	n.d.	n.d.	n.d.	+	[370]
[Cu(DNAzPTz)$_2$(NO$_3$)$_2$]	9.2	304	62.5	n.d.	36.2	204	1.74	n.d.	n.d.	n.d.	n.d.	n.d.	n.d.	-	[370]
[Cu(ADMPTz)$_2$(NO$_3$)$_2$]	15.8	>360	62.5	n.d.	33.3	228	1.83	n.d.	n.d.	n.d.	n.d.	n.d.	n.d.	-	[370]
[Cu(DNAzDMPTz)$_2$(NO$_3$)$_2$]	6.8	317.5	62.5	n.d.	30.3	140	1.90	n.d.	n.d.	n.d.	n.d.	n.d.	n.d.	+	[370]
[Cu(ADMPTz)$_2$(NO$_3$)]NO$_3$	-	-	-	n.d.	33.3	145	1.49	n.d.	n.d.	n.d.	n.d.	n.d.	n.d.	+	[370]
[Cu(DNAzDMPTz)$_2$(NO$_3$)]NO$_3$	511.6	>360	125	n.d.	30.3	127	1.66	n.d.	n.d.	n.d.	n.d.	n.d.	n.d.	-	[370]
[Cu(H$_2$DTzIm)(NO$_3$)]$_n$	>40	>360	n.d.	n.d.	46.9	256	2.227	n.d.	6464	9543	446	n.d.	n.d.	n.d.	[371]
[K(H$_2$DABCO)](NO$_3$)$_3$	29	>360	n.d.	n.d.	20.6	406	1.68	-339.1	5430	7566	234	n.d.	n.d.	n.d.	[376]
[Cu(PATz)$_2$](NO$_3$)$_2$	4	30	n.d.	n.d.	32.3	n.d.	n.d.	n.d.	n.d.	n.d.	n.d.	n.d.	n.d.	n.d.	[362]
[Hg(PATz)$_2$](NO$_3$)$_2$	20	80	n.d.	n.d.	27.5	n.d.	n.d.	n.d.	n.d.	n.d.	n.d.	n.d.	n.d.	n.d.	[362]
[Co(PATz)$_3$](NO$_3$)$_3$	4	160	n.d.	n.d.	33.4	n.d.	n.d.	n.d.	n.d.	n.d.	n.d.	n.d.	n.d.	n.d.	[362]
[Ni(PATz)$_3$](NO$_3$)$_2$	2	48	n.d.	n.d.	34.0	n.d.	n.d.	n.d.	n.d.	n.d.	n.d.	n.d.	n.d.	n.d.	[362]
[Zn(PATz)$_3$](NO$_3$)$_2$	3	128	n.d.	n.d.	33.8	n.d.	n.d.	n.d.	n.d.	n.d.	n.d.	n.d.	n.d.	n.d.	[362]
[Cd(PATz)$_3$](NO$_3$)$_2$	7.5	128	n.d.	n.d.	32.4	n.d.	n.d.	n.d.	n.d.	n.d.	n.d.	n.d.	n.d.	n.d.	[362]
[Cr(PATz)$_3$](NO$_3$)$_3$	4	180	n.d.	n.d.	33.6	n.d.	n.d.	n.d.	n.d.	n.d.	n.d.	n.d.	n.d.	n.d.	[362]
[Co(BIU)$_2$(H$_2$O)$_2$](NO$_3$)$_2$	>40	>360	n.d.	n.d.	26.3	243	1.870	n.d.	n.d.	7122	203	n.d.	n.d.	n.d.	[369]
[Ni(BIU)$_2$(H$_2$O)$_2$](NO$_3$)$_2$	>40	>360	n.d.	n.d.	26.4	323	1.861	n.d.	n.d.	7235	224	n.d.	n.d.	n.d.	[369]
[Cu(BIU)$_2$ (NO$_3$)$_2$]	35	288	n.d.	n.d.	28.5	242	1.997	n.d.	n.d.	6996	205	n.d.	n.d.	n.d.	[369]
Ag(BIU)NO$_3$	40	324	n.d.	n.d.	20.5	190	2.516	n.d.	n.d.	7352	285	n.d.	n.d.	n.d.	[369]
[Cu(NO$_3$)$_2$(µ-1A123Tr)$_2$]	<1	30	100	100-500	39.3	167	2.165	n.d.	n.d.	n.d.	n.d.	def.	def.	def.	[68]
[Zn(1A123Tr)$_4$](NO$_3$)$_2$	6	45	700	100-500	48.0	188	1.768	n.d.	n.d.	n.d.	n.d.	def.	def.	-	[68]
[Cu(1NOETz)$_3$(NO$_3$)$_2$]	5	48	1080	500-1000	35.8	121	1.888	n.d.	n.d.	n.d.	n.d.	def.	def.	def.	[89]
[Ag(DAF)NO$_3$]	16.2	353	n.d.	n.d.	25.9	215	2.694		2954	6950	283	n.d.	n.d.	n.d.	[374]
[Zn(DAF)(H$_2$O)$_4$](NO$_3$)$_2$	n.d.	360	n.d.	n.d.	n.d.	235	1.901	872.5	8890	8489	353	n.d.	n.d.	n.d.	[375]
[Cu(DAF)$_2$(H$_2$O)$_2$(NO$_3$)$_2$]	n.d.	240	n.d.	n.d.	n.d.	120	1.990	2329.3	10231	9405	466	n.d.	n.d.	n.d.	[375]
[Cu(1TzAN)$_6$](NO$_3$)$_2$	>40	>360	106	100-500	53.2	117	1.581	n.d.	n.d.	n.d.	n.d.	dec.	dec.	dec.	[73]

Table S6. cont.

Compound	*IS* [J]	*FS* [N]	*ESD* [mJ]	Grain size [μm]	N content [%]	T_{dec} [°C]	*d* [g cm^{-3}]	*HOF* [kJ mol^{-1}]	ΔU_{ex} [kJ kg^{-1}]	*DV* [m s^{-1}]	p_{ex} [kbar]	HN	HP	Laser test	Ref.
$[Cu(NO_3)_2(1NMTz)_2]$	<1	80	n.d.	n.d.	37.7	155	1.97	n.d.	n.d.	8082	n.d.	det.	def.	dec.	[386]
$[Cu(1NOMTz)_6](NO_3)_2$	<1	5	90	>1000	42.4	121	1.806	n.d.	n.d.	7865	n.d.	def.	def.	det.	[387]
**CHLORATES, PERCHLORATES**															
$[Co(hz)_3](ClO_4)_3$	>25	28	840	n.d.	18.5	n.d.	n.d.	n.d.	n.d.	n.d.	n.d.	n.d.	n.d.	n.d.	[342]
$[Co(hz)_5(ClO_4)_2]$	5	n.d.	det.	n.d.	33.5	1.948	194	-349.8	~5000	7500-9000	251-336	n.d.	n.d.	n.d.	[350]
$[Ni(hz)_5(ClO_4)_2]$	extremely senstitive			n.d.	33.5	1.983	n.d.	-367.1	~5200	9200	368	n.d.	n.d.	n.d.	[350]
$[Ni(hz)_3](ClO_4)_2$	extremely senstitive			n.d.	23.8	n.d.	n.d.	n.d.	n.d.	n.d.	n.d.	n.d.	n.d.	n.d.	[342]
$[Zn(hz)_3](ClO_4)_2$	>25	192	9000	n.d.	23.3	n.d.	n.d.	n.d.	n.d.	n.d.	n.d.	n.d.	n.d.	n.d.	[342]
$[Cd(hz)_3](ClO_4)_2$	6	2	10	n.d.	20.6	n.d.	n.d.	n.d.	n.d.	n.d.	n.d.	n.d.	n.d.	n.d.	[342]
$[Cr(hz)_3](ClO_4)_3$	2	96	1750	n.d.	18.8	n.d.	n.d.	n.d.	n.d.	n.d.	n.d.	n.d.	n.d.	n.d.	[342]
$[Co_2(hz)_4(hzc)_2](ClO_4)_2\cdot H_2O$	7.5	n.d.	det.	n.d.	28.3	231	2.000	n.d.	~3300	n.d.	n.d.	n.d.	n.d.	n.d.	[382]
$[Zn_2(hz)_3(hzc)_2](ClO_4)_2\cdot H_2O$	25	n.d.	-	n.d.	23.6	293	2.117	n.d.	~3500	n.d.	n.d.	n.d.	n.d.	n.d.	[382]
$[Co(NH_3)_6](ClO_4)_3$	*3.8*	n.d.	n.d.	n.d.	18.3	n.d.	n.d.	n.d.	n.d.	n.d.	n.d.	n.d.	n.d.	n.d.	[351]
	3.6	n.d.	n.d.	n.d.		360	n.d.	n.d.	n.d.	n.d.	n.d.	n.d.	n.d.	n.d.	[352]
$[Co(NH_3)_5(H_2O)](ClO_4)_3$	*4.2*	n.d.	n.d.	n.d.	15.2	n.d.	n.d.	n.d.	n.d.	n.d.	n.d.	n.d.	n.d.	n.d.	[351]
$[Co(NH_3)_4(H_2O)_2](ClO_4)_3$	*4.8*	n.d.	n.d.	n.d.	12.1	n.d.	n.d.	n.d.	n.d.	n.d.	n.d.	n.d.	n.d.	n.d.	[351]
$[Co(NH_3)_5Cl](ClO_4)_2$	*6.6*	n.d.	n.d.	n.d.	18.5	n.d.	n.d.	n.d.	n.d.	n.d.	n.d.	n.d.	n.d.	n.d.	[351]
	4.2	n.d.	n.d.	n.d.		320	n.d.	n.d.	n.d.	n.d.	n.d.	n.d.	n.d.	n.d.	[352]
trans- $[Co(NH_3)_4Cl_2]ClO_4$	*4*	n.d.	n.d.	n.d.	16.8	n.d.	n.d.	n.d.	n.d.	n.d.	n.d.	n.d.	n.d.	n.d.	[351]
$[Co(NH_3)_5(SCN)](ClO_4)_2$	*11*	n.d.	n.d.	n.d.	21.0	325	n.d.	n.d.	n.d.	n.d.	n.d.	n.d.	n.d.	n.d.	[352]
$[Co(NH_3)_4(SCN)_2](ClO_4)$	*7*	n.d.	n.d.	n.d.	24.5	335	n.d.	n.d.	n.d.	n.d.	n.d.	n.d.	n.d.	n.d.	[352]
$[Co(en)_3](ClO_4)_3$	*3.4*	n.d.	n.d.	n.d.	15.6	n.d.	n.d.	n.d.	n.d.	n.d.	n.d.	n.d.	n.d.	n.d.	[351]
trans-$[Co(en)_2Cl_2]ClO_4$	*36*	n.d.	n.d.	n.d.	17.2	n.d.	n.d.	n.d.	n.d.	n.d.	n.d.	n.d.	n.d.	n.d.	[351]
	17	n.d.	n.d.	n.d.		300	n.d.	n.d.	n.d.	n.d.	n.d.	n.d.	n.d.	n.d.	[352]
$[Co(NH_3)_6](ClO_3)_3$	*2.8*	n.d.	n.d.	n.d.	20.4	n.d.	n.d.	n.d.	n.d.	n.d.	n.d.	n.d.	n.d.	n.d.	[351]
$[Co(en)_3](ClO_3)_3$	*4.8*	n.d.	n.d.	n.d.	20.4	n.d.	n.d.	n.d.	n.d.	n.d.	n.d.	n.d.	n.d.	n.d.	[351]
trans-$[Co(en)_2Cl_2]ClO_3$	*3.2*	n.d.	n.d.	n.d.	18.1	n.d.	n.d.	n.d.	n.d.	n.d.	n.d.	n.d.	n.d.	n.d.	[351]
	-	n.d.	n.d.	n.d.		320	n.d.	n.d.	n.d.	n.d.	n.d.	n.d.	n.d.	n.d.	[352]
$[Co(H_2NCH_2CH_2NCH_2CH_2NH_2)_2](ClO_3)_3$	*4.2*	n.d.	n.d.	n.d.	16.4	325	n.d.	n.d.	n.d.	n.d.	n.d.	n.d.	n.d.	n.d.	[352]
$[Cu(NH_3)_4](ClO_4)_2$	2.6	93	n.d.	n.d.	17.0	n.d.	n.d.	n.d.	4322	3228	n.d.	n.d.	n.d.	n.d.	[410]
$[Cu(NH_3)_4](ClO_3)_2$	3	n.d.	n.d.	n.d.	18.8	n.d.	n.d.	n.d.	n.d.	4300	n.d.	n.d.	n.d.	n.d.	[15]
$[Mn(AzMeTz)_6](ClO_4)_2$	<1	0.5	740	n.d.	58.9	137	n.d.	n.d.	n.d.	n.d.	n.d.	det.	def.	n.d.	[20]
$[Fe(AzMeTz)_6](ClO_4)_2$	<1	<0.1	380	n.d.	58.5	127	n.d.	n.d.	n.d.	n.d.	n.d.	det.	det.	n.d.	[20]
$[Cu(AzMeTz)_6](ClO_4)_2$	<1	<0.1	480	n.d.	58.1	133	n.d.	n.d.	n.d.	n.d.	n.d.	det.	det.	n.d.	[20]

Table S6. cont.

Compound	*IS* [J]	*FS* [N]	*ESD* [mJ]	Grain size [μm]	N content [%]	T_{dec} [°C]	*d* [g cm^{-3}]	*HOF* [kJ mol^{-1}]	ΔU_{ex} [kJ kg^{-1}]	*DV* [m s^{-1}]	p_{ex} [kbar]	HN	HP	Laser test	Ref.
[Zn(AzMeTz)$_6$](ClO$_4$)$_2$	<1	<0.1	380	n.d.	58.0	130	n.d.	n.d.	n.d.	n.d.	n.d.	def.	def.	n.d.	[20]
[Mn(AzMeTz)$_6$](ClO$_3$)$_2$	<1	<0.1	380	n.d.	60.0	123	1.71	n.d.	n.d.	n.d.	n.d.	def.	det.	n.d.	[20]
[Fe(1AzETz)$_6$](ClO$_4$)$_2$	3	3.75	65	100-500	54.0	151	1.648	n.d.	n.d.	n.d.	n.d.	def.	def.	det.	[357]
[Zn(1AzETz)$_6$](ClO$_4$)$_2$	15	40	368	<100	53.5	196	n.d.	n.d.	n.d.	n.d.	n.d.	def.	def.	-	[357]
[Cu(1AzETz)$_6$](ClO$_4$)$_2$	<1	15	368	100-500	53.4	158	n.d.	n.d.	n.d.	n.d.	n.d.	def.	def.	det.	[357]
[Cu(1ETz)$_6$](ClO$_4$)$_2$	10	120	960	>1000	39.5	210	1.545	n.d.	n.d.	n.d.	n.d.	def.	def.	dec.	[357]
[Cu(1AzETz)$_6$](ClO$_3$)$_2$	2.5	4	226	500-1000	55.2	146	n.d.	n.d.	n.d.	n.d.	n.d.	def.	def.	det.	[357]
[Cu(1ETz)$_6$](ClO$_3$)$_2$	7	60	608	>1000	41.0	158	1.415	n.d.	n.d.	n.d.	n.d.	def.	def.	dec.	[357]
[Ag(1AzETz)]ClO$_4$	<1	0.6	65	<100	28.3	165	2.470	n.d.	n.d.	n.d.	n.d.	def.	def.	dec.	[357]
[Ag(μ-2AzETz)(ClO$_4$)]	<1	0.6	13	100-500	28.3	151	2.412	n.d.	n.d.	n.d.	n.d.	det.	det.	n.d.	[358]
[Mn(1AzPTz)$_6$](ClO$_4$)$_2$	4.5	60	368	500-1000	50.2	208	1.534	n.d.	n.d.	n.d.	n.d.	def.	def.	n.d.	[70]
[Fe(1AzPTz)$_6$](ClO$_4$)$_2$	2.5	28	317	100-500	50.2	146	n.d.	n.d.	n.d.	n.d.	n.d.	def.	def.	def.	[70]
[Cu(1AzPTz)$_6$](ClO$_4$)$_2$	2.5	32	368	500-1000	49.8	165	n.d.	n.d.	n.d.	n.d.	n.d.	def.	def.	det.	[70]
[Zn(1AzPTz)$_6$](ClO$_4$)$_2$	9	80	422	500-1000	49.7	183	n.d.	n.d.	n.d.	n.d.	n.d.	def.	def.	-	[70]
[Fe(1PTz)$_6$](ClO$_4$)$_2$	25	192	1220	100-500	36.3	202	n.d.	n.d.	n.d.	n.d.	n.d.	dec.	dec.	n.d.	[70]
[Cu(1PTz)$_6$](ClO$_4$)$_2$	30	240	>1500	>1000	35.9	209	n.d.	n.d.	n.d.	n.d.	n.d.	dec.	dec.	dec.	[70]
[Cu(1AzPTz)$_6$](ClO$_3$)$_2$	2.5	24	>1500	500-1000	51.2	151	n.d.	n.d.	n.d.	n.d.	n.d.	def.	def.	def.	[70]
[Cu(1PTz)$_6$](ClO$_3$)$_2$	>40	168	960	>1000	37.2	152	n.d.	n.d.	n.d.	n.d.	n.d.	dec.	dec.	dec.	[70]
[Ag$_2$5ATz(ClO$_4$)]	2	<5	+	n.d.	17.7	319	n.d.	n.d.	n.d.	n.d.	n.d.	n.d.	det.	n.d.	[355]
[Ag(1M5ATz)]ClO$_4$	<5	120-360	-	n.d.	22.2	252	2.432	n.d.	n.d.	n.d.	n.d.	n.d.	def.	n.d.	[355]
[Ag(2M5ATz)]ClO$_4$	2	<5	+	n.d.	23.0	289	2.414	n.d.	n.d.	n.d.	n.d.	n.d.	def.	n.d.	[355]
[Cu(ClO$_4$)(μ-5MHTz)(H5MHTz)]$_2$	1	5	10	<100	43.1	217	1.873	n.d.	n.d.	n.d.	n.d.	det.	det.	det.	[243]
[Cu(ClO$_4$)(μ-5MHTz)]$_n$	1	5	7	<100	28.6	206	n.d.	n.d.	n.d.	n.d.	n.d.	det.	det.	det.	[243]
[Cu(H$_2$O)$_2$(H$_2$55DTM)](ClO$_4$)$_2$	<1	<5	>100	500-1000	37.2	85	2.038	n.d.	n.d.	n.d.	n.d.	n.d.	n.d.	n.d.	[356]
[Cu(ClO$_4$)$_2$(H$_2$55DTM)]	<1	<5	n.d.	500-1000	41.9	233	2.128	n.d.	n.d.	n.d.	n.d.	n.d.	det.	n.d.	[356]
[Ag(11DTM)$_2$]ClO$_4$	<1	30	1080	100-500	43.8	163	2.190	n.d.	n.d.	n.d.	n.d.	def.	def.	n.d.	[67]
[Ag(12DTM)$_4$]ClO$_4$	<1	50	226	100-500	54.9	140	1.901	n.d.	n.d.	n.d.	n.d.	def.	def.	n.d.	[67]
[Cu(11DTM)$_3$](ClO$_4$)$_2$	<1	0.75	42	<100	46.8	230	1.951	n.d.	n.d.	n.d.	n.d.	def.	def.	n.d.	[67]
[Cu(12DTM)$_3$](ClO$_4$)$_2$	1.5	1	47	<100	46.8	215	1.912	n.d.	n.d.	n.d.	n.d.	det.	det.	n.d.	[67]
[Cu(22DTM)$_3$](ClO$_4$)$_2$	<1	0.75	37	<100	46.8	242	1.913<br>2.003	n.d.	n.d.	n.d.	n.d.	det.	def.	n.d.	[67]
[Ag$_5$(5NTz)$_4$(ClO$_4$)]$_n$	1	2	5	n.d.	25.6	305	n.d.	n.d.	n.d.	n.d.	n.d.	n.d.	n.d.	n.d.	[354]
[Cu(1NMTz)$_6$](ClO$_3$)$_2$·0.5H$_2$O	4	42	n.d.	n.d.	41.4	102	1.77	n.d.	n.d.	n.d.	n.d.	def.	def.	-	[386]
[Cu(1NMTz)$_6$](ClO$_4$)$_2$	<1	30	n.d.	n.d.	40.5	160	1.75	n.d.	n.d.	7793	n.d.	det.	def.	det.	[386]
[Fe(1NMTz)$_6$](ClO$_4$)$_2$	<1	14	n.d.	n.d.	40.8	176	1.80	n.d.	n.d.	8033	n.d.	def.	def.	det.	[386]

Table S6. cont.

Compound	*IS* [J]	*FS* [N]	*ESD* [mJ]	Grain size [μm]	N content [%]	T_{dec} [°C]	*d* [g cm^{-3}]	*HOF* [kJ mol^{-1}]	ΔU_{ex} [kJ kg^{-1}]	*DV* [m s^{-1}]	p_{ex} [kbar]	HN	HP	Laser test	Ref.
$[Ni(1NMTz)_6](ClO_4)_2$	<1	32	n.d.	n.d.	40.7	217	n.d.	n.d.	n.d.	n.d.	n.d.	def.	def.	-	[386]
$[Zn(1NMTz)_6](ClO_4)_2$	<1	28	n.d.	n.d.	40.5	187	1.76	n.d.	n.d.	7824	n.d.	def.	def.	n.d.	[386]
$[Cu(1NOMTz)_6](ClO_3)_2$	<1	0.4	139	>1000	38.2	145	1.822	n.d.	n.d.	n.d.	n.d.	det.	def.	det.	[387]
$[Cu(1NOMTz)_6](ClO_4)_2$	2	0.75	13	>1000	37.1	119	1.848	n.d.	n.d.	7910	n.d.	def.	def.	det.	[387]
$[Cu(H_255DTE)_2(H_2O)](ClO_4)_2$	1	5	n.d.	100-500	36.3	206	1.908	n.d.	n.d.	n.d.	n.d.	det.	n.d.	det.	[359]
$[Cu(H_255DTE)_2(H_2O)_2](ClO_4)_2{\cdot}2H_2O$	3	14	n.d.	500-1000	33.6	n.d.	1.881	n.d.	n.d.	n.d.	n.d.	def.	n.d.	n.d.	[359]
$[Cu(B5MeTzC2)_2(H_2O)_2](ClO_4)_2$	4	80	n.d.	500-1000	32.6	278	1.790	n.d.	n.d.	n.d.	n.d.	def.	n.d.	def.	[359]
$[Co(NH_3)_4(1MTz)_2](ClO_4)_3$	9	160	36	<100	29.3	201	n.d.	n.d.	n.d.	n.d.	n.d.	n.d.	n.d.	def.	[71]
$[Mn(1MTz)_6](ClO_4)_2$	7	120	80	<100	44.3	223	1.582	n.d.	n.d.	n.d.	n.d.	n.d.	n.d.	-	[71]
$[Fe(1MTz)_6](ClO_4)_2$	1	60	100	<100	44.3	213	n.d.	n.d.	n.d.	n.d.	n.d.	n.d.	n.d.	def.	[71]
$[Co(1MTz)_6](ClO_4)_2$	3	60	500	<100	44.1	253	1.614	n.d.	n.d.	n.d.	n.d.	n.d.	n.d.	-	[71]
$[Ni(1MTz)_6](ClO_4)_2$	5	60	100	<100	44.1	271	1.629	n.d.	n.d.	n.d.	n.d.	n.d.	n.d.	def.	[71]
$[Cu(1MTz)_6](ClO_4)_2$	2.5	54	80	<100	43.8	211	1.645	n.d.	n.d.	n.d.	n.d.	n.d.	n.d.	det.	[71]
$[Zn(1MTz)_6](ClO_4)_2$	10	120	250	<100	43.8	221	1.635	n.d.	n.d.	n.d.	n.d.	n.d.	n.d.	n.d.	[71]
$[Mn(1MTz)_6](ClO_4)_2{\cdot}2(1MTz)$	7	216	460	100-500	48.4	216	1.590	n.d.	n.d.	n.d.	n.d.	n.d.	n.d.	n.d.	[71]
$[Fe(1MTz)_6](ClO_4)_2{\cdot}2(1MTz)$	10	120	400	100-500	48.4	215	1.592	n.d.	n.d.	n.d.	n.d.	n.d.	n.d.	def.	[71]
$[Co(1MTz)_6](ClO_4)_2{\cdot}2(1MTz)$	10	80	130	500-1000	48.2	210	1.612	n.d.	n.d.	n.d.	n.d.	n.d.	n.d.	n.d.	[71]
$[Ni(1MTz)_6](ClO_4)_2{\cdot}2(1MTz)$	6	192	200	<100	48.2	207	1.621	n.d.	n.d.	n.d.	n.d.	n.d.	n.d.	def.	[71]
$[Cu(1MTz)_6](ClO_4)_2{\cdot}2(1MTz)$	7	72	200	500-1000	47.9	206	1.623	n.d.	n.d.	n.d.	n.d.	n.d.	n.d.	def.	[71]
$[Zn(1MTz)_6](ClO_4)_2{\cdot}2(1MTz)$	10	144	600	100-500	47.8	212	1.630	n.d.	n.d.	n.d.	n.d.	n.d.	n.d.	dec.	[71]
$[Mn(1ATz)_6](ClO_4)_2$	<1	0.5	11	100-500	55.0	195	1.736	n.d.	n.d.	n.d.	n.d.	def.	det.	n.d.	[361]
$[Fe(1ATz)_6](ClO_4)_2$	<1	<0.1	12	100-500	54.9	187	1.766	n.d.	n.d.	n.d.	n.d.	det.	det.	det.	[361]
$[Cu(1ATz)_6](ClO_4)_2$	<1	<0.1	5	<100	54.4	174	1.803	n.d.	n.d.	n.d.	n.d.	det.	det.	det.	[361]
$[Zn(1ATz)_6](ClO_4)_2$	<1	0.5	12	100-500	54.2	189	1.783	n.d.	n.d.	n.d.	n.d.	det	def.	n.d.	[361]
$[Fe(2ATz)_6](ClO_4)_2{\cdot}2(2ATz)$	<1	0.1	25	100-500	59.9	169	1.784	n.d.	n.d.	n.d.	n.d.	def.	det.	det.	[361]
$[Zn(2ATz)_6](ClO_4)_2{\cdot}2(2ATz)$	2	0.2	30	100-500	59.3	192	1.812	n.d.	n.d.	n.d.	n.d.	det.	det.	n.d.	[361]
$[Cu(2ATz)_6](ClO_4)_2{\cdot}2(2ATz)$	1	<0.2	1.2	100-500	59.4	157	1.815	n.d.	n.d.	n.d.	n.d.	det.	det.	det.	[361]
$[Fe(2ATz)_5(H_2O)](ClO_4)_2$	1.5	0.15	10	100-500	50.2	159	1.855	n.d.	n.d.	n.d.	n.d.	det.	det.	n.d.	[361]
$[Zn(2ATz)_5(H_2O)](ClO_4)_2$	1.5	0.5	10	<100	49.5	200	1.884	n.d.	n.d.	n.d.	n.d.	det.	det.	n.d.	[361]
$[Cu(1ATz)_4(H_2O)_2](ClO_4)_2$	1	0.4	10	100-500	43.8	173	1.947	n.d.	n.d.	n.d.	n.d.	det.	det.	n.d.	[361]
$[Cu(H_2O)(55DT11P)_2](ClO_4)_2{\cdot}H_2O$	3	10	65	100-500	34.0	251	1.848	n.d.	n.d.	n.d.	n.d.	n.d.	n.d.	det.	[360]
$[Co(55DT11P)_3](ClO_4)_3{\cdot}6H_2O$	12.5	72	500	500-1000	33.4	206	n.d.	n.d.	n.d.	n.d.	n.d.	n.d.	n.d.	n.d.	[360]
$[Ni(H_2O)_2(55DT11P)_2](ClO_4)_2{\cdot}3H_2O$	20	216	750	500-1000	31.7	190	n.d.	n.d.	n.d.	n.d.	n.d.	n.d.	n.d.	n.d.	[360]
$[Zn(H_2O)(55DT11P)_2](ClO_4)_2$	2	20	200	<100	34.9	256	1.807	n.d.	n.d.	n.d.	n.d.	n.d.	n.d.	n.d.	[360]
$[Fe(55DT11P)_3](ClO_4)_3{\cdot}6H_2O$	<1	15	100	100-500	33.5	176	n.d.	n.d.	n.d.	n.d.	n.d.	n.d.	n.d.	def.	[360]
$[Cu(1cPTz)_6](ClO_3)_2$	2	32	100	500-1000	37.7	156	1.527	n.d.	n.d.	n.d.	n.d.	dec.	def.	n.d.	[19]

Table S6. cont.

Compound	*IS* [J]	*FS* [N]	*ESD* [mJ]	Grain size [μm]	N content [%]	T_{dec} [°C]	*d* [g cm^{-3}]	*HOF* [kJ mol^{-1}]	ΔU_{ex} [kJ kg^{-1}]	*DV* [m s^{-1}]	p_{ex} [kbar]	HN	HP	Laser test	Ref.
$[Cu(1cBTz)_4](ClO_3)_2$	5	36	200	100-500	30.8	153	1.582	n.d.	n.d.	n.d.	n.d.	dec.	def.	n.d.	[19]
$[Mn(1cPTz)_6](ClO_4)_2$	5	216	90	>1000	36.8	206	1.503	n.d.	n.d.	n.d.	n.d.	def.	def.	n.d.	[19]
$[Fe(1cPTz)_6](ClO_4)_2$	3	54	90	>1000	36.7	192	1.566<br>1.485	n.d.	n.d.	n.d.	n.d.	def.	def.	n.d.	[19]
$[Cu(1cPTz)_6](ClO_4)_2$	4	80	480	500-1000	36.4	183	1.543	n.d.	n.d.	n.d.	n.d.	dec.	def.	n.d.	[19]
$[Zn(1cPTz)_6](ClO_4)_2$	6	144	250	500-1000	36.3	201	1.547	n.d.	n.d.	n.d.	n.d.	def.	def.	n.d.	[19]
$[Cu(1cBTz)_4(H_2O)](ClO_3)_2$	8	84	250	>1000	28.8	191	1.576	n.d.	n.d.	n.d.	n.d.	dec.	def.	n.d.	[19]
$[Cu(1cPeTz)_4(H_2O)](ClO_3)_2$	4	160	250	>1000	26.9	207	1.558	n.d.	n.d.	n.d.	n.d.	dec.	def.	n.d.	[19]
$[Ni(5NTz)_2(NH_3)_4]ClO_4$	*6*	*30*	n.d.	n.d.	43.2	278	n.d.	n.d.	n.d.	n.d.	n.d.	n.d.	n.d.	n.d.	[384]
$[Co(5NTz)_2(NH_3)_4]ClO_4$	*6.4*	*30*	n.d.	n.d.	43.1	268	n.d.	n.d.	n.d.	n.d.	n.d.	n.d.	n.d.	n.d.	[384]
	n.d.	n.d.	n.d.	n.d.	n.d.	269	2.05	n.d.	n.d.	8100	n.d.	n.d.	n.d.	n.d.	[385]
$[Cu(5NTz)(NH_3)_3]ClO_4$	*6*	*48*	n.d.	n.d.	48.3	260	n.d.	n.d.	n.d.	n.d.	n.d.	n.d.	n.d.	n.d.	[384]
$[Zn(5NTz)(NH_3)_3]ClO_4$	*7.2*	*140*	n.d.	n.d.	47.9	120	n.d.	n.d.	n.d.	n.d.	n.d.	n.d.	n.d.	n.d.	[384]
$[Mn(15DMTz)_6](ClO_4)_2$	10	>360	n.d.	n.d.	39.9	307	n.d.	n.d.	n.d.	n.d.	n.d.	def.	def.	n.d.	[69]
$[Cu(15DMTz)_4(H_2O)](ClO_4)_2$	3	128	n.d.	n.d.	33.3	226	n.d.	n.d.	n.d.	n.d.	n.d.	n.d.	n.d.	n.d.	[69]
$[Zn(15DMTz)_4](ClO_4)_2$	2	180	n.d.	n.d.	34.1	321	n.d.	n.d.	n.d.	n.d.	n.d.	def.	def.	n.d.	[69]
$[Zn(TATOT)_4](ClO_4)_2{\cdot}2H_2O$	35	>360	1500	<100	48.9	274	1.829	n.d.	n.d.	n.d.	n.d.	n.d.	n.d.	n.d.	[363]
$[Cu(H_2O)_2(2M5ATz)_4](ClO_4)_2{\cdot}H_2O$	5	30	260	500-1000	39.3	176	1.669	n.d.	n.d.	n.d.	n.d.	n.d.	n.d.	def.	[388]
$[Cu(H_2O)_2(2M5ATz)_4](ClO_4)_2$	<1	24	20	<100	40.3	171	1.788	n.d.	n.d.	n.d.	n.d.	n.d.	n.d.	dec.	[388]
$[Cu(H_2O)_2(2M5ATz)_2(ClO_4)_2]$	2	2.2	150	<100	28.2	192	1.997	n.d.	n.d.	n.d.	n.d.	n.d.	n.d.	dec.	[388]
$[Cu(H_2O)_2(2MTz)_4](ClO_4)_2$	5	5	50	100-500	35.3	240	1.702	n.d.	n.d.	n.d.	n.d.	n.d.	n.d.	dec.	[388]
$[Co(ANQ)_2(H_2O)_2](ClO_4)_2$	3	10	30	500-1000	26.3	176	2.068	n.d.	n.d.	n.d.	n.d.	n.d.	n.d.	det.	[368]
$[Ni(ANQ)_2(H_2O)_2](ClO_4)_2$	3	10	40	500-1000	26.3	230	2.159	n.d.	n.d.	n.d.	n.d.	n.d.	n.d.	-	[368]
$[Cu(ANQ)_2(ClO_4)_2]{\cdot}2H_2O$	<1	16	500	100-500	26.1	134	2.128	n.d.	n.d.	n.d.	n.d.	n.d.	n.d.	det.	[368]
$[Zn(ANQ)_2(H_2O)_2](ClO_4)_2$	3	28	300	100-500	26.0	198	2.193	n.d.	n.d.	n.d.	n.d.	n.d.	n.d.	-	[368]
$[Ag(ANQ)_2]ClO_4$	<1	<5	10	100-500	31.4	148	2.316	n.d.	n.d.	n.d.	n.d.	n.d.	n.d.	det.	[368]
$[Cu(AG)_2](ClO_4)_2$	4	20	450	n.d.	27.3	215	n.d.	n.d.	n.d.	n.d.	n.d.	n.d.	n.d.	n.d.	[563]
$[Co(H_24ATTz)_2(H_2O)_2](ClO_4)_3{\cdot}4H_2O$	4	60	500	500-1000	27.96	176	2.022	n.d.	n.d.	n.d.	n.d.	n.d.	n.d.	n.d.	[289]
$[Cu(1FMeTz)_6](ClO_4)_2$	<1	24	181	100-500	38.4	184	1.991	n.d.	n.d.	n.d.	n.d.	n.d.	n.d.	n.d.	[403]
$[Cu(1FMeTz)_6](ClO_3)_2$	<1	24	224	<100	39.9	87	1.752	n.d.	n.d.	n.d.	n.d.	n.d.	n.d.	n.d.	[403]
$[Co(NH_3)_5(4ATr)](ClO_4)_3$	+	n.d.	n.d.	n.d.	23.8	n.d.	2.08	310	n.d.	7540	n.d.	n.d.	n.d.	n.d.	[404]
$[Co(NH_3)_5(1M5ATz)](ClO_4)_3$	+	n.d.	n.d.	n.d.	25.9	n.d.	1.95	270	n.d.	7320	n.d.	n.d.	n.d.	n.d.	[404]
$[Co(NH_3)_5(15ATz)](ClO_4)_3$	+	n.d.	n.d.	n.d.	28.4	n.d.	2.03	250	n.d.	7680	n.d.	n.d.	n.d.	n.d.	[404]
$[Fe(APTt)_3](ClO_4)_2$	4.2	78	62.5	n.d.	39.2	208	1.633	n.d.	n.d.	n.d.	n.d.	n.d.	n.d.	-	[406]
$[Fe(DMPTrTt)_3](ClO_4)_2$	8.5	183	62.5	n.d.	41.0	180	n.d.	n.d.	n.d.	n.d.	n.d.	n.d.	n.d.	+	[406]
$[Fe(ADMPTrTt)_3](ClO_4)_2$	12.4	195	62.5	n.d.	43.8	178	n.d.	n.d.	n.d.	n.d.	n.d.	n.d.	n.d.	+	[406]

Table S6. cont.

Compound	*IS* [J]	*FS* [N]	*ESD* [mJ]	Grain size [µm]	N content [%]	T_{dec} [°C]	*d* [g cm^{-3}]	*HOF* [kJ mol^{-1}]	ΔU_{ex} [kJ kg^{-1}]	*DV* [m s^{-1}]	p_{ex} [kbar]	HN	HP	Laser test	Ref.
$[Fe(ANPTt)_2(CH_3CN)_2](ClO_4)_2$	23.4	69	62.5	n.d.	33.5	237	1.85	n.d.	n.d.	n.d.	n.d.	n.d.	n.d.	-	[407]
$[Fe(ANPTrTt)_2(H_3O)_2](ClO_4)_2$	16.4	195	<25	n.d.	35.6	244	n.d.	n.d.	n.d.	n.d.	n.d.	n.d.	n.d.	+	[407]
$[Cu(ClO_3)_2(1A123Tr)_4]$	1	5	20	<100	39.5	119	1.916	n.d.	n.d.	n.d.	n.d.	n.d.	n.d.	det.	[391]
$[Cu_2(4ATr)_6](ClO_3)_4$	1	5	15	<100	34.8	186	1.964	n.d.	n.d.	n.d.	n.d.	n.d.	n.d.	det.	[391]
$[Cu(ClO_3)_2(1MTz)_4]$	3	7	100	100-500	39.5	159	1.807	n.d.	n.d.	n.d.	n.d.	n.d.	n.d.	det.	[391]
$[Cu(ClO_3)(1M5ATz)_4](ClO_3)$	2	16	110	<100	44.7	157	1.832	n.d.	n.d.	n.d.	n.d.	n.d.	n.d.	det.	[391]
$[Cu(ClO_3)_2(H_2O)_2(2M5ATz)_2]$	2	5	50	100-500	30.1	125	2.000	n.d.	n.d.	n.d.	n.d.	n.d.	n.d.	det.	[391]
$[Cu(11DTP)_3](ClO_3)_2$	2	18	100	<100	43.6	168	1.711	n.d.	n.d.	n.d.	n.d.	n.d.	n.d.	det.	[391]
$[Cu(ClO_3)_2(12DTP)_2]$	1	9	100	100-500	37.9	162	1.863	n.d.	n.d.	n.d.	n.d.	n.d.	n.d.	det.	[391]
$[Cu(ClO_3)_2(22DTP)_2]$	1	5	50	100-500	37.9	176	1.821	n.d.	n.d.	n.d.	n.d.	n.d.	n.d.	det.	[391]
$[Cu(H_2O)_2(11BTziP)_2](ClO_3)_2$	1	32	65	100-500	35.8	156	1.764	n.d.	n.d.	n.d.	n.d.	n.d.	n.d.	def.	[391]
$[Cu(ClO_3)_2(11DTB)_2]$	3	32	100	<100	36.2	157	1.840	n.d.	n.d.	n.d.	n.d.	n.d.	n.d.	det.	[391]
$[Mn(11DTP)_3](ClO_4)_2$	3	216	300	<100	42.3	243	1.656	n.d.	n.d.	n.d.	n.d.	n.d.	n.d.	-	[88]
$[Fe(11DTP)_3](ClO_4)_2$	2	72	100	<100	42.3	234	1.754	n.d.	n.d.	n.d.	n.d.	n.d.	n.d.	det.	[88]
$[Co(11DTP)_3](ClO_4)_2$	1	18	80	<100	42.1	255	1.712	n.d.	n.d.	n.d.	n.d.	n.d.	n.d.	det.	[88]
$[Ni(11DTP)_3](ClO_4)_2$	3	120	200	<100	42.1	297	n.d.	n.d.	n.d.	n.d.	n.d.	n.d.	n.d.	dec.	[88]
$[Cu(11DTP)_3](ClO_4)_2$	1	54	330	<100	41.7	231	1.721	n.d.	n.d.	n.d.	n.d.	n.d.	n.d.	det.	[88]
$[Zn(11DTP)_3](ClO_4)_2$	1	288	200	<100	41.7	253	1.706	n.d.	n.d.	n.d.	n.d.	n.d.	n.d.	-	[88]
$[Ag(12DTP)]ClO_4$	8	16	150	<100	28.9	185	2.267	n.d.	n.d.	n.d.	n.d.	n.d.	n.d.	n.d.	[88]
$[Cu(12DTP)_3](ClO_4)_2$	1.5	18	100	<100	41.9	222	1.693	n.d.	n.d.	n.d.	n.d.	n.d.	n.d.	det.	[88]
$[Cu(H_2O)_2(22DTP)_2](ClO_4)_2$	1.5	64	300	<100	34.0	257	1.765	n.d.	n.d.	n.d.	n.d.	n.d.	n.d.	det.	[88]
$[Mn(1A5MTz)_6](ClO_4)_2$	2	9	323	>1000	49.5	199	1.689	n.d.	n.d.	n.d.	n.d.	def.	def.	n.d.	[389]
$[Fe(1A5MTz)_6](ClO_4)_2$	<1	0.45	250	100-500	49.5	177	1.711	n.d.	n.d.	n.d.	n.d.	det.	det.	n.d.	[389]
$[Cu(1A5MTz)_4(H_2O)](ClO_4)_2$	<1	0.75	270	100-500	41.4	163	1.787	n.d.	n.d.	n.d.	n.d.	det.	def.	n.d.	[389]
$[Zn(1A5MTz)_6](ClO_4)_2$	<1	5	630	100-500	48.9	198	1.734	n.d.	n.d.	n.d.	n.d.	def.	def.	n.d.	[389]
$[Cu(1M5ATz)_4(H_2O)_2](ClO_4)_2$	2.7	>360	n.d.	n.d.	40.3	254	1.493	622	n.d.	n.d.	n.d.	n.d.	n.d.	n.d.	[390]
$[Cu(MSC)_2(ClO_4)_2]$	2	24	70	<100	19.1	186	2.055	n.d.	n.d.	n.d.	n.d.	def.	def.	det.	[75]
$[Zn(MSC)_2(H_2O)_2](ClO_4)_2$	>40	160	300	<100	17.6	231	n.d.	n.d.	n.d.	n.d.	n.d.	n.d.	n.d.	def.	[75]
$[Zn(MSC)_3](ClO_4)_2 \cdot H_2O$	25	>360	600	<100	22.9	333	n.d.	n.d.	n.d.	n.d.	n.d.	n.d.	n.d.	def.	[75]
$[Mn(MSC)_2(H_2O)_2](ClO_4)_2$	10	216	1000	<100	17.1	234	n.d.	n.d.	n.d.	n.d.	n.d.	n.d.	n.d.	def.	[75]
$[Ni(MSC)_3(ClO_4)_2]$	10	<60	100	<100	24.0	258	n.d.	n.d.	n.d.	n.d.	n.d.	n.d.	n.d.	det.	[75]
$[Co(CHZ)_3](ClO_4)_2$	*11*	*10*	n.d.	n.d.	31.8	~280	n.d.	n.d.	n.d.	n.d.	n.d.	n.d.	n.d.	n.d.	[401]
	1	≤5	35	<100		243	n.d.	n.d.	n.d.	n.d.	n.d.	n.d.	n.d.	+	[402]
$[Ni(CHZ)_3](ClO_4)_2$	*2.2*	*10*	n.d.	n.d.	31.8	~310	n.d.	n.d.	n.d.	n.d.	n.d.	n.d.	n.d.	n.d.	[401]
	1	≤5	300	<100		273	n.d.	n.d.	n.d.	n.d.	n.d.	n.d.	n.d.	+	[402]

Table S6. cont.

Compound	*IS* [J]	*FS* [N]	*ESD* [mJ]	Grain size [µm]	N content [%]	T_{dec} [°C]	*d* [g cm^{-3}]	*HOF* [kJ mol^{-1}]	ΔU_{ex} [kJ kg^{-1}]	*DV* [m s^{-1}]	p_{ex} [kbar]	HN	HP	Laser test	Ref.
$[Cu(CHZ)_2](ClO_4)_2$	*12*	*10*	n.d.	n.d.	25.3	120	n.d.	n.d.	n.d.	n.d.	n.d.	n.d.	n.d.	n.d.	[401]
	3	<<5	20	<100		186	n.d.	n.d.	n.d.	n.d.	n.d.	n.d.	n.d.	+	[347]
$[Zn(CHZ)_3](ClO_4)_2$	*12*	*10*	n.d.	n.d.	31.4	~285	n.d.	n.d.	n.d.	n.d.	n.d.	n.d.	n.d.	n.d.	[401]
	1.5	20	700	<100		268	n.d.	n.d.	n.d.	n.d.	n.d.	n.d.	n.d.	-	[402]
$[Mg(CHZ)_3](ClO_4)_2$	2.5	60	200	<100	34.1	239	n.d.	n.d.	n.d.	n.d.	n.d.	n.d.	n.d.	-	[402]
$[Mn(CHZ)_3](ClO_4)_2$	2	24	500	<100	32.1	263	n.d.	n.d.	n.d.	n.d.	n.d.	n.d.	n.d.	-	[402]
$[Cd(DATz)_6](ClO_4)_2$	*1.2*	*10*	n.d.	n.d.	55.3	243	1.855	n.d.	n.d.	n.d.	n.d.	n.d.	n.d.	n.d.	[405]
$[Cu(PATz)_2](ClO_4)_2$	25	48	n.d.	n.d.	26.2	n.d.	n.d.	n.d.	n.d.	n.d.	n.d.	n.d.	n.d.	n.d.	[362]
$[Hg(PATz)_2](ClO_4)_2$	>25	28	n.d.	n.d.	22.6	n.d.	n.d.	n.d.	n.d.	n.d.	n.d.	n.d.	n.d.	n.d.	[362]
$[Co(PATz)_3](ClO_4)_3$	15	160	n.d.	n.d.	27.0	n.d.	n.d.	n.d.	n.d.	n.d.	n.d.	n.d.	n.d.	n.d.	[362]
$[Ni(PATz)_3](ClO_4)_2$	7.5	112	n.d.	n.d.	29.3	n.d.	n.d.	n.d.	n.d.	n.d.	n.d.	n.d.	n.d.	n.d.	[362]
$[Zn(PATz)_3](ClO_4)_2$	4	216	n.d.	n.d.	29.2	n.d.	n.d.	n.d.	n.d.	n.d.	n.d.	n.d.	n.d.	n.d.	[362]
$[Cd(PATz)_3](ClO_4)_2$	10	128	n.d.	n.d.	28.0	n.d.	n.d.	n.d.	n.d.	n.d.	n.d.	n.d.	n.d.	n.d.	[362]
$[Cr(PATz)_3](ClO_4)_3$	>25	112	n.d.	n.d.	27.1	n.d.	n.d.	n.d.	n.d.	n.d.	n.d.	n.d.	n.d.	n.d.	[362]
$[Cu(ClO_4)_2(HURZ)_2]$	<1	2	n.d.	100-500	22.7	214	2.37	n.d.	n.d.	n.d.	n.d.	n.d.	n.d.	n.d.	[437]
$[Co(BIU)_2(H_2O)_2](ClO_4)_2{\cdot}BIU$	>40	>360	n.d.	n.d.	21.5	227	1.891	n.d.	n.d.	6867	208	n.d.	n.d.	n.d.	[369]
$[Ni(BIU)_2(H_2O)_2](ClO_4)_2$	>40	>360	n.d.	n.d.	16.8	381	2.047	n.d.	n.d.	8013	297	n.d.	n.d.	n.d.	[369]
$[Cu(BIU)_2(ClO_4)_2]$	25	240	n.d.	n.d.	17.9	328	2.097	n.d.	n.d.	7489	251	n.d.	n.d.	n.d.	[369]
$Ag(BIU)ClO_4$	30	252	n.d.	n.d.	20.3	182	2.191	n.d.	n.d.	6404	202	n.d.	n.d.	n.d.	[369]
$[Cu(4ATr)_3](ClO_4)_2$	1	9.8	+	n.d.	32.7	311	n.d.	801.5	n.d.	6500	n.d.	n.d.	n.d.	n.d.	[392, 393]
$[Cu(H_2O)_2(1A123Tr)_4](ClO_4)_2$	1	0.45	100	500-1000	35.3	167	1.810	n.d.	n.d.	n.d.	n.d.	det.	def.	det.	[68]
$[Mn(1A123Tr)_6](ClO_4)_2$	3	10	60	100-500	44.3	194	1.673	n.d.	n.d.	n.d.	n.d.	def.	def.	-	[68]
$[Fe(1A123Tr)_6](ClO_4)_2$	1.5	2	40	100-500	44.3	166	1.697	n.d.	n.d.	n.d.	n.d.	def.	def.	det.	[68]
$[Zn(1A123Tr)_6](ClO_4)_2$	3	6.75	70	100-500	43.7	188	1.740	n.d.	n.d.	n.d.	n.d.	def.	def.	-	[68]
$[Cu(1A124Tr)_4(ClO_3)_2]$	<1	23	10	100-500	32.5	132	1.888	n.d.	n.d.	n.d.	n.d.	def.	def.	def.	[68]
$[Ag(ATrCA)]ClO_4$	5	72	n.d.	n.d.	24.0	230	2.534	n.d.	2138	6800	n.d.	n.d.	n.d.	n.d.	[394]
$[Cu(1NOETz)_6](ClO_3)_2$	2	15	250	500-1000	35.5	149	1.689	n.d.	n.d.	n.d.	n.d.	def.	def.	dec.	[89]
$[Zn(1NOETz)_6](ClO_3)_2$	7	14	750	500-1000	35.4	154	n.d.	n.d.	n.d.	n.d.	n.d.	dec.	def.	-	[89]
$[Cu(1NOETz)_6](ClO_4)_2$	3	25	480	<100	34.5	165	1.727	n.d.	n.d.	n.d.	n.d.	def.	def.	dec.	[89]
$[Cu(2NOETz)_6](ClO_4)_2$	2	5	1080	100-500	34.5	143	n.d.	n.d.	n.d.	n.d.	n.d.	def.	def.	dec.	[89]
$[Co(NH_3)_4(Tz)_2]ClO_4$	64%/ 12.8 J	n.d.	n.d.	n.d.	46.1	239	1.86	n.d.	n.d.	6900	n.d.	n.d.	n.d.	def.	[395]
$[Co(NH_3)_4(5NTz)_2]ClO_4$	8%/ 12.8 J	n.d.	n.d.	n.d.	43.1	234	1.97	n.d.	n.d.	8100	n.d.	n.d.	n.d.	det.	[395]
$[Co(NH_3)_4(5MTz)_2]ClO_4$	-	n.d.	n.d.	n.d.	46.1	252	1.75	n.d.	n.d.	6800	n.d.	n.d.	n.d.	-	[395]

Table S6. cont.

Compound	*IS* [J]	*FS* [N]	*ESD* [mJ]	Grain size [µm]	N content [%]	T_{dec} [°C]	*d* [g cm^{-3}]	*HOF* [kJ mol^{-1}]	ΔU_{ex} [kJ kg^{-1}]	*DV* [m s^{-1}]	p_{ex} [kbar]	HN	HP	Laser test	Ref.
$[Co(NH_3)_4(5ATz)_2]ClO_4$	14%/ 12.8 J	n.d.	n.d.	n.d.	49.7	242	1.81	n.d.	n.d.	6700	n.d.	n.d.	n.d.	det.	[395]
$[Co(NH_3)_4(5NATz)]ClO_4$	10%/ 12.8 J	n.d.	n.d.	n.d.	39.5	238	1.90	n.d.	n.d.	7100	n.d.	n.d.	n.d.	-	[395]
$[Co(NH_3)_4(15DATz)_2](ClO_4)_3$	32%/ 12.8 J	n.d.	n.d.	n.d.	35.8	233	1.85	n.d.	n.d.	7300	n.d.	n.d.	n.d.	det.	[395]
$[Co(NH_3)_4(15DMTz)_2](ClO_4)_3$	8%/ 12.8 J	n.d.	n.d.	n.d.	31.5	234	1.90	n.d.	n.d.	7500	n.d.	n.d.	n.d.	-	[395]
$[Co(NH_3)_5(5CNTz)](ClO_4)_2$	n.d.	n.d.	n.d.	n.d.	32.1	n.d.	n.d.	n.d.	n.d.	n.d.	n.d.	det.	n.d.	n.d.	[398]
$[Co(1M5ATz)(NH_3)_5](ClO_4)_3$	40%/ 12.8 J	n.d.	n.d.	n.d.	25.9	218	1.95	n.d.	n.d.	7320	n.d.	n.d.	n.d.	n.d.	[399]
$[Co(15DATz)(NH_3)_5](ClO_4)_3$	12%/ 12.8 J	n.d.	n.d.	n.d.	28.4	216	2.03	n.d.	n.d.	7680	n.d.	n.d.	n.d.	n.d.	[399]
$[Cu(11TNP)_3](ClO_4)_2$	<1	10	n.d.	n.d.	44.7	246	n.d.	n.d.	n.d.	n.d.	n.d.	def.	def.	n.d.	[400]
$[Fe(11TNP)_4(ClO_4)_2]$	<1	9	n.d.	n.d.	48.3	220	n.d.	n.d.	n.d.	n.d.	n.d.	def.	def.	n.d.	[400]
$[Cu(1TzAN)_6](ClO_4)_2$	<1	108	33	100-500	45.8	173	1.697	n.d.	n.d.	n.d.	n.d.	def.	def.	dec.	[73]
$[Cu_2(\mu\text{-}1TzAN)_2(1TzAN)_8](ClO_4)_4$	<1	60	160	100-500	43.3	177	1.667	n.d.	n.d.	n.d.	n.d.	det.	def.	det.	[73]
$[Fe(1TzAN)_6](ClO_4)_2$	2	60	33	100-500	46.2	188	1.701	n.d.	n.d.	n.d.	n.d.	def.	def.	def.	[73]
$[Zn(1TzAN)_6](ClO_4)_2$	10	240	1080	100-500	45.7	180	1.653	n.d.	n.d.	n.d.	n.d.	dec.	dec.	n.d.	[73]
$[Ag(\mu\text{-}1TzAN)(ClO_4)]$	<1	1	5	100-500	22.1	173	2.54	n.d.	n.d.	n.d.	n.d.	def.	def.	-	[73]
$[Cu(\mu\text{-}2TzAN)_2(2TzAN)_2](ClO_4)_2$	<1	1.5	13	<100	40.1	190	1.831	n.d.	n.d.	n.d.	n.d.	det.	def.	det.	[73]
$[Ag(\mu\text{-}2TzAN)_2](ClO_4)$	<1	6	5	100-500	32.9	67	2.159	n.d.	n.d.	n.d.	n.d.	det.	def.	-	[73]
$[Cu(1PyrTz)_6](ClO_4)_2$	2	5	25	100-500	36.9	135	1.515	n.d.	n.d.	n.d.	n.d.	det.	def.	det.	[125]
$[Fe(1PyrTz)_6](ClO_4)_2\cdot H_2O$	2	6	50	100-500	36.5	168	n.d.	n.d.	n.d.	n.d.	n.d.	det.	def.	det.	[125]
$[Zn(1PyrTz)_6](ClO_4)_2\cdot H_2O$	2	15	50	100-500	36.1	164	n.d.	n.d.	n.d.	n.d.	n.d.	def.	def.	-	[125]
**TETRAZOLE SALTS**															
HTz	<4	>360	n.d.	n.d.	80.0	188	1.529	237	3941	7813	210	explosive upon heating			[189, 196]
LiTz	>100	>360	n.d.	n.d.	73.7	380	1.472	76	n.d.	n.d.	n.d.	n.d.	n.d.	n.d.	[196]
$NaTz\cdot H_2O$	>100	>360	n.d.	n.d.	20.9	303	1.745	-291	n.d.	n.d.	n.d.	n.d.	n.d.	n.d.	[196]
KTz	>100	>360	n.d.	n.d.	51.8	308	1.774	174	n.d.	n.d.	n.d.	n.d.	n.d.	n.d.	[196]
RbTz	>100	>360	n.d.	n.d.	36.3	240	2.367	150	n.d.	n.d.	n.d.	n.d.	n.d.	n.d.	[196]
CsTz	>100	>360	n.d.	n.d.	27.7	305	3.118	144	n.d.	n.d.	n.d.	n.d.	n.d.	n.d.	[196]
AgTz	n.d.	n.d.	n.d.	n.d.	n.d.	n.d.	n.d.	n.d.	n.d.	n.d.	n.d.	explosive upon heating			[190]
$Ba(Tz)_2$	35	>360	800	n.d.	40.7	323	2.80	-3	5905	n.d.	n.d.	n.d.	n.d.	n.d.	[191]

Table S6. cont.

Compound	*IS* [J]	*FS* [N]	*ESD* [mJ]	Grain size [µm]	N content [%]	T_{dec} [°C]	*d* [g cm^{-3}]	*HOF* [kJ mol^{-1}]	ΔU_{ex} [kJ kg^{-1}]	*DV* [m s^{-1}]	p_{ex} [kbar]	HN	HP	Laser test	Ref.
$Cu(5ClTz)_2$	<1	<5	25	n.d.	41.4	275	n.d.	n.d.	n.d.	n.d.	n.d.	explosive upon heating			[193, 194]
$Cu(5BrTz)_2$	<1	<5	20	n.d.	31.4	265	n.d.	n.d.	n.d.	n.d.	n.d.	explosive upon heating			[193, 194]
$Na5ClTz{\cdot}2H_2O$	insensitive			n.d.	34.4	n.d.	n.d.	n.d.	n.d.	n.d.	n.d.	n.d.	n.d.	n.d.	[193]
$Hg_2(5HOTz)_2$	n.d.	n.d.	n.d.	n.d.	n.d.	n.d.	n.d.	n.d.	n.d.	n.d.	n.d.	explosive upon heating			[194]
$Hg(1MTz)_2$	n.d.	n.d.	n.d.	n.d.	30.6	n.d.	n.d.	n.d.	n.d.	n.d.	n.d.	n.d.	+	n.d.	[192]
$[Cu_4Na(5MTz)_5(CH_3CN)]_n$	36	>360	n.d.	n.d.	40.1	384	1.975	n.d.	9898	7225	244	n.d.	n.d.	n.d.	[195]
Li5ATz	>75	n.d.	n.d.	n.d.	77.0	n.d.	1.736	-399	6422	n.d.	n.d.	n.d.	n.d.	n.d.	[198]
$Na5ATz{\cdot}3H_2O$	n.d.	n.d.	n.d.	n.d.	43.5	n.d.	1.554	-1136	4100	n.d.	n.d.	n.d.	n.d.	n.d.	[198]
K5ATz	n.d.	n.d.	n.d.	n.d.	56.9	n.d.	1.961	-202	6209	n.d.	n.d.	n.d.	n.d.	n.d.	[198]
Rb5ATz	n.d.	n.d.	n.d.	n.d.	41.3	n.d.	2.474	-26	5565	n.d.	n.d.	n.d.	n.d.	n.d.	[198]
Cs5ATz	n.d.	n.d.	n.d.	n.d.	32.3	n.d.	2.837	3	4514	n.d.	n.d.	n.d.	n.d.	n.d.	[198]
$Mg(5ATz)_2{\cdot}4H_2O$	>40	>360	-	n.d.	53.0	≥300	1.702	n.d.	n.d.	n.d.	n.d.	n.d.	n.d.	n.d.	[197]
$Ca(5ATz)_2{\cdot}6H_2O$	>40	>360	-	n.d.	44.3	≥355	1.809	n.d.	n.d.	n.d.	n.d.	n.d.	n.d.	n.d.	[197]
$Sr(5ATz)_2{\cdot}6H_2O$	>40	>360	-	n.d.	38.5	≥350	1.712	n.d.	n.d.	n.d.	n.d.	n.d.	n.d.	n.d.	[197]
$Ba(5ATz)_2{\cdot}4H_2O$	>40	>360	-	n.d.	37.1	≥360	n.d.	n.d.	n.d.	n.d.	n.d.	n.d.	n.d.	n.d.	[197]
	>100	244	1250	n.d.	37.1	360	2.13	-925	5657	n.d.	n.d.	n.d.	n.d.	n.d.	[191]
$Na5CNTz{\cdot}1.5H_2O$	>40	>360	n.d.	n.d.	48.6	275	1.597	n.d.	n.d.	n.d.	n.d.	n.d.	n.d.	n.d.	[218]
	40	160	380	100-500	48.6	263	n.d.	n.d.	n.d.	n.d.	n.d.	n.d.	n.d.	n.d.	[219]
K5CNTz	>40	>360	n.d.	n.d.	52.6	265	1.923	n.d.	n.d.	n.d.	n.d.	n.d.	n.d.	n.d.	[218]
Cs5CNTz	n.d.	n.d.	n.d.	n.d.	38.5	n.d.	2.730	n.d.	n.d.	n.d.	n.d.	n.d.	n.d.	n.d.	[217]
Ag5CNTz	<1	<5	1	<100	34.7	284	n.d.	n.d.	n.d.	n.d.	n.d.	n.d.	n.d.	n.d.	[219]
$Sr(5CATz)_2{\cdot}3H_2O$	-	-	n.d.	500-1000	27.0	337	n.d.	n.d.	n.d.	n.d.	n.d.	n.d.	n.d.	n.d.	[220]
$Ba(5CATz)_2{\cdot}3H_2O$	-	-	n.d.	500-1000	18.5	366	n.d.	n.d.	n.d.	n.d.	n.d.	n.d.	n.d.	n.d.	[220]
$Cu(5CATz)_2{\cdot}3.5H_2O$	-	>288	n.d.	80-160	23.5	254	n.d.	n.d.	n.d.	n.d.	n.d.	n.d.	n.d.	n.d.	[220]
$Mn(5CATz)_2{\cdot}4H_2O$	-	>324	n.d.	250-500	21.8	386	n.d.	n.d.	n.d.	n.d.	n.d.	n.d.	n.d.	n.d.	[220]
$Ag5CATz{\cdot}1.56H_2O$	-	-	n.d.	250-500	20.8	200	n.d.	n.d.	n.d.	n.d.	n.d.	n.d.	n.d.	n.d.	[220]
$Li5NTz{\cdot}3H_2O$	25	324	n.d.	n.d.	40.0	270	1.609	610	5606	n.d.	n.d.	n.d.	n.d.	n.d.	[200]
$Na5NTz{\cdot}2H_2O$	>30	~360	n.d.	n.d.	40.5	200	1.731	350	5021	n.d.	n.d.	n.d.	n.d.	n.d.	[200]
Na5NTz	+	n.d.	n.d.	n.d.	51.1	n.d.	n.d.	n.d.	n.d.	n.d.	n.d.	+	n.d.	n.d.	[205]
K5NTz	10	<5	n.d.	n.d.	45.7	195	2.027	n.d.	n.d.	n.d.	n.d.	n.d.	n.d.	n.d.	[200]
Rb5NTz	5	<5	n.d.	n.d.	35.1	192	2.489	n.d.	n.d.	n.d.	n.d.	n.d.	n.d.	n.d.	[200]
Cs5NTz	10	<5	n.d.	n.d.	28.4	194	2.986	n.d.	n.d.	n.d.	n.d.	n.d.	n.d.	n.d.	[200]
$Mg(5NTz)_2{\cdot}6H_2O$	>40	240	+	n.d.	38.9	195	n.d.	n.d.	n.d.	n.d.	n.d.	n.d.	n.d.	n.d.	[201]
$Ca(5NTz)_2{\cdot}6H_2O$	35	84	+	n.d.	37.2	180	n.d.	n.d.	n.d.	n.d.	n.d.	n.d.	n.d.	n.d.	[201]

Table S6. cont.

Compound	*IS* [J]	*FS* [N]	*ESD* [mJ]	Grain size [µm]	N content [%]	T_{dec} [°C]	*d* [g cm^{-3}]	*HOF* [kJ mol^{-1}]	ΔU_{ex} [kJ kg^{-1}]	*DV* [m s^{-1}]	p_{ex} [kbar]	HN	HP	Laser test	Ref.
$Sr(5NTz)_2{\cdot}5H_2O$	15	48	+	n.d.	34.5	210	n.d.	n.d.	n.d.	n.d.	n.d.	n.d.	n.d.	n.d.	[201]
$Ba(5NTz)_2{\cdot}5H_2O$	2.5-5	<20	+	n.d.	30.8	235	n.d.	n.d.	n.d.	n.d.	n.d.	n.d.	n.d.	n.d.	[201]
$Sr(Tz)_2{\cdot}5H_2O$	>100	>360	1000	n.d.	35.5	335	1.88	n.d.	5476	n.d.	n.d.	n.d.	n.d.	n.d.	[196, 303]
Cu5NTz	0.04	0.1	0.012	n.d.	39.4	337	n.d.	n.d.	n.d.	n.d.	n.d.	n.d.	n.d.	n.d.	[133]
	0.051	0.4	7.3	n.d.		321	n.d.	n.d.	n.d.	n.d.	n.d.	n.d.	n.d.	n.d.	[208]
$Cu(5NTz)_2{\cdot}H5NTz{\cdot}2H_2O$	<3	<5	+	n.d.	47.5	230	n.d.	n.d.	n.d.	n.d.	n.d.	n.d.	+	n.d.	[207]
$[Cu(en)_2](5NTz)_2$	<7	<252	-	n.d.	47.7	225	1.757	n.d.	n.d.	n.d.	n.d.	n.d.	+	n.d.	[207]
$[Cu(NH_3)_3(5NTz)_2]$	<2	<18	-	n.d.	53.2	245	n.d.	n.d.	n.d.	n.d.	n.d.	n.d.	+	n.d.	[207]
Ag5NTz	<1	<5	+	n.d.	31.6	273	n.d.	n.d.	n.d.	n.d.	n.d.	n.d.	+	n.d.	[207]
[Ag(en)]5NTz	>40	<240	-	n.d.	34.8	212	n.d.	n.d.	n.d.	n.d.	n.d.	n.d.	+	n.d.	[207]
$Hg(5NTz)_2$	*1.75*	+	*0.27*	n.d.	32.7	190	n.d.	n.d.	n.d.	n.d.	n.d.	n.d.	n.d.	n.d.	[204, 205]
$Pb(5NTz)_2{\cdot}Pb(OH)_2$	more than MF			n.d.	20.7	~220	n.d.	n.d.	n.d.	n.d.	n.d.	n.d.	n.d.	n.d.	[203]
$Na_2[Fe(5NTz)_4(H_2O)_2]$	*3*	*0.2*	>360	n.d.	47.2	250	n.d.	n.d.	n.d.	n.d.	n.d.	n.d.	n.d.	n.d.	[214]
$Na_2[Cu(5NTz)_4(H_2O)_2]$	*3*	*0.4*	>360	n.d.	46.6	259	n.d.	n.d.	n.d.	n.d.	n.d.	n.d.	n.d.	n.d.	[214]
$(NH_4)_2[Fe(5NTz)_4(H_2O)_2]$	*3*	*27*	>360	n.d.	52.8	255	2.18	n.d.	n.d.	7140	n.d.	n.d.	n.d.	n.d.	[214, 216]
$(NH_4)_2[Cu(5NTz)_4(H_2O)_2]$	*3*	*5*	>360	n.d.	52.1	265	n.d.	n.d.	n.d.	n.d.	n.d.	n.d.	n.d.	n.d.	[214]
$NH_4[Fe(5NTz)_3(H_2O)_3]$	*4*	*42*	>360	n.d.	47.7	261	2.10	n.d.	n.d.	n.d.	n.d.	n.d.	n.d.	n.d.	[215]
$(NH_4)_3[Fe(5NTz)_5(H_2O)]$	*2.5*	*13*	>360	n.d.	55.1	253	2.34	n.d.	n.d.	n.d.	n.d.	n.d.	n.d.	n.d.	[215]
$(NH_4)_4[Fe(5NTz)_6]$	*2*	*8*	>360	n.d.	56.4	252	2.45	n.d.	n.d.	n.d.	n.d.	n.d.	n.d.	n.d.	[215]
$NH_4[Co(5NTz)_3(H_2O)_3]$	*5.5*	*8*	>360	n.d.	47.4	270	2.04	n.d.	n.d.	6740	n.d.	n.d.	n.d.	n.d.	[216]
$(NH_4)_2[Ni(5NTz)_4(H_2O)_2]$	*4.5*	*15*	>360	n.d.	52.2	270	2.44	n.d.	n.d.	7020	n.d.	n.d.	n.d.	n.d.	[216]
$(NH_4)_2[Cu(5NTz)_4(H_2O)_2]$	*5.75*	*6*	>360	n.d.	52.1	265	1.94	n.d.	n.d.	7390	n.d.	n.d.	n.d.	n.d.	[216]
$Na[Co(5NTz)_3(H_2O)_3]$	+	*<5*	>360	n.d.	44.0	264	n.d.	n.d.	n.d.	n.d.	n.d.	n.d.	n.d.	n.d.	[216]
$Na[Ni(5NTz)_4(H_2O)_2]$	+	*<5*	>360	n.d.	46.9	265	n.d.	n.d.	n.d.	n.d.	n.d.	n.d.	n.d.	n.d.	[216]
$Na_2[Cu(5NTz)_4(H_2O)_2]$	+	*<5*	>360	n.d.	46.6	250	n.d.	n.d.	n.d.	n.d.	n.d.	n.d.	n.d.	n.d.	[216]
$[Co(NH_3)_4(NO_2)_2](5NTz)$	5	>216	n.d.	n.d.	43.9	230	n.d.	n.d.	n.d.	n.d.	n.d.	n.d.	n.d.	n.d.	[46]
HAzTz	<1	<5	<2.2	n.d.	88.3	165	1.720	611	5721	8986	327	n.d.	n.d.	n.d.	[564]
$LiAzTz{\cdot}H_2O$	1	11	70	1-50	72.6	152	1.683	n.d.	n.d.	n.d.	n.d.	n.d.	n.d.	n.d.	[221]
$NaAzTz{\cdot}H_2O$	<1	<5	20	1-50	64.9	155	1.743	n.d.	n.d.	n.d.	n.d.	n.d.	n.d.	n.d.	[221]
KAzTz	extremely sensitive			n.d.	65.7	148	1.917	n.d.	n.d.	n.d.	n.d.	n.d.	n.d.	n.d.	[221]
CsAzTz	spontaneous explosion			n.d.	40.4	n.d.	2.810	n.d.	n.d.	n.d.	n.d.	n.d.	n.d.	n.d.	[221]
$Ca(AzTz){\cdot}13/3H_2O$	2	25	160	1-50	55.0	173	1.657	n.d.	n.d.	n.d.	n.d.	n.d.	n.d.	n.d.	[221]
AgAzTz	extremely sensitive			n.d.	n.d.	n.d.	n.d.	n.d.	n.d.	n.d.	n.d.	extremely sensitive			[222]

Table S6. cont.

Compound	*IS* [J]	*FS* [N]	*ESD* [mJ]	Grain size [µm]	N content [%]	T_{dec} [°C]	*d* [g cm^{-3}]	*HOF* [kJ mol^{-1}]	ΔU_{ex} [kJ kg^{-1}]	*DV* [m s^{-1}]	p_{ex} [kbar]	HN	HP	Laser test	Ref.
H$_2$5NATz	1	8	n.d.	n.d.	64.6	n.d.	n.d.	n.d.	n.d.	n.d.	n.d.	n.d.	n.d.	n.d.	[229]
H1M5NATz	12	160	n.d.	n.d.	58.3	n.d.	n.d.	n.d.	n.d.	n.d.	n.d.	n.d.	n.d.	n.d.	[229]
H2M5NATz	3	145	n.d.	n.d.	58.3	n.d.	n.d.	n.d.	n.d.	n.d.	n.d.	n.d.	n.d.	n.d.	[229]
Na$_2$NATz	n.d.	n.d.	n.d.	n.d.	47.9	207	n.d.	n.d.	n.d.	n.d.	n.d.	n.d.	n.d.	n.d.	[224]
Ca5NATz·5H$_2$O	75	240	1050	n.d.	32.6	360	1.9	n.d.	n.d.	n.d.	n.d.	n.d.	def.	n.d.	[228]
Ca5NATz	5	112	150	n.d.	50.0	360	2.0	195.1	4632	n.d.	n.d.	n.d.	def.	n.d.	[228]
Sr5NATz·2H$_2$O	30	>360	1000	n.d.	33.4	n.d.	n.d.	n.d.	n.d.	n.d.	n.d.	n.d.	n.d.	n.d.	[227]
Sr(5HNATz)$_2$·4H$_2$O	20	288	400	n.d.	40.2	n.d.	n.d.	n.d.	n.d.	n.d.	n.d.	n.d.	n.d.	n.d.	[227]
Sr(2M5NATz)$_2$·4H$_2$O	>50	>360	10	n.d.	37.7	n.d.	n.d.	n.d.	n.d.	n.d.	n.d.	n.d.	n.d.	n.d.	[227]
Sr(1M5NATz)$_2$·H$_2$O	>40	>360	900	n.d.	42.9	350	2.19	n.d.	7411	n.d.	n.d.	n.d.	n.d.	n.d.	[303] [227]
Sr(1ClP5NATz)$_2$·H$_2$O	>10	>360	750	n.d.	34.4	208	2.03	n.d.	7030	n.d.	n.d.	n.d.	n.d.	n.d.	[303]
Ba5NATz·2H$_2$O	30	240	600	n.d.	27.9	376	2.91	-660	2860	n.d.	n.d.	n.d.	n.d.	n.d.	[191]
Ba5NATz	*ca.* LA sensitivity		330	n.d.	31.7	385	n.d.	n.d.	n.d.	n.d.	n.d.	n.d.	n.d.	n.d.	[226]
Ba(5NATz)$_2$·4H$_2$O	7	252	600	n.d.	36.0	236	2.33	-797	4270	n.d.	n.d.	n.d.	n.d.	n.d.	[191]
Ba(1M5NATz)$_2$·H$_2$O	15	>360	650	n.d.	38.1	349	2.30	-612	6050	n.d.	n.d.	n.d.	n.d.	n.d.	[191]
Ba(2M5NATz)$_2$·2H$_2$O	7	>360	500	n.d.	36.6	257	2.15	-317	7071	n.d.	n.d.	n.d.	n.d.	n.d.	[191]
[Cu(H5NATz)$_2$(H$_2$O)$_4$]	30	360	n.d.	n.d.	42.7	128	2.062	n.d.	n.d.	n.d.	n.d.	n.d.	n.d.	n.d.	[229]
[Cu$_2$(5NATz)$_2$(NH$_3$)$_6$]	>50	>360	n.d.	n.d.	51.9	217	2.010	n.d.	n.d.	n.d.	n.d.	n.d.	n.d.	n.d.	[229]
(NH$_4$)$_2$[Cu(5NATz)$_2$(H$_2$O)$_2$]	>40	360	n.d.	n.d.	49.7	220	2.011	n.d.	n.d.	n.d.	n.d.	n.d.	n.d.	n.d.	[229]
[Cu(1M5NATz)$_2$(NH$_3$)$_2$]	15	>360	n.d.	n.d.	51.1	243	1.850	n.d.	n.d.	n.d.	n.d.	n.d.	n.d.	n.d.	[229]
[Cu(2M5NATz)$_2$(H2M5NATz)$_2$]	2	30	n.d.	n.d.	52.7	124	1.859	n.d.	n.d.	n.d.	n.d.	n.d.	n.d.	n.d.	[229]
[Cu(2M5NATz)$_2$]	1	18	n.d.	n.d.	52.7	218	2.073	n.d.	n.d.	n.d.	n.d.	n.d.	n.d.	n.d.	[229]
[Cu(2M5NATz)$_2$(NH$_3$)$_2$]	20	300	n.d.	n.d.	49.2	200	1.864	n.d.	n.d.	n.d.	n.d.	n.d.	n.d.	n.d.	[229]
[Cu(2M5NATz)$_2$(NH$_3$)$_4$]·H$_2$O	40	50	n.d.	n.d.	51.4	317	1.718	n.d.	9052	n.d.	n.d.	n.d.	n.d.	n.d.	[229]
[Cu(1M5NATz)$_2$(H$_2$O)$_2$]·2H$_2$O	>100	>360	n.d.	n.d.	39.9	256	1.870	n.d.	n.d.	n.d.	n.d.	n.d.	n.d.	n.d.	[230]
[Cu(1M5NATz)$_2$(H$_2$O)$_2$]	>100	>360	n.d.	n.d.	43.8	256	2.035	n.d.	n.d.	n.d.	n.d.	n.d.	n.d.	n.d.	[230]
[Cu(1M5NATz)$_2$]	>0.7	>40	n.d.	n.d.	48.1	252	2.067	-83.7	n.d.	n.d.	n.d.	n.d.	n.d.	n.d.	[230]
[Cu(1M5NATz)$_2$(H$_2$O)$_2$]·2MeOH	>55	>360	n.d.	n.d.	37.4	n.d.	1.740	n.d.	n.d.	n.d.	n.d.	n.d.	n.d.	n.d.	[230]
[Ni(NH$_3$)$_3$(5NATz)]$_2$	explode			n.d.	53.0	n.d.	1.94	n.d.	n.d.	n.d.	n.d.	n.d.	n.d.	n.d.	[225]
Ag1M5NATz	>50	>360	n.d.	n.d.	33.5	n.d.	2.948	n.d.	n.d.	n.d.	n.d.	n.d.	n.d.	n.d.	[231]
Li1M5NATz·H$_2$O	>100	>360	n.d.	n.d.	50.0	>300	1.756	-401	8368	n.d.	n.d.	n.d.	n.d.	n.d.	[232]
Na1M5NATz	>100	>360	n.d.	n.d.	50.6	>300	1.939	9	8954	n.d.	n.d.	n.d.	n.d.	n.d.	[232]
K1M5NATz	>100	>360	n.d.	n.d.	46.1	>300	1.948	4	8284	n.d.	n.d.	n.d.	n.d.	n.d.	[232]
Rb1M5NATz	>100	>360	n.d.	n.d.	37.0	>300	2.258	-148	5950	n.d.	n.d.	n.d.	n.d.	n.d.	[232]
Cs1M5NATz	>100	>360	n.d.	n.d.	30.8	>300	2.618	-217	4707	n.d.	n.d.	n.d.	n.d.	n.d.	[232]

Table S6. cont.

Compound	*IS* [J]	*FS* [N]	*ESD* [mJ]	Grain size [µm]	N content [%]	T_{dec} [°C]	*d* [g cm^{-3}]	*HOF* [kJ mol^{-1}]	ΔU_{ex} [kJ kg^{-1}]	*DV* [m s^{-1}]	p_{ex} [kbar]	HN	HP	Laser test	Ref.
$H_2$15DNATz	1	<5	n.d.	n.d.	59.0	110	1.93	486.3	5921	9967	434	n.d.	n.d.	n.d.	[238]
$K_2$15DNATz	1	<5	n.d.	n.d.	42.1	240	2.137	-112.4	3938	10011	522	n.d.	n.d.	n.d.	[238]
$K_2Fe(15DNATz)_2·4H_2O$	3	16	n.d.	n.d.	38.5	154	2.095	73.2	4911	8147	298	n.d.	n.d.	n.d.	[240]
$K_2Cu(15DNATz)_2·4H_2O$	3	16	n.d.	n.d.	38.0	167	2.135	-41.3	3438	8348	315	n.d.	n.d.	n.d.	[240]
$K_2Ni(15DNATz)_2·4H_2O$	6	26	n.d.	n.d.	38.3	209	2.111	74.6	4798	8169	297	n.d.	n.d.	n.d.	[240]
$K_2Co(15DNATz)_2·4H_2O$	5	22	n.d.	n.d.	38.3	185	2.124	170.5	5143	8188	303	n.d.	n.d.	n.d.	[240]
$K_2Zn(15DNATz)_2·4H_2O$	4	18	n.d.	n.d.	37.9	178	2.138	71.3	3751	8478	328	n.d.	n.d.	n.d.	[240]
$[Ag_7(5CMTz)_3(H5CMTz)_2(H_25CMTz)(H_2O)]_n$	>40	>360	n.d.	n.d.	21.9	260	2.572	8930.2	8033	8016	345	n.d.	n.d.	n.d.	[235]
$[Ag_7(5CMTz)_3(H5CMTz)_2(H_25CMTz)]_n$	>40	353	n.d.	n.d.	22.2	250	2.566	9706.2	8452	8089	350	n.d.	n.d.	n.d.	[235]
HTzFOX	30	>360	n.d.	n.d.	57.3	275	1.83	181.5	n.d.	8499	267	n.d.	n.d.	n.d.	[237]
KTzFOX	7.5	100	n.d.	n.d.	46.9	292	1.92	124.7	n.d.	8057	242	n.d.	n.d.	n.d.	[237]
CuTzFOX	10	120-160	n.d.	n.d.	41.7	340	2.16	n.d.	n.d.	n.d.	n.d.	n.d.	n.d.	n.d.	[236]
Na1ClENATz	>50	>360	n.d.	n.d.	39.2	184	1.86	n.d.	n.d.	n.d.	n.d.	n.d.	n.d.	n.d.	[245]
K1AzENATz	25	300	n.d.	n.d.	53.2	205	1.78	n.d.	n.d.	n.d.	n.d.	n.d.	n.d.	n.d.	[245]
$[Cu(1OHENATz)_2(H_2O)_2]$	>50	n.d.	n.d.	n.d.	37.7	245	1.821	n.d.	n.d.	n.d.	n.d.	n.d.	n.d.	n.d.	[245]
$[Cu(1ClENATz)_2(H_2O)_2]·2H_2O$	>50	n.d.	n.d.	n.d.	32.4	233	1.871	n.d.	n.d.	n.d.	n.d.	n.d.	n.d.	n.d.	[245]
$[Cu(1AzENATz)_2(H_2O)_2]$	10	n.d.	n.d.	n.d.	50.9	217	1.764	n.d.	n.d.	n.d.	n.d.	n.d.	n.d.	n.d.	[245]
H2AzENATz	10	360	-	n.d.	62.4	93	1.60	647.1	5281	8138	248	n.d.	n.d.	n.d.	[233]
K2AzENATz	1	30	25	n.d.	53.1	180	1.93	n.d.	n.d.	n.d.	n.d.	n.d.	n.d.	n.d.	[233]
Ag2AzENATz	<1	15	13	n.d.	41.2	181	2.362	n.d.	n.d.	n.d.	n.d.	n.d.	n.d.	n.d.	[233]
HNIATz	<0.25	<1	25	100-500	67.6	85	1.835	496	6498	9460	403	n.d.	n.d.	n.d.	[239]
AgNIATz	<1	<5	n.d.	100-500	38.9	158	n.d.	n.d.	n.d.	n.d.	n.d.	n.d.	n.d.	n.d.	[239]
K1NATz	1	<0.1	0.9	n.d.	50.0	180	1.926	93.4	4613	7643	237	n.d.	n.d.	n.d.	[241, 242]
K2NATz	5	<0.1	8.2	n.d.	50.0	176	1.938	77.9	4526	7882	251	n.d.	n.d.	n.d.	[242]
KNIATz	1	2	35	n.d.	53.5	180	1.955	-210.5	2723	7567	218	n.d.	n.d.	n.d.	[241]
HMeHTz	>40	n.d.	n.d.	n.d.	73.7	193	1.540	286	16954	n.d.	n.d.	n.d.	n.d.	n.d.	[244]
$Cu(MeHTz)_2$	>40	n.d.	n.d.	n.d.	58.0	212	2.031	93	11259	n.d.	n.d.	n.d.	n.d.	n.d.	[244]
$AgMeHTz·H_2O$	>40	n.d.	n.d.	n.d.	35.2	200	2.465	-263	6460	n.d.	n.d.	n.d.	n.d.	n.d.	[244]
$[CuCl(\mu_3-Cl)(MeHTz)]·H_2O$	40	252	1500	<100	31.5	185	2.098	n.d.	n.d.	n.d.	n.d.	dec.	dec.	n.d.	[243]
$H_2DNABTz$	<1	<5	30	n.d.	65.1	107	1.930	932.1	6629	10142	456	n.d.	n.d.	n.d.	[267]
$Li_2DNABTz·2H_2O$	<1	<5	20	<100	54.9	239	1.538	n.d.	n.d.	n.d.	n.d.	dec.	det.	n.d.	[265]
$Na_2DNABTz·2H_2O$ (α)	<1	<5	30	500-1000	49.7	241	1.889	n.d.	n.d.	n.d.	n.d.	dec.	det.	n.d.	[265]
$Na_2DNABTz·2H_2O$ (β)	<1	<5	150	500-1000	49.7	247	1.887	n.d.	n.d.	n.d.	n.d.	dec.	det.	n.d.	[265]

Table S6. cont.

Compound	*IS* [J]	*FS* [N]	*ESD* [mJ]	Grain size [µm]	N content [%]	T_{dec} [°C]	*d* [g cm^{-3}]	*HOF* [kJ mol^{-1}]	ΔU_{ex} [kJ kg^{-1}]	*DV* [m s^{-1}]	p_{ex} [kbar]	HN	HP	Laser test	Ref.
K_2DNABTz	1	≤1	3	n.d.	50.3	200	2.172	326.4	4959	8330	317	n.d.	n.d.	n.d.	[266]
Rb_2DNABTz	<1	<5	20	100-500	39.4	186	2.501	n.d.	n.d.	n.d.	n.d.	det.	det.	n.d.	[265]
Cs_2DNABTz	<1	<5	<20	100-500	32.2	220	2.774	n.d.	n.d.	n.d.	n.d.	det.	det.	n.d.	[265]
Ag_2DNABTz	n.d.	n.d.	n.d.	n.d.	35.6	n.d.	3.177	n.d.	n.d.	n.d.	n.d.	n.d.	n.d.	n.d.	[265]
CaDNABTz·$4H_2O$	n.d.	n.d.	n.d.	n.d.	45.7	n.d.	n.d.	n.d.	n.d.	n.d.	n.d.	n.d.	n.d.	n.d.	[265]
SrDNABTz·$4H_2O$	n.d.	n.d.	n.d.	n.d.	40.4	n.d.	1.937	n.d.	n.d.	n.d.	n.d.	n.d.	n.d.	n.d.	[265]
BaDNABTz·$6H_2O$	n.d.	n.d.	n.d.	n.d.	33.5	n.d.	2.156	n.d.	n.d.	n.d.	n.d.	n.d.	n.d.	n.d.	[265]
$[Ni(NH_3)_6]$DNABTz	3	80	130	100-500	60.5	167	1.675	n.d.	n.d.	n.d.	n.d.	def.	det.	det.	[265]
$[Cu(NH_3)_4]$DNABTz·H_2O	2	10	150	100-500	55.2	155	1.809	n.d.	n.d.	n.d.	n.d.	dec.	def.	det.	[265]
$[Zn(NH_3)_4]$DNABTz	1.5	6	100	100-500	57.5	182	1.777	n.d.	n.d.	n.d.	n.d.	def.	det.	n.d.	[265]
Be(55BTz)·$5H_2O$	n.d.	n.d.	n.d.	n.d.	47.7	no dec.	1.605	n.d.	n.d.	n.d.	n.d.	n.d.	n.d.	n.d.	[246]
Mg(55BTz)·$5H_2O$	40	360	300	<100	34.7	no dec.	1.584	n.d.	n.d.	n.d.	n.d.	n.d.	n.d.	n.d.	[246]
Ca(55BTz)·$9H_2O$	40	360	200	<100	42.1	no dec.	1.765	n.d.	n.d.	n.d.	n.d.	n.d.	n.d.	n.d.	[246]
Sr(55BTz)·$4H_2O$	35	360	150	<100	31.7	no dec.	1.608	n.d.	n.d.	n.d.	n.d.	n.d.	n.d.	n.d.	[246]
Ba(55BTz)·$4H_2O$	30	360	150	100-500	32.4	no dec.	2.412	n.d.	n.d.	n.d.	n.d.	n.d.	n.d.	n.d.	[246]
Na(55BTz)·$3H_2O$	40	288	500	<100	52.3	300	n.d.	n.d.	n.d.	n.d.	n.d.	n.d.	n.d.	n.d.	[247]
$[Cu(H_2O)_2]$BTz	30	80	50	<100	47.6	238	n.d.	n.d.	n.d.	n.d.	n.d.	n.d.	det.	n.d.	[247]
H15TzTz	1	5	130	<100	81.1	145	1.728	622	4769	8355	282	n.d.	n.d.	n.d.	[248]
HM15BTZ	9	240	300	100-500	73.7	135	1.568	592	4301	7593	213	n.d.	n.d.	n.d.	[248]
Na15TzTz·$2H_2O$	>40	>360	n.d.	100-500	57.1	222	1.645	n.d.	n.d.	n.d.	n.d.	n.d.	n.d.	n.d.	[248]
K15TzTz·$2H_2O$	>40	>360	n.d.	100-500	52.8	205	1.627	n.d.	n.d.	n.d.	n.d.	n.d.	n.d.	n.d.	[248]
Cu15TzTz	<1	<5	5	100	55.9	136	n.d.	n.d.	n.d.	n.d.	n.d.	n.d.	n.d.	n.d.	[248]
Ag15TzTz	<1	<5	10	100	45.0	196	n.d.	n.d.	n.d.	n.d.	n.d.	n.d.	n.d.	n.d.	[248]
$[Cu(15TzTz)_2(H_2O)_4]$	>40	>360	n.d.	100-500	54.7	145	1.992	n.d.	n.d.	n.d.	n.d.	n.d.	n.d.	n.d.	[248]
$[Cu(15TzTz)_2(H_2O)_2(NH_3)_2]$	>40	>360	n.d.	100-500	61.8	155	1.829	n.d.	n.d.	n.d.	n.d.	n.d.	n.d.	n.d.	[248]
$[CuCl_2(M15BTZ)_2]$	>40	>360	n.d.	100-500	51.1	172	1.901	n.d.	n.d.	n.d.	n.d.	n.d.	n.d.	n.d.	[248]
K_2DNATzDA	<1	<5	40	n.d.	54.1	180	2.081	625.5	5398	8911	343	n.d.	n.d.	n.d.	[267]
KA15TzTz	17	>360	n.d.	n.d.	65.9	314	1.85	443.5	n.d.	8086	230	n.d.	n.d.	n.d.	[249]
HNA15TzTz	1	8	n.d.	n.d.	70.7	122	1.77	710.8	n.d.	9099	336	n.d.	n.d.	n.d.	[249]
KNA15TzTz	5	56	n.d.	n.d.	59.3	137	1.92	648.8	n.d.	9165	322	n.d.	n.d.	n.d.	[249]
H_2DNAHPTz	5	240	700	n.d.	53.2	156	1.60	467	5470	8024	240	n.d.	n.d.	n.d.	[268]

Table S6. cont.

Compound	*IS* [J]	*FS* [N]	*ESD* [mJ]	Grain size [µm]	N content [%]	T_{dec} [°C]	*d* [g cm^{-3}]	*HOF* [kJ mol^{-1}]	ΔU_{ex} [kJ kg^{-1}]	*DV* [m s^{-1}]	p_{ex} [kbar]	HN	HP	Laser test	Ref.
Li$_2$DNAHPBTz·2H$_2$O	>40	>360	>1500	50-100	46.2	160	n.d.	n.d.	n.d.	n.d.	n.d.	n.d.	n.d.	n.d.	[268]
Na$_2$DNAHPBTz·4H$_2$O	>40	>360	>1500	50-100	38.9	245	n.d.	n.d.	n.d.	n.d.	n.d.	n.d.	n.d.	n.d.	[268]
K$_2$DNAHPBTz·H$_2$O	>40	>360	>1500	50-100	40.9	271	n.d.	n.d.	n.d.	n.d.	n.d.	n.d.	n.d.	n.d.	[268]
SrDNAHPBTz·2H$_2$O	>40	>360	>1500	50-100	38.4	299	n.d.	n.d.	n.d.	n.d.	n.d.	n.d.	n.d.	n.d.	[268]
BaDNAHPBTz·2H$_2$O	>40	>360	>1500	50-100	34.5	166	n.d.	n.d.	n.d.	n.d.	n.d.	n.d.	n.d.	n.d.	[268]
Na$_2$BTzDAO·5H$_2$O	40	324	200	500-1000	44.3	222	n.d.	n.d.	n.d.	n.d.	n.d.	n.d.	n.d.	n.d.	[257]
BaBTzDAO·5H$_2$O	7	160	600	500-1000	34.4	188	n.d.	n.d.	n.d.	n.d.	n.d.	n.d.	n.d.	n.d.	[257]
CuBTzDA	<3	n.d.	n.d.	n.d.	61.5	217	2.10	n.d.	n.d.	n.d.	n.d.	n.d.	n.d.	n.d.	[255]
[Cu(NH$_3$)$_4$]BTzDA·2H$_2$O	28	n.d.	n.d.	n.d.	59.1	189	1.75	-1490	n.d.	n.d.	n.d.	n.d.	n.d.	n.d.	[255]
CdBTzDA	<3	n.d.	n.d.	n.d.	50.7	239	2.59	n.d.	n.d.	n.d.	n.d.	n.d.	n.d.	n.d.	[255]
[Cd(NH$_3$)$_2$(H$_2$O)$_2$]BTzDA	25	n.d.	n.d.	n.d.	48.5	213	2.11	-1793	n.d.	n.d.	n.d.	n.d.	n.d.	n.d.	[255]
[Ag(NH$_3$)]$_2$BTzDA	2	<5	5	n.d.	40.6	n.d.	2.84	n.d.	n.d.	n.d.	n.d.	n.d.	n.d.	n.d.	[256]
[Co(NH$_3$)$_5$(NO$_2$)](BTzDA)·2H$_2$O	8	>216	n.d.	n.d.	57.4	>290	1.605	n.d.	n.d.	n.d.	n.d.	n.d.	n.d.	n.d.	[46]
[Co(NH$_3$)$_4$(py)(NO$_2$)](BTzDA)·2H$_2$O	>10	>360	n.d.	n.d.	49.6	260	1.671	n.d.	n.d.	n.d.	n.d.	n.d.	n.d.	n.d.	[46]
[Co(NH$_3$)$_4$(NO$_2$)$_2$]$_2$(BTzDA)	8	>216	n.d.	n.d.	51.2	>250	1.989	n.d.	n.d.	n.d.	n.d.	n.d.	n.d.	n.d.	[46]
[Co(NH$_3$)$_5$(NO$_2$)(N$_3$)](BTzDA)$_2$·H$_2$O	3.5-5	216	n.d.	n.d.	68.5	256	1.726	n.d.	n.d.	n.d.	n.d.	n.d.	n.d.	n.d.	[46]
[Cu(BTzA)(NH$_3$)$_2$]	>40	>360	750	n.d.	62.0	281	1.988	367.3	9353	n.d.	n.d.	n.d.	n.d.	n.d.	[251, 303]
(NH$_4$)$_2$[Cu(BTzA)$_2$]·2.5H$_2$O	>40	>360	n.d.	n.d.	64.0	250-270	1.353	-122.2	n.d.	n.d.	n.d.	n.d.	n.d.	n.d.	[251]
[Pb(BTzA) ·2H$_2$O]$_n$	>40	n.d.	n.d.	n.d.	32.0	342	3.250	n.d.	4970	8963	475				[252]
Na$_2$[Zn(BTzA)$_2$(H$_2$O)$_8$]·H$_2$O	>40	>360	n.d.	n.d.	43.8	355	1.823	39.1	n.d.	n.d.	n.d.	n.d.	n.d.	n.d.	[253]
K$_2$[Zn(BTzA)$_2$(H$_2$O)$_4$]	>40	>360	n.d.	n.d.	48.7	349	2.015	251.1	n.d.	n.d.	n.d.	n.d.	n.d.	n.d.	[253]
H$_2$BTzTt	6	72	200	n.d.	77.1	226	n.d.	n.d.	n.d.	n.d.	n.d.	n.d.	n.d.	n.d.	[259]
Na$_2$BTzTt·6H$_2$O	40	360	1000	n.d.	45.4	270	n.d.	n.d.	n.d.	n.d.	n.d.	n.d.	n.d.	n.d.	[259]
Cs$_2$BTzTt·H$_2$O	40	360	1000	n.d.	33.6	n.d.	n.d.	n.d.	n.d.	n.d.	n.d.	n.d.	n.d.	n.d.	[259]
Ag$_2$BTzTt	<1	<5	70	n.d.	38.9	219	n.d.	n.d.	n.d.	n.d.	n.d.	n.d.	n.d.	n.d.	[259]
NaBTzT	>40	>360	n.d.	n.d.	58.5	208	1.698	689.8	4897	7962	232	n.d.	n.d.	n.d.	[258]
KBTzT	>40	>360	n.d.	n.d.	55.1	185	1.674	614.8	4680	7546	207	n.d.	n.d.	n.d.	[258]
[Bi$_2$(OH)$_4$]BTzDN	n.d.	+	n.d.	n.d.	21.6	180	n.d.	n.d.	n.d.	n.d.	n.d.	n.d.	n.d.	n.d.	[274]
(BiO)$_2$BTzDN	n.d.	+	n.d.	n.d.	34.6	180	n.d.	n.d.	n.d.	n.d.	n.d.	n.d.	n.d.	n.d.	[274]
[(BiO)$_5$OH](BTzDN)$_2$	n.d.	+	n.d.	n.d.	10.7	180	n.d.	n.d.	n.d.	n.d.	n.d.	n.d.	n.d.	n.d.	[274]
[Bi$_2$(OH)$_4$]BTz	n.d.	+	n.d.	n.d.	18.0	280	n.d.	n.d.	n.d.	n.d.	n.d.	n.d.	n.d.	n.d.	[274]
[Bi$_3$(OH)$_6$]BTzN3	n.d.	+	n.d.	n.d.	17.0	240	n.d.	n.d.	n.d.	n.d.	n.d.	n.d.	n.d.	n.d.	[274]
[(BiO)$_5$OH](BTzH)$_2$	n.d.	+	n.d.	n.d.	8.8	150	n.d.	n.d.	n.d.	n.d.	n.d.	n.d.	n.d.	n.d.	[274]
[Bi(OH)$_2$]NTz	n.d.	-	n.d.	n.d.	19.6	196	n.d.	n.d.	n.d.	n.d.	n.d.	n.d.	n.d.	n.d.	[274]

Table S6. cont.

Compound	*IS* [J]	*FS* [N]	*ESD* [mJ]	Grain size [μm]	N content [%]	T_{dec} [°C]	*d* [g cm^{-3}]	*HOF* [kJ mol^{-1}]	ΔU_{ex} [kJ kg^{-1}]	*DV* [m s^{-1}]	p_{ex} [kbar]	HN	HP	Laser test	Ref.
$[Bi(OH)_2]NATz$	n.d.	-	n.d.	n.d.	23.6	190	n.d.	n.d.	n.d.	n.d.	n.d.	n.d.	n.d.	n.d.	[274]
H1TzO	10	28	960	n.d.	65.1	186	1.63	252	5333	8405	269	n.d.	n.d.	n.d.	[275]
$Na1TzO\cdot H_2O$ (α)	>40	>380	1080	n.d.	44.5	273	1.81	-366	2792	7555	189	n.d.	n.d.	n.d.	[275]
$Na1TzO\cdot H_2O$ (β)	>40	>380	1080	n.d.	44.5	273	1.78	-364	2817	7428	182	n.d.	n.d.	n.d.	[275]
K1TzO	4	54	63	n.d.	45.1	236	1.88	47	3265	7056	187	n.d.	n.d.	n.d.	[275]
Ag1TzO	<1	1	<0.28	n.d.	29.0	211	3.47	n.d.	n.d.	n.d.	n.d.	n.d.	n.d.	n.d.	[275]
$NaAz2TzO\cdot H_2O$	>40	120	500	n.d.	58.7	n.d.	1.609	n.d.	n.d.	n.d.	n.d.	n.d.	n.d.	n.d.	[276]
KAz2TzO	1.5	<5	1.3	n.d.	59.4	n.d.	2.073	n.d.	n.d.	n.d.	n.d.	n.d.	n.d.	n.d.	[276]
AgAz2TzO	<1	<5	n.d.	n.d.	n.d.	n.d.	n.d.	n.d.	n.d.	n.d.	n.d.	n.d.	n.d.	n.d.	[276]
AgCN2TzO	8	240	50	n.d.	32.1	224	n.d.	n.d.	n.d.	n.d.	n.d.	n.d.	n.d.	n.d.	[277]
$NaCN2TzO\cdot H_2O$	>40	360	1500	<100	46.4	276	1.758	n.d.	n.d.	n.d.	n.d.	n.d.	n.d.	n.d.	[278]
AgCN1TzO	>4	120	80	n.d.	32.1	201	n.d.	n.d.	n.d.	n.d.	n.d.	n.d.	n.d.	n.d.	[277]
$NaNTX\cdot H_2O$	>40	>288	600	n.d.	40.9	220	2.147	n.d.	n.d.	n.d.	n.d.	n.d.	n.d.	n.d.	[279]
KNTX	10	96	600	n.d.	41.4	172	2.148	n.d.	n.d.	n.d.	n.d.	n.d.	n.d.	n.d.	[279]
RbNTX	>6	144	400	n.d.	32.5	206	2.615	n.d.	n.d.	n.d.	n.d.	n.d.	n.d.	n.d.	[279]
CsNTX	3	48	156	n.d.	26.6	188	2.93	n.d.	n.d.	n.d.	n.d.	n.d.	n.d.	n.d.	[279]
$Ca(NTX)_2\cdot 3H_2O$	>40	360	800	n.d.	39.5	180	n.d.	n.d.	n.d.	n.d.	n.d.	n.d.	n.d.	n.d.	[279]
$Sr(NTX)_2\cdot 2H_2O$	30	360	800	n.d.	36.5	220	n.d.	n.d.	n.d.	n.d.	n.d.	n.d.	n.d.	n.d.	[279]
$Ba(NTX)_2\cdot H_2O$	40	360	700	n.d.	33.7	176	2.112	n.d.	n.d.	n.d.	n.d.	n.d.	n.d.	n.d.	[279]
AgNTX	„as RDX”			n.d.	29.4	n.d.	n.d.	n.d.	n.d.	n.d.	n.d.	n.d.	n.d.	n.d.	[279]
K_2NIT	>40	>360	>1500	n.d.	37.8	320	2.228	-280.0	3273	7846	240	n.d.	n.d.	n.d.	[280]
$Li_2BTO1\cdot 4H_2O$	40	324	1000	n.d.	44.1	225	1.765	n.d.	n.d.	n.d.	n.d.	n.d.	n.d.	n.d.	[281]
$Na_2BTO1\cdot 4H_2O$	>40	360	700	n.d.	39.2	330	1.767	n.d.	n.d.	n.d.	n.d.	n.d.	n.d.	n.d.	[281]
K_2BTO1	2	20	700	n.d.	45.5	335	n.d.	n.d.	n.d.	n.d.	n.d.	n.d.	n.d.	n.d.	[281]
Cs_2BTO1	2	60	700	n.d.	25.8	314	3.285	n.d.	n.d.	n.d.	n.d.	n.d.	n.d.	n.d.	[281]
$CaBTO1\cdot 4H_2O$	27	56	200	n.d.	39.9	300	1.953	n.d.	n.d.	n.d.	n.d.	n.d.	n.d.	n.d.	[281]
$SrBTO1\cdot 4H_2O$	30	360	500	n.d.	34.2	300	2.154	n.d.	n.d.	n.d.	n.d.	n.d.	n.d.	n.d.	[281]
$BaBTO1\cdot H_2O$	>40	216	500	n.d.	36.7	290	n.d.	n.d.	n.d.	n.d.	n.d.	n.d.	n.d.	n.d.	[281]
$[MnBTO1\cdot 2H_2O]_n$	16	180	n.d.	n.d.	43.2	313	2.129	n.d.	2548	6412	199	n.d.	n.d.	n.d.	[282]
$[CoBTO1\cdot 2H_2O]_n$	12.5	260	n.d.	n.d.	42.6	282	2.158	n.d.	3159	7164	251	n.d.	n.d.	n.d.	[282]
$[NiBTO1\cdot 2H_2O]_n$	32.5	192	n.d.	n.d.	42.6	274	2.128	n.d.	2623	6769	222	n.d.	n.d.	n.d.	[282]
$[CuBTO1\cdot 2H_2O]_n$	20	216	n.d.	n.d.	41.8	276	2.355	n.d.	3029	7968	325	n.d.	n.d.	n.d.	[282]
$[ZnBTO1\cdot 2H_2O]_n$	60	192	n.d.	n.d.	41.6	318	2.371	n.d.	3234	7635	299	n.d.	n.d.	n.d.	[282]
HANPTz	25	168	n.d.	n.d.	52.3	222	1.85	848	n.d.	9161	383	n.d.	n.d.	n.d.	[286]
AgANPTz	2	60	n.d.	n.d.	36.2	263	2.92	n.d.	n.d.	n.d.	n.d.	n.d.	n.d.	n.d.	[286]
$NaANPTz\cdot 2H_2O$	7	160	n.d.	n.d.	47.9	269	1.93	710.4	n.d.	8430	320	n.d.	n.d.	n.d.	[286]

Table S6. cont.

Compound	*IS* [J]	*FS* [N]	*ESD* [mJ]	Grain size [μm]	N content [%]	T_{dec} [°C]	*d* [g cm^{-3}]	*HOF* [kJ mol^{-1}]	ΔU_{ex} [kJ kg^{-1}]	*DV* [m s^{-1}]	p_{ex} [kbar]	HN	HP	Laser test	Ref.
$KANPTz\cdot 2H_2O$	4	144	n.d.	n.d.	45.2	253	1.98	630.4	n.d.	8380	314	n.d.	n.d.	n.d.	[286]
$H_3BTO1N3\cdot H_2O$	<1	4	n.d.	n.d.	66.7	100	1.80	734	5896	9433	362	n.d.	n.d.	n.d.	[275]
$Li_3BTO1N3\cdot 6H_2O$	>40	>360	n.d.	n.d.	45.5	311	1.67	-1505	3568	7911	213	n.d.	n.d.	n.d.	[275]
$Na_3BTO1N3\cdot 6H_2O$	>40	>360	n.d.	n.d.	39.8	339	1.71	-1480	3003	7352	173	n.d.	n.d.	n.d.	[275]
$K_3BTO1N3\cdot 3H_2O$	>40	>360	n.d.	n.d.	40.4	294	1.97	-1475	1239	6546	143	n.d.	n.d.	n.d.	[275]
$Rb_3BTO1N3\cdot 3H_2O$	>40	288	n.d.	n.d.	29.6	292	2.55	n.d.	n.d.	n.d.	n.d.	n.d.	n.d.	n.d.	[275]
$[Cu(BTO1N3)(NH_3)]$	11	288	n.d.	n.d.	48.6	205	1.96	-53	3173	7725	249	n.d.	n.d.	n.d.	[275]
$H_2BTO1DA$	<1	<5	10	n.d.	70.7	170	1.902	883.2	6648	9548	424	n.d.	n.d.	n.d.	[283]
$K_2BTO1DA$	20	>360	350	n.d.	51.1	285	2.200	240.2	n.d.	9753	410	n.d.	n.d.	n.d.	[283]
$[CoBTO1DA(H_2O)_4\cdot 2H_2O]_n$	>40	>360	n.d.	n.d.	35.6	195	2.021	n.d.	5920	8556	347	n.d.	n.d.	n.d.	[284]
$[CdBTO1DA(H_2O)_4\cdot 2H_2O]_n$	>40	>360	n.d.	n.d.	33.6	224	2.178	n.d.	6556	8573	363	n.d.	n.d.	n.d.	[284]
$[NiBTO1DA(H_2O)_4\cdot 2H_2O]_n$	37.5	324	n.d.	n.d.	38.6	192	2.049	n.d.	6004	8591	353	n.d.	n.d.	n.d.	[284]
$[CuBTO1DA(H_2O)_3]_n$	35	288	n.d.	n.d.	44.6	174	2.079	n.d.	8711	9344	420	n.d.	n.d.	n.d.	[284]
$[Mg(H_2O)_6]BTO1DA\cdot 2H_2O$	39.2	>360	n.d.	n.d.	38.4	296	1.73	-777.8	5289	n.d.	n.d.	n.d.	n.d.	n.d.	[285]
$H_2MCABTz$	1	14	n.d.	n.d.	49.2	191	1.64	325.5	3905	7403	201	n.d.	n.d.	n.d.	[269]
$Na_2MCABTz$	40	360	n.d.	n.d.	42.6	102	1.65	232.8	4762	7041	175	n.d.	n.d.	n.d.	[269]
$H1MTzN3\cdot H_2O$	3	360	n.d.	n.d.	67.8	182	1.528	514	3934	7825	209	n.d.	n.d.	n.d.	[270]
H2MTzN3	2.5	70	n.d.	n.d.	73.7	183	1.532	792	4450	7982	221	n.d.	n.d.	n.d.	[270]
Na2MTzN3	n.d.	n.d.	n.d.	n.d.	61.8	195	1.327	n.d.	n.d.	n.d.	n.d.	n.d.	n.d.	n.d.	[270]
$Na1MTzN3\cdot 5H_2O$	n.d.	n.d.	n.d.	n.d.	48.0	256	n.d.	n.d.	n.d.	n.d.	n.d.	n.d.	n.d.	n.d.	[270]
$H5NTzMTz\cdot H_2O$	<8	120-360	n.d.	n.d.	58.6	214	1.796	644	10192	8341	287	n.d.	n.d.	n.d.	[572]
H5NTzMTz	<2	<180	n.d.	n.d.	64.0	214	1.802	1414	10954	8688	302	n.d.	n.d.	n.d.	[572]
$Li5NTzMTz\cdot 1.5H_2O$	<4	<5	n.d.	n.d.	54.8	180	n.d.	n.d.	n.d.	n.d.	n.d.	n.d.	n.d.	n.d.	[271]
$Na5NTzMTz\cdot 2H_2O$	<6	<12	n.d.	n.d.	49.4	192	n.d.	n.d.	n.d.	n.d.	n.d.	n.d.	n.d.	n.d.	[271]
$K5NTzMTz\cdot H_2O$	<8	<30	n.d.	n.d.	49.8	184	n.d.	n.d.	n.d.	n.d.	n.d.	n.d.	n.d.	n.d.	[271]
$Rb5NTzMTz\cdot H_2O$	<6	<5	n.d.	n.d.	42.1	170	n.d.	n.d.	n.d.	n.d.	n.d.	n.d.	n.d.	n.d.	[271]
$Cs5NTzMTz\cdot H_2O$	<15	<60	n.d.	n.d.	36.3	186	n.d.	n.d.	n.d.	n.d.	n.d.	n.d.	n.d.	n.d.	[271]
$Ag5NTzMTz\cdot 0.5H_2O$	<3.5	<5	n.d.	n.d.	40.3	193	n.d.	n.d.	n.d.	n.d.	n.d.	n.d.	n.d.	n.d.	[271]
$Cu(5NTzMTz)_2\cdot H_2O$	<7.5	<80	n.d.	n.d.	53.2	250	n.d.	n.d.	n.d.	n.d.	n.d.	n.d.	n.d.	n.d.	[271]
$H_2NA15DTM$	12	160	n.d.	n.d.	66.0	178	1.76	615.9	8652	296	n.d.	n.d.	n.d.	n.d.	[272]
$K_2NA15DTM$	>40	>360	n.d.	n.d.	48.6	306	1.94	119.5	n.d.	7174	193	n.d.	n.d.	n.d.	[272]
$NaH4ATTz\cdot 2H_2O$	40	360	300	500-1000	49.6	330	1.733	n.d.	n.d.	n.d.	n.d.	n.d.	n.d.	n.d.	[287]
H25PNTz	2	80	n.d.	n.d.	39.0	73	1.88	284.3	8511	312	n.d.	n.d.	n.d.	n.d.	[290]
K25PNTz	1	40	n.d.	n.d.	34.9	118	1.98	108.4	7910	276	n.d.	n.d.	n.d.	n.d.	[290]
$H_225TNTz$	3	120	n.d.	n.d.	40.3	88	1.92	274.5	9123	377	n.d.	n.d.	n.d.	n.d.	[290]

Table S6. cont.

Compound	*IS* [J]	*FS* [N]	*ESD* [mJ]	Grain size [µm]	N content [%]	T_{dec} [°C]	*d* [g cm^{-3}]	*HOF* [kJ mol^{-1}]	ΔU_{ex} [kJ kg^{-1}]	*DV* [m s^{-1}]	p_{ex} [kbar]	HN	HP	Laser test	Ref.
$K_2$5TNTz	4	80	n.d.	n.d.	31.6	128	2.05	-347.2	7394	239	n.d.	n.d.	n.d.	n.d.	[290]
H_2DNTz	5	64	n.d.	n.d.	48.3	92	1.636	n.d.	n.d.	n.d.	n.d.	n.d.	n.d.	n.d.	[292]
	3	28	n.d.	>500	48.3	88	1.871	669.8	6447	9594	403	n.d.	n.d.	n.d.	[293]
H_2DNTz·2H_2O	15	168	n.d.	n.d.	40.0	103	1.738	n.d.	n.d.	n.d.	n.d.	n.d.	n.d.	n.d.	[292]
KH5DNTz	3.5	10	n.d.	n.d.	39.6	155	2.153	n.d.	n.d.	n.d.	n.d.	n.d.	n.d.	n.d.	[292]
$K_2$5DNTz	4	160	100	100-500	33.6	291	2.157	n.d.	n.d.	n.d.	n.d.	n.d.	n.d.	n.d.	[291]
NaHDNTz·3H_2O	<1	14	60	250-500	33.6	n.d.	1.835	n.d.	n.d.	n.d.	n.d.	n.d.	n.d.	n.d.	[293]
Na_2DNTz·2H_2O	<1	324	160	>1000	33.1	134	1.896	n.d.	n.d.	n.d.	n.d.	n.d.	n.d.	n.d.	[293]
Sr(HDNTz)$_2$	7	160	130	160-500	38.8	n.d.	n.d.	n.d.	n.d.	n.d.	n.d.	n.d.	n.d.	n.d.	[293]
SrDNTz	7	160	130	160-250	32.4	n.d.	n.d.	n.d.	n.d.	n.d.	n.d.	n.d.	n.d.	n.d.	[293]
H5TNTz	9	9	n.d.	n.d.	44.8	101	1.918	n.d.	n.d.	n.d.	n.d.	n.d.	n.d.	n.d.	[292]
Rb5TNTz	0.5	<1	n.d.	n.d.	32.3	120	2.379	n.d.	n.d.	n.d.	n.d.	n.d.	n.d.	n.d.	[292]
Cs5TNTz	0.5	<1	n.d.	n.d.	27.9	130	2.568	n.d.	n.d.	n.d.	n.d.	n.d.	n.d.	n.d.	[292]
Ag5TNTz	5	5	n.d.	n.d.	30.1	123	n.d.	n.d.	n.d.	n.d.	n.d.	n.d.	n.d.	n.d.	[292]
{[Ag(NH_3)$_2$]$_2$[Ag_2(5TNTz)$_3$]}$_2$	9.5	5	n.d.	n.d.	31.8	123	2.411	n.d.	n.d.	n.d.	n.d.	n.d.	n.d.	n.d.	[292]
Cu(5TNTz)$_2$·xH_2O	2.5	80	n.d.	n.d.	n.d.	123	n.d.	n.d.	n.d.	n.d.	n.d.	n.d.	n.d.	n.d.	[292]
[Cu(NH_3)$_3$(5TNTz)$_2$]	2.5	95	n.d.	n.d.	43.2	117	1.975	n.d.	n.d.	n.d.	n.d.	n.d.	n.d.	n.d.	[292]
H_2DNPPTz	15	192	n.d.	n.d.	47.8	281	1.79	1111.5	n.d.	8721	309	n.d.	n.d.	n.d.	[288]
K_2DNPPTz	25	252	n.d.	n.d.	58.7	329	2.00	638.9	n.d.	8807	258	n.d.	n.d.	n.d.	[288]
HDNDEt2NATz	8	80	n.d.	n.d.	51.3	131	1.81	812.5	n.d.	8748	327	n.d.	n.d.	n.d.	[294]
NaDNPEt2NATz	10	80	n.d.	n.d.	48.3	135	1.83	752.1	n.d.	8164	286	n.d.	n.d.	n.d.	[294]
H_2DNABTzEO·3H_2O	n.d.	n.d.	n.d.	n.d.	45.2	143	n.d.	n.d.	n.d.	n.d.	n.d.	detonate on heating			[273]
H_2DNABTzEO	n.d.	n.d.	n.d.	n.d.	52.8	125	n.d.	n.d.	n.d.	n.d.	n.d.	detonate on heating			[273]
Ag_2DNABTzEO	n.d.	n.d.	n.d.	n.d.	31.6	200	n.d.	n.d.	n.d.	n.d.	n.d.	detonate on heating			[273]
PbCu(BTzA)$_2$	32.7	n.d.	n.d.	n.d.	44.0	231	n.d.	n.d.	n.d.	n.d.	n.d.	n.d.	n.d.	n.d.	[261]
[Co_9(BTzA)$_{10}$(HBTzA)$_2$(H_2O)$_{10}$]$_n$	27	>360	n.d.	n.d.	60.0	253	n.d.	n.d.	n.d.	n.d.	n.d.	n.d.	n.d.	n.d.	[263]
H_2AzTTz	<1	20	50	<100	78.4	164	1.66	777	4578	7874	241	n.d.	n.d.	n.d.	[573]
AgHAzTTz	1	5	14	<100	49.2	194	n.d.	n.d.	n.d.	n.d.	n.d.	det.	det.	n.d.	[296]
CuHAzTTz	40	360	1000	<100	58.2	293	n.d.	n.d.	n.d.	n.d.	n.d.	def.	n.d.	n.d.	[296]
KHAzTTz	1	5	5	<100	64.8	172	n.d.	n.d.	n.d.	n.d.	n.d.	det.	det.	n.d.	[296]
CsHAzTTz·H_2O	1	15	20	<100	42.7	200	n.d.	n.d.	n.d.	n.d.	n.d.	det.	det.	n.d.	[296]
K_2AzTTz	1	14	10	<100	55.1	173	n.d.	n.d.	n.d.	n.d.	n.d.	det.	det.	n.d.	[296]
Cs_2AzTTz	1	16	5	<100	31.1	162	n.d.	n.d.	n.d.	n.d.	n.d.	det.	det.	n.d.	[296]
CuAzTTz	2	5	15	<100	58.2	164	n.d.	n.d.	n.d.	n.d.	n.d.	det.	det.	n.d.	[296]
H_2AzTTzO	4	120	>260	<100	66.0	144	1.69	795	5360	8239	275	n.d.	n.d.	n.d.	[574]
AgHAzTTzO	1	5	5	<100	49.5	198	n.d.	n.d.	n.d.	n.d.	n.d.	n.d.	n.d.	n.d.	[295]

Table S6. cont.

Compound	*IS* [J]	*FS* [N]	*ESD* [mJ]	Grain size [µm]	N content [%]	T_{dec} [°C]	*d* [g cm^{-3}]	*HOF* [kJ mol^{-1}]	ΔU_{ex} [kJ kg^{-1}]	*DV* [m s^{-1}]	p_{ex} [kbar]	HN	HP	Laser test	Ref.
CuHAzTTzO	40	360	800	<100	51.9	293	n.d.	n.d.	n.d.	n.d.	n.d.	n.d.	n.d.	n.d.	[295]
KHAzTTzO	1	6	15	<100	60.3	221	n.d.	n.d.	n.d.	n.d.	n.d.	n.d.	n.d.	n.d.	[295]
CsHAzTTzO	1	10	10	<100	43.0	205	n.d.	n.d.	n.d.	n.d.	n.d.	n.d.	n.d.	n.d.	[295]
K_2AzTTzO	n.d.	n.d.	n.d.	n.d.	49.2	178	n.d.	n.d.	n.d.	n.d.	n.d.	n.d.	n.d.	n.d.	[295]
CuAzTTzO	5	60	300	<100	48.0	168	n.d.	n.d.	n.d.	n.d.	n.d.	n.d.	n.d.	n.d.	[295]
[Cu(ATTz)$_2$(H_2O)$_2$]·2H_2O	40	360	1500	100-500	47.7	264	n.d.	n.d.	n.d.	n.d.	n.d.	n.d.	n.d.	n.d.	[289]
K_2NTrTz	35	360	1000	n.d.	43.4	361	2.17	288.8	4694	8238	274	n.d.	n.d.	n.d.	[176]
[Pb(HTTz)$_2$(H_2O)]$_n$	>40	>360	n.d.	n.d.	39.4	340	2.519	665.5	5686	7715	316	n.d.	n.d.	n.d.	[298]
[Pb(H_2TTz)O]$_n$	>40	>360	n.d.	n.d.	27.2	318	3.511	889.6	1067	8122	401	n.d.	n.d.	n.d.	[298]
[Cu(TTz)]$_n$	>40	>360	n.d.	n.d.	49.4	360	2.216	2868.8	14213	8429	400	n.d.	n.d.	n.d.	[297]
{[Cu(TTz)](H_2O)}$_n$	>40	>360	n.d.	n.d.	45.2	325	2.316	n.d.	5531	7920	320	n.d.	n.d.	n.d.	[297]
	>40	>360	>24.75	n.d.	45.2	325	2.308	n.d.	5531	7920	320	n.d.	n.d.	n.d.	[299]
[Cu(HTTz)$_2$(H_2O)$_2$]$_n$	>40	>360	>24.75	n.d.	52.7	345	1.885	n.d.	8904	8180	306	n.d.	n.d.	n.d.	[299]
[Cu(HTTz)]$_n$	32	>360	>24.75	n.d.	49.1	355	2.419	n.d.	16561	10400	565	n.d.	n.d.	n.d.	[299]
[Na(BTzTzA)$_2$(gua)$_5$]$_n$	>40	>360	n.d.	n.d.	76.6	221	1.625	n.d.	n.d.	n.d.	n.d.	n.d.	n.d.	n.d.	[301]
[Cu(HDTzIm)(H_2O)]$_n$	>40	>360	n.d.	n.d.	49.5	262	1.851	n.d.	6464	7183	233	n.d.	n.d.	n.d.	[371]
[Cu(HDTzIm)]$_n$	>40	>360	n.d.	n.d.	34.2	325	3.186	244.8	4845	7842	359	n.d.	n.d.	n.d.	[372]
[ZnI(I3PTz)(CH_3OH)]$_n$	80.3	>353	>250	n.d.	11.2	275	3.32	-693.9	n.d.	n.d.	n.d.	n.d.	n.d.	n.d.	[300]
[Cu(1MeTr)$_2$(CNBTz)$_2$]$_n$	>40	>360	n.d.	n.d.	50.3	203	1.560	-10083	n.d.	n.d.	n.d.	n.d.	n.d.	n.d.	[302]
**OTHER COMPOUNDS**															
[Cu(TrDA)(IO_3)$_2$]$_n$	18	60	n.d.	n.d.	19.4	267	3.11	1181	6059	7271	406	n.d.	n.d.	n.d.	[366]
KDNATr	1	20	20	n.d.	43.2	210	1.922	256.1	9736	8823	367	n.d.	n.d.	n.d.	[458]
AgDNATr	2	5	20	n.d.	33.1	238	2.682	n.d.	n.d.	n.d.	n.d.	n.d.	n.d.	n.d.	[458]
HNCAz	<1	0.5	n.d	n.d.	53.4	83	1.708	147.5	5066	8333	270	n.d.	n.d.	n.d.	[432]
NaNCAz·H_2O	<1	0.4	65	n.d.	40.9	81	1.891	-350.6	n.d.	n.d.	n.d.	n.d.	n.d.	n.d.	[432]
KNCAz	<1	<0.1	10	n.d.	41.4	120	1.986	-63.4	4227	7441	223	n.d.	n.d.	n.d.	[432]
[Co(ANQ)$_2$$Cl_2$]·2$H_2O$	10	360	700	100-500	34.7	186	1.927	n.d.	n.d.	n.d.	n.d.	n.d.	n.d.	-	[368]
[Ni(ANQ)$_2$$Cl_2$]·2$H_2O$	10	360	700	100-500	34.7	250	1.950	n.d.	n.d.	n.d.	n.d.	n.d.	n.d.	-	[368]
[Zn(ANQ)$_2$$Cl_2$]·2$H_2O$	10	360	700	100-500	34.1	172	1.944	n.d.	n.d.	n.d.	n.d.	n.d.	n.d.	-	[368]
[Co(ANQ)$_2$(H_2O)$_2$](ADN)$_2$·2H_2O	5	80	700	100-500	38.6	118	1.964	n.d.	n.d.	n.d.	n.d.	n.d.	n.d.	-	[368]
[Ni(ANQ)$_2$(H_2O)$_2$](ADN)$_2$·2H_2O	3	80	600	100-500	38.6	142	1.982	n.d.	n.d.	n.d.	n.d.	n.d.	n.d.	-	[368]
[Ag(ANQ)(ADN)]·H_2O	2	7	100	n.d.	31.9	108	2.456	n.d.	n.d.	n.d.	n.d.	n.d.	n.d.	-	[368]
[Cu_2(4ATr)$_6$](BrO_3)$_4$·H_2O	2	<5	25	<100	29.0	137	2.299	n.d.	n.d.	n.d.	n.d.	n.d.	n.d.	det.	[413]
[Cu(BrO_3)$_2$(1MTz)$_2$]	1	<5	60	100-500	23.0	169	2.491	n.d.	n.d.	n.d.	n.d.	n.d.	n.d.	det.	[413]
[Cu(BrO_3)$_2$(2M5ATz)$_4$]·H_2O	2	<5	90	100-500	31.2	92	1.983	n.d.	n.d.	n.d.	n.d.	n.d.	n.d.	det.	[413]
[Cu(BrO_3)$_2$(12DTP)$_2$]	<1	10	150	100-500	32.9	146	2.142	n.d.	n.d.	n.d.	n.d.	n.d.	n.d.	det.	[413]

Table S6. cont.

Compound	*IS* [J]	*FS* [N]	*ESD* [mJ]	Grain size [µm]	N content [%]	T_{dec} [°C]	*d* [g cm^{-3}]	*HOF* [kJ mol^{-1}]	ΔU_{ex} [kJ kg^{-1}]	*DV* [m s^{-1}]	p_{ex} [kbar]	HN	HP	Laser test	Ref.
$[Cu(BrO_3)_2(22DTP)_2]\cdot4H_2O$	8	108	260	100-500	29.8	150	2.002	n.d.	n.d.	n.d.	n.d.	n.d.	n.d.	dec.	[413]
$[Cu(H_2O)_2(11BTziP)_2](BrO_3)_2$	1	40	150	100-500	31.3	129	1.989	n.d.	n.d.	n.d.	n.d.	n.d.	n.d.	det.	[413]
$K_2BDNMTr$	1	60	n.d.	n.d.	27.8	131	2.04	-135	n.d.	8715	283	n.d.	n.d.	n.d.	[459]
$[Co(NH_3)_6](MnO_4)_3$	*1.4*	n.d.	n.d.	n.d.	16.2	n.d.	n.d.	n.d.	n.d.	n.d.	n.d.	n.d.	n.d.	n.d.	[351]
trans-$[Co(en)_2Cl_2]MnO_4$	*3*	n.d.	n.d.	n.d.	15.4	n.d.	n.d.	n.d.	n.d.	n.d.	n.d.	n.d.	n.d.	n.d.	[351]
$[Co(NH_3)_6](BrO_3)_3$	*2.4*	n.d.	n.d.	n.d.	15.4	n.d.	n.d.	n.d.	n.d.	n.d.	n.d.	n.d.	n.d.	n.d.	[351]
$[Co(en)_3](BrO_3)_3$	*3*	n.d.	n.d.	n.d.	13.5	n.d.	n.d.	n.d.	n.d.	n.d.	n.d.	n.d.	n.d.	n.d.	[351]
trans-$[Co(en)_2Cl_2]BrO_3$	*3.2*	n.d.	n.d.	n.d.	14.8	n.d.	n.d.	n.d.	n.d.	n.d.	n.d.	n.d.	n.d.	n.d.	[351]
$[Co(NH_3)_6](IO_3)_3$	*8.6*	n.d.	n.d.	n.d.	12.3	n.d.	n.d.	n.d.	n.d.	n.d.	n.d.	n.d.	n.d.	n.d.	[351]
	20	n.d.	n.d.	n.d.		355	n.d.	n.d.	n.d.	n.d.	n.d.	n.d.	n.d.	n.d.	[352]
$[Co(NH_3)_6][Co(NO_2)_6]$	*17*	n.d.	n.d.	n.d.	33.9	-	n.d.	n.d.	n.d.	n.d.	n.d.	n.d.	n.d.	n.d.	[352]
$[Co(NO_2)_3(NH_3)_3]$	*8.8*	n.d.	n.d.	n.d.	33.9	305	n.d.	n.d.	n.d.	n.d.	n.d.	n.d.	n.d.	n.d.	[352]
$(NH_4)_3[Co(NO_2)_6]$	*6.6*	n.d.	n.d.	n.d.	32.4	230	n.d.	n.d.	n.d.	n.d.	n.d.	n.d.	n.d.	n.d.	[352]
$[Co(NH_3)_6](NO_2)_3$	*8.2*	n.d.	n.d.	n.d.	42.1	n.d.	n.d.	n.d.	n.d.	n.d.	n.d.	n.d.	n.d.	n.d.	[351]
$[Co(NH_3)_5Cl](NO_2)_2$	*28.2*	n.d.	n.d.	n.d.	36.1	n.d.	n.d.	n.d.	n.d.	n.d.	n.d.	n.d.	n.d.	n.d.	[351]
KCPT	7.5	240	n.d.	n.d.	39.0	323	1.98	529.4	5965	8457	325	n.d.	n.d.	n.d.	[445]
$K_2DNMOxon$	3	120	100	50-100	21.0	219	2.16	n.d.	n.d.	n.d.	n.d.	n.d.	n.d.	n.d.	[480]
BaDNMOxon	10	160	100	50-100	17.2	145	2.07	n.d.	n.d.	n.d.	n.d.	n.d.	n.d.	n.d.	[480]
KDNMMOx	4	120	750	50-100	17.3	165	1.92	n.d.	n.d.	n.d.	n.d.	n.d.	n.d.	n.d.	[480]
K_2NPA	1.5	60	800	n.d.	33.5	315	2.15	501.1	5035	8275	309	n.d.	n.d.	n.d.	[447]
$H_2BANATr$	9	120	150	n.d.	58.7	259	1.756	691.9	5520	8846	312	n.d.	n.d.	n.d.	[575]
$Li_2BANATr\cdot3H_2O$	35	360	1500	n.d.	47.7	240	n.d.	n.d.	n.d.	n.d.	n.d.	n.d.	n.d.	n.d.	[460]
$Na_2BANATr\cdot4H_2O$	40	360	n.d.	<100	41.8	287	1.716	n.d.	n.d.	n.d.	n.d.	n.d.	n.d.	n.d.	[462]
$K_2BANATr$	3	360	n.d.	<100	46.4	250	2.001	n.d.	n.d.	n.d.	n.d.	n.d.	n.d.	n.d.	[462]
	4	40	n.d.	n.d.		220	1.98	531.6	n.d.	7827	258	n.d.	n.d.	n.d.	[461]
$Rb_2BANATr$	10	360	n.d.	<100	36.9	150	2.424	n.d.	n.d.	n.d.	n.d.	n.d.	n.d.	n.d.	[462]
$Cs_2BANATr$	8	360	n.d.	<100	30.7	220	2.670	n.d.	n.d.	n.d.	n.d.	n.d.	n.d.	n.d.	[462]
$CaBANATr\cdot5H_2O$	40	360	n.d.	<100	40.6	150	1.737	n.d.	n.d.	n.d.	n.d.	n.d.	n.d.	n.d.	[462]
$SrBANATr\cdot6H_2O$	40	360	n.d.	<100	35.0	160	1.908	n.d.	n.d.	n.d.	n.d.	n.d.	n.d.	n.d.	[462]
$BaBANATr\cdot2H_2O$	40	360	n.d.	<100	36.4	280	n.d.	n.d.	n.d.	n.d.	n.d.	n.d.	n.d.	n.d.	[462]
$Ag_2BDNMOx$	2	60	70	n.d.	20.0	273	3.01	n.d.	n.d.	n.d.	n.d.	n.d.	n.d.	n.d.	[481]
$Ag_2BDNMOxC$	25	120	n.d.	n.d.	19.5	133	n.d.	n.d.	n.d.	n.d.	n.d.	n.d.	n.d.	n.d.	[482]
$Cu(DCA)_2$	40	>360	630	<100	43.0	254	n.d.	n.d.	n.d.	n.d.	n.d.	n.d.	n.d.	n.d.	[438]
$[Cu(DCA)_2(1MTz)_2]_n$	8	>360	>1500	<100	53.9	170	1.735	n.d.	n.d.	n.d.	n.d.	n.d.	n.d.	n.d.	[438]
$[Cu(DCA)_2(AzEtTz)_2]_n$	6	192	1500	<100	59.1	131	1.772	n.d.	n.d.	n.d.	n.d.	n.d.	n.d.	n.d.	[438]
$[Cu(DCA)_2(1ATz)_2]_n$	6	144	1350	<100	61.3	141	1.860	n.d.	n.d.	n.d.	n.d.	n.d.	n.d.	n.d.	[438]

Table S6. cont.

Compound	*IS* [J]	*FS* [N]	*ESD* [mJ]	Grain size [µm]	N content [%]	T_{dec} [°C]	*d* [g cm^{-3}]	*HOF* [kJ mol^{-1}]	ΔU_{ex} [kJ kg^{-1}]	*DV* [m s^{-1}]	p_{ex} [kbar]	HN	HP	Laser test	Ref.
$[Cu(DCA)_2(2ATz)_2]_n$ α	9	80	540	<100	61.3	129	1.858	n.d.	n.d.	n.d.	n.d.	n.d.	n.d.	n.d.	[438]
$[Cu(DCA)_2(2ATz)_2]_n$ β	9	80	540	<100	61.3	126	1.757	n.d.	n.d.	n.d.	n.d.	n.d.	n.d.	n.d.	[438]
$[Cu(DCA)_2(11DTE)_2]_n$ α	10	>360	1220	<100	54.2	126	1.817	n.d.	n.d.	n.d.	n.d.	n.d.	n.d.	n.d.	[438]
$K34DNPO{\cdot}H_2O$	5	216	700	100-500	24.3	197	1.955	-391.7	4636	7920	269	n.d.	n.d.	n.d.	[450]
$K35DNPO{\cdot}0.5H_2O$	6	240	n.d.	100-500	33.5	229	n.d.	n.d.	n.d.	n.d.	n.d.	n.d.	n.d.	n.d.	[450]
AgAzCNTr	2	16	18	<100	40.5	186	n.d.	n.d.	n.d.	n.d.	n.d.	n.d.	n.d.	n.d.	[465]
AgAzCOATr	3	24	10	<100	40.8	178	n.d.	n.d.	n.d.	n.d.	n.d.	n.d.	n.d.	n.d.	[465]
$NaAzNTr{\cdot}H_2O$	3	144	300	<100	50.3	177	n.d.	n.d.	n.d.	n.d.	n.d.	n.d.	n.d.	n.d.	[466]
KAzNTr	<1	<5	15	<100	50.8	175	1.933	n.d.	n.d.	n.d.	n.d.	n.d.	det.	n.d.	[466]
CsAzNTr	<1	<5	7	<100	34.2	199	n.d.	n.d.	n.d.	n.d.	n.d.	n.d.	det.	n.d.	[466]
AgAzNTr	<1	<5	3	<100	37.4	171	n.d.	n.d.	n.d.	n.d.	n.d.	n.d.	det.	n.d.	[466]
$Pb(AzNTr)_2$	<1	8	n.d.	<100	36.8	149	2.768	n.d.	n.d.	n.d.	n.d.	n.d.	det.	n.d.	[466]
$K_2BNATrNAC3{\cdot}2H_2O$	20	>360	n.d.	100-500	36.8	207	n.d.	n.d.	n.d.	n.d.	n.d.	n.d.	n.d.	n.d.	[472]
$Na_2BNATrNAC3{\cdot}2H_2O$	40	>360	n.d.	100-500	39.6	193	n.d.	n.d.	n.d.	n.d.	n.d.	n.d.	n.d.	n.d.	[472]
$K_2BNATrOC3{\cdot}H_2O$	15	>360	n.d.	300-1000	35.5	195	n.d.	n.d.	n.d.	n.d.	n.d.	n.d.	n.d.	n.d.	[472]
$Na_2BNATrOC3{\cdot}H_2O$	40	>360	n.d.	300-1000	38.7	208	n.d.	n.d.	n.d.	n.d.	n.d.	n.d.	n.d.	n.d.	[472]
$K_2BNATrHC1$	10	>360	n.d.	500-1000	44.9	205	1.93	106	n.d.	7271	202	n.d.	n.d.	n.d.	[472]
$Li34DNIm{\cdot}3H_2O$	>40	>360	250	100-500	25.7	231	1.634	n.d.	n.d.	n.d.	n.d.	n.d.	n.d.	n.d.	[441]
$Na34DNIm{\cdot}H_2O$	25	>360	600	100-500	28.3	235	1.918	n.d.	n.d.	n.d.	n.d.	n.d.	n.d.	n.d.	[441]
$K34DNIm{\cdot}H_2O$	>40	288	600	100-500	26.2	253	1.852	n.d.	n.d.	n.d.	n.d.	n.d.	n.d.	n.d.	[441]
$Ca(34DNIm)_2{\cdot}3H_2O$	3	360	100	100-500	27.5	268	1.873	n.d.	n.d.	n.d.	n.d.	n.d.	n.d.	n.d.	[441]
$Sr(34DNIm)_2{\cdot}5H_2O$	>40	>360	200	500-1000	22.8	261	1.960	n.d.	n.d.	n.d.	n.d.	n.d.	n.d.	n.d.	[441]
$Ba(34DNIm)_2{\cdot}4H_2O$	40	324	170	500-1000	21.4	239	2.220	n.d.	n.d.	n.d.	n.d.	n.d.	n.d.	n.d.	[441]
$Li35DNP{\cdot}3H_2O$	>40	360	700	<100	25.7	325	n.d.	n.d.	n.d.	n.d.	n.d.	n.d.	n.d.	n.d.	[444]
$Na35DNP{\cdot}2H_2O$	>40	360	500	<100	25.9	324	n.d.	n.d.	n.d.	n.d.	n.d.	n.d.	n.d.	n.d.	[444]
K35DNP	20	216	100	100-500	28.6	306	n.d.	n.d.	n.d.	n.d.	n.d.	n.d.	n.d.	n.d.	[444]
$Ca(35DNP)_2{\cdot}4H_2O$	8	360	500	100-500	26.3	>400	n.d.	n.d.	n.d.	n.d.	n.d.	n.d.	n.d.	n.d.	[444]
$Sr(35DNP)_2{\cdot}6H_2O$	>40	360	200	100-500	22.0	>400	n.d.	n.d.	n.d.	n.d.	n.d.	n.d.	n.d.	n.d.	[444]
$Ba(35DNP)_2{\cdot}H_2O$	30	72	100	500-1000	23.8	361	1.863	n.d.	n.d.	n.d.	n.d.	n.d.	n.d.	n.d.	[444]
$LiTNP{\cdot}4H_2O$	40	96	200	100-500	24.9	274	n.d.	n.d.	n.d.	n.d.	n.d.	n.d.	n.d.	n.d.	[444]
NaTNP	25	80	200	100-500	31.1	254	n.d.	n.d.	n.d.	n.d.	n.d.	n.d.	n.d.	n.d.	[444]
$Sr(TNP)_2{\cdot}3H_2O$	40	80	200	100-500	25.7	193	2.103	n.d.	n.d.	n.d.	n.d.	n.d.	n.d.	n.d.	[444]
$Ba(TNP)_2{\cdot}3H_2O$	5	144	100	100-500	23.5	302	2.162	n.d.	n.d.	n.d.	n.d.	n.d.	n.d.	n.d.	[444]
$LiTNIm{\cdot}3H_2O$	>40	>360	500	<100	26.6	252	1.740	n.d.	n.d.	n.d.	n.d.	n.d.	n.d.	n.d.	[444]
NaTNIm	>40	>360	600	<100	31.1	249	n.d.	n.d.	n.d.	n.d.	n.d.	n.d.	n.d.	n.d.	[444]
KTNIm	25	120	200	100-500	29.0	231	n.d.	n.d.	n.d.	n.d.	n.d.	n.d.	n.d.	n.d.	[444]

Table S6. cont.

Compound	*IS* [J]	*FS* [N]	*ESD* [mJ]	Grain size [μm]	N content [%]	T_{dec} [°C]	*d* [g cm^{-3}]	*HOF* [kJ mol^{-1}]	ΔU_{ex} [kJ kg^{-1}]	*DV* [m s^{-1}]	p_{ex} [kbar]	HN	HP	Laser test	Ref.
$Ca(TNIm)_2 \cdot 2H_2O$	>40	>360	700	100-500	29.2	188	n.d.	n.d.	n.d.	n.d.	n.d.	n.d.	n.d.	n.d.	[444]
$Sr(TNIm)_2 \cdot H_2O$	>40	>360	700	<100	27.5	239	n.d.	n.d.	n.d.	n.d.	n.d.	n.d.	n.d.	n.d.	[444]
$Ba(TNIm)_2 \cdot 2H_2O$	>40	>360	600	100-500	24.3	166	n.d.	n.d.	n.d.	n.d.	n.d.	n.d.	n.d.	n.d.	[444]
$Cu(TNIm)_2 \cdot 4H_2O$	3	80	100	100-500	26.0	170	n.d.	n.d.	n.d.	n.d.	n.d.	n.d.	n.d.	n.d.	[444]
NaNDNMP	9	120	n.d.	n.d.	28.8	203	1.87	-55.7	n.d.	8256	286	n.d.	n.d.	n.d.	[446]
KNDNMP	4	36	n.d.	n.d.	27.4	171	2.01	28.2	n.d.	8132	295	n.d.	n.d.	n.d.	[446]
$[Na_2(DNGTt)(H_2O)_6]_n$	>24	n.d.	n.d.	n.d.	38.4	250	1.724	-1069.6	3892	7954	227	n.d.	n.d.	n.d.	[489]
$[Na_2(DNGTt)(H_2O)_2]_n$	>24	n.d.	n.d.	n.d.	45.9	262	1.947	-242	4293	8808	289	n.d.	n.d.	n.d.	[489]
$K_2DNDNAPr$	4	40	n.d.	n.d.	30.6	220	2.10	-106.7	n.d.	9157	368	det.	def.	n.d.	[498]
$K_2BNA134Ox$	5	288	750	<100	33.5	260	2.04	-382.7	3886	7567	233	n.d.	n.d.	n.d.	[486]
KADNM134Ox	10	80	n.d.	n.d.	30.1	235	2.05	-203.2	n.d.	9035	330	n.d.	n.d.	n.d.	[485]
$KDNBP \cdot H_2O$	30	>360	750	100-500	30.0	299	1.77	-224.3	3549	6498	169	n.d.	n.d.	n.d.	[451]
$CsDNBP \cdot H_2O$	30	>360	840	100-500	22.5	341	2.21	n.d.	n.d.	n.d.	n.d.	n.d.	n.d.	n.d.	[451]
$KTNBP \cdot H_2O$	7	324	100	100-500	30.2	333	1.84	-197.6	4258	7384	221	n.d.	n.d.	n.d.	[451]
$K_2QNBP \cdot H_2O$	1.5	120	160	100-500	28.7	n.d.	n.d.	n.d.	n.d.	n.d.	n.d.	n.d.	n.d.	n.d.	[452]
$Na_2DAMA \cdot 0.5H_2O$	>40	>360	1000	100-500	33.9	126	1.86	-786	3167	6589	148	n.d.	n.d.	n.d.	[440]
K_2DAMA	5	288	160	500-1000	32.0	105	1.95	-455	2471	7275	190	n.d.	n.d.	n.d.	[440]
$K_2BNNATr$	2	40	n.d.	n.d.	32.9	287	1.005	1.0	n.d.	8989	322	n.d.	n.d.	n.d.	[462]
K_2DNPP	14	160	n.d.	n.d.	30.2	395	2.14	n.d.	n.d.	n.d.	n.d.	n.d.	n.d.	n.d.	[453]
Na_2DNPP	>40	160	n.d.	n.d.	30.6	365	2.20	n.d.	n.d.	n.d.	n.d.	n.d.	n.d.	n.d.	[453]
Ag_2DNPP	29	160	n.d.	n.d.	19.6	327	3.27	n.d.	n.d.	n.d.	n.d.	n.d.	n.d.	n.d.	[453]
$K_2DNADNPP$	2	20	n.d.	n.d.	35.5	208	2.11	152.9	n.d.	8306	312	n.d.	n.d.	n.d.	[454]
$K_2DNADNP$	15	120	n.d.	n.d.	31.6	272	2.161	-135.8	n.d.	7979	290	n.d.	n.d.	n.d.	[455]
KTNPO	3	60	n.d.	n.d.	27.2	179	2.102	-98.0	n.d.	8315	317	n.d.	n.d.	n.d.	[455]
$CsANABTr \cdot H_2O$	60	360	n.d.	n.d.	34.9	225	2.413	974.1	n.d.	6780	239	n.d.	n.d.	n.d.	[463]
$[Cu(H_2O)_2(11DTP)_2](CDNM)_2$	10	>360	500	<100	42.8	212	1.761	n.d.	n.d.	n.d.	n.d.	n.d.	n.d.	dec.	[88]
$[Cu(11DTP)_3](ADN)_2$	3	>360	700	<100	51.5	153	1.711	n.d.	n.d.	n.d.	n.d.	n.d.	n.d.	def.	[88]
$[Cu(CDNM)_2(1MTz)_4]$	4	324	1500	100-500	46.4	201	1.746	n.d.	n.d.	n.d.	n.d.	n.d.	n.d.	dec.	[71]
$[Cu(ADN)_2(NH_3)_2]$	2	60	1080	500-1000	40.8	179	n.d.	n.d.	n.d.	n.d.	n.d.	dec.	dec.	dec.	[434]
$[Cu(4ATr)_3](ADN)_2$	8	80	14	<100	47.8	203	n.d.	n.d.	n.d.	n.d.	n.d.	dec.	def.	def.	[434]
$[Cu(BTr)_2(H_2O)_2](ADN)_2$	7	144	181	<100	43.2	195	1.945	n.d.	n.d.	n.d.	n.d.	dec.	def.	dec.	[434]
$[Cu(1MTz)_6](ADN)_2$	8	120	203	500-1000	53.9	159	n.d.	n.d.	n.d.	n.d.	n.d.	def.	def.	def.	[434]
$[Cu(AzEtTz)_2(ADN)_2]$	2	5	250	<100	52.8	106	n.d.	n.d.	n.d.	n.d.	n.d.	def.	def.	def.	[434]
$[Cu(2M5ATz)_4(ADN)_2] \cdot 2H_2O$	4	80	>1500	500-1000	49.9	114	n.d.	n.d.	n.d.	n.d.	n.d.	def.	def.	def.	[434]
$[Cu(1A5MTz)_4(H_2O)_2](ADN)_2$	2	3	250	<100	52.8	89	1.689	n.d.	n.d.	n.d.	n.d.	det.	def.	det.	[434]
$[Cu(11DTM)_2(H_2O)_2](ADN)_2$	2	40	>1500	>1000	50.0	165	2.017	n.d.	n.d.	n.d.	n.d.	def.	def.	def.	[434]

Table S6. cont.

Compound	*IS* [J]	*FS* [N]	*ESD* [mJ]	Grain size [µm]	N content [%]	T_{dec} [°C]	*d* [g cm^{-3}]	*HOF* [kJ mol^{-1}]	ΔU_{ex} [kJ kg^{-1}]	*DV* [m s^{-1}]	p_{ex} [kbar]	HN	HP	Laser test	Ref.
$[Cu(22DTM)_3](ADN)_2$	2	15	270	500-1000	57.4	147	1.893	n.d.	n.d.	n.d.	n.d.	def.	def.	def.	[434]
$[Cu(11DTE)_3](ADN)_2$	1	72	76	<100	54.3	164	1.748	n.d.	n.d.	n.d.	n.d.	def.	def.	def.	[434]
$[Cu(22DTE)_3](ADN)_2$	2	30	250	500-1000	54.3	150	1.816	n.d.	n.d.	n.d.	n.d.	def.	def.	dec.	[434]
$[Cu(11BTziP)_2(H_2O)_2](ADN)_2$	2	120	317	<100	45.9	152	1.753	n.d.	n.d.	n.d.	n.d.	def.	def.	dec.	[434]
$[Cu(12DTP)_3](ADN)_2$	2	60	840	100-500	51.5	141	n.d.	n.d.	n.d.	n.d.	n.d.	def.	def.	dec.	[434]
$[Cu(11DTB)_2](ADN)_2$	3	240	>1500	<100	49.0	147	1.663	n.d.	n.d.	n.d.	n.d.	dec.	dec.	def.	[434]
$[Cu(1NOETz)_2(ADN)_2]$	2	7	1080	500-1000	37.7	110	1.953	n.d.	n.d.	n.d.	n.d.	def.	def.	det.	[89]
$[CuCl_4(H1A123Tr)_2]$	35	216	1400	100-500	29.8	148	2.038	n.d.	n.d.	n.d.	n.d.	n.d.	n.d.	n.d.	[68]
$[CuCl_2(1A123Tr)_2]$	>40	>360	700	<100	37.0	165	n.d.	n.d.	n.d.	n.d.	n.d.	n.d.	n.d.	n.d.	[68]
KADN	>20	>360	>5.6	<1000	29.0	220-230	n.d.	n.d.	n.d.	n.d.	n.d.	n.d.	n.d.	n.d.	[433]
CsADN	>20	>360	>5.6	<1000	17.6	220-225	n.d.	n.d.	n.d.	n.d.	n.d.	n.d.	n.d.	n.d.	[433]
AgNCA	3	10	51	<100	55.6	158	n.d.	n.d.	n.d.	n.d.	n.d.	def.	def.	n.d.	[439]
$[Cu(H_2O)_4(NCA)_2]$	>40	>360	1500	500-1000	27.3	108	n.d.	n.d.	n.d.	n.d.	n.d.	dec.	dec.	n.d.	[439]
$[Cu(1MTz)_4(NCA)_2]$	40	240	750	500-1000	53.9	159	1.715	n.d.	n.d.	n.d.	n.d.	dec.	dec.	n.d.	[439]
$[Cu(1A5MTz)_4(NCA)](NCA)$	<1	20	250	100-500	57.6	123	1.635	n.d.	n.d.	n.d.	n.d.	def.	def.	n.d.	[439]
$[Cu(11DTM)_2(NCA)_2]$	7	112	750	<100	57.1	191	1.880	n.d.	n.d.	n.d.	n.d.	dec.	def.	n.d.	[439]
$[Cu(12BTzC)_2(NCA)_2]$	2	80	480	<100	57.1	157	1.932	n.d.	n.d.	n.d.	n.d.	dec.	def.	n.d.	[439]
$[Cu(11DTE)_2(NCA)_2]$	6	180	>1500	500-1000	54.3	174	1.824	n.d.	n.d.	n.d.	n.d.	dec.	dec.	n.d.	[439]
$[Cu(11DTE)_3](NCA)_2 \cdot 2H_2O$	<1	160	>1500	>1000	54.6	168	n.d.	n.d.	n.d.	n.d.	n.d.	dec.	dec.	n.d.	[439]
$Na_4TNAE \cdot H_2O$	2	240	300	100-500	29.8	197	2.060	n.d.	n.d.	n.d.	n.d.	n.d.	n.d.	n.d.	[436]
$K_4TNAE \cdot 2H_2O$	10	>360	1500	100-500	24.4	142	2.159	n.d.	n.d.	n.d.	n.d.	n.d.	n.d.	n.d.	[436]
K_4TNAE	4	>360	1500	<100	26.5	225	2.152	-1554	n.d.	n.d.	n.d.	n.d.	n.d.	n.d.	[436]
HADNPCOx	12	200	n.d.	n.d.	39.9	201	1.80	n.d.	6360	8402	320	n.d.	n.d.	n.d.	[483]
KADNPCOx	6	100	n.d.	n.d.	35.6	200	1.93	n.d.	5104	7704	269	n.d.	n.d.	n.d.	[483]
$K_2NADNPCOx$	1.2	40	n.d.	n.d.	32.1	231	2.04	n.d.	6192	8059	309	n.d.	n.d.	n.d.	[483]
$K_2BDNMNP$	6	40	n.d.	n.d.	26.8	202	2.10	-475	n.d.	7965	293	n.d.	n.d.	n.d.	[456]
H_2DNOP	32	160	n.d.	n.d.	32.2	172	1.86	-76.8	n.d.	8541	323	n.d.	n.d.	n.d.	[457]
Na_2DNOP	>40	>360	n.d.	n.d.	25.7	395	2.13	-445	n.d.	6840	210	n.d.	n.d.	n.d.	[457]
K_2DNOP	25	>360	n.d.	n.d.	22.4	376	2.22	-543	n.d.	7085	218	n.d.	n.d.	n.d.	[457]
Na_2ADNP	27	240	n.d.	n.d.	35.9	317	1.85	14.3	n.d.	7378	234	n.d.	n.d.	n.d.	[457]
K_2ADNP	18	160	n.d.	n.d.	33.2	315	1.90	-62.3	n.d.	7299	225	n.d.	n.d.	n.d.	[457]
$LiBTrBo \cdot 1.5H_2O$	>40	>360	>1500	100-500	46.0	317	1.40	n.d.	n.d.	n.d.	n.d.	n.d.	n.d.	n.d.	[494]
$LiBTzBo \cdot H_2O$	10	>360	200	<100	63.7	213	1.30	n.d.	n.d.	n.d.	n.d.	n.d.	n.d.	n.d.	[494]
LiBNPBo	2	144	420	100-500	34.5	200	n.d.	n.d.	n.d.	n.d.	n.d.	n.d.	n.d.	n.d.	[495]

Table S6. cont.

Compound	*IS* [J]	*FS* [N]	*ESD* [mJ]	Grain size [μm]	N content [%]	T_{dec} [°C]	*d* [g cm^{-3}]	*HOF* [kJ mol^{-1}]	ΔU_{ex} [kJ kg^{-1}]	*DV* [m s^{-1}]	p_{ex} [kbar]	HN	HP	Laser test	Ref.
LiBATzBo	>40	>360	>1500	100-500	74.6	280	n.d.	n.d.	n.d.	n.d.	n.d.	n.d.	n.d.	n.d.	[495]
KBNTrBo	4	360	1200	<100	40.3	242	n.d.	n.d.	n.d.	n.d.	n.d.	n.d.	n.d.	n.d.	[496]
Ba(BNTrBo)$_2$	5	360	1200	<100	36.4	224	n.d.	n.d.	n.d.	n.d.	n.d.	n.d.	n.d.	n.d.	[496]
KBDNPBo	3	216	1000	100-500	30.6	344	n.d.	n.d.	n.d.	n.d.	n.d.	n.d.	n.d.	n.d.	[496]
Ba(BDNPBo)$_2$	5	180	400	100-500	28.3	342	n.d.	n.d.	n.d.	n.d.	n.d.	n.d.	n.d.	n.d.	[496]
KBDNImBo	>40	>360	1500	100-500	30.6	165	n.d.	n.d.	n.d.	n.d.	n.d.	n.d.	n.d.	n.d.	[496]
Ba(BDNImBo)	>40	>360	1500	100-500	28.3	349	n.d.	n.d.	n.d.	n.d.	n.d.	n.d.	n.d.	n.d.	[496]
H_2EDN	5	240	960	100-1000	37.3	183	1.720	-364	-5443	8634	301	dec.	def.	n.d.	[435]
Li_2EDN·2H_2O	>40	>360	>1500	<100	28.3	276	1.746	n.d.	n.d.	n.d.	n.d.	dec.	dec.	n.d.	[435]
Na_2EDN	3	240	1350	<100	28.9	304	n.d.	n.d.	n.d.	n.d.	n.d.	def.	def.	n.d.	[435]
K_2EDN·2H_2O	5	>360	>1500	<100	21.4	329	1.864	n.d.	n.d.	n.d.	n.d.	def.	def.	n.d.	[435]
Rb_2EDN	3	288	>1500	<100	17.6	342	2.641	n.d.	n.d.	n.d.	n.d.	def.	def.	n.d.	[435]
Cs_2EDN	9	216	203	100-500	13.5	349	3.170	n.d.	n.d.	n.d.	n.d.	def.	def.	n.d.	[435]
Ag_2EDN	2	72	368	<100	15.4	211	n.d.	n.d.	n.d.	n.d.	n.d.	def.	def.	n.d.	[435]
MgEDN·6H_2O	>40	>360	>1500	500-1000	20.0	131	1.623	n.d.	n.d.	n.d.	n.d.	dec.	dec.	n.d.	[435]
CaEDN·6H_2O	>40	>360	>1500	500-1000	18.9	285	1.707	n.d.	n.d.	n.d.	n.d.	dec.	dec.	n.d.	[435]
BaEDN·H_2O	>40	>360	123	<100	18.5	268	n.d.	n.d.	n.d.	n.d.	n.d.	dec.	def.	n.d.	[435]
[Cu(EDN)(NH_3)$_2$]	3	180	>1500	500-1000	34.2	212	2.007	n.d.	n.d.	n.d.	n.d.	dec.	def.	dec.	[435]
[Cu_3(4ATr)$_2$(EDN)$_2$(OH)$_2$]·2H2O	2	360	227	<100	30.9	194	1.838	n.d.	n.d.	n.d.	n.d.	dec.	def.	dec.	[435]
[Cu(11DTE)(EDN)]	10	48	106	<100	44.5	203	1.794	n.d.	n.d.	n.d.	n.d.	dec.	def.	def.	[435]
[Cu(11BTziP)(EDN)]	<1	64	106	<100	42.9	180	n.d.	n.d.	n.d.	n.d.	n.d.	dec.	def.	def.	[435]
[Cu(11BTC3)$_2$(EDN)]	10	60	177	<100	49.0	177	1.702	n.d.	n.d.	n.d.	n.d.	dec.	def.	def.	[435]
[Ag_2(EDN)(NH_3)$_2$]	9	>360	840	>1000	21.1	237	2.874	n.d.	n.d.	n.d.	n.d.	dec.	def.	n.d.	[435]
[Ag_2(EDN)(4ATr)$_2$]·2H_2O	>40	>360	540	500-1000	29.6	222	2.432	n.d.	n.d.	n.d.	n.d.	dec.	def.	n.d.	[435]
HTNMNNATr	4	144	n.d.	n.d.	35.1	121	1.97	-223.1	n.d.	9033	342	n.d.	n.d.	n.d.	[469]
KTNMNNATr	2	32	n.d.	n.d.	33.2	115	2.00	-183.0	n.d.	8739	309	n.d.	n.d.	n.d.	[469]
[Cu(BNTr)(TrDA)$_3$]$_n$	>40	>360	n.d.	n.d.	52.4	323	1.93	n.d.	4346	7700	n.d.	n.d.	n.d.	n.d.	[490]
[Cu(BNTrO)(TrDA)$_2$(H_2O)$_2$]$_n$	18	360	n.d.	n.d.	44.8	333	1.96	n.d.	4476	7860	n.d.	n.d.	n.d.	n.d.	[490]
[Ni(HENAPr)$_2$(Im)($CH_3C(NH_2)O_2$)]	8	360	12	n.d.	35.4	155	1.7	n.d.	n.d.	n.d.	n.d.	n.d.	n.d.	+	[499]
[Cu(ENAPrP)$_2$]·2H_2O	8	360	51	n.d.	32.5	128	1.320	240.6	3931	5440	110	n.d.	n.d.	+	[499]
H34DNP	25	360	1000	<100	35.4	175	1.79	124.2	5409	8426	306	n.d.	n.d.	n.d.	[448]
H35DNP	25	360	1000	<100	35.4	299	1.76	189.1	5752	8459	308	n.d.	n.d.	n.d.	[448]
Na34DNP·2H_2O	40	360	1000	<500	25.9	142	1.74	-1071	2070	6245	141	n.d.	n.d.	n.d.	[448]
K34DNP·2H_2O	40	360	1500	<500	24.1	174	1.72	-675.4	3543	6800	178	n.d.	n.d.	n.d.	[448]
Na35DNP·2H_2O	40	360	1500	<500	25.9	297	1.72	n.d.	n.d.	n.d.	n.d.	n.d.	n.d.	n.d.	[448]
K35DNP	8.5	240	400	<500	28.6	307	1.99	34	4764	8109	259	n.d.	n.d.	n.d.	[448]

Table S6. cont.

Compound	*IS* [J]	*FS* [N]	*ESD* [mJ]	Grain size [μm]	N content [%]	T_{dec} [°C]	*d* [g cm^{-3}]	*HOF* [kJ mol^{-1}]	ΔU_{ex} [kJ kg^{-1}]	*DV* [m s^{-1}]	p_{ex} [kbar]	HN	HP	Laser test	Ref.
[Ag(34DNP)]·1.5H_2O	10	120	n.d.	n.d.	20.1	195	2.162	n.d.	2908	5488	157	n.d.	n.d.	n.d.	[449]
[Ag(34DNP)]	7.5	115	n.d.	n.d.	21.1	262	2.160	n.d.	3017	5532	159	n.d.	n.d.	n.d.	[449]
[Co(DNBA)$_2$(H_2O)$_4$]·4H_2O	>40	>360	n.d.	n.d.	9.0	369	1.724	n.d.	n.d.	n.d.	n.d.	n.d.	n.d.	n.d.	[487]
[Ni(DNBA)$_2$(H_2O)$_4$]·4H_2O	>40	>360	n.d.	n.d.	8.9	351	1.742	n.d.	n.d.	n.d.	n.d.	n.d.	n.d.	n.d.	[487]
[Zn(DNBA)$_2$]$_n$	15	>360	n.d.	n.d.	11.5	374	1.574	n.d.	n.d.	n.d.	n.d.	n.d.	n.d.	n.d.	[487]
Co(SCZ)$_2$(DNBA)$_2$	25	>360	n.d.	n.d.	22.2	247	1.764	n.d.	n.d.	n.d.	n.d.	n.d.	n.d.	n.d.	[487]
Ni(SCZ)$_2$(DNBA)$_2$	17.5	>360	n.d.	n.d.	22.2	268	1.785	n.d.	n.d.	n.d.	n.d.	n.d.	n.d.	n.d.	[487]
Zn(SCZ)$_2$(DNBA)$_2$	30	>360	n.d.	n.d.	22.0	221	1.778	n.d.	n.d.	n.d.	n.d.	n.d.	n.d.	n.d.	[487]
Co(15DATz)$_2$(DNBA)$_2$(H_2O)$_2$	22.5	>360	n.d.	n.d.	31.2	190	1.742	n.d.	n.d.	n.d.	n.d.	n.d.	n.d.	n.d.	[487]
Ni(15DATz)$_2$(DNBA)$_2$(H_2O)$_2$	10	>360	n.d.	n.d.	31.2	197	1.754	n.d.	n.d.	n.d.	n.d.	n.d.	n.d.	n.d.	[487]
Zn(15DATz)$_2$(DNBA)$_2$(H_2O)$_2$	27.5	>360	n.d.	n.d.	31.0	201	1.765	n.d.	n.d.	n.d.	n.d.	n.d.	n.d.	n.d.	[487]
[Cu(DNBA)$_2$(bpy)]	20.7	>360	n.d.	n.d.	13.1	249	n.d.	n.d.	n.d.	n.d.	n.d.	n.d.	n.d.	n.d.	[488]
[Zn(μ_3-OH)$_2$(ASTr)$_4$]$_n$	>40	>360	n.d.	n.d.	32.4	248	2.032	2012.7	13599	n.d.	n.d.	n.d.	n.d.	n.d.	[470]
AgPz	0.5	1	n.d.	n.d.	39.4	98	n.d.	n.d.	n.d.	n.d.	n.d.	n.d.	n.d.	n.d.	[476]
KPz	5	40	n.d.	n.d.	64.2	110	1.959	136.8	n.d.	6977	209	n.d.	n.d.	n.d.	[476]
LiPz	3	20	n.d.	n.d.	91.0	133	1.75	264.5	n.d.	11362	409	n.d.	n.d.	n.d.	[477]
[Na(H_2O)Pz]·2H_2O	+	+	n.d.	n.d.	47.6	111	1.471	n.d.	n.d.	n.d.	n.d.	n.d.	n.d.	n.d.	[474]
[Mn(H_2O)$_4$Pz$_2$]·4H_2O	+	+	n.d.	n.d.	41.3	104	1.608	n.d.	n.d.	n.d.	n.d.	n.d.	n.d.	n.d.	[474]
[Fe(H_2O)$_4$Pz$_2$]·4H_2O	+	+	n.d.	n.d.	41.2	115	1.599	n.d.	n.d.	n.d.	n.d.	n.d.	n.d.	n.d.	[474]
[Mg(H_2O)$_4$Pz$_2$]·4H_2O	+	+	n.d.	n.d.	40.7	104	1.437	n.d.	n.d.	n.d.	n.d.	n.d.	n.d.	n.d.	[474]
[Co(H_2O)$_4$Pz$_2$]·4H_2O	+	+	n.d.	n.d.	40.8	80	1.694	n.d.	n.d.	n.d.	n.d.	n.d.	n.d.	n.d.	[475]
	+	+	n.d.	n.d.		59	1.696	n.d.	n.d.	n.d.	n.d.	n.d.	n.d.	n.d.	[474]
[Ag(NH_3)$_2$][Ag$_3$Pz$_4$]	14.8	+	n.d.	n.d.	41.3	90-134	3.200	n.d.	n.d.	n.d.	n.d.	n.d.	n.d.	n.d.	[478]
[Fe(H_2O)$_6$}Pz$_3$·9H_2O	n.d.	n.d.	n.d.	n.d.	39.2	109	1.428	n.d.	n.d.	n.d.	n.d.	n.d.	n.d.	n.d.	[473]
[Al(H_2O)$_6$}Pz$_3$·9H_2O	n.d.	n.d.	n.d.	n.d.	41.4	141	1.384	n.d.	n.d.	n.d.	n.d.	n.d.	n.d.	n.d.	[473]
[Pb(OH)]$_4$Pz$_4$	9.5	120	n.d.	n.d.	23.8	80	4.537	n.d.	n.d.	n.d.	n.d.	n.d.	n.d.	n.d.	[479]
[Pb$_3$(H_2O)$_9$(NO_3)Pz$_3$]$_4$Pz$_9$(H_2O)$_5$	20	240	n.d.	n.d.	29.9	89	2.852	n.d.	n.d.	n.d.	n.d.	n.d.	n.d.	n.d.	[479]
[Pb(OH)]$_4$Pz$_3$(NO_3)(H_2O)$_3$	16	160	n.d.	n.d.	18.3	97	3.913	n.d.	n.d.	n.d.	n.d.	n.d.	n.d.	n.d.	[479]
[Pb(OH)]$_4$Pz$_3$(ClO_4)(H_2O)$_3$	12	180	n.d.	n.d.	17.2	110	4.417	n.d.	n.d.	n.d.	n.d.	n.d.	n.d.	n.d.	[479]
H_2TNBIm·2H_2O	0.4	>360	n.d.	n.d.	32.0	269	n.d.	n.d.	n.d.	n.d.	n.d.	n.d.	n.d.	n.d.	[443]
[Cu(NH_3)$_2$(H_2O)(TNBIm)]	6.5	>360	n.d.	n.d.	32.8	200	n.d.	n.d.	n.d.	n.d.	n.d.	n.d.	n.d.	n.d.	[443]
HTNMTrCA	5	100	n.d.	n.d.	32.1	137	1.821	n.d.	6485	8720	339	n.d.	n.d.	n.d.	[471]
KDNMTrCAH	2	80	n.d.	n.d.	27.4	168	2.001	n.d.	4686	7730	281	n.d.	n.d.	n.d.	[471]
RbDNMTrCAH	2	80	n.d.	n.d.	23.3	154	2.297	n.d.	n.d.	n.d.	n.d.	n.d.	n.d.	n.d.	[471]
[Cs(DNMTrCAH)(DNMTrCA)]·3H_2O	2.5	80	n.d.	n.d.	22.7	144	2.301	n.d.	n.d.	n.d.	n.d.	n.d.	n.d.	n.d.	[471]
KTNMTrCA	1.5	60	n.d.	n.d.	28.0	162	1.880	n.d.	4853	7910	285	n.d.	n.d.	n.d.	[471]

Table S6. cont.

Compound	*IS* [J]	*FS* [N]	*ESD* [mJ]	Grain size [μm]	N content [%]	T_{dec} [°C]	*d* [g cm^{-3}]	*HOF* [kJ mol^{-1}]	ΔU_{ex} [kJ kg^{-1}]	*DV* [m s^{-1}]	p_{ex} [kbar]	HN	HP	Laser test	Ref.
AgTNMTrCA	1.5	50	n.d.	n.d.	22.8	190	2.595	n.d.	5063	8740	411	n.d.	n.d.	n.d.	[471]
NaBCM	>40	>360	-	n.d.	44.4	296	2.02	n.d.	n.d.	n.d.	n.d.	n.d.	n.d.	n.d.	[500]
KPCM	10	240	n.d.	n.d.	34.9	315	1.97	104.8	n.d.	8008	280	n.d.	n.d.	n.d.	[501]
{[CuCl(55DT11P)]Cl}·7H_2O	>40	>360	1.50	100-500	40.2	259	1.596	n.d.	n.d.	n.d.	n.d.	n.d.	n.d.	n.d.	[360]
[Co(H_2O)(55DT11P)$_2$]Cl_2	>40	>360	1.50	500-1000	42.4	253	1.678	n.d.	n.d.	n.d.	n.d.	n.d.	n.d.	n.d.	[360]
[Ni(H_2O)$_2$(55DT11P)$_2$]Cl_2	>40	>360	1.50	500-1000	42.6	207	1.683	n.d.	n.d.	n.d.	n.d.	n.d.	n.d.	n.d.	[360]
[Cu(H_2O)(55DT11P)$_2$]SO_4·H_2O	>40	>360	0.75	100-500	40.3	225	1.776	n.d.	n.d.	n.d.	n.d.	n.d.	n.d.	n.d.	[360]
[Cu(μ-Cl)$_2$(2ATz)]	<2	40	10	<100	46.0	171	2.177	n.d.	n.d.	n.d.	n.d.	n.d.	n.d.	n.d.	[361]
[CuCl(μ-Cl)(1ATz)(μ-1ATz)]	3	60	50	<100	46.0	175	2.146	n.d.	n.d.	n.d.	n.d.	n.d.	n.d.	n.d.	[361]
{[Cu(TrDA)$_3$](BF_4)$_2$}$_n$	>40	>360	>24.75	n.d.	46.1	204	1.76	-816.8	-2365	6150	167	n.d.	n.d.	def.	[491]
{[Cu(TrDA)$_3$]SO_4}$_n$	>40	>360	>24.75	n.d.	51.6	221	1.95	-53.1	2468	5380	120	n.d.	n.d.	def.	[491]
[$CuCl_2$($H_2$55DTM)$_2$]·2H_2O	>40	>360	1500	100-500	47.2	139	1.859	n.d.	n.d.	n.d.	n.d.	n.d.	n.d.	n.d.	[356]
[Co(H_2O)$_2$($H_2$55DTM)$_2$]Cl_2	>40	>360	1000	100-500	47.7	243	1.920	n.d.	n.d.	n.d.	n.d.	n.d.	n.d.	n.d.	[356]
[Ni(H_2O)$_2$($H_2$55DTM)$_2$]Cl_2	>40	>360	1000	100-500	47.7	n.d.	1.934	n.d.	n.d.	n.d.	n.d.	n.d.	n.d.	n.d.	[356]
{[Cu_3(SO_4)$_2$(H55DTM)$_2$(H_2O)$_4$ ($H_2$55DTM)$_2$]·2H_2O}$_n$	>40	>360	800	<100	40.9	224	2.125	n.d.	n.d.	n.d.	n.d.	n.d.	n.d.	n.d.	[356]
HANNATzPd·0.5H_2O	18	84	n.d.	n.d.	50.4	175	1.940	521.9	n.d.	9345	380	n.d.	n.d.	n.d.	[502]
LiANNATzPd·2H_2O	>20	84	n.d.	n.d.	44.4	208	1.858	33.3	n.d.	8657	287	n.d.	n.d.	n.d.	[502]
NaANNATzPd·2H_2O	>20	80	n.d.	n.d.	42.1	249	1.898	-13.2	n.d.	8114	251	n.d.	n.d.	n.d.	[502]
KANNATzPd·2H_2O	>20	72	n.d.	n.d.	40.0	290	1.923	-25.9	n.d.	8324	258	n.d.	n.d.	n.d.	[502]
RbANNATzPd	>20	64	n.d.	n.d.	38.7	269	2.316	-38.2	n.d.	n.d.	n.d.	n.d.	n.d.	n.d.	[502]
CsANNATzPd	>20	64	n.d.	n.d.	35.6	276	2.516	-36.0	n.d.	n.d.	n.d.	n.d.	n.d.	n.d.	[502]
K_2HNNaTzPd·2H_2O	>20	96	n.d.	n.d.	31.6	316	1.931	-937.6	n.d.	6893	147	n.d.	n.d.	n.d.	[502]
Cs_2HNNaTzPd	>20	72	n.d.	n.d.	22.2	314	2.977	-946.6	n.d.	n.d.	n.d.	n.d.	n.d.	n.d.	[502]
LiHANTzPd·2H_2O	>20	108	n.d.	n.d.	41.0	277	1.668	-246.9	n.d.	6991	166	n.d.	n.d.	n.d.	[502]
NaHANTzPd·2H_2O	>20	96	n.d.	n.d.	38.4	285	1.782	-297.4	n.d.	6726	153	n.d.	n.d.	n.d.	[502]
KHANTzPd·2H_2O	>20	108	n.d.	n.d.	36.2	202	1.866	-312.6	n.d.	7285	176	n.d.	n.d.	n.d.	[502]
RbHANTzPd	>20	84	n.d.	n.d.	34.8	274	2.324	-324.8	n.d.	n.d.	n.d.	n.d.	n.d.	n.d.	[502]
[Cu(DCA)$_2$(1PryTz)$_2$]	4	>360	90	100-500	47.6	120	1.649	n.d.	n.d.	n.d.	n.d.	dec.	dec.	dec.	[125]
[Cu(AzEt124Tr)$_4$(NO_3)$_2$]	35	>360	42	<100	49.2	179	n.d.	n.d.	n.d.	n.d.	n.d.	dec.	dec.	-	[358]
[Cu(AzEt124Tr)$_4$(ClO_4)$_2$]	<1	108	50	<100	39.6	158	1.717	n.d.	n.d.	n.d.	n.d.	dec.	def.	det.	[358]
[Fe(AzEt124Tr)](ClO_4)$_2$	3	3.75	65	100-500	46.5	151	n.d.	n.d.	n.d.	n.d.	n.d.	det.	det.	dec.	[358]
[Cu_3(AzEt124Tr)$_2$(N_3)$_6$]	<1	0.2	2.5	<100	58.4	112	n.d.	n.d.	n.d.	n.d.	n.d.	det.	det.	-	[358]

References

1. Shimelmitz, R.; Kuhn, S.L.; Jelinek, A.J.; Ronen, A.; Clark, A.E.; Weinstein-Evron, M. 'Fire at will': The emergence of habitual fire use 350,000 years ago. *J. Hum. Evol.* **2014**, *77*, 196–203. https://doi.org/10.1016/j.jhevol.2014.07.005.
2. Canfarini, F.; Reverberi, A.; Vocciante, M.; Fabiano, B. Safety concerns and chemical aspects of improvised explosive devices and homemade explosives. *Chem. Eng. Trans.* **2002**, *91*, 181–186. https://doi.org/10.3303/CET2291031.
3. Pawlus, K.; Kwiatkowski, M.; Stolarczyk, A.; Glosz, K.; Jarosz, T. Synthesis of explosive peroxides using unrecognised explosive precursors—Percarbonates and perborates. *FirePhysChem* **2022**, *2*, 285–293. https://doi.org/10.1016/j.fpc.2022.06.005.
4. Jurczak, R. Analiza mikrośladów trinadtlenku triacetonu (TATP) w fazie gazowej z zastosowaniem TD-GC/MS. *Przegl. Bezp. Wewn.* **2021**, *13*, 250–276.
5. Curtius, T.; Radenhausen, R. Zur Kenntniss der Stickstoffwasserstoffsäure. *J. Prakt. Chem.* **1891**, *43*, 207–208. https://doi.org/10.1002/prac.18910430124.
6. Evans, B.L.; Yoffe, A.D.; Gray, P. Physics and chemistry of the inorganic azides. *Chem. Rev.* **1959**, *59*, 515–568. https://doi.org/10.1021/cr50028a001.
7. Fedoroff, B.T.; Aaronson, H.A.; Reese, E.F.; Sheffield, O.E.; Clift, G.D. *Encyclopedia of Explosives and Related Items. Vol. 1–10*; U.S. Army Research And Development Command Tacom, Ardec, Warheads, Energetics And Combat Support Center: Picatinny Arsenal, NJ, USA, 1960–1983.
8. Treitler, D.S.; Leung, S. How dangerous is too dangerous? A perspective on azide chemistry. *J. Org. Chem.* **2022**, *87*, 11293–11295. https://doi.org/10.1021/acs.joc.2c01402.
9. Papliński, A.; Maranda, A. Research on the detonation process of explosives containing sodium azide. *Cent. Eur. J. Energ. Mater.* **2019**, *16*, 520–532. https://doi.org/10.22211/cejem/115263.
10. Matyáš, R.; Šelešovský, J.; Musil, T. Sensitivity to friction for primary explosives. *J. Hazard. Mater.* **2012**, *213–214*, 236–241. https://doi.org/10.1016/j.jhazmat.2012.01.085.
11. Matyáš, R.; Pachman, J. *Primary Explosives*; Springer: Berlin, Germany, 2013.
12. Tammann, G.; Kröger, C. Über die Verpuffungstemperatur und Schlagempfindlichkeit von flüssigen und festen Explosivstoffen. *Z. Anorg. Allg. Chem.* **1928**, *169*, 1–32. https://doi.org/10.1002/zaac.19281690102.
13. Wöhler, L.; Krupko, W. Über die Lichtempfindlichkeit der Azide des Silbers, Quecksilberoxyduls, Bleis und Kupferoxyduls, sowie-über basisches Blei- und Cupriazid. *Ber. Dtsch. Chem. Ges.* **1913**, *46*, 2045–2057. https://doi.org/10.1002/cber.191304602121.
14. Todd, G.; Tasker, M.P. The identity of the gamma modification of basic lead azide type I. *Helv. Chim. Acta* **1971**, *54*, 2210–2212. https://doi.org/10.1002/hlca.19710540758.
15. Urbański, T. Chemistry and Technology of Explosives. Vol. 1–4; Pergamon Press: Oxford, UK, 1964–1984.
16. Fair, H.D.; Walker, R.F. Energetic Materials: Physics and Chemistry of Inorganic Azides; Plenum Press: New York, NY, USA, 1977.
17. Evans, B.L.; Yoffe, A.D.; Bowden, F.P. The burning and explosion of single crystals. *Proc. R. Soc. A: Math. Phys. Sci.* **1957**, *238*, 325–333. https://doi.org/10.1098/rspa.1957.0003.
18. Feng, Y.; Chen, S.; Li, Z.; Zhang, T. Performance of copper(II)-azide with hydrogen bonding as initiating explosive. *Chem. Eng. J.* **2022**, *429*, 132186. https://doi.org/10.1016/j.cej.2021.132186.
19. Braun, V.; Wurzenberger, M.H.H.; Weippert, V.; Stierstorfer, J. Tailoring the properties of 3d transition metal complexes with different *N*-cycloalkyl-substituted tetrazoles. *New J. Chem.* **2021**, *45*, 11042–11050. https://doi.org/10.1039/D1NJ01675J.
20. Kofen, M.; Lommel, M.; Wurzenberger, M.H.H.; Klapötke, T.M.; Stierstorfer, J. 1-(Azidomethyl)-5*H*-Tetrazole: A Powerful New Ligand for Highly Energetic Coordination Compounds. *Chem. Eur. J.* **2022**, *28*, e202200492. https://doi.org/10.1002/chem.202200492.
21. Wu, B.-d.; Wang, S.-w.; Yang, L.; Zhang, T.-l.; Zhang, J.-g.; Zhou, Z.-n.; Yu, K.-b. Preparation, crystal structures, thermal decomposition and explosive properties of two novel energetic compounds $M(IMI)_4(N_3)_2$ (M = Cu^{II} and Ni^{II}, IMI = Imidazole): The new high–nitrogen materials (N > 46 %). *Eur. J. Inorg. Chem.* **2011**, *2011*, 2616–2623. https://doi.org/10.1002/ejic.201100054.
22. Wurzenberger, M.H.H.; Lommel, M.; Gruhne, M.S.; Szimhardt, N.; Stierstorfer, J. Refinement of copper(II) azide with 1-alkyl-5*H*-tetrazoles: Adaptable energetic complexes. *Angew. Chem. Int. Ed.* **2020**, *59*, 12367–12370. https://doi.org/10.1002/anie.202002823.
23. Joyner, T.B.; Cohen, A.J.; McEwan, W.S. Explosive sensitivity of cobalt ammine azides. *Nature* **1962**, *196*, 470–471. https://doi.org/10.1038/196470a0.
24. Patil, K.C.; Nesamani, C.; Pai Verneker, V.R. Synthesis and characterisation of metal hydrazine nitrate, azide and perchlorate complexes. *Synth. React. Inorg. Met. Org. Chem.* **1982**, *12*, 383–395. https://doi.org/10.1080/00945718208063122.
25. Wu, B.-D.; Yang, L.; Wang, S.-W.; Zhang, T.-L.; Zhang, J.-G.; Zhou, Z.-N.; Yu, K.-B. Preparation, crystal structure, thermal decomposition, and explosive properties of a novel energetic compound $[Zn(N_2H_4)_2(N_3)_2]_n$:

A new high-nitrogen material (N = 65.60%). *Z. Anorg. Allg. Chem.* **2011**, *637*, 450–455. https://doi.org/10.1002/zaac.201000340.
26. Liu, Z.; Zhang, T.; Zhang, J.; Wang, S. Studies on three-dimensional coordination polymer $[Cd_2(N_2H_4)_2(N_3)_4]_n$: Crystal structure, thermal decomposition mechanism and explosive properties. *J. Hazard. Mater.* **2008**, *154*, 832–838. https://doi.org/10.1016/j.jhazmat.2007.10.099.
27. Yang, L.; Wu, B.; Zhang, T.; Liu, Z.; Zhang, J. Preparation, crystal structure, thermal decomposition, and explosive properties of $[Cd(en)(N_3)_2]_n$. *Propell. Explos. Pyrot.* **2010**, *35*, 521–528. https://doi.org/10.1002/prep.200900095.
28. Wang, T.-w.; Wang, X.-j.; Yi, Z.-x.; Cao, W.-l.; Dong, W.-s.; Bi, Y.-f.; Zhu, S.-g.; Zhang, J.-G. Competitive coordination of azide groups: Synthesis of solvent-free and chlorine-free primary explosives based on 3-amino-1-nitroguanidine. *Cryst. Growth Des.* **2021**, *21*, 7002–7007. https://doi.org/10.1021/acs.cgd.1c00926.
29. Agrell, I. The crystal structure of diazidodipyridinecadmium $[Cd(N_3)_2(C_5H_5N)_2]$. *Acta Chem. Scand.* **1970**, *24*, 3575–3589. https://doi.org/10.3891/acta.chem.scand.24-3575.
30. Agrell, I. The infra-red spectra of some inorganic azide compounds. *Acta Chem. Scand.* **1971**, *25*, 2965–2974. https://doi.org/10.3891/acta.chem.scand.25-2965.
31. Lund, H.; Oeckler, O.; Schröder, T.; Schulz, A.; Villinger, A. Mercury azides and the azide of Millon's Base. *Angew. Chem. Int. Ed.* **2013**, *52*, 10900–10904. https://doi.org/10.1002/anie.201305545.
32. Li, H.; Wang, Y.; Wei, Z.; Yang, X.; Liang, L.; Xia, L.; Long, M.; Li, Z.; Zhang, T. Tunable copper complexes with functional ligands: A promising strategy for green primary explosives. *Chem. Eng. J.* **2022**, *430*, 132739. https://doi.org/10.1016/j.cej.2021.132739.
33. Xu, J.-G.; Sun, C.; Zhang, M.-J.; Liu, B.-W.; Li, X.-Z.; Lu, J.; Wang, S.-H.; Zheng, F.-K.; Guo, G.-C. Coordination polymerization of metal azides and powerful nitrogen-rich ligand toward primary explosives with excellent energetic performances. *Chem. Mater.* **2017**, *29*, 9725–9733. https://doi.org/10.1021/acs.chemmater.7b03453.
34. Pelletier, V.; Bhattacharyya, S.; Knoke, I.; Forohar, F.; Bichay, M.; Gogotsi, Y. Copper azide confined inside templated carbon nanotubes. *Adv. Funct. Mater.* **2010**, *20*, 3168–3174. https://doi.org/10.1002/adfm.201000858.
35. Wang, Q.; Feng, X.; Wang, S.; Song, N.; Chen, Y.; Tong, W.; Han, Y.; Yang, L.; Wang, B. Explosives: Metal-organic framework templated synthesis of copper azide as the primary explosive with low electrostatic sensitivity and excellent initiation ability. *Adv. Mater.* **2016**, *28*, 5766. https://doi.org/10.1002/adma.201670192.
36. Xu, J.-G.; Li, X.-Z.; Wu, H.-F.; Zheng, F.-K.; Chen, J.; Guo, G.-C. Substitution of nitrogen-rich linkers with insensitive linkers in azide-based energetic coordination polymers toward safe energetic materials. *Cryst. Growth Des.* **2019**, *19*, 3934–3944. https://doi.org/10.1021/acs.cgd.9b00351.
37. Xu, J.-G.; Lin, S.-J.; Li, X.-Z.; Wu, H.-F.; Lu, J.; Wang, W.-F.; Chen, J.; Zheng, F.-K.; Guo, G.-C. Energetic azide-based coordination polymers: Sensitivity tuning through diverse structural motifs. *Chem. Eng. J.* **2020**, *390*, 124587. https://doi.org/10.1016/j.cej.2020.124587.
38. Puszynski, M.M.; Mehta, N.; Cheng, G.; Oyler, K.D.; Fischer, D.; Klapötke, T.M.; Stierstorfer, J. M42 primer processing with potassium and copper salt formulations. *Jacobs J. Inorg. Chem.* **2016**, *1*, 003.
39. Yu, C.; Zhang, W.; Xian, M.; Wang, J.; Chen, J.; Chen, Y.; Shi, W.; Yang, G.; Ye, J.; Ma, K.; et al. Copper azide nanoparticle-encapsulating MOF-derived porous carbon: Electrochemical preparation for high-performance primary explosive film. *Small* **2022**, *18*, 2107364. https://doi.org/10.1002/smll.202107364.
40. Liu, X.; Hu, Y.; Chen, B.; Wei, H.; Su, J.; Yang, C.; Hu, J.; Ye, Y.; Shen, R. Fabrication and properties of MEMS compatible energetic arrays based on carbon-based copper azide. *Appl. Surf. Sci.* **2022**, *577*, 150643. https://doi.org/10.1016/j.apsusc.2021.150643.
41. Guang, F.; Yin-Li, Z.; Jia-Juan, S.; Min-Yan, Z. Energetic cuprous azide complex: Synthesis, crystal structure, and characterization. *J. Coord. Chem.* *64*, 3711-3717. https://doi.org/10.1080/00958972.2011.629656.
42. Tornieporth-Oetting, I.C.; Klapötke, T.M. Covalent inorganic azides. *Angew. Chem. Int. Ed. Engl.* **1995**, *34*, 511–520. https://doi.org/10.1002/anie.199505111.
43. Linhard, M.; Flygare, H. Über Komplexverbindungen. IV. Azido-pentamminkobalt(III)-komplexe. *Z. Anorg. Allg. Chem.* **1950**, *262*, 328–343. https://doi.org/10.1002/zaac.19502620606.
44. Linhard, M.; Weigel, M.; Flygare, H. Über Komplexverbindungen. V. *Cis*- und *trans*-Diazido-tetrammin-kobalt(III)-salze. *Z. Anorg. Allg. Chem.* **1950**, *263*, 233–244. https://doi.org/10.1002/zaac.19502630504.
45. Linhard, M.; Weigel, M. Über Komplexverbindungen. VI. Triazido-triammin-kobalt mit 1 Abbildung. *Z. Anorg. Allg. Chem.* **1950**, *263*, 245–252. https://doi.org/10.1002/zaac.19502630505.
46. Deblitz, R.; Hrib, C.G.; Blaurock, S.; Jones, P.G.; Plenikowski, G.; Edelmann, F.T. Explosive Werner-type cobalt(III) complexes. *Inorg. Chem. Front.* **2014**, *1*, 621–640. https://doi.org/10.1039/C4QI00094C.
47. Siebert, H.; Macht, R. Hexaazidocobaltate(III) $[Co(N_3)_6]^{3-}$. *Z. Anorg. Allg. Chem.* **1982**, *489*, 77–84. https://doi.org/10.1002/zaac.19824890110.
48. Druding, L.F.; Sancilio, F.D.; Lukaszewski, D.M. Triammines of cobalt(III). IV. Preparation, properties, and structure of the polymerization isomer *trans*-diazidotetraamminecobalt(III) *trans*-tetraazidodiamminecobaltate(III), $[Co(NH_3)_4(N_3)_2][Co(NH_3)_2(N_3)_4]$. *Inorg. Chem.* **1975**, *14*, 1365–1369. https://doi.org/10.1021/ic50148a032.

49. Explosive Precursor Chemicals; Interpol: Lyon, France, 2018.
50. Batt, D. Explosive Package Bore Al-Qaida Hallmarks, Dubai Police Say. *Guardian* 2010. Available online: https://www.theguardian.com/world/2010/oct/30/explosive-packages-alqaida-hallmarks-dubai (accessed on 15 September 2024).
51. Multiple Explosive Devices Found in Streeterville High-Rise After Man Found Dead in Condo, Sources Say. *CBS Chicago* 2021. Available online: https://www.cbsnews.com/chicago/news/chicago-streeterville-hazmat-fbi-swat-team/ (accessed on 15 September 2024).
52. Aguilar, J. Bombs in Home Lead to Arrest. *Daily Camera* 2009. Available online: https://www.dailycamera.com/2009/08/14/bombs-in-home-lead-to-arrest/ (accessed on 15 September 2024).
53. Kurzer, F. Fulminic acid in the history of organic chemistry. *J. Chem. Educ.* **2000**, *77*, 851–857. https://doi.org/10.1021/ed077p851.
54. Beck, W.; Swoboda, P.; Feldl, K.; Tobias, R.S. Eigenschaften und IR-Spektrum der Knallsäure HCNO. *Chem. Ber.* **1971**, *104*, 533–543. https://doi.org/10.1002/cber.19711040223.
55. Wieland, H.; Heß, H. Die Polymerisation der Knallsäure. *Ber. Dtsch. Chem. Ges.* **1909**, *42*, 1346–1363. https://doi.org/10.1002/cber.190904201219.
56. Howard, E. On a new fulminating mercury. *Philos. Trans. R. Soc. Lond.* **1800**, *90*, 204–238. https://doi.org/10.1098/rstl.1800.0012.
57. Wöhler, L.; Weber, A. Neue Salze der Knallsäure. *Ber. Dtsch. Chem. Ges.* **1929**, *62*, 2742–2748. https://doi.org/10.1002/cber.19290621014.
58. Bos, W.; Bour, J.J.; Steggerda, J.J. Reactions of gold(I) compounds with carbon monoxide to form gold clusters and fulminates. *Inorg. Chem.* **1985**, *24*, 4298–4301. https://doi.org/10.1021/ic00219a020.
59. Steinhauser, G.; Evers, J.; Jakob, S.; Klapötke, T.M.; Oehlinger, G. A review on fulminating gold (Knallgold). *Gold Bull.* **2008**, *41*, 305–317. https://doi.org/10.1007/BF03214888.
60. Wurzenberger, M.H.H.; Gruhne, M.S.; Lommel, M.; Braun, V.; Szimhardt, N.; Stierstorfer, J. Taming the dragon: Complexation of silver fulminate with nitrogen-rich azole ligands. *Inorg. Chem.* **2020**, *59*, 17875–17879. https://doi.org/10.1021/acs.inorgchem.0c03027.
61. Hanley, R.U.S. Links Man With 3 Bombs To a Terror Plot. *New York Times* 4 February 1989, p. 30.
62. Bhowmick, S.; Kunte, S.S.; Bhowmick, K.C. A comprehensive study on the effect of acid additives in 1(*R*),2(*R*)-bis[(*S*)-prolinamido]cyclohexane catalyzed direct asymmetric aldol reactions in aqueous media. *Ind. J. Chem.* **2015**, *54B*, 84–92.
63. Pearce, P.J.; Simkins, R.J.J. Acid strengths of some substituted picric acids. *Can. J. Chem.* **1968**, *46*, 241–248. https://doi.org/10.1139/v68-038.
64. Khabarov, Y.G.; Patrakeev, A.A.; Veshnyakov, V.A.; Kosyakov, D.S.; Ul'yanovskii, N.V.; Garkotin, A.Y. One-step synthesis of picric acid from phenol. *Org. Prep. Proced. Int.* **2017**, *49*, 178–181. https://doi.org/10.1080/00304948.2017.1291008.
65. Matsukawa, M.; Matsunaga, T.; Yoshida, M.; Fujiwara, S. Synthesis and properties of lead picrates. *Sci. Technol. Energetic Mater.* **2004**, *65*, 7–13.
66. Wurzenberger, M.H.H.; Bissinger, B.R.G.; Lommel, M.; Gruhne, M.S.; Szimhardt, N.; Stierstorfer, J. Synthesis and comparison of copper(II) complexes with various *N*-aminotetrazole ligands involving trinitrophenol anions. *New J. Chem.* **2019**, *43*, 18193–18202. https://doi.org/10.1039/C9NJ03937F.
67. Wurzenberger, M.H.H.; Braun, V.; Lommel, M.; Klapötke, T.M.; Stierstorfer, J. Closing the gap: Synthesis of three isomeric *N,N*-ditetrazolymethane ligands and their coordination proficiency in adaptable laser responsive copper(II) and sensitive silver(I) complexes. *Inorg. Chem.* **2020**, *59*, 10938–10952. https://doi.org/10.1021/acs.inorgchem.0c01403.
68. Szimhardt, N.; Wurzenberger, M.H.H.; Zeisel, L.; Gruhne, M.S.; Lommel, M.; Klapötke, T.M.; Stierstorfer, J. 1-Amino-triazole transition metal complexes as laser ignitable and lead-free primary explosives. *Chem. Eur. J.* **2019**, *25*, 1963–1974. https://doi.org/10.1002/chem.201803372.
69. Kofen, M.; Harter, A.G.; Klapötke, T.M.; Stierstorfer, J. 1,5-Dimethyltetrazole as a ligand in energetic $3d^5$ to $3d^{10}$-metal coordination compounds. *Energ. Mat. Frontiers* **2022**, *3*, 111–121. https://doi.org/10.1016/j.enmf.2022.07.004.
70. Wurzenberger, M.H.H.; Endraß, S.M.J.; Lommel, M.; Klapötke, T.M.; Stierstorfer, J. Comparison of 1-Propyl-5*H*-tetrazole and 1-Azidopropyl-5*H*-tetrazole as Ligands for Laser Ignitable Energetic Materials. *ACS Appl. Energy Mater.* **2020**, *3*, 3798–3806. https://doi.org/10.1021/acsaem.0c00229.
71. Szimhardt, N.; Wurzenberger, M.H.H.; Beringer, A.; Daumann, L.J.; Stierstorfer, J. Coordination chemistry with 1-methyl-5*H*-tetrazole: Cocrystallization, laser-ignition, lead-free primary explosives—One ligand, three goals. *J. Mater. Chem. A* **2017**, *5*, 23753–23765. https://doi.org/10.1039/C7TA07780G.
72. Bi, Y.-G.; Feng, Y.-A.; Li, Y.; Wu, B.-D.; Zhang, T.-L. Synthesis, structure, and thermal decomposition of two copper coordination compounds $[Cu(DAT)_2(PA)_2]$ and $[Cu(DAT)_2(HTNR)_2]$ with nitrogen rich 1,5-diaminotetrazole (DAT). *J. Coord. Chem.* **2015**, *68*, 181–194. https://doi.org/10.1080/00958972.2014.981167.

73. Endraß, S.M.J.; Klapötke, T.M.; Lommel, M.; Stierstorfer, J.; Weidemann, M.L.; Werner, M. 1- and 2-Tetrazolylacetonitrile as versatile ligands for laser ignitable energetic coordination compounds. *ChemPlusChem* **2024**, *89*, e202400031. https://doi.org/10.1002/cplu.202400031.
74. Wu, B.-D.; Zhang, J.-G.; Zhang, T.-L.; Yang, L.; Zhou, Z.-N. Two environmentally friendly energetic compounds, $[Mn(AZT)_4(H_2O)_2](PA)_2{\cdot}4H_2O$ and $[Co(AZT)_2(H_2O)_4](PA)_2$, based on 3-azido-1,2,4-triazole (AZT) and picrate (PA). *Eur. J. Inorg. Chem.* **2012**, *2012*, 1261–1268. https://doi.org/10.1002/ejic.201100806.
75. Szimhardt, N.; Stierstorfer, J. Methylsemicarbazide as a ligand in late 3*d* transition metal complexes. *Chem. Eur. J.* **2018**, *24*, 2687–2698. https://doi.org/10.1002/chem.201705030.
76. Polis, M.; Wilk, Z.; Nikolczuk, K. The illegal synthesis of picric acid in the context of applicable legal regulations. *Materiały Wysokoenergetyczne* **2020**, *12*, 139–145. https://doi.org/10.22211/matwys/0202.
77. Zingaro, R.A. Lead salts of 2,4,6-trinitroresorcinol. *J. Am. Chem. Soc.* **1954**, *76*, 816–819. https://doi.org/10.1021/ja01632a054.
78. Pierce-Butler, M. Structures of the barium salt of 2,4,6-trinitro-1,3-benzenediol monohydrate and the isomorphous lead salt (β-polymorph). *Acta. Crystallogr. B* **1982**, *38*, 3100–3104. https://doi.org/10.1107/S0567740882010966.
79. Payne, J.R. Thermochemistry of lead styphnate. *Thermochim. Acta* **1994**, *242*, 13–21. https://doi.org/10.1016/0040-6031(94)85003-8.
80. Pierce-Butler, M. The structure of the monobasic lead salt of 2,4,6-trinitro-1,3-benzenediol (β polymorph), $Pb_2(OH)_2^{2+}{\cdot}C_6HN_3O_8^{2-}$. *Acta. Crystallogr. C* **1984**, *40*, 1364–1367. https://doi.org/10.1107/S0108270184007988.
81. Du, Z.-Y. Structure of barium 2,4,6-trinitroresorcinolate monohydrate. *Acta. Crystallogr. B* **1982**, *38*, 3095–3097. https://doi.org/10.1107/S0567740882010942.
82. Zheng, H.; Zhang, T.-L.; Zhang, J.-G.; Qiao, X.-J.; Yang, L.; Yu, K.-B. Synthesis, X-ray crystal structure and thermal decomposition mechanism of $[RbHTNR]_\infty$. *Chin. J. Chem* **2006**, *24*, 845–848. https://doi.org/10.1002/cjoc.200690161.
83. Li, Z.-M.; Zhang, T.-L.; Huang, H.-S.; Zhang, J.-G.; Yang, L.; Zhou, Z.-N.; Yu, K.-B. Syntheses, crystal structures, thermal behaviors, and sensitivities of new initiator compositions: Rubidium salts of trinitrophenol and trinitroresorcinol. *J. Coord. Chem.* **2014**, *67*, 1923–1937. https://doi.org/10.1080/00958972.2014.932353.
84. Payne, J.R. Thermochemistry of thallous styphnate. *Thermochim. Acta* **1994**, *237*, 229–234. https://doi.org/10.1016/0040-6031(94)80179-7.
85. Payne, J.R. Thermochemistry of silver styphnate. *Thermochim. Acta* **1994**, *242*, 7–12. https://doi.org/10.1016/0040-6031(94)85002-X.
86. Liu, J.-W.; Zhang, J.-G.; Zhang, T.-L.; Zheng, H.; Yang, L.; Yu, K.-B. A new hetero-bimetallic coordination polymer, cesium, and sodium complex of styphnate trihydrate $[CsNa(TNR)(H_2O)_3]_n$. *Struct. Chem.* **2009**, *20*, 387–392. https://doi.org/10.1007/s11224-009-9417-0.
87. Bley, U.; Hagel, R.; Hoschenko, A.; Lechner, P.S. Salts of Styphnic Acid. Patent Application No. WO2007071649A2, 18 December 2006.
88. Szimhardt, N.; Wurzenberger, M.H.H.; Klapötke, T.M.; Lechner, J.T.; Reichherzer, H.; Unger, C.C.; Stierstorfer, J. Highly functional energetic complexes: Stability tuning through coordination diversity of isomeric propyl-linked ditetrazoles. *J. Mater. Chem. A* **2018**, *6*, 6565–6577. https://doi.org/10.1039/C8TA01412D.
89. Gruhne, M.S.; Lenz, T.; Rösch, M.; Lommel, M.; Wurzenberger, M.H.H.; Klapötke, T.M.; Stierstorfer, J. Nitratoethyl-5*H*-tetrazoles: Improving the oxygen balance through application of organic nitrates in energetic coordination compounds. *Dalton Trans.* **2021**, *50*, 10811–10825. https://doi.org/10.1039/D1DT01898A.
90. Yang, L.; Tong, W.; Li, H.; Zhang, G.; Liu, J. Chelates with π-stacking and hydrogen-bonding interactions as safer and structurally reinforced energetic materials. *Inorg. Chim. Acta* **2017**, *466*, 405–409. https://doi.org/10.1016/j.ica.2017.06.071.
91. Zhang, G.-Y.; Yang, L.; Tong, W.-C.; Han, J.-M.; Song, N.-M. Cocrystallization of energetic Mn(II) complex with nitrogen-rich ligand SCZ and oxygen-rich ligand TNR. *J. Coord. Chem.* **2019**, *72*, 468–479. https://doi.org/10.1080/00958972.2019.1575962.
92. Tong, W.; Bian, M.; Feng, Y.; Zhang, T.; Hu, S.; Yang, L. A highly stable octa-coordinated energetic complex. *CrystEngComm* **2020**, *22*, 6591–6595. https://doi.org/10.1039/D0CE01008A.
93. Zhang, R.; Tong, W.-C.; Wang, Y.-L.; Liu, J.-C.; Yang, L. Seven-coordinated chelate $[Cd(SCZ)_3{\cdot}H_2O](HTNR)_2(H_2O)_2$: Synthesis, crystal structure and energetic properties. *Mol. Cryst. Liq. Cryst.* **2017**, *650*, 102–109. https://doi.org/10.1080/15421406.2017.1328226.
94. Staba, E.A. Crystalline Double Salt of Lead Nitroamnotetrazole and Lead Styphnate. U.S. Patent 3310569A, 21 March 1967.
95. Payne, J.R. Thermochemistry of lead 2,4- and 4,6-dinitroresorcinol. *Thermochim. Acta* **1995**, *265*, 73–87. https://doi.org/10.1016/0040-6031(95)02548-G.
96. Taylor, G.; Thomas, A.; Williams, R. Lead Compounds of 4:6-Dinitroresorcinol. U.S. Patent 3803190A, 09 April 1974.

97. Wang, L.; Chen, H.; Zhang, T.; Zhang, J.; Yang, L. Synthesis, characterization, thermal and explosive properties of potassium salts of trinitrophloroglucinol. *J. Hazard. Mater.* **2007**, *147*, 576–580. https://doi.org/10.1016/j.jhazmat.2007.01.043.
98. Chen, H.; Zhang, T.; Zhang, J.; Qiao, X.; Yu, K. Crystal structure, thermal decomposition mechanism and explosive properties of $[Na(H_2TNPG)(H_2O)_2]_n$. *J. Hazard. Mater.* **2006**, *129*, 31–36. https://doi.org/10.1016/j.jhazmat.2005.08.014.
99. Chen, H.; Zhang, T.; Zhang, J.; Chen, C. Crystal structure, thermal decomposition and properties of cesium 3,5-dihydroxy-2,4,6-trinitrophenolate. *Propell. Explos. Pyrot.* **2006**, *31*, 285–289. https://doi.org/10.1002/prep.200600038.
100. Endraß, S.M.J.; Neuer, A.; Klapötke, T.M.; Stierstorfer, J. Trinitro-orcinolate and trinitro-resorcinate—Sensitivity trends in nitroaromatic energetic materials. *ChemistrySelect* **2022**, *7*, e202203140. https://doi.org/10.1002/slct.202203140.
101. Drechsel, A.; Klapötke, T.M.; Witkowski, T.G. Synthesis and characterization of 5-methyl-2,4,6-trinitrobenzene-1,3-diol and its energetic cesium salt. *Jacobs J. Inorg. Chem.* **2016**, *1*, 008.
102. Rosenthal, U. Transition metal acetylides. In *Acetylene Chemistry*; Diederich, F., Stang, P.J., Tykwiński, R.R., Eds.; Wiley: Weinheim, Germany, 2004; pp. 139–171.
103. Nieuwland, J.A.; Maguire, J.A. Reactions of acetylene with acidified solutions of mercury and silver salts. *J. Am. Chem. Soc.* **1906**, *28*, 1025–1031. https://doi.org/10.1021/ja01974a009.
104. Shaw, J.; Fisher, E. New compounds. A new acetylene silver nitrate complex. *J. Am. Chem. Soc.* **1946**, *68*, 2745. https://doi.org/10.1021/ja01216a646.
105. Keiser, E.H. The composition of the explosive copper and silver compounds of acetylene. *Am. Chem. J.* **1892**, *14*, 285–290.
106. Guo, G.-C.; Zhou, G.-D.; Wang, Q.-G.; Mak, T.C.W. A fully encapsulated acetylenediide in $Ag_2C_2{\cdot}8\,AgF$. *Angew. Chem. Int. Ed.* **1998**, *37*, 630–632. https://doi.org/10.1002/(SICI)1521-3773(19980316)37:5%3C630::AID-ANIE630%3E3.0.CO;2-K.
107. Shaw, J.A.; Fisher, E. Silver Acetylide Compound and Process of Making Same. U.S. Patent 2483440A, 07 February 1946.
108. Berthelot, M. Ueber eine neue Klasse zusammengesetzter metallhaltiger Radicale. *Liebigs Ann. Chem.* **1866**, *139*, 245–253. https://doi.org/10.1002/jlac.18661390215.
109. Cataldo, F.; Casari, C.S. Synthesis, structure and thermal properties of copper and silver polyynides and acetylides. *J. Inorg. Organomet. Polym. Mater.* **2007**, *17*, 641–651. https://doi.org/10.1007/s10904-007-9150-3.
110. Taylor, C.A.; Rinkenbach, W.H. Sensitivities of detonating compounds to frictional impact, impact, and heat. *J. Franklin Inst.* **1927**, *204*, 369–376. https://doi.org/10.1016/S0016-0032(27)92103-6.
111. Pei, M.J.; Xu, H.B.; Wang, D.W.; Yao, W.B.; Yang, J.; Zhang, D.Z.; Zhang, J.S. Detonation characteristics of light-initiated explosive silver acetylide-silver nitrate. *Chin. J. High Press. Phys.* **2017**, *31*, 813–819. https://doi.org/10.11858/gywlxb.2017.06.017.
112. Xu, H.; Yang, J.; Wu, K.; Chen, B.; Sui, Y.; Wang, D. Simultaneous initiation of light-initiated explosive silver acetylide-silver nitrate. *Acta Armamentarii* **2022**, *43*, 2791–2797. https://doi.org/10.12382/bgxb.2021.0611.
113. Wang, D.; Li, J.; Zhang, Y.; Li, H.; Wang, S. Study on characteristics of the light-initiated high explosive-based pulse laser initiation. *Materials* **2022**, *15*, 4100. https://doi.org/10.3390/ma15124100.
114. Covert, T.T.; Chavez, M.A. Synthesis, Microstructure, and Explosive Properties of Spray-Deposited Silver Acetylide-Silver Nitrate Composite Light Initiated High Explosives; Report; Sandia National Laboratories: Albuquerque, New Mexico, 2013.
115. Guo, G.-D.; Zhou, G.-D.; Mak, T.C.W. Structural variation in novel double salts of silver acetylide with silver nitrate: Fully encapsulated acetylide dianion in different polyhedral silver cages. *J. Am. Chem. Soc.* **1999**, *121*, 3136–3141. https://doi.org/10.1021/ja984117n.
116. Boettger, R. Ueber die Einwirkung des Leuchtgases auf verschiedene Salzsolutionen, insbesondere auf eine ammoniakalische Kupferchlorürlösung. *Liebigs Ann. Chem.* **1859**, *109*, 351–362.
117. Michael, A. Ueber die Einwirkung von Diazobenzolimid auf Acetylendicarbonsäuremethylester. *J. Pract. Chem.* **1893**, *48*, 94–95.
118. Brameld, V.F.; Clark, M.T.; Seyfang, A.P. Copper acetylides. *J. Soc. Chem. Ind.* **1947**, *66*, 346–353. https://doi.org/10.1002/jctb.5000661007.
119. Jones, E.; Bowden, F.P. The ignition of solid explosive media by hot wires. *Proc. R. Soc. A Math. Phys. Sci.* **1949**, *198*, 523–539. https://doi.org/10.1098/rspa.1949.0117.
120. Sun, F.; Wang, G. Investigation of an accidental explosion caused by reaction runaway of a mixture containing copper acetylide and butynediol. *J. Loss Prev. Process Ind.* **2019**, *62*, 103967. https://doi.org/10.1016/j.jlp.2019.103967.
121. Miller, S.A.; Penny, E. Hazards in handling acetylene in chemical processes particularly under pressure. In Proceedings of the Symposium on Chemical Process Hazards with Special Reference to Plant Design,

Manchester, UK, 29–31 March, 1960; Institution of Chemical Engineers (Great Britain). North Western Branch: Manchester, UK, 1960; pp. 87–94.
122. Steikamp, F.L. Factors Influence the Safety of Unconventional Explosives. Ph.D. Thesis, University of Rhode Island, Kingston, RI, USA, 2013.
123. Cataldo, F.; Kanazirev, V. Synthesis and thermal stability of mercury diacetylide $Hg(CCH)_2$. *Polyhedron* **2013**, *62*, 42–50. https://doi.org/10.1016/j.poly.2013.06.005.
124. Cataldo, F. Structural relationships between dicopper diacetylide (Cu-C≡C-C≡C-Cu) and dicopper acetylide (Cu-C≡C-Cu). *Eur. J. Solid State Inorg. Chem.* **1998**, *35*, 281–291. https://doi.org/10.1016/S0992-4361(98)80009-X.
125. Endraß, S.M.J.; Klapötke, T.M.; Lechner, J.T.; Stierstorfer, J. Application of 1- and 2-propargyl-tetrazole in laser-ignitable energetic coordination compounds. *FirePhysChem* **2023**, *3*, 330–338. https://doi.org/10.1016/j.fpc.2023.03.005.
126. Goldman, D. *Improvised Primary Explosives*. Available online: https://miningandblasting.wordpress.com/wp-content/uploads/2009/09/primary-explosives.pdf (accessed on 10 October 2024).
127. Nagel, M.C. Dangerous demos. *J. Chem. Educ.* **1986**, *63*, 81–82. https://doi.org/10.1021/ed063p81.
128. Fleming, D. *Incendiary Silver. An Impressive Organometallic Explosive*. Available online: https://edu.rsc.org/exhibition-chemistry/incendiary-silver/2500473.article (accessed on 15 March 2023).
129. Spear, R.J.; Norris, W.P.; Read, R. *4,6-Dinitrobenzofuroxan, an Important Explosive Intermediate*; Technical Note; Department of Defence, Defence Science and Technology Organisation Materials Research Laboratories: Melbourne, Australia, 1983.
130. Fu, X.-L.; Fan, X.-Z.; Wang, B.-Z.; Huo, H.; Li, J.-Z.; Hu, R.-Z. Thermal behavior, decomposition mechanism and thermal safety of 5,7-diamino-4,6-dinitrobenzenfuroxan (CL-14). *J. Therm. Anal. Calorim.* **2016**, *124*, 993–1001. https://doi.org/10.1007/s10973-015-4992-3.
131. Nietzki, R.; Dietschy, R. Ueber Tetranitrobenzol, Dinitrosodinitrobenzol und Trinitrophenylhydroxylamin. *Ber. Dtsch. Chem. Ges.* **1901**, *34*, 55–60. https://doi.org/10.1002/cber.19010340112.
132. Millar, R.W. Lead-Free Initiator Materials for Small Electro-Explosive Devices for Medium Caliber Munitions; Report; SERDP & ESTCP: Farnborough, UK, 2003.
133. Fronabarger, J.; Williams, M.; Bichay, M. Environmentally acceptable alternatives to lead azide and lead styphnate. In Proceedings of the 43rd AIAA/ASME/SAE/ASEE Joint Propulsion Conference & Exhibit, Cincinnati, OH, USA, 8–11 July 2007; AIAA: Reston, VA, USA, 2007; p. 5132. https://doi.org/10.2514/6.2007-5132.
134. Chen, L.-Y.; Zhang, J.-G.; Zhou, Z.-N.; Zhang, T.-L. A biography of potassium complexes as versatile, green energetic materials. *RSC Adv.* **2016**, *6*, 98381–98405. https://doi.org/10.1039/C6RA22525J.
135. Norris, W.P.; Spear, R.J.; Read, R.W. Explosive Meisenheimer complexes formed by addition of nucleophilic reagents to 4,6-dinitrobenzofurazan 1-oxide. *Aust. J. Chem.* **1983**, *36*, 297–309. https://doi.org/10.1071/CH9830297.
136. Norris, W.P.; Osmundsen, J. 4,6-Dinitrobenzofuroxan. I. Covalent Hydration. *J. Org. Chem.* **1965**, *30*, 2407–2409. https://doi.org/10.1021/jo01018a072.
137. Brown, N.E.; Keyes, R.T. Structure of salts of 4,6-dinitrobenzofuroxan. *J. Org. Chem.* **1965**, *30*, 2452–2454. https://doi.org/10.1021/jo01018a503.
138. Sinditskii, V.P.; Egorshev, V.Y.; Serushkin, V.V.; Margolin, A.V.; Dong, H.W. Study on combustion of metal-derivatives of 4,6-dinitrobenzofuroxan. In Proceedings of the 4th International Autumn Seminar on Propellants, Explosives and Pyrotechnics, Shaoxing, China, 25–28 October 2001; Beijing Institute of Technology: Beijing, China, 2001; pp. 69–77.
139. Jones, D.E.G.; Lightfoot, P.D.; Fouchard, R.C.; Kwok, Q.; Turcotte, A.M.; Ridley, W. Hazard characterization of KDNBF using a variety of different techniques. *Thermochim. Acta* **2002**, *384*, 57–69. https://doi.org/10.1016/S0040-6031(01)00777-8.
140. Mehilal; Sikder, A.K.; Pawar, S.; Sikder, N. Synthesis, characterisation, thermal and explosive properties of 4,6-dinitrobenzofuroxan salts. *J. Hazard. Mater.* **2002**, *90*, 221–227. https://doi.org/10.1016/S0304-3894(01)00344-2.
141. Jones, D.E.G.; Feng, H.T.; Fouchard, R.C. Kinetic studies of the thermal decomposition of KDNBF, a primer for explosives. *J. Therm. Anal. Calorim.* **2000**, *60*, 917–926. https://doi.org/10.1023/A:1010167910410.
142. Zhang, T.L.; Zhang, J.-G. Thermal decomposition processes and non-isothermal kinetics of KDNBF. *Chin. J. Energ. Mat.* **2004**, *12*, 203–206.
143. Zahálka, M.; Pelikán, V.; Matyáš, R. Characterization and optimalization of electrostatic discharge (ESD) sensitiveness of potassium 4,6-dinitrobenzofuroxane. In Proceedings of the 21st Seminar on New Trends in Research of Energetic Materials, Pardubice, Czech Republic, 18–20 April 2018; pp. 1193–1197.
144. Feng, J.; Zhang, J.-G.; Zhang, T.L.; Li, Z.; Yang, L.; Wang, S. Morphology control and its influence on the decomposition behavior and sensitivity of KDNBF. *Acta Phys.-Chim. Sin.* **2010**, *26*, 2613–2618. https://doi.org/10.3866/PKU.WHXB20100950.

145. Zhang, T.L.; Miao, Y.L.; Zhang, J.-G. A new way to synthesize spherical KDNBF. *Chin. J. Explos. Propellants* **2003**, *26*, 53–56.
146. Li, J.-S.; Chen, F.-J.; Yang, H.-W.; Lu, K.-T. Study on synthesis and characterization of primary explosive KDNBF with different morphologies. *Propell. Explos. Pyrot.* **2020**, *45*, 1313–1325. https://doi.org/10.1002/prep.202000052.
147. Wang, S.; Zhang, T.; Yang, L.; Zhang, J.; Sun, Y. Synthesis, thermal decomposition and sensitivity study of CsDNBF. *Propell. Explos. Pyrot.* **2007**, *32*, 16–19. https://doi.org/10.1002/prep.200700003.
148. Lur'e, B.A.; Sinditskii, V.P.; Smirnov, S.P. Thermal decomposition of 2,4-dinitrobenzofuroxan and some of its compounds with metal hydroxides. *Combust. Explos. Shock Waves* **2003**, *39*, 534–543. https://doi.org/10.1023/A:1026157617792.
149. Mehilal; Sikder, N.; Chougule, S.K.; Sikder, A.K.; Gandhe, B.R. Synthesis, characterization, and thermal and explosive properties of alkali metal salts of 5,7-diamino-4,6-dinitrobenzofuroxan (CL-14). *J. Energ. Mater.* **2004**, *22*, 117–126. https://doi.org/10.1080/07370650490522767.
150. Shinde, P.D.; Mehilal; Salunke, R.B.; Agrawal, J.P. Some transition metal salts of 4,6-dinitrobenzofuroxan: Synthesis, characterization and evaluation of their properties. *Propell. Explos. Pyrot.* **2003**, *28*, 77–82. https://doi.org/10.1002/prep.200390012.
151. Spear, R.J.; Norris, W.P. Structure and properties of the potassium hydroxide-dinitrobenzofuroxan adduct (KDNBF) and related explosive salts. *Propell. Explos. Pyrot.* **1983**, *8*, 85–88. https://doi.org/10.1002/prep.830080308.
152. Boulton, A.J.; Clifford, D.P. 1006. Two explosive compounds: The potassium salt of 4,6-dinitro-benzofuroxan, and 3,4-dimethyl-4-(3,4-dimethyl-5-isoxazolylazo)isox-azolin-5-one. *J. Chem. Soc.* **1965**, 5414–5416. https://doi.org/10.1039/JR9650005414.
153. Read, R.; Spear, R.; Norris, W. Synthesis of 4,6-dinitrobenzofurazan, a new electron-deficient aromatic. *Aust. J. Chem.* **1983**, *36*, 1227–1237. https://doi.org/10.1071/CH9831227.
154. Whelan, D.J.; Spear, R.J.; Read, R.W. The thermal decomposition of some primary explosives as studied by differential scanning calorimetry. *Thermochim. Acta* **1984**, *80*, 149–163. https://doi.org/10.1016/0040-6031(84)87193-2.
155. Norris, W.P. Primary Explosive. U.S. Patent 4529801, 16 July 1985.
156. Terrier, F.; Halle, J.C.; Simonnin, M.P.; Pouet, M.J. Nonconventional electrophilic heteroaromatic substitutions: Ring vs. side-chain reactivity of 2,5-dimethyl five-membered ring heterocycles toward electron-deficient aromatics. *J. Org. Chem.* **1984**, *49*, 4363–4367. https://doi.org/10.1021/jo00197a007.
157. Buncel, E.; Renfrow, R.A.; Strauss, M.J. Ambident nucleophilic reactivity in .sigma.-complex formations. 6. Reactivity-selectivity relationships in reactions of ambident nucleophiles with the superelectrophiles 4,6-dinibrobenzofuroxan and 4,6-dinitro-2-(2,4,6-trinitrophenyl) benzotriazole 1-oxide. *J. Org. Chem.* **1987**, *52*, 488–495. https://doi.org/10.1021/jo00380a003.
158. Terrier, F.; Simonnin, M.P.; Pouet, M.J.; Strauss, M.J. Reactivity of carbon acids toward 4,6-dinitrobenzofuroxan. Studies of keto-enol equilibriums and diastereoisomerism in carbon-bonded anionic σ complexes. *J. Org. Chem.* **1981**, *46*, 3537–3543. https://doi.org/10.1021/jo00330a033.
159. Sleadd, B.A.; Fronabarger, J. 3-Azido-2,4,6-Trinitrophenol, Method of Making, and Method of Transforming. U.S. Patent 8748639, 2014.
160. Carter, G.B. Composition Pour Appret Primer Composition. Patent Application No. CA2156974, 28 February 1996.
161. Zhang, S.; Xie, Z.; Chen, L.; Dang, P. Preparation of 4,6-dinitro-7-oxygen-benzofuraxan sodium salt (NaDNP) by "one-pot" method. *J. Phys. Conf. Ser.* **2022**, *2239*, 012024. https://doi.org/10.1088/1742-6596/2239/1/012024.
162. Fronabarger, J.; Williams, M.; Sanborn, W.; Sitzmann, M.; Bichay, M.; Gilardi, R. Characterization and output testing of the novel primary explosive, bis(furoxano)nitrophenol, potassium salt. In Proceedings of the 41st AIAA/ASME/SAE/ASEE Joint Propulsion Conference and Exhibit, Tuscon, AZ, USA, 10–13 July 2005; AIAA: Reston, VA, USA, 2005; p. 3512. https://doi.org/10.2514/6.2005-3512.
163. Sitzmann, M.E.; Bichay, M.; Fronabarger, J.W.; Williams, M.D.; Sanborn, W.B.; Gilardi, R. Hydroxynitrobenzodifuroxan and its salts. *J. Heterocycl. Chem.* **2005**, *42*, 1117–1125. https://doi.org/10.1002/jhet.5570420613.
164. Fronabarger, J.; Sitzmann, M. Nitrobenzodifuroxan Compounds, Including Their Salts, and Methods Thereof. U.S. Patent 7271267B1, 18 September 2007.
165. Zhang, Y.F.; Sheng, D.L.; Ma, F.E.; Zhu, Y.H.; Yang, B. New primary explosive bis-furoxano-nitrophenol potassium salt. *Chin. J. Energ. Mat.* **2007**, *15*, 600–603. https://doi.org/10.1016/S1001-6058(07)60030-4.
166. Terrier, F.; Lakhdar, S.; Boubaker, T.; Goumont, R. Ranking the reactivity of superelectrophilic heteroaromatics on the electrophilicity scale. *J. Org. Chem.* **2005**, *70*, 6242–6253. https://doi.org/10.1021/jo0505526.
167. He, C.; Shreeve, J.M. Potassium 4,5-bis(dinitromethyl)furoxanate: A green primary explosive with a positive oxygen balance. *Angew. Chem. Int. Ed.* **2016**, *55*, 772–775. https://doi.org/10.1002/anie.201509209.

168. Guo, T.; Wang, Z.; Tang, W.; Wang, W.; Bi, F.; Wang, B.; Zhou, Z.; Meng, Z.; Ge, Z. A good balance between the energy density and sensitivity from assembly of bis(dinitromethyl) and bis(fluorodinitromethyl) with a single furazan ring. *J. Anal. Appl. Pyrolysis* **2018**, *134*, 218–230. https://doi.org/10.1016/j.jaap.2018.06.012.
169. Tang, Y.; He, C.; Mitchell, L.A.; Parrish, D.A.; Shreeve, J.M. Potassium 4,4′-bis(dinitromethyl)-3,3′-azofurazanate: A highly energetic 3D metal–organic framework as a promising primary explosive. *Angew. Chem. Int. Ed.* **2016**, *55*, 5565–5567. https://doi.org/10.1002/anie.201601432.
170. Guo, D.; An, Q. Thermal stability and detonation properties of potassium 4,4′-bis(dinitromethyl)-3,3′-azofurazanate, an environmentally friendly energetic three-dimensional metal–organic framework. *ACS Appl. Mater. Interfaces* **2019**, *11*, 1512–1519. https://doi.org/10.1021/acsami.8b19611.
171. Zhai, L.; Fan, X.; Wang, B.; Bi, F.; Li, Y.; Zhu, Y. A green high-initiation-power primary explosive: Synthesis, 3D structure and energetic properties of dipotassium 3,4-bis(3-dinitromethylfurazan-4-oxy)furazan. *RSC Adv.* **2015**, *5*, 57833–57841. https://doi.org/10.1039/C5RA09822J.
172. Li, Y.; Huang, H.; Shi, Y.; Yang, J.; Pan, R.; Lin, X. Potassium nitraminofurazan derivatives: Potential green primary explosives with high energy and comparable low friction sensitivities. *Chem. Eur. J.* **2017**, *23*, 7353–7360. https://doi.org/10.1002/chem.201700739.
173. Fischer, D.; Klapötke, T.M.; Reymann, M.; Stierstorfer, J.; Völkl, M.B.R. Energetic alliance of tetrazole-1-oxides and 1,2,5-oxadiazoles. *New J. Chem.* **2015**, *39*, 1619–1627. https://doi.org/10.1039/C4NJ01351D.
174. Larin, A.A.; Muravyev, N.V.; Pivkina, A.N.; Suponitsky, K.Y.; Ananyev, I.V.; Khakimov, D.V.; Fershtat, L.L.; Makhova, N.N. Assembly of tetrazolylfuroxan organic salts: Multipurpose green energetic materials with high enthalpies of formation and excellent detonation performance. *Chem. Eur. J.* **2019**, *25*, 4225–4233. https://doi.org/10.1002/chem.201806378.
175. Guo, T.; Wang, Z.; Huo, H.; Tang, W.; Zhu, Y.; Bi, F.; Wang, B.; Meng, Z.; Ge, Z. An efficient method of preparation and comprehensive properties for energetic salts based on nitrofurazan-functionalized hydroxytetrazoles. *ChemistrySelect* **2018**, *3*, 11835–11841. https://doi.org/10.1002/slct.201802791.
176. Liu, Y.; Yi, P.; Gong, L.; Yi, X.; He, P.; Wang, T.; Zhang, J. Three-dimensional metal–organic frameworks as super heat-resistant explosives: Potassium 4,4′-oxybis [3,3′-(5-tetrazol)]furazan and potassium (1,2,4-triazol-3-yl)tetrazole. *Inorg. Chem.* **2023**, *62*, 3186–3194. https://doi.org/10.1021/acs.inorgchem.2c04108.
177. Tang, Y.; He, C.; Imler, G.H.; Parrish, D.A.; Shreeve, J.M. Energetic 4,4′-oxybis [3,3′-(1-hydroxytetrazolyl)]furazan and its salts. *Chem. Asian J.* **2016**, *11*, 3113–3117. https://doi.org/10.1002/asia.201601144.
178. Zhai, L.; Qu, X.; Wang, B.; Bi, F.; Chen, S.; Fan, X.; Xie, G.; Wei, Q.; Gao, S. High energy density materials incorporating 4,5-bis(dinitromethyl)-furoxanate and 4,5-bis(dinitromethyl)-3-oxy-furoxanate. *ChemPlusChem* **2016**, *81*, 1156–1159. https://doi.org/10.1002/cplu.201600287.
179. Qu, X.-N.; Zhang, S.; Wang, B.-Z.; Yang, Q.; Han, J.; Wei, Q.; Xie, G.; Chen, S.-P. An Ag(I) energetic metal–organic framework assembled with the energetic combination of furazan and tetrazole: Synthesis, structure and energetic performance. *Dalton Trans.* **2016**, *45*, 6968–6973. https://doi.org/10.1039/C6DT00218H.
180. Hao, W.; Jin, B.; Zhang, J.; Li, X.; Huang, T.; Shen, J.; Peng, R. Novel energetic metal–organic frameworks assembled from the energetic combination of furazan and tetrazole. *Dalton Trans.* **2020**, *49*, 6295–6301. https://doi.org/10.1039/D0DT00862A.
181. Shen, C.; Liu, Y.; Zhu, Z.-q.; Xu, Y.-g.; Lu, M. Self-assembly of silver(i)-based high-energy metal–organic frameworks (HE-MOFs) at ambient temperature and pressure: Synthesis, structure and superior explosive performance. *Chem. Commun.* **2017**, *53*, 7489–7492. https://doi.org/10.1039/C7CC03869K.
182. Huang, T.; Jin, B.; Hao, W.; Zhang, J.; Luo, L.; Peng, H.; Hou, C.; Zhang, Q.; Peng, R. Combination of 3-aminofurazan-4-carboxylic acid and transition metals to prepare functional energetic catalysts for catalyzing the decomposition of ammonium perchlorate. *Cryst. Growth Des.* **2022**, *22*, 5802–5813. https://doi.org/10.1021/acs.cgd.1c01502.
183. Sun, Q.; Liu, Y.; Li, X.; Lu, M.; Lin, Q. Alkali metals-based energetic coordination polymers as promising primary explosives: Crystal structures, energetic properties, and environmental impact. *Chem. Eur. J.* **2018**, *24*, 14213–14219. https://doi.org/10.1002/chem.201802494.
184. Fischer, D.; Klapötke, T.M.; Reymann, M.; Stierstorfer, J. Dense energetic nitraminofurazanes. *Chem. Eur. J.* **2014**, *20*, 6401–6411. https://doi.org/10.1002/chem.201400362.
185. Gospodinov, I.; Hermann, T.; Klapötke, T.M.; Stierstorfer, J. Energetic compounds based on 3,4-bis(4-nitramino- 1,2,5-oxadiazol-3-yl)-1,2,5-furoxan (BNAFF). *Propell. Explos. Pyrot.* **2018**, *43*, 355–363. https://doi.org/10.1002/prep.201700289.
186. Chinnam, A.K.; Staples, R.J.; Shreeve, J.M. Construction of highly thermostable and insensitive three-dimensional energetic salts based on [1,2,5]oxadiazolo [3,4-*d*]pyrimidine. *Org. Lett.* **2022**, *24*, 7544–7548. https://doi.org/10.1021/acs.orglett.2c02889.
187. Fershtat, L.L.; Makhova, N.N. 1,2,5-Oxadiazole-based high-energy-density materials: Synthesis and performance. *ChemPlusChem* **2020**, *85*, 13–42. https://doi.org/10.1002/cplu.201900542.

188. Wang, L.; Zhai, L.; She, W.; Wang, M.; Zhang, J.; Wang, B. Synthetic strategies toward nitrogen-rich energetic compounds via the reaction characteristics of cyanofurazan/furoxan. *Front. Chem.* **2022**, *10*, e871684. https://doi.org/10.3389/fchem.2022.871684.
189. Benson, F.R. The chemistry of the tetrazoles. *Chem. Rev.* **1947**, *41*, 1–61. https://doi.org/10.1021/cr60128a001.
190. von Pechmann, H.; Wedekind, E. Ueber die Constitution der Tetrazoliumbasen. *Ber. Dtsch. Chem. Ges.* **1895**, *28*, 1688–1695. https://doi.org/10.1002/cber.189502802106.
191. Damavarapu, R.; Klapötke, T.M.; Stierstorfer, J.; Tarantik, K.R. Barium salts of tetrazole derivatives—Synthesis and characterization. *Propell. Explos. Pyrot.* **2010**, *35*, 395–406. https://doi.org/10.1002/prep.200900058.
192. Beck, W.; Burger, K.; Fehlhammer, W.P. Zur Reaktion von Azido-Metallverbindungen mit Isonitrilen: Tetrazolato-Komplexe mit Metall-Kohlenstoff-Bindung. *Chem. Ber.* **1971**, *104*, 1816–1825. https://doi.org/10.1002/cber.19711040617.
193. Fischer, D.; Klapötke, T.M.; Piercey, D.G.; Stierstorfer, J. Copper salts of halo tetrazoles: Laser-ignitable primary explosives. *J. Energ. Mater.* **2012**, *30*, 40–54. https://doi.org/10.1080/07370652.2010.539998.
194. Stollé, R. Zur Kenntnis des Amino-5-tetrazols. *Ber. Dtsch. Chem. Ges.* **1929**, *62*, 1118–1126. https://doi.org/10.1002/cber.19290620505.
195. Feng, Y.; Liu, X.; Duan, L.; Yang, Q.; Wei, Q.; Xie, G.; Chen, S.; Yang, X.; Gao, S. In situ synthesized 3D heterometallic metal–organic framework (MOF) as a high-energy-density material shows high heat of detonation, good thermostability and insensitivity. *Dalton Trans.* **2015**, *44*, 2333–2339. https://doi.org/10.1039/C4DT03131H.
196. Klapötke, T.M.; Stein, M.; Stierstorfer, J. Salts of 1*H*-tetrazole—Synthesis, characterization and properties. *Z. Anorg. Allg. Chem.* **2008**, *634*, 1711–1723. https://doi.org/10.1002/zaac.200800139.
197. Miró Sabaté, C.; Jeanneau, E.; Stierstorfer, J. Synthesis and comprehensive characterization of hydrated alkaline earth metal salts of 5-amino-1*H*-tetrazole. *Z. Anorg. Allg. Chem.* **2011**, *637*, 1490–1501. https://doi.org/10.1002/zaac.201100193.
198. Ernst, V.; Klapötke, T.M.; Stierstorfer, J. Alkali salts of 5-aminotetrazole—Structures and properties. *Z. Anorg. Allg. Chem.* **2007**, *633*, 879–887. https://doi.org/10.1002/zaac.200700013.
199. Klapötke, T.M.; Sabaté, C.M.; Stierstorfer, J. Neutral 5-nitrotetrazoles: Easy initiation with low pollution. *New J. Chem.* **2009**, *33*, 136–147. https://doi.org/10.1039/B812529E.
200. Klapötke, T.M.; Sabaté, C.M.; Welch, J.M. Alkali metal 5-nitrotetrazolate salts: Prospective replacements for service lead(II) azide in explosive initiators. *Dalton Trans.* **2008**, *45*, 6372–6380. https://doi.org/10.1039/B811410B.
201. Klapötke, T.M.; Sabaté, C.M.; Welch, J.M. Alkaline earth metal salts of 5-nitro-2*H*-tetrazole: Prospective candidates for environmentally friendly energetic applications. *Eur. J. Inorg. Chem.* **2009**, *2009*, 769–776. https://doi.org/10.1002/ejic.200801080.
202. Bates, L.R.; Jenkins, J.M. Production of 5-Netrotetrazole Salts. U.S. Patent 4094879, 13 Juny 1978.
203. von Herz, E. C-Nitrotetrazole Compounds. U.S. Patent 2066954, 05 January 1937.
204. Jenkins, J.M.; White, J.R. The Salts of 5-Substituted Tetrazoles: Part 3: Further Investigations into the Preparation and Properties of Silver 5-Nitrotetrazole and Mercuric 5-Nitrotetrazole; Report; Defence Research Information Centre: Orpington, UK, 1974.
205. Gilligan, W.H.; Kamlet, M.J. *Synthesis of Mercuric 5-Nitrotetrazole*; Report; Naval Surface Weapons Center: White Oak, TX, USA, 1976.
206. Talawar, M.B.; Chhabra, J.S.; Agrawal, A.P.; Asthana, S.N.; Rao, K.U.B.; Singh, H. Synthesis, characterization, thermolysis and performance evaluation of mercuric-5-nitrotetrazole (MNT). *J. Hazard. Mater.* **2004**, *113*, 27–33. https://doi.org/10.1016/j.jhazmat.2004.06.001.
207. Klapötke, T.; Sabaté, C. Less sensitive transition metal salts of the 5-nitrotetrazolate anion. *Cent. Eur. J. Energ. Mater.* **2010**, *7*, 161–173.
208. Li, W.-H.; Tseng, K.-C.; Yang, T.-M.; Li, J.-S.; Lu, K.-T. Optimization of synthesis parameters and characterization of green primary explosive copper(I) 5-nitrotetrazolate (DBX-1). *Propell. Explos. Pyrot.* **2020**, *45*, 1831–1840. https://doi.org/10.1002/prep.202000173.
209. Yu, C.; Gu, B.; Wang, J.; Chen, J.; Zhang, W.; Shi, W.; Yang, G.; Lei, X.; Zhu, J. Valence-oriented electrosynthesis strategies of Cu-based 5-nitrotetrazolate for environmentally acceptable primary explosives. *Inorg. Chem.* **2022**, *61*, 19379–19387. https://doi.org/10.1021/acs.inorgchem.2c03183.
210. Tappan, A.S.; Ball, J.P.; Miller, J.C. DBX-1 (copper(I)-5-nitrotetrazolate) reactions at sub-millimeter diameters. In Proceedings of the 39th International Pyrotechnics Seminar, Valencia, Spain, 27–31 May 2013.
211. Fronabarger, J.W.; Williams, M.D.; Sanborn, W.B.; Bragg, J.G.; Parrish, D.A.; Bichay, M. DBX-1—A lead free replacement for lead azide. *Propell. Explos. Pyrot.* **2011**, *36*, 541–550. https://doi.org/10.1002/prep.201100056.
212. Klapötke, T.M.; Piercey, D.G.; Mehta, N.; Oyler, K.D.; Jorgensen, M.; Lenahan, S.; Salan, J.S.; Fronabarger, J.W.; Williams, M.D. Preparation of high purity sodium 5-nitrotetrazolate (NaNT): An essential precursor to the

environmentally acceptable primary explosive, DBX-1. *Z. Anorg. Allg. Chem.* **2013**, *639*, 681–688. https://doi.org/10.1002/zaac.201300010.
213. Bates, L.R.; Jenkins, J.M. Search for new detonators. In Proceedings of the International Conference on Research in Primary Explosives, 17–19 March 1975; Waltham Abbey, UK; ERDE: Waltham Abbey, UK, 1975; pp. 14/11–14/18.
214. Huynh, M.H.V.; Hiskey, M.A.; Meyer, T.J.; Wetzler, M. Green primaries: Environmentally friendly energetic complexes. *Proc. Natl. Acad. Sci. USA* **2006**, *103*, 5409–5412. https://doi.org/10.1073/pnas.0600827103.
215. Huynh, M.H.V.; Coburn, M.D.; Meyer, T.J.; Wetzler, M. Green primary explosives: 5-Nitrotetrazolato-N^2-ferrate hierarchies. *Proc. Natl. Acad. Sci. USA* **2006**, *103*, 10322–10327. https://doi.org/10.1073/pnas.0604241103.
216. Hiskey, M.A.; Huynh, M.H.V. Primary Explosives. U.S. Patent 20060030715A1, 09 February 2006.
217. Arp, H.P.H.; Decken, A.; Passmore, J.; Wood, D.J. Preparation, characterization, x-ray crystal structure, and energetics of cesium 5-cyano-1,2,3,4-tetrazolate: Cs[NCCNNNN]. *Inorg. Chem.* **2000**, *39*, 1840–1848. https://doi.org/10.1021/ic990828q.
218. Crawford, M.-J.; Klapötke, T.M.; Martin, F.A.; Miró Sabaté, C.; Rusan, M. Energetic salts of the binary 5-cyanotetrazolate anion ($[C_2N_5]^-$) with nitrogen-rich cations. *Chem. Eur. J.* **2011**, *17*, 1683–1695. https://doi.org/10.1002/chem.201002161.
219. Fischer, N.; Klapötke, T.M.; Rappenglück, S.; Stierstorfer, J. The reactivity of 5-cyanotetrazole towards water and hydroxylamine. *ChemPlusChem* **2012**, *77*, 877–888. https://doi.org/10.1002/cplu.201200136.
220. Hartdegen, V.; Klapötke, T.M.; Sproll, S.M. Tetrazole-5-carboxylic acid based salts of earth alkali and transition metal cations. *Inorg. Chem.* **2009**, *48*, 9549–9556. https://doi.org/10.1021/ic901413n.
221. Klapötke, T.M.; Stierstorfer, J. The CN_7^- anion. *J. Am. Chem. Soc.* **2009**, *131*, 1122–1134. https://doi.org/10.1021/ja8077522.
222. Hofmann, K.A.; Hock, H.; Roth, R. Diazoverbindungen aus Amidoguanidin, Beiträge zur Kenntnis der Diazohydrazoverbindungen (Tetrazene). *Ber. Dtsch. Chem. Ges.* **1910**, *43*, 1087–1095.
223. Herbst, R.M.; Garrison, J.A. The nitration of 5-aminotetrazole. *J. Org. Chem.* **1953**, *18*, 941–945. https://doi.org/10.1021/jo50014a007.
224. O'Connor, T.E.; Fleming, G.; Reilly, J. Diazotization of nitroaminoguanidine. *J. Soc. Chem. Ind.* **1949**, *68*, 309–310. https://doi.org/10.1002/jctb.5000681101.
225. Palopoli, S.F.; Geib, S.J.; Rheingold, A.L.; Brill, T.B. Synthesis and modes of coordination of energetic nitramine ligands in copper(II), nickel(II), and palladium(II) complexes. *Inorg. Chem.* **1988**, *27*, 2963–2971. https://doi.org/10.1021/ic00290a015.
226. Ni, D.; Liu, J.; Yuxin, J.; Yang, B.; Yu, G. Barium 5-nitriminotetrazolate—A powerful primary explosive with resistance high temperature properties. *J. Phys. Conf. Ser.* **2022**, *2239*, 012022. https://doi.org/10.1088/1742-6596/2239/1/012022.
227. Klapötke, T.M.; Stierstorfer, J.; Tarantik, K.R.; Thoma, I.D. Strontium nitriminotetrazolates—Suitable colorants in smokeless pyrotechnic compositions. *Z. Anorg. Allg. Chem.* **2008**, *634*, 2777–2784. https://doi.org/10.1002/zaac.200800171.
228. Fischer, N.; Klapötke, T.M.; Stierstorfer, J. Calcium 5-nitriminotetrazolate—A green replacement for lead azide in priming charges. *J. Energ. Mater.* **2011**, *29*, 61–74. https://doi.org/10.1080/07370652.2010.505939.
229. Klapötke, T.M.; Stierstorfer, J.; Weber, B. New energetic materials: Synthesis and characterization of copper 5-nitriminotetrazolates. *Inorg. Chim. Acta* **2009**, *362*, 2311–2320. https://doi.org/10.1016/j.ica.2008.10.014.
230. Geisberger, G.; Klapötke, T.M.; Stierstorfer, J. Copper bis(1-methyl-5-nitriminotetrazolate): A promising new primary explosive. *Eur. J. Inorg. Chem.* **2007**, *2007*, 4743–4750. https://doi.org/10.1002/ejic.200700395.
231. Klapötke, T.M.; Stierstorfer, J.; Wallek, A.U. Nitrogen-rich salts of 1-methyl-5-nitriminotetrazolate: An auspicious class of thermally stable energetic materials. *Chem. Mater.* **2008**, *20*, 4519–4530. https://doi.org/10.1021/cm8004166.
232. Klapötke, T.M.; Radies, H.; Stierstorfer, J. Alkali salts of 1-methyl-5-nitriminotetrazole—Structures and properties. *Z. Naturforsch. B* **2007**, *62*, 1343–1352. https://doi.org/10.1515/znb-2007-1101.
233. Benz, M.; Klapötke, T.M.; Lenz, T.; Stierstorfer, J. Tuning the properties of 5-azido and 5-nitramino-tetrazoles by diverse functionalization—General concepts for future energetic materials. *Chem. Eur. J.* **2022**, *28*, e202200772. https://doi.org/10.1002/chem.202200772.
234. Benz, M.; Klapötke, T.M.; Stierstorfer, J. 1-Nitrimino-5-azidotetrazole: Extending energetic tetrazole chemistry. *ChemPlusChem* **2022**, *87*, e202200186. https://doi.org/10.1002/cplu.202200186.
235. Ma, X.; Cai, C.; Sun, W.; Song, W.; Ma, Y.; Liu, X.; Xie, G.; Chen, S.; Gao, S. Enhancing energetic performance of multinuclear Ag(I)-cluster MOF-based high-energy-density materials by thermal dehydration. *ACS Appl. Mater. Interfaces* **2019**, *11*, 9233–9238. https://doi.org/10.1021/acsami.9b00834.
236. Huang, W.; Yin, Z.; Dong, Y.; Liu, Y.; Tang, Y. An energetic coordination polymer with high thermal stability and initiation power. *Propell. Explos. Pyrot.* **2022**, *47*, e202200011. https://doi.org/10.1002/prep.202200011.

237. Yin, Z.; Huang, W.; Tang, Y. Synthesis and properties of lead-free primary explosive: Potassium 5-(2,2-diamino-1-nitrovinyl)tetrazolate. *Propell. Explos. Pyrot.* **2021**, *46*, 1150–1154. https://doi.org/10.1002/prep.202100079.
238. Fischer, D.; Klapötke, T.M.; Stierstorfer, J. 1,5-Di(nitramino)tetrazole: High sensitivity and superior explosive performance. *Angew. Chem. Int. Ed.* **2015**, *54*, 10299–10302. https://doi.org/10.1002/anie.201502919.
239. Klapötke, T.M.; Martin, F.A.; Stierstorfer, J. *N*-Bound primary nitramines based on 1,5-diaminotetrazole. *Chem. Eur. J.* **2012**, *18*, 1487–1501. https://doi.org/10.1002/chem.201102142.
240. Li, Y.; Yu, T.; Zhang, Y.; Hu, J.; Chen, T.; Wang, Y.; Xu, K. Novel energetic coordination polymers based on 1,5-di(nitramino)tetrazole with high oxygen content and outstanding properties: Syntheses, crystal structures, and detonation properties. *Front. Chem.* **2019**, *7*, e672. https://doi.org/10.3389/fchem.2019.00672.
241. Benz, M.; Klapötke, T.M.; Stierstorfer, J. 1-Nitramino-5-aminotetrazole—A simple accessible highly energetic building block. *Energetic Materials Frontiers* **2022**, *3*, 161–165. https://doi.org/10.1016/j.enmf.2022.08.003.
242. Szimhardt, N.; Wurzenberger, M.H.H.; Spieß, P.; Klapötke, T.M.; Stierstorfer, J. Potassium *N*-nitramino-5*H*-tetrazolates—Powerful green primary explosives with high initiation capabilities. *Propell. Explos. Pyrot.* **2018**, *43*, 1203–1209. https://doi.org/10.1002/prep.201800221.
243. Joas, M.; Klapötke, T.M.; Stierstorfer, J.; Szimhardt, N. Synthesis and characterization of various photosensitive copper(II) complexes with 5-(1-methylhydrazinyl)-1*H*-tetrazole as ligand and perchlorate, nitrate, dinitramide, and chloride as anions. *Chem. Eur. J.* **2013**, *19*, 9995–10003. https://doi.org/10.1002/chem.201300688.
244. Tao, G.-H.; Parrish, D.A.; Shreeve, J.M. Nitrogen-rich 5-(1-methylhydrazinyl)tetrazole and its copper and silver complexes. *Inorg. Chem.* **2012**, *51*, 5305–5312. https://doi.org/10.1021/ic300242e.
245. Stierstorfer, J.; Tarantik, K.R.; Klapötke, T.M. New energetic materials: Functionalized 1-ethyl-5-aminotetrazoles and 1-ethyl-5-nitriminotetrazoles. *Chem. Eur. J.* **2009**, *15*, 5775–5792. https://doi.org/10.1002/chem.200802203.
246. Fischer, N.; Klapötke, T.M.; Peters, K.; Rusan, M.; Stierstorfer, J. Alkaline earth metal salts of 5,5′-bistetrazole—From academical interest to practical application. *Z. Anorg. Allg. Chem.* **2011**, *637*, 1693–1701. https://doi.org/10.1002/zaac.201100263.
247. Joas, M.; Klapötke, T.M.; Stierstorfer, J. Preparation and crystal structure of diaqua(μ-5,5′-bistetrazolato-$\kappa^4 N^1,N^2,N^5,N^6$)copper(II). *Crystals* **2012**, *2*, 958–966. https://doi.org/10.3390/cryst2030958.
248. Fischer, N.; Izsák, D.; Klapötke, T.M.; Stierstorfer, J. The chemistry of 5-(tetrazol-1-yl)-2*H*-tetrazole: An extensive study of structural and energetic properties. *Chem. Eur. J.* **2013**, *19*, 8948–8957. https://doi.org/10.1002/chem.201300691.
249. Wang, B.; Qi, X.; Zhang, W.; Wang, K.; Li, W.; Zhang, Q. Synthesis of 1-(2*H*-tetrazol-5-yl)-5-nitraminotetrazole and its derivatives from 5-aminotetrazole and cyanogen azide: A promising strategy towards the development of C–N linked bistetrazolate energetic materials. *J. Mater. Chem. A* **2017**, *5*, 20867–20873. https://doi.org/10.1039/C7TA05905A.
250. Guo, Y.; Gao, H.; Twamley, B.; Shreeve, J.M. Energetic nitrogen rich salts of *N*,*N*-bis [1(2)*H*-tetrazol-5-yl]amine. *Adv. Mater.* **2007**, *19*, 2884–2888. https://doi.org/10.1002/adma.200602647.
251. Friedrich, M.; Gálvez-Ruiz, J.C.; Klapötke, T.M.; Mayer, P.; Weber, B.; Weigand, J.J. BTA copper complexes. *Inorg. Chem.* **2005**, *44*, 8044–8052. https://doi.org/10.1021/ic050657r.
252. Liu, Q.; Jin, B.; Zhang, Q.; Shang, Y.; Guo, Z.; Tan, B.; Peng, R. Nitrogen-rich energetic metal-organic framework: Synthesis, structure, properties, and thermal behaviors of Pb(II) complex based on *N*,*N*-bis(1*H*-tetrazole-5-yl)-amine. *Materials* **2016**, *9*, e681. https://doi.org/10.3390/ma9080681.
253. Li, F.; Zhao, W.; Chen, S.; Zhang, T.; Zhou, Z.; Yang, L. Nitrogen-rich alkali metal salts (Na and K) of [bis(*N*,*N*-bis(1*H*-tetrazol-5-yl)amine)-zinc(II)] anion: Syntheses, crystal structures, and energetic properties. *Z. Anorg. Allg. Chem.* **2015**, *641*, 911–916. https://doi.org/10.1002/zaac.201500078.
254. Hammerl, A.; Holl, G.; Klapötke, T.M.; Mayer, P.; Nöth, H.; Piotrowski, H.; Warchhold, M. Salts of 5,5′-azotetrazolate. *Eur. J. Inorg. Chem.* **2002**, *2002*, 834–845. https://doi.org/10.1002/1099-0682(200203)2002:4%3C834::AID-EJIC834%3E3.0.CO;2-Q.
255. Tao, G.-H.; Twamley, B.; Shreeve, J.M. Energetic nitrogen-rich Cu(II) and Cd(II) 5,5′-azobis(tetrazolate) complexes. *Inorg. Chem.* **2009**, *48*, 9918–9923. https://doi.org/10.1021/ic901492r.
256. Laus, G.; Kahlenberg, V.; Wurst, K.; Schottenberger, H.; Fischer, N.; Stierstorfer, J.; Klapötke, T.M. Synthesis and crystal structures of new 5,5′-azotetrazolates. *Crystals* **2012**, *2*, 127–136. https://doi.org/10.3390/cryst2010127.
257. Fischer, N.; Hüll, K.; Klapötke, T.M.; Stierstorfer, J.; Laus, G.; Hummel, M.; Froschauer, C.; Wurst, K.; Schottenberger, H. 5,5′-Azoxytetrazolates—A new nitrogen-rich dianion and its comparison to 5,5′-azotetrazolate. *Dalton Trans.* **2012**, *41*, 11201–11211. https://doi.org/10.1039/C2DT31217D.
258. Wu, L.; He, P.; Li, Z.; Wang, Q.; Yang, J.; Sinditskii, V.P.; Zhang, J.-G. Synthesis of 3,5-ditetrazolyl-1,2,4-triazole-based complexes: A strategy for developing C-N-linked triheterocyclic energetic compounds. *New J. Chem.* **2019**, *43*, 4975–4979. https://doi.org/10.1039/C8NJ06556J.

259. Klapötke, T.M.; Piercey, D.G.; Rohrbacher, F.; Stierstorfer, J. Synthesis and characterization of energetic salts of the ($C_4N_{12}^{2-}$) dianion. *Z. Anorg. Allg. Chem.* **2012**, *638*, 2235–2242. https://doi.org/10.1002/zaac.201200363.
260. Zhang, M.; Xu, J.-G.; Zhang, N.-N.; Lu, J.; Xin, X.-H.; Zheng, F.-K.; Guo, G.-C. A highly stable and tightly packed 3D energetic coordination polymer assembled from nitrogen-rich tetrazole derivatives. *New J. Chem.* **2018**, *42*, 13927–13932. https://doi.org/10.1039/C8NJ02659A.
261. Yang, Q.; Song, X.; Zhang, W.; Hou, L.; Gong, Q.; Xie, G.; Wei, Q.; Chen, S.; Gao, S. Three new energetic complexes with *N*,*N*-bis(1*H*-tetrazole-5-yl)-amine as high energy density materials: Syntheses, structures, characterization and effects on the thermal decomposition of RDX. *Dalton Trans.* **2017**, *46*, 2626–2634. https://doi.org/10.1039/C6DT04439E.
262. Xu, Y.; Liu, W.; Li, D.; Chen, H.; Lu, M. In situ synthesized 3D metal–organic frameworks (MOFs) constructed from transition metal cations and tetrazole derivatives: A family of insensitive energetic materials. *Dalton Trans.* **2017**, *46*, 11046–11052. https://doi.org/10.1039/C7DT02582C.
263. Zhang, S.; Liu, X.; Yang, Q.; Su, Z.; Gao, W.; Wei, Q.; Xie, G.; Chen, S.; Gao, S. A new strategy for storage and transportation of sensitive high-energy materials: Guest-dependent energy and sensitivity of 3D metal-organic-framework-based energetic compounds. *Chem. Eur. J.* **2014**, *20*, 7906–7910. https://doi.org/10.1002/chem.201402783.
264. Li, F.; Bi, Y.; Zhao, W.; Zhang, T.; Zhou, Z.; Yang, L. Nitrogen-rich salts based on the energetic [monoaquabis(*N*,*N*-bis(1*H*-tetrazol-5-yl)amine)-zinc(II)] anion: A promising design in the development of new energetic materials. *Inorg. Chem.* **2015**, *54*, 2050–2057. https://doi.org/10.1021/ic503021c.
265. Szimhardt, N.; Bölter, M.F.; Born, M.; Klapötke, T.M.; Stierstorfer, J. Metal salts and complexes of 1,1′-dinitramino-5,5′-bitetrazole. *Dalton Trans.* **2017**, *46*, 5033–5040. https://doi.org/10.1039/C7DT00536A.
266. Fischer, D.; Klapötke, T.M.; Stierstorfer, J. Potassium 1,1′-dinitramino-5,5′-bistetrazolate: A primary explosive with fast detonation and high initiation power. *Angew. Chem. Int. Ed.* **2014**, *53*, 8172–8175. https://doi.org/10.1002/anie.201404790.
267. Fischer, D.; Klapötke, T.M.; Stierstorfer, J.; Szimhardt, N. 1,1′-Nitramino-5,5′-bitetrazoles. *Chem. Eur. J.* **2016**, *22*, 4966–4970. https://doi.org/10.1002/chem.201600177.
268. Klapötke, T.M.; Stiasny, B.; Stierstorfer, J. Synthesis and investigation of 1,3-bis(5-nitraminotetrazol-1-yl)propan-2-ol and its salts. *Z. Anorg. Allg. Chem.* **2017**, *643*, 228–234. https://doi.org/10.1002/zaac.201600389.
269. He, P.; Liu, J.; Wu, J.; Mei, H.; Zhang, J. Dimethoxycarbonyl groups surrounding a symmetric diaminobistetrazole ring: Exploring new green energetic materials. *Chem. Asian J.* **2019**, *14*, 3845–3849. https://doi.org/10.1002/asia.201901271.
270. Klapötke, T.M.; Minar, N.K.; Stierstorfer, J. Investigations of bis(methyltetrazolyl)triazenes as nitrogen-rich ingredients in solid rocket propellants—Synthesis, characterization and properties. *Polyhedron* **2009**, *28*, 13–26. https://doi.org/10.1016/j.poly.2008.09.015.
271. Klapötke, T.M.; Sabaté, C.M.; Rasp, M. Alkali and transition metal (Ag, Cu) salts of bridged 5-nitrotetrazole derivatives for energetic applications. *Dalton Trans.* **2009**, 1825–1834. https://doi.org/10.1039/B818531J.
272. Kumar, D.; Imler, G.H.; Parrish, D.A.; Shreeve, J.M. Aminoacetonitrile as precursor for nitrogen rich stable and insensitive asymmetric *N*-methylene-C linked tetrazole-based energetic compounds. *J. Mater. Chem. A* **2017**, *5*, 16767–16775. https://doi.org/10.1039/C7TA05394K.
273. Joo, Y.-H.; Shreeve, J.M. Nitroimino-tetrazolates and oxy-nitroimino-tetrazolates. *J. Am. Chem. Soc.* **2010**, *132*, 15081–15090. https://doi.org/10.1021/ja107729c.
274. Nesveda, J. Bismuth-Based Energetic Materials. Patent Application No. WO2015/067228A1,14 April 2015.
275. Klapötke, T.M.; Kofen, M.; Schmidt, L.; Stierstorfer, J.; Wurzenberger, M.H.H. Selective synthesis and characterization of the highly energetic materials 1-hydroxy-5*H*-tetrazole (CHN_4O), its anion 1-oxido-5*H*-tetrazolate (CN_4O^-) and bis(1-hydroxytetrazol-5-yl)triazene. *Chem. Asian J.* **2021**, *16*, 3001–3012. https://doi.org/10.1002/asia.202100714.
276. Klapötke, T.M.; Piercey, D.G.; Stierstorfer, J. The taming of CN_7^-: The azidotetrazolate 2-oxide anion. *Chem. Eur. J.* **2011**, *17*, 13068–13077. https://doi.org/10.1002/chem.201102064.
277. Boneberg, F.; Kirchner, A.; Klapötke, T.M.; Piercey, D.G.; Poller, M.J.; Stierstorfer, J. A study of cyanotetrazole oxides and derivatives thereof. *Chem. Asian J.* **2013**, *8*, 148–159. https://doi.org/10.1002/asia.201200903.
278. Klapötke, T.M.; Kurz, M.Q.; Scharf, R.; Schmid, P.C.; Stierstorfer, J.; Sućeska, M. 5-(1*H*-Tetrazolyl)-2-hydroxy-tetrazole: A selective 2*N*-monoxidation of bis(1*H*-tetrazole). *ChemPlusChem* **2015**, *80*, 97–106. https://doi.org/10.1002/cplu.201402124.
279. Härtel, M.A.C.; Klapötke, T.M.; Piercey, D.G.; Stierstorfer, J. Synthesis and characterization of alkaline and alkaline earth salts of the nitrotetrazolate-2*N*-oxide anion. *Z. Anorg. Allg. Chem.* **2012**, *638*, 2008–2014. https://doi.org/10.1002/zaac.201200049.
280. Fischer, D.; Klapötke, T.M.; Stierstorfer, J. 5-Nitriminotetrazole 1-oxide: An exciting oxygen- and nitrogen-rich heterocycle. *Eur. J. Inorg. Chem.* **2015**, *2015*, 4628–4632. https://doi.org/10.1002/ejic.201500944.

281. Fischer, N.; Klapötke, T.M.; Marchner, S.; Rusan, M.; Scheutzow, S.; Stierstorfer, J. A Selection of alkali and alkaline earth metal salts of 5,5′-bis(1-hydroxytetrazole) in pyrotechnic compositions. *Propell. Explos. Pyrot.* **2013**, *38*, 448–459. https://doi.org/10.1002/prep.201200177.
282. Zhang, Q.; Chen, D.; Jing, D.; Fan, G.; He, L.; Li, H.; Wang, W.; Nie, F. Access to green primary explosives via constructing coordination polymers based on bis-tetrazole oxide and non-lead metals. *Green Chem.* **2019**, *21*, 1947–1955. https://doi.org/10.1039/C8GC03973A.
283. Fischer, D.; Klapötke, T.M.; Piercey, D.G.; Stierstorfer, J. Synthesis of 5-aminotetrazole-1*N*-oxide and its azo derivative: A key step in the development of new energetic materials. *Chem. Eur. J.* **2013**, *19*, 4602–4613. https://doi.org/10.1002/chem.201203493.
284. Zhang, J.; Jin, B.; Li, X.; Hao, W.; Huang, T.; Lei, B.; Guo, Z.; Shen, J.; Peng, R. Study of H_2AzTO-based energetic metal-organic frameworks for catalyzing the thermal decomposition of ammonium perchlorate. *Chem. Eng. J.* **2021**, *404*, 126287. https://doi.org/10.1016/j.cej.2020.126287.
285. He, P.; Zhang, J.-G.; Wu, L.; Wu, J.-T.; Zhang, T.-L. Magnesium azotetrazole-1,1′-dioxide: Synthesis and promising properties of green insensitive energetic materials. *Z. Anorg. Allg. Chem.* **2017**, *643*, 432–436. https://doi.org/10.1002/zaac.201600471.
286. Fu, W.; Zhao, B.; Zhang, M.; Li, C.; Gao, H.; Zhang, J.; Zhou, Z. 3,4-Dinitro-1-(1*H*-tetrazol-5-yl)-1*H*-pyrazol-5-amine (HANTP) and its salts: Primary and secondary explosives. *J. Mater. Chem. A* **2017**, *5*, 5044–5054. https://doi.org/10.1039/C6TA08376E.
287. Fischer, N.; Hüll, K.; Klapötke, T.M.; Stierstorfer, J. Synthesis and characterization of the new heterocycle 5-(4-amino-1,2,4-triazol-3-on-5′-yl)-1*H*-tetrazole and some ionic nitrogen-rich derivatives. *J. Heterocycl. Chem.* **2014**, *51*, 85–95. https://doi.org/10.1002/jhet.1877.
288. Xia, H.; Zhang, W.; Jin, Y.; Song, S.; Wang, K.; Zhang, Q. Synthesis of thermally stable and insensitive energetic materials by incorporating the tetrazole functionality into a fused-ring 3,6-dinitropyrazolo-[4,3-*c*]pyrazole framework. *ACS Appl. Mater. Interfaces* **2019**, *11*, 45914–45921. https://doi.org/10.1021/acsami.9b17384.
289. Joas, M.; Kießling, S.; Klapötke, T.M.; Schmid, P.C.; Stierstorfer, J. Energetic complexes of 5-(4-amino-1,2,4-triazol-3-on-5-yl)tetrazole and ionic derivatives of its 2*N*-oxide. *Z. Anorg. Allg. Chem.* **2014**, *640*, 2759–2765. https://doi.org/10.1002/zaac.201400417.
290. Yu, Q.; Imler, G.H.; Parrish, D.A.; Shreeve, J.M. Challenging the limits of nitro groups associated with a tetrazole ring. *Org. Lett.* **2019**, *21*, 4684–4688. https://doi.org/10.1021/acs.orglett.9b01565.
291. Kettner, M.A.; Klapötke, T.M. New energetic polynitrotetrazoles. *Chem. Eur. J.* **2015**, *21*, 3755–3765. https://doi.org/10.1002/chem.201405659.
292. Haiges, R.; Christe, K.O. Energetic high-nitrogen compounds: 5-(trinitromethyl)-2*H*-tetrazole and -tetrazolates, preparation, characterization, and conversion into 5-(dinitromethyl)tetrazoles. *Inorg. Chem.* **2013**, *52*, 7249–7260. https://doi.org/10.1021/ic400919n.
293. Klapötke, T.M.; Steemann, F.X. Dinitromethyltetrazole and its salts—A comprehensive study. *Propell. Explos. Pyrot.* **2010**, *35*, 114–129. https://doi.org/10.1002/prep.200900049.
294. Kumar, D.; Imler, G.H.; Parrish, D.A.; Shreeve, J.M. Resolving synthetic challenges faced in the syntheses of asymmetric *N*,*N*′-ethylene-bridged energetic compounds. *New J. Chem.* **2017**, *41*, 4040–4047. https://doi.org/10.1039/C7NJ00327G.
295. Izsák, D.; Klapötke, T.M.; Reuter, S. Salts of 5-(5-azido-1*H*-1,2,4-triazol-3-yl)tetrazol-1-ol: From highly sensitive primary explosives to insensitive nitrogen-rich salts. *Eur. J. Inorg. Chem.* **2013**, *2013*, 5641–5651. https://doi.org/10.1002/ejic.201300900.
296. Huber, S.; Izsák, D.; Karaghiosoff, K.; Klapötke, T.M.; Reuter, S. Energetic salts of 5-(5-azido-1*H*-1,2,4-triazol-3-yl)tetrazole. *Propell. Explos. Pyrot.* **2014**, *39*, 793–801. https://doi.org/10.1002/prep.201300203.
297. Zhang, Y.; Zhang, S.; Sun, L.; Yang, Q.; Han, J.; Wei, Q.; Xie, G.; Chen, S.; Gao, S. A solvent-free dense energetic metal–organic framework (EMOF): To improve stability and energetic performance via in situ microcalorimetry. *Chem. Commun.* **2017**, *53*, 3034–3037. https://doi.org/10.1039/C7CC00545H.
298. Gao, W.; Liu, X.; Su, Z.; Zhang, S.; Yang, Q.; Wei, Q.; Chen, S.; Xie, G.; Yang, X.; Gao, S. High-energy-density materials with remarkable thermostability and insensitivity: Syntheses, structures and physicochemical properties of Pb(II) compounds with 3-(tetrazol-5-yl) triazole. *J. Mater. Chem. A* **2014**, *2*, 11958–11965. https://doi.org/10.1039/C4TA01746C.
299. Liu, X.; Gao, W.; Sun, P.; Su, Z.; Chen, S.; Wei, Q.; Xie, G.; Gao, S. Environmentally friendly high-energy MOFs: Crystal structures, thermostability, insensitivity and remarkable detonation performances. *Green Chem.* **2015**, *17*, 831–836. https://doi.org/10.1039/C4GC02184C.
300. Kumar Chinnam, A.; Shlomovich, A.; Shamis, O.; Petrutik, N.; Kumar, D.; Wang, K.; Komarala, E.P.; Tov, D.S.; Sućeska, M.; Yan, Q.L.; et al. Combustion of energetic iodine-rich coordination polymer—Engineering of new biocidal materials. *Chem. Eng. J.* **2018**, *350*, 1084–1091. https://doi.org/10.1016/j.cej.2018.06.056.
301. Zhang, G.; Liu, Y.; Dong, Z.; Liu, Y.; Li, H.; Ye, Z. New Energetic Metal-Organic Framework (E-MOF) based on a sodium(I)-containing energetic metal salt incorporating guanidinium ions. *Z. Anorg. Allg. Chem.* **2022**, *648*, e202100270. https://doi.org/10.1002/zaac.202100270.

302. Mei, H.; Xu, Y.; Lei, G.; Cao, W.; Li, Z.; Zhang, J. Synthesis, structure and properties of a high-energy metal–organic framework fuel $[Cu(MTZ)_2(CTB)_2]_n$. *New J. Chem.* **2022**, *46*, 1687–1692. https://doi.org/10.1039/D1NJ05710C.
303. Klapötke, T.M.; Radies, H.; Stierstorfer, J.; Tarantik, K.R.; Chen, G.; Nagori, A. Coloring properties of various high-nitrogen compounds in pyrotechnic compositions. *Propell. Explos. Pyrot.* **2010**, *35*, 213–219. https://doi.org/10.1002/prep.201010018.
304. Steinhauser, G.; Klapötke, T.M. "Green" pyrotechnics: A chemists' challenge. *Angew. Chem. Int. Ed.* **2008**, *47*, 3330–3347. https://doi.org/10.1002/anie.200704510.
305. Sabatini, J.J.; Moretti, J.D. High-nitrogen-based pyrotechnics: Perchlorate-free red- and green-light illuminants based on 5-aminotetrazole. *Chem. Eur. J.* **2013**, *19*, 12839–12845. https://doi.org/10.1002/chem.201300779.
306. He, P.; Zhang, J.-G.; Yin, X.; Wu, J.-T.; Wu, L.; Zhou, Z.-N.; Zhang, T.-L. Energetic salts based on tetrazole *N*-oxide. *Chem. Eur. J.* **2016**, *22*, 7670–7685. https://doi.org/10.1002/chem.201600257.
307. Wang, T.; Gao, H.; Shreeve, J.M. Functionalized tetrazole energetics: A route to enhanced performance. *Z. Anorg. Allg. Chem.* **2021**, *647*, 157–191. https://doi.org/10.1002/zaac.202000361.
308. Larin, A.A.; Fershtat, L.L. High-energy hydroxytetrazoles: Design, synthesis and performance. *Energetic Mater. Front.* **2021**, *2*, 3–13. https://doi.org/10.1016/j.enmf.2021.01.001.
309. Singh, R.P.; Verma, R.D.; Meshri, D.T.; Shreeve, J.M. Energetic nitrogen-rich salts and ionic liquids. *Angew. Chem. Int. Ed.* **2006**, *45*, 3584–3601. https://doi.org/10.1002/anie.200504236.
310. Wu, J.T.; Zhang, J.-G.; Zhang, T.L.; Yang, L. Energetic nitrogen-rich salts. *Cent. Eur. J. Energ. Mater.* **2015**, *12*, 417–437.
311. Gao, H.; Shreeve, J.M. Azole-based energetic salts. *Chem. Rev.* **2011**, *111*, 7377–7436. https://doi.org/10.1021/cr200039c.
312. Sajjadi, M.; Nasrollahzadeh, M.; Ghafuri, H.; Pombeiro, A.J.L.; Hazra, S. Copper tetrazole compounds: Structures, properties and applications. *Coord. Chem. Rev.* **2024**, *504*, 215604. https://doi.org/10.1016/j.ccr.2023.215604.
313. Andrate, T. The Gunpowder Age: China, Military Innovation, and the Rise of the West in World History; Princeton University Press: Princeton, NJ, USA, 2016.
314. Padmanabhan, T.; Padmanabhan, V. The Dawn of Science. Glimpses from History for the Curious Mind; Springer: New York, NY, USA, 2019.
315. Romocki, S.J. *Geschichte der Explosivstoffe*; Oppenheim: Berlin, Germany, 1895.
316. Ledgard, J.B. The Preparatory Manual of Black Powder and Pyrotechnics; Lulu Press: Morrisville, NY, USA, 2006.
317. Foreman, B.; Ittenbach, J.; Swartzendruber, C. Speculation on the explosive decomposition of "Yellow Powder". *J. Pyrotech.* **2001**, *14*, 65–68.
318. Cook, M.A.; Clay, R.B.; Udy, L.L. Blasting Slurry Compositions Containing Calicum Nitrate and Method of Preparation. U.S. Patent 3713917A, 30 January 1973.
319. Wilson, J.S.; Clark, W.F.; Slykhouse, T.E. Method of Making Calcium Nitrate Explosive Composition. U.S. Patent 3816191A, 11 Juny 1974.
320. Hess, G. Explosive question: Indiana nitrogen fertilizer plant on hold over link to bombs in Afghanistan. *Chem. Eng. News* **2013**, *91*, 24–25. https://doi.org/10.1021/cen-09108-govpol1.
321. Cullum, H.; Lowe, A.; Marshall, M.; Hubbard, P. Physical and chemical evidence remaining after the explosion of large improvised bombs. Part 2: Firings of calcium ammonium nitrate/sugar mixtures. *J. For. Sci.* **2000**, *45*, 333–348. https://doi.org/10.1520/JFS14686J.
322. Russel, M.S. *The Chemistry of Fireworks*, 2nd ed.; RSC Publishing: Cambridge, UK, 2009.
323. Conkling, J.A.; Mocella, C.J. *Chemistry of Pyrotechnics: Basic Principles and Theory*, 2nd ed.; CRC Publishing: Boca Raton, FL, USA, 2010.
324. Ajith, V.; Arumugaprabu, V.; Ramalakshmi, R.; Indumathi, N. A study on thermal characterisation of effective pyrotechnic flash compositions. *AIP Conf. Proc.* **2022**, *2520*, e030008. https://doi.org/10.1063/5.0103830.
325. Selvakumar, N.; Azhagurajan, A.; Sheikmohamed, P.; Suresh, A. Ballistic behaviour of gun powder and flash powder for firework chemicals as a function of particle sizes. *Measurement* **2013**, *46*, 3202–3210. https://doi.org/10.1016/j.measurement.2013.06.006.
326. Sivapirakasam, S.P.; Surianarayanan, M.; Chandrasekaran, F. Thermal characterization of pyrotechnic flash compositions. *Sci. Technol. Energetic Mater.* **2010**, *71*, 11–16.
327. Sivapirakasam, S.P.; Surianarayanan, M. Experimental investigation of mechanical sensitivity and noise level for different pyrotechnic flash compositions. *Sci. Technol. Energetic Mater.* **2009**, *70*, 140–144.
328. Koch, E.C. Special materials in pyrotechnics. Part 2. Application of cæsium and rubidium compounds in pyrotechnics. *J. Pyrotech.* **2002**, *15*, 9–23.
329. Jennings-White, C. Glitter chemistry. *J. Pyrotech.* **1998**, *8*, 53–70.
330. Koch, E.C. Evaluation of lithium compounds as color agents for pyrotechnic flames. *J. Pyrotech.* **2001**, *13*, 1–8.

331. Koch, E.-C. Special materials in pyrotechnics: III. Application of lithium and its compounds in energetic systems. *Propell. Explos. Pyrot.* **2004**, *29*, 67–80. https://doi.org/10.1002/prep.200400032.
332. Pulpea, B.G.; Pulpea, D.; Trană, E.; Rotariu, T.; Ginghină, R.E.; Toader, G.; Dirloman, F.M. Design and evaluation of screening smoke compositions based on red phosphorus in open field conditions. *Appl. Sci.* **2022**, *12*, e12893. https://doi.org/10.3390/app122412893.
333. Warpinski, N.; Engler, B.; Uhl, J.; Grubelich, M.; Kravitz, S.; Nogan, J.; Rivas, R.; Cooper, P.; Dulleck, G.; Ingram, B.; et al. *Autonomous Microexplosives Subsurface Tracing System Final Report*; Sandia National Laboratories: Albuquerque, New Mexico, 2023.
334. Traynor, K. Pharmacy products could be on bomb makers' shopping lists. *Am. J. Health Syst. Pharm.* **2001**, *58*, 2222. https://doi.org/10.1093/ajhp/58.23.2222a.
335. Mellor, J.M.; Mittoo, S.; Parkes, R.; Millar, R.W. Improved nitrations using metal nitrate-sulfuric acid systems. *Tetrahedron* **2000**, *56*, 8019–8024. https://doi.org/10.1016/S0040-4020(00)00720-1.
336. Straessler, N.A. Methods for Nitrating Compounds. U.S. Patent 7737308B1, 15 Juny 2010.
337. Sheikh, I. Utilizing Nitrate Salts in Order to Produce Explosives. U.S. Patent 2016/0039722A1, 11 February 2016.
338. Guy, A. *Explosive Nitrated Carbon Compounds*. Available online: https://edu.rsc.org/exhibition-chemistry/explosive-nitrated-carbon-compounds/2020052.article (accessed on 2 April 2023).
339. Iqubal, R.; Aslam, M.M. Nitration of aromatic compounds with potassium nitrate in polyphosphotic acid. *J. Chem. Soc. Pak.* **1986**, *8*, 443–447.
340. Supniewski, J. *Preparatyka Nieorganiczna*; PWN: Warszawa, Poland, 1957.
341. Sękowski, S. *Pierwiastki w Moim Laboratorium*; WSiP: Warszawa, Poland, 1989.
342. Wojewódka, A.; Bełzowski, J. Hydrazynowe kompleksy metali przejściowych jako perspektywiczne materiały wybuchowe. *Chemik* **2011**, *65*, 20–27.
343. Wojewódka, A.; Bełzowski, J.; Wilk, Z.; Staś, J. Energetic characteristics of transition metal complexes. *J. Hazard. Mater.* **2009**, *171*, 1175–1177. https://doi.org/10.1016/j.jhazmat.2009.06.104.
344. Chhabra, J.S.; Talawar, M.B.; Makashir, P.S.; Asthana, S.N.; Singh, H. Synthesis, characterization and thermal studies of (Ni/Co) metal salts of hydrazine: Potential initiatory compounds. *J. Hazard. Mater.* **2003**, *99*, 225–239. https://doi.org/10.1016/S0304-3894(02)00247-9.
345. Zhang, G.-y.; Liu, J.-c.; Song, N.-m.; Liu, Y.; Yang, L. Research on the thermal performance and storage life of series of high-energy hydrazine nitrate complexes. *J. Therm. Anal. Calorim.* **2017**, *129*, 1887–1897. https://doi.org/10.1007/s10973-017-6323-3.
346. Shunguan, Z.; Youchen, W.; Wenyi, Z.; Jingyan, M. Evaluation of a new primary explosive: Nickel hydrazine nitrate (NHN) complex. *Propell. Explos. Pyrot.* **1997**, *22*, 317–320. https://doi.org/10.1002/prep.19970220604.
347. Talawar, M.B.; Agrawal, A.P.; Anniyappan, M.; Wani, D.S.; Bansode, M.K.; Gore, G.M. Primary explosives: Electrostatic discharge initiation, additive effect and its relation to thermal and explosive characteristics. *J. Hazard. Mater.* **2006**, *137*, 1074–1078. https://doi.org/10.1016/j.jhazmat.2006.03.043.
348. Talawar, M.B.; Agrawal, A.; Chhabra, J.S.; Ghatak, C.; Asthana, S.N.; Rao, K.U.M. Studies on nickel hydrazinium nitrate (NHN) and bis-(5-nitro-2*H* tetrazolato-N^2)tetraamino cobalt(III) perchlorate (BNCP): Potential lead-free advanced primary explosives. *J. Sci. Ind. Res.* **2004**, *63*, 677–681.
349. Cartwright, M. Investigation of preparation, solubility and stability properties of nickel hydrazine nitrate (NiHN). *Propell. Explos. Pyrot.* **2018**, *43*, 1270–1276. https://doi.org/10.1002/prep.201700255.
350. Bushuyev, O.S.; Brown, P.; Maiti, A.; Gee, R.H.; Peterson, G.R.; Weeks, B.L.; Hope-Weeks, L.J. Ionic polymers as a new structural motif for high-energy-density materials. *J. Am. Chem. Soc.* **2012**, *134*, 1422–1425. https://doi.org/10.1021/ja209640k.
351. Joyner, T.B. Explosive sensitivity of cobalt(III) ammine complexes. *Can. J. Chem.* **1969**, *47*, 2719–2730. https://doi.org/10.1139/v69-452.
352. Tomlinson, W.R.; Ottoson, K.G.; Audrieth, L.F. Explosive properties of metal ammines. *J. Am. Chem. Soc.* **1949**, *71*, 375–376. https://doi.org/10.1021/ja01169a519.
353. Künzel, M.; Vodochodský, O.; Matyáš, R.; Jalový, Z.; Pachman, J.; Maixner, J. Tetraamminecopper(II) nitrate and its effects on ammonium nitrate(V). *Cent. Eur. J. Energ. Mater.* **2017**, *14*, 169–183. https://doi.org/10.22211/cejem/67469.
354. Sun, Q.; Li, X.; Lin, Q.; Lu, M. Dancing with 5-substituted monotetrazoles, oxygen-rich ions, and silver: Towards primary explosives with positive oxygen balance and excellent energetic performance. *J. Mater. Chem. A* **2019**, *7*, 4611–4618. https://doi.org/10.1039/C8TA12506F.
355. Karaghiosoff, K.; Klapötke, T.M.; Miró Sabaté, C. Energetic silver salts with 5-aminotetrazole ligands. *Chem. Eur. J.* **2009**, *15*, 1164–1176. https://doi.org/10.1002/chem.200801666.
356. Freis, M.; Klapötke, T.M.; Stierstorfer, J.; Szimhardt, N. Di(1*H*-tetrazol-5-yl)methane as neutral ligand in energetic transition metal complexes. *Inorg. Chem.* **2017**, *56*, 7936–7947. https://doi.org/10.1021/acs.inorgchem.7b00432.

357. Wurzenberger, M.H.H.; Gruhne, M.S.; Lommel, M.; Szimhardt, N.; Klapötke, T.M.; Stierstorfer, J. Comparison of 1-ethyl-5*H*-tetrazole and 1-azidoethyl-5*H*-tetrazole as ligands in energetic transition metal complexes. *Chem. Asian J.* **2019**, *14*, 2018–2028. https://doi.org/10.1002/asia.201900269.
358. Bauer, L.; Endraß, S.M.J.; Klapötke, T.M.; Stierstorfer, J.; Zeitlmeir, N. *N*-Azidoethyl azoles through *N*-alkylation under highly harmonized reaction conditions: Synthesis, characterization, and complexation as energetic coordination compounds. *J. Heterocycl. Chem.* **2024**, *61*, 839–851. https://doi.org/10.1002/jhet.4803.
359. Evers, J.; Gospodinov, I.; Joas, M.; Klapötke, T.M.; Stierstorfer, J. Cocrystallization of photosensitive energetic copper(II) perchlorate complexes with the nitrogen-rich ligand 1,2-di(1*H*-tetrazol-5-yl)ethane. *Inorg. Chem.* **2014**, *53*, 11749–11756. https://doi.org/10.1021/ic5020175.
360. Szimhardt, N.; Gruhne, M.S.; Lommel, M.; Hess, A.; Wurzenberger, M.H.H.; Klapötke, T.M.; Stierstorfer, J. 2,2-Bis(5-tetrazolyl)propane as ligand in energetic 3*d* transition metal complexes. *Z. Anorg. Allg. Chem.* **2019**, *645*, 354–361. https://doi.org/10.1002/zaac.201800288.
361. Szimhardt, N.; Wurzenberger, M.H.H.; Zeisel, L.; Gruhne, M.S.; Lommel, M.; Stierstorfer, J. Maximization of the energy capability level in transition metal complexes through application of 1-amino- and 2-amino-5*H*-tetrazole ligands. *J. Mater. Chem. A* **2018**, *6*, 16257–16272. https://doi.org/10.1039/C8TA06326E.
362. Wojewódka, A.; Bełzowski, J. Synthesis and safety properties of new explosive coordination compounds. *Cent. Eur. J. Energ. Mater.* **2017**, *14*, 351–360. https://doi.org/10.22211/cejem/71200.
363. Klapötke, T.M.; Schmid, P.C.; Stierstorfer, J.; Szimhardt, N. Synthesis and characterization of tetrahedral zinc(II) complexes with 3,6,7-triamino-7*H*-[1,2,4]triazolo [4,3-*b*][1,2,4]triazole as nitrogen-rich ligand. *Z. Anorg. Allg. Chem.* **2016**, *642*, 383–389. https://doi.org/10.1002/zaac.201600006.
364. Jin, X.; Zhang, J.-G.; Xu, C.-X.; Yin, X.; He, P.; Qin, Q. Eco-friendly energetic complexes based on transition metal nitrates and 3,4-diamino-1,2,4-triazole (DATr). *J. Coord. Chem.* **2014**, *67*, 3202–3215. https://doi.org/10.1080/00958972.2014.960862.
365. Li, S.; Wang, Y.; Qi, C.; Zhao, X.; Zhang, J.; Zhang, S.; Pang, S. 3D energetic metal–organic frameworks: Synthesis and properties of high energy materials. *Angew. Chem. Int. Ed.* **2013**, *52*, 14031–14035. https://doi.org/10.1002/anie.201307118.
366. Zhang, J.; Zhu, Z.; Zhou, M.; Zhang, J.; Hooper, J.P.; Shreeve, J.M. Superior high-energy-density biocidal agent achieved with a 3D metal–organic framework. *ACS Appl. Mater. Interfaces* **2020**, *12*, 40541–40547. https://doi.org/10.1021/acsami.0c12251.
367. Liu, Y.; Jin, S.; Yang, H.; Li, S.; Xie, W.; Zhao, Y.; Zhang, W.; Chen, Y.; Fan, X. Application of 3D energetic metal-organic frameworks containing Cu as the combustion catalyst to composite solid propellant. *Combust. Flame* **2021**, *225*, 57–64. https://doi.org/10.1016/j.combustflame.2020.10.035.
368. Fischer, N.; Joas, M.; Klapötke, T.M.; Stierstorfer, J. Transition metal complexes of 3-amino-1-nitroguanidine as laser ignitible primary explosives: Structures and properties. *Inorg. Chem.* **2013**, *52*, 13791–13802. https://doi.org/10.1021/ic402038x.
369. Zhang, L.; Wang, T.-W.; Dong, W.-S.; Zhang, C.; Lu, Z.-J.; Zhou, Z.-N.; Zhang, J.-G. Tailoring transition metals and biuret into laser-ignitable energetic coordination polymers with improved oxygen balance and multidentate coordination structures. *Cryst. Growth Des.* **2023**, *23*, 1959–1971. https://doi.org/10.1021/acs.cgd.2c01505.
370. Myers, T.W.; Brown, K.E.; Chavez, D.E.; Scharff, R.J.; Veauthier, J.M. Correlating the structural, electronic, and explosive sensitivity properties of Cu^{II} tetrazine complexes. *Eur. J. Inorg. Chem.* **2016**, *2016*, 3178–3183. https://doi.org/10.1002/ejic.201600344.
371. Yang, G.; Li, X.; Wang, M.; Xia, Z.; Yang, Q.; Wei, Q.; Xie, G.; Chen, S.; Gao, S.; Lu, J.Y. Improved detonation performance via coordination substitution: Synthesis and characterization of two new green energetic coordination polymers. *ACS Appl. Mater. Interfaces* **2021**, *13*, 563–569. https://doi.org/10.1021/acsami.0c18271.
372. Yang, Q.; Yang, G.; Zhang, W.; Zhang, S.; Yang, Z.; Xie, G.; Wei, Q.; Chen, S.; Gao, S. Superior thermostability, good detonation properties, insensitivity, and the effect on the thermal decomposition of ammonium perchlorate for a new solvent-free 3D energetic PbII-MOF. *Chem. Eur. J.* **2017**, *23*, 9149–9155. https://doi.org/10.1002/chem.201701325.
373. Zhang, L.; Dong, W.-S.; Lu, Z.-J.; Wang, T.-W.; Zhang, C.; Zhou, Z.-N.; Zhang, J.-G. Synthesis and characterization of thermally stable energetic complexes with 3,5-diaminopyrazolone-4-oxime as a nitrogen-rich ligand. *CrystEngComm* **2022**, *24*, 5519–5526. https://doi.org/10.1039/D2CE00715K.
374. Zhang, X.; Wu, Y.; Wang, P.; Lin, Q.; Chen, S.; Jin, S.; Xu, Y.; Lu, M. An interesting 3D energetic metal–framework based Ag(I) ions and 3,4-diaminofurazan. *J. Energ. Mater.* **2022**, *42*, 349–361. https://doi.org/10.1080/07370652.2022.2073486.
375. Gong, L.; Chen, G.; Liu, Y.; Wang, T.; Zhang, J.; Yi, X.; He, P. Energetic metal–organic frameworks achieved from furazan and triazole ligands: Synthesis, crystal structure, thermal stability and energetic performance. *New J. Chem.* **2021**, *45*, 22299–22305. https://doi.org/10.1039/D1NJ04486A.

376. Chen, S.-l.; Shang, Y.; Jiang, J.; Huang, M.; Ren, J.-t.; Guo, T.; Yu, C.-x.; Zhang, W.-x.; Chen, X.-m. A new nitrate-based energetic molecular perovskite as a modern edition of black powder. *Energetic Mat. Front.* **2022**, *3*, 122–127. https://doi.org/10.1016/j.enmf.2022.07.003.
377. McLain, J.H. Pyrotechnics from the Viewpoint of Solid State Chemistry; Franklin Institute Press: Philadelphia, PA, USA, 1980.
378. Donner, J. A professional Guide Do Pyrotechnics: Understanding and Making Exploding Fireworks; Paladin Press: Boulder, CO, USA, 1997.
379. Ellern, H. *Military and Civilian Pyrotechnics*; Chemical Publishing Company: New York, NY, USA, 1968.
380. Amiel, J. Sur les perchlorates et les bromates cuivriques complexes formés avec quelques amines primaires. *C. R. Hebd. Seances Acad. Sci.* **1935**, *200*, 672–674.
381. Maissen, B.; Schwarzebach, G. Eine Substanz von äusserster Gefärlichkeit: Hydrazinnickelperchlorat. *Helv. Chim. Acta* **1951**, *34*, 252–253. https://doi.org/10.1002/hlca.19510340650.
382. Bushuyev, O.S.; Peterson, G.R.; Brown, P.; Maiti, A.; Gee, R.H.; Weeks, B.L.; Hope-Weeks, L.J. Metal–organic frameworks (MOFs) as safer, structurally reinforced energetics. *Chem. Eur. J.* **2013**, *19*, 1706–1711. https://doi.org/10.1002/chem.201203610.
383. Horrocks, A.J.; Detata, D.; Pitts, K.; Lewis, S.W. Chlorate-based homemade explosives: A review. *WIREs Forensic Sci.* **2024**, *6*, e1506. https://doi.org/10.1002/wfs2.1506.
384. Talawar, M.B.; Agrawal, A.P.; Asthana, S.N. Energetic co-ordination compounds: Synthesis, characterization and thermolysis studies on bis-(5-nitro-2*H*-tetrazolato-N^2)tetraammine cobalt(III) perchlorate (BNCP) and its new transition metal (Ni/Cu/Zn) perchlorate analogues. *J. Hazard. Mater.* **2005**, *120*, 25–35. https://doi.org/10.1016/j.jhazmat.2004.12.021.
385. Zhilin, A.Y.; Ilyushin, M.A.; Tselinskii, I.V.; Brykov, A.S. Synthesis of a high-energy-capacity compound, tetrammine-*cis*-bis(nitro-2*H*-tetrazolato-N^2)cobalt(III) perchlorate. *Russ. J. Appl. Chem.* **2001**, *74*, 99–102. https://doi.org/10.1023/A:1012756202841.
386. Kofen, M.; Klapötke, T.M.; Stierstorfer, J. Energetic coordination compounds of late 3*d* metals with 1*N*-(Nitromethyl)-5*H*-tetrazole, a ligand with astonishing properties from the rare class of *N*-nitromethyl azoles. *Chem. Eng. J.* **2023**, *452*, 139375. https://doi.org/10.1016/j.cej.2022.139375.
387. Kofen, M.; Braun, V.; Endraß, S.M.J.; Klapötke, T.M.; Stierstorfer, J. 1-(Nitratomethyl)-5*H*-tetrazole: A highly sensitive ligand with improved oxygen balance for laser-ignitable coordination compounds. *Inorg. Chem.* **2022**, *61*, 17212–17225. https://doi.org/10.1021/acs.inorgchem.2c02805.
388. Zeisel, L.; Szimhardt, N.; Wurzenberger, M.H.H.; Klapötke, T.M.; Stierstorfer, J. 2-Methyl-substituted monotetrazoles in copper(II) perchlorate complexes: Manipulating coordination chemistry and derived energetic properties. *New J. Chem.* **2019**, *43*, 609–616. https://doi.org/10.1039/C8NJ05375H.
389. Wurzenberger, M.H.H.; Gruhne, M.S.; Lommel, M.; Stierstorfer, J. 1-Amino-5-methyltetrazole in energetic 3*d* transition metal complexes—Ligand design for future primary explosives. *Propell. Explos. Pyrot.* **2021**, *46*, 207–213. https://doi.org/10.1002/prep.202000179.
390. Tang, H.; Zhou, Z.; Li, Z.; Chen, S.; Wang, L.; Zhang, T. Copper(II) complexes of 1-methyl-5-aminotetrazole with different energetic anions: Syntheses, crystal structures and properties. *J. Energ. Mater.* **2021**, *39*, 23–32. https://doi.org/10.1080/07370652.2020.1748763.
391. Wurzenberger, M.H.H.; Szimhardt, N.; Stierstorfer, J. Copper(II) chlorate complexes: The renaissance of a forgotten and misjudged energetic anion. *J. Am. Chem. Soc.* **2018**, *140*, 3206–3209. https://doi.org/10.1021/jacs.7b13230.
392. Cudziło, S.; Nita, M. Synthesis and explosive properties of copper(II) chlorate(VII) coordination polymer with 4-amino-1,2,4-triazole bridging ligand. *J. Hazard. Mater.* **2010**, *177*, 146–149. https://doi.org/10.1016/j.jhazmat.2009.12.008.
393. Cudziło, S.; Trzciński, W.; Paszula, J.; Nita, M. Detonation and decomposition characteristics of dichlorate(VII) μ-tris(4-amino-1,2,4-triazole)copper(II). *Cent. Eur. J. Energ. Mater.* **2014**, *11*, 539–552.
394. Wang, T.; Zhang, Q.; Deng, H.; Shang, L.; Chen, D.; Li, Y.; Zhu, S.; Li, H. Evolution of oxidizing inorganic metal salts: Ultrafast laser initiation materials based on energetic cationic coordination polymers. *ACS Appl. Mater. Interfaces* **2019**, *11*, 41523–41530. https://doi.org/10.1021/acsami.9b14353.
395. Zhilin, A.Y.; Ilyushin, M.A.; Tselinskii, I.V.; Kozlov, A.S.; Lisker, I.S. High-energy-capacity cobalt(III) tetrazolates. *Russ. J. Appl. Chem.* **2003**, *76*, 572–576.
396. Sinditskii, V.; Dutov, M.; Fogelzang, A.; Vernidub, T.; Sokol, V.; Porai-Koshits, M. Synthesis and spectroscopic studies on nickel(II), cobalt(II) and copper(II) complexes of tetrazolyl-l-acethydrazide. Crystal structure of $[Cu(TH\text{-}1)_2](ClO_4)_2$. *Inorg. Chim. Acta* **1991**, *189*, 259–266. https://doi.org/10.1016/S0020-1693(00)80198-X.
397. Sinditskii, V.; Vernidub, T.; Fogel'zang, A.; Zueva, N.A. Coordination compounds of 4-amino-1,2,4-triazole with metal chlorates, bromates, and nitrates. *Izv. Vyss. Uchebnykh Zaved.* **1991**, *34*, 15–19.
398. Graeber, E.J.; Morosin, B. Structures of pentaammine(5-cyanotetrazolato-N^2)cobalt(III) perchlorate (CP), $[Co(C_2N_5)(NH_3)_5](ClO_4)_2$, and (5-amidinotetrazolato-N^1,N^5)tetraamminecobalt(III) bromide (ATCB), $[Co(C_2H_3N_6)(NH_3)_4]Br_2$. *Acta. Crystallogr. C* **1983**, *39*, 567–570. https://doi.org/10.1107/S010827018300548X.

399. Smirnov, A.V.; Ilyushin, M.A.; Tselinskii, I.V. Synthesis of cobalt(III) ammine complexes as explosives for safe priming charges. *Russ. J. Appl. Chem.* **2004**, *77*, 794–796. https://doi.org/10.1023/B:RJAC.0000038815.59138.f3.
400. Lechner, J.T.; Riedelsheimer, C.; Endraß, S.M.J.; Gerold, N.M.; Heidrich, J.; Krumm, B.; Stierstorfer, J.; Klapötke, T.M. Synthesis of bridged tetrazoles with promising properties and potential applications by a one-step Finkelstein reaction. *Chem. Eur. J.* **2024**, *30*, e202303021. https://doi.org/10.1002/chem.202303021.
401. Talawar, M.B.; Agrawal, A.P.; Chhabra, J.S.; Asthana, S.N. Studies on lead-free initiators: Synthesis, characterization and performance evaluation of transition metal complexes of carbohydrazide. *J. Hazard. Mater.* **2004**, *113*, 57–65. https://doi.org/10.1016/j.jhazmat.2004.07.001.
402. Joas, M.; Klapötke, T.M. Laser Initiation of tris(carbohydrazide)metal(II) perchlorates and bis(carbohydrazide)diperchlorato-copper(II). *Propell. Explos. Pyrot.* **2015**, *40*, 246–252. https://doi.org/10.1002/prep.201400142.
403. Reichel, M.; Wurzenberger, M.H.H.; Lommel, M.; Kofen, M.; Krumm, B.; Stierstorfer, J.; Karaghiosoff, K. *N*-fluoromethylated (amino)tetrazoles: Manipulating thermal and energetic properties. *Z. Anorg. Allg. Chem.* **2021**, *647*, 341–349. https://doi.org/10.1002/zaac.202000341.
404. Ilyushin, M.A.; Aleksandrova, M.A.; Bachurina, I.V.; Smirnov, A.V.; Tselinskii, I.V. Synthesis and properties of an energetic complex pentaamminecobalt(III) perchlorate, with 4-amino-1,2,4-triazole as ligand. *Russ. J. Appl. Chem.* **2010**, *83*, 92–96. https://doi.org/10.1134/S1070427210010180.
405. Cui, Y.; Zhang, J.; Zhang, T.; Yang, L.; Zhang, J.; Hu, X. Synthesis, structural investigation, thermal decomposition mechanism and sensitivity properties of an energetic compound $[Cd(DAT)_6](ClO_4)_2$ (DAT = 1,5-diaminotetrazole). *J. Hazard. Mater.* **2008**, *160*, 45–50. https://doi.org/10.1016/j.jhazmat.2008.02.078.
406. Myers, T.W.; Bjorgaard, J.A.; Brown, K.E.; Chavez, D.E.; Hanson, S.K.; Scharff, R.J.; Tretiak, S.; Veauthier, J.M. Energetic chromophores: Low-energy laser initiation in explosive Fe(II) tetrazine complexes. *J. Am. Chem. Soc.* **2016**, *138*, 4685–4692. https://doi.org/10.1021/jacs.6b02155.
407. Myers, T.W.; Brown, K.E.; Chavez, D.E.; Scharff, R.J.; Veauthier, J.M. Laser initiation of Fe(II) complexes of 4-nitro-pyrazolyl substituted tetrazine ligands. *Inorg. Chem.* **2017**, *56*, 2297–2303. https://doi.org/10.1021/acs.inorgchem.6b02998.
408. Ugryumov, I.; Ilyushin, M.; Tselinskii, I.; Kozlov, A. Synthesis and properties of photosensitive complex perchlorates of *d* metals with 3(5)-hydrazino-4-amino-1,2,4-triazole as ligand. *Russ. J. Appl. Chem.* **2003**, *76*, 439–441. https://doi.org/10.1023/A:1025661019880.
409. Oam, D.R. A ten year retrospective of the bombing campaign in Indonesia by terrorists, 2002–2006. *Aust. J. Forensic Sci.* **2013**, *45*, 123–146. https://doi.org/10.1080/00450618.2012.733027.
410. Vodochodský, O.; Künzel, M.; Matyáš, R.; Kučera, J.; Pachman, J. Tetraamminecopper perchlorate (TACP): Explosive properties. *Propell. Explos. Pyrot.* **2021**, *46*, 280–285. https://doi.org/10.1002/prep.202000131.
411. Matyáš, R.; Jalový, Z.; Vodochodský, O.; Zmrhalová, Z.; Maixner, J.; Lapčák, L.; Baroš, P.; Hausner, J.; Kolba, P.; Čapková, Z. Analysis and detection of homemade explosive TACP. *Forensic Sci. Int.* **2024**, *364*, e112217. https://doi.org/10.1016/j.forsciint.2024.112217.
412. Waselowsky, K. 225 x Chemie—Ein Experimentierbuch. Ausgewählte Versuche aus der Anorganischem Chemie; Kosmos: Stuttgart, Germany, 1982.
413. Wurzenberger, M.H.H.; Szimhardt, N.; Stierstorfer, J. Nitrogen-rich copper(II) bromate complexes: An exotic class of primary explosives. *Inorg. Chem.* **2018**, *57*, 7940–7949. https://doi.org/10.1021/acs.inorgchem.8b01045.
414. Beck, M.W.; Brown, M.E. Burning of antimony/potassium permanganate pyrotechnic compositions in closed systems. *Combust. Flame* **1986**, *65*, 263–271. https://doi.org/10.1016/0010-2180(86)90040-4.
415. Beck, M.W.; Brown, M.E. Thermal analysis of antimony/potassium permanganate pyrotechnic compositions. *Thermochim. Acta* **1983**, *65*, 197–212. https://doi.org/10.1016/0040-6031(83)80022-7.
416. Kanga, S. *Potassium Permanganate Explosive Mixtures*. Patent Application No. KR20050006670A, 17 January 2005.
417. Polis, M.; Szydło, K.; Zakusylo, R.; Hawelek, L.; Stolarczyk, A.; Jarosz, T. Study of the combustion mechanism of $Zn/KMnO_4$ pyrotechnic composition. *Molecules* **2023**, *28*, e5741. https://doi.org/10.3390/molecules28155741.
418. Beck, M.W.; Brown, M.E. Modification of the burning rate of antimony/potassium permanganate pyrotechnic delay compositions. *Combust. Flame* **1986**, *66*, 67–75. https://doi.org/10.1016/0010-2180(86)90033-7.
419. Babar, Z.; Malik, A.Q. Thermal decomposition, ignition and kinetic evaluation of magnesium and aluminium fuelled pyrotechnic compositions. *Cent. Eur. J. Energ. Mater.* **2015**, *12*, 579–592.
420. Shakhashiri, B.Z. Chemical Demonstrations: A Handbook for Teachers of Chemistry. Vol. 1; University of Wisconsin Press: Madison, WI, USA, 1983.
421. Snowdon, W. Fighting forest fires with fire: Pyrotechnics and flaming Ping-Pong balls. *CBC News* 2020, 22 June 2020 (on-line edition).
422. Koch, K.R. Oxidation by Mn_2O_7: An impressive demonstration of the powerful oxidizing property of dimanganeseheptoxide. *J. Chem. Educ.* **1982**, *59*, 973. https://doi.org/10.1021/ed059p973.3.
423. Bircumshaw, L.L.; Tayler, F.M. The thermal decomposition of ammonium permanganate. *J. Chem. Soc.* **1950**, *1950*, 3674–3678. https://doi.org/10.1039/JR9500003674.

424. Griffing, V.; Maček, A. The effect of ultrasonics on the explosive sensitivity of ammonium permanganate. *Trans. Faraday Soc.* **1954**, *50*, 1331–1334. https://doi.org/10.1039/TF9545001331.
425. Jäger, H.; Lütolf, J.; Meyer, M.W. Detonation of benzyl(triethyl)ammonium permanganate. *Angew. Chem. Int. Ed. Engl.* **1979**, *18*, 786–787. https://doi.org/10.1002/anie.197907861.
426. Collett, B.G. An Examination of the Precursor Chemicals Used in the Manufacture of Explosive Compositions Found Within Improvised Explosive Devices (IEDs); Action on Armed Violence: London, UK, 2021.
427. Fiedel, R. Basic Chemistry of Explosives and Hazards of Home-Made Explosives and Chemical Precursors: Handbook; Geneva International Centre For Humanitarian Demining: Geneva, Switzerland, 2021.
428. Reducing the Threat of Improvised Explosive Device Attacks by Restricting Access to Explosive Precursor Chemicals; National Academies of Sciences, Engineering, and Medicine: Washington, DC, USA, 2018.
429. Berg, A.; Cari-Mautrand, L. Sur de nouveaux explosifs. *Bull. Soc. Chim. Fr. Ser. 3* **1893**, *9*, 94–95.
430. Cavazzi, A. Sopra un miscuglio explosivo. *Gazz. Chim. Italiana* **1886**, *16*, 172.
431. Osborn, O.; Williams, M.C.; Hradel, J.R. Magnesium Hydride Explosive Compositions. U.S. Patent 3053710A, 11 October 1962.
432. Benz, M.; Klapötke, T.M.; Krumm, B.; Lommel, M.; Stierstorfer, J. Nitrocarbamoyl azide $O_2NN(H)C(O)N_3$: A stable but highly energetic member of the carbonyl azide family. *J. Am. Chem. Soc.* **2021**, *143*, 1323–1327. https://doi.org/10.1021/jacs.0c12507.
433. Berger, B.P.; Mathieu, J.; Folly, P. Alkali-ninitramide salts. Part 2: Oxidizers for special pyrotechnic applications. *Propell. Explos. Pyrot.* **2006**, *31*, 269–277. https://doi.org/10.1002/prep.200600036.
434. Gruhne, M.S.; Wurzenberger, M.H.H.; Lommel, M.; Stierstorfer, J. A smart access to the dinitramide anion—The use of dinitraminic acid for the preparation of nitrogen-rich energetic copper(II) complexes. *Chem. Eur. J.* **2021**, *27*, 9112–9123. https://doi.org/10.1002/chem.202100747.
435. Gruhne, M.S.; Lommel, M.; Wurzenberger, M.H.H.; Klapötke, T.M.; Stierstorfer, J. Investigation of ethylenedinitramine as a versatile building block in energetic salts, cocrystals, and coordination compounds. *Inorg. Chem.* **2021**, *60*, 4816–4828. https://doi.org/10.1021/acs.inorgchem.0c03752.
436. Born, M.; Härtel, M.A.C.; Klapötke, T.M.; Mallmann, M.; Stierstorfer, J. Investigation on the sodium and potassium tetrasalts of 1,1,2,2-tetranitraminoethane. *Z. Anorg. Allg. Chem.* **2016**, *642*, 1412–1418. https://doi.org/10.1002/zaac.201600339.
437. Klapötke, T.M.; Krumm, B.; Riedelsheimer, C.; Stierstorfer, J.; Unger, C.C.; Wurzenberger, M.H.H. Urazine—A long established heterocycle and energetic chameleon. *Eur. J. Org. Chem.* **2020**, *2020*, 4916–4924. https://doi.org/10.1002/ejoc.202000886.
438. Wurzenberger, M.H.H.; Lechner, J.T.; Stierstorfer, J. Copper(II) dicyanamide complexes with *N*-substituted tetrazole ligands—Energetic coordination polymers with moderate sensitivities. *ChemPlusChem* **2020**, *85*, 769–775. https://doi.org/10.1002/cplu.202000156.
439. Gruhne, M.; Benz, M.; Lorenzen, T.; Lenz, T.; Klapötke, T.M.; Stierstorfer, J. Hybridization of dinitramide and dicyanamide: Evaluation of nitrocyanamide in energetic salts and coordination compounds. *Cryst. Growth Des.* **2022**, *22*, 200–212. https://doi.org/10.1021/acs.cgd.1c00858.
440. Harter, A.G.; Klapötke, T.M.; Riedelsheimer, C.; Stierstorfer, J.; Voggenreiter, M. Synthesis and characterization of geminal diazido derivatives based on diethyl malonate. *Eur. J. Inorg. Chem.* **2021**, *2021*, 2241–2247. https://doi.org/10.1002/ejic.202100212.
441. Klapötke, T.M.; Müller, T.G.; Rusan, M.; Stierstorfer, J. Metal salts of 4,5-dinitro-1,3-imidazole as colorants in pyrotechnic compositions. *Z. Anorg. Allg. Chem.* **2014**, *640*, 1347–1354. https://doi.org/10.1002/zaac.201400082.
442. Klapötke, T.M.; Preimesser, A.; Stierstorfer, J. Energetic derivatives of 4,4′,5, 5′-tetranitro-2,2′-bisimidazole (TNBI). *Z. Anorg. Allg. Chem.* **2012**, *638*, 1278–1286. https://doi.org/10.1002/zaac.201200188.
443. Lewczuk, R.; Rećko, J.; Szala, M. Synteza i właściwości nowego wysokoenergetycznego kompleksu $[Cu(TNBI)(NH_3)_2(H_2O)]$. *Chemik* **2015**, *69*, 123–128.
444. Drukenmüller, I.E.; Klapötke, T.M.; Morgenstern, Y.; Rusan, M.; Stierstorfer, J. Metal salts of dinitro-, trinitropyrazole, and trinitroimidazole. *Z. Anorg. Allg. Chem.* **2014**, *640*, 2139–2148. https://doi.org/10.1002/zaac.201400233.
445. Li, C.; Zhang, M.; Chen, Q.; Li, Y.; Gao, H.; Fu, W.; Zhou, Z. Three-dimensional metal–organic framework as super heat-resistant explosive: Potassium 4-(5-amino-3-nitro-1*H*-1,2,4-triazol-1-yl)-3,5-dinitropyrazole. *Chem. Eur. J.* **2017**, *23*, 1490–1493. https://doi.org/10.1002/chem.201605382.
446. Lei, C.; Yang, H.; Cheng, G. New pyrazole energetic materials and their energetic salts: Combining the dinitromethyl group with nitropyrazole. *Dalton Trans.* **2020**, *49*, 1660–1667. https://doi.org/10.1039/C9DT04235K.
447. Zhang, M.; Fu, W.; Li, C.; Gao, H.; Tang, L.; Zhou, Z. (*E*)-1,2-Bis(3,5-dinitro-1*H*-pyrazol-4-yl)diazene—Its 3D potassium metal–organic framework and organic salts with super-heat-resistant properties. *Eur. J. Inorg. Chem.* **2017**, *2017*, 2883–2891. https://doi.org/10.1002/ejic.201700001.

448. Bölter, M.F.; Harter, A.; Klapötke, T.M.; Stierstorfer, J. Isomers of dinitropyrazoles: Synthesis, comparison and tuning of their physicochemical properties. *ChemPlusChem* **2018**, *83*, 804–811. https://doi.org/10.1002/cplu.201800318.
449. Zhang, X.; Wu, Y.; Wang, P.; Lin, Q.; Chen, S.; Jin, S.; Xu, Y.; Lu, M. A Solvent-free energetic coordination polymer [*N*,*N*-(3,4-dinitropyrazolate)Ag]: Synthesis, structure and energetic performance. *Z. Anorg. Allg. Chem.* **2022**, *648*, e202100395. https://doi.org/10.1002/zaac.202100395.
450. Bölter, M.F.; Klapötke, T.M.; Kustermann, T.; Lenz, T.; Stierstorfer, J. Improving the energetic properties of dinitropyrazoles by utilization of current concepts. *Eur. J. Inorg. Chem.* **2018**, *2018*, 4125–4132. https://doi.org/10.1002/ejic.201800781.
451. Gospodinov, I.; Domasevitch, K.V.; Unger, C.C.; Klapötke, T.M.; Stierstorfer, J. Midway between energetic molecular crystals and high-density energetic salts: Crystal engineering with hydrogen bonded chains of polynitro bipyrazoles. *Cryst. Growth Des.* **2020**, *20*, 755–764. https://doi.org/10.1021/acs.cgd.9b01177.
452. Gospodinov, I.; Domasevitch, K.V.; Unger, C.C.; Benz, M.; Stierstorfer, J.; Klapötke, T.M. Energetic derivatives of 3,3′,5,5′-tetranitro-4,4′-bipyrazole (TNBPz): Synthesis, characterization and properties. *FirePhysChem* **2024**, *4*, 1–9. https://doi.org/10.1016/j.fpc.2023.04.005.
453. Zhang, J.; Parrish, D.A.; Shreeve, J.M. Thermally stable 3,6-dinitropyrazolo [4,3-*c*]pyrazole-based energetic materials. *Chem. Asian J.* **2014**, *9*, 2953–2960. https://doi.org/10.1002/asia.201402538.
454. Yin, P.; Zhang, J.; Mitchell, L.A.; Parrish, D.A.; Shreeve, J.M. 3,6-Dinitropyrazolo [4,3-*c*]pyrazole-based multipurpose energetic materials through versatile *N*-functionalization strategies. *Angew. Chem. Int. Ed.* **2016**, *55*, 12895–12897. https://doi.org/10.1002/anie.201606894.
455. Yin, P.; Mitchell, L.A.; Parrish, D.A.; Shreeve, J.M. Energetic *N*-Nitramino/*N*-oxyl-functionalized pyrazoles with versatile π–π stacking: Structure–property relationships of high-performance energetic materials. *Angew. Chem. Int. Ed.* **2016**, *55*, 14409–14411. https://doi.org/10.1002/anie.201608499.
456. Singh, J.; Staples, R.J.; Shreeve, J.M. Pushing the limit of nitro groups on a pyrazole ring with energy-stability balance. *ACS Appl. Mater. Interfaces* **2021**, *13*, 61357–61364. https://doi.org/10.1021/acsami.1c21510.
457. Zhang, J.; Zhang, J.; Imler, G.H.; Parrish, D.A.; Shreeve, J.M. Sodium and potassium 3,5-dinitro-4-hydropyrazolate: Three-dimensional metal–organic frameworks as promising super-heat-resistant explosives. *ACS Appl. Energy Mater.* **2019**, *2*, 7628–7634. https://doi.org/10.1021/acsaem.9b01608.
458. Klapötke, T.M.; Petermayer, C.; Piercey, D.G.; Stierstorfer, J. 1,3-Bis(nitroimido)-1,2,3-triazolate anion, the *N*-nitroimide moiety, and the strategy of alternating positive and negative charges in the design of energetic materials. *J. Am. Chem. Soc.* **2012**, *134*, 20827–20836. https://doi.org/10.1021/ja310384y.
459. Gu, H.; Ma, Q.; Huang, S.; Zhang, Z.; Zhang, Q.; Cheng, G.; Yang, H.; Fan, G. *Gem*-dinitromethyl-substituted energetic metal–organic framework based on 1,2,3-triazole from in situ controllable synthesis. *Chem. Asian J.* **2018**, *13*, 2786–2790. https://doi.org/10.1002/asia.201800722.
460. Glück, J.; Klapötke, T.M.; Rusan, M.; Sabatini, J.J.; Stierstorfer, J. A strontium- and chlorine-free pyrotechnic illuminant of high color purity. *Angew. Chem. Int. Ed.* **2017**, *56*, 16507–16509. https://doi.org/10.1002/anie.201710746.
461. Xu, Y.; Wang, P.; Lin, Q.; Du, Y.; Lu, M. Cationic and anionic energetic materials based on a new amphotère. *Sci. China Mater.* **2019**, *62*, 751–758. https://doi.org/10.1007/s40843-018-9374-2.
462. Glück, J.; Gospodinov, I.; Klapötke, T.M.; Stierstorfer, J. Metal salts of 3,3′-diamino-4,4′-dinitramino-5,5′-bi-1,2,4-triazole in pyrotechnic compositions. *Z. Anorg. Allg. Chem.* **2019**, *645*, 370–376. https://doi.org/10.1002/zaac.201800179.
463. Wang, T.; Zhou, J.; Zhang, Q.; Zhang, L.; Zhu, S.; Li, Y. Novel 3D cesium(I)-based EMOFs of nitrogen-rich triazole derivatives as "green" orange-light pyrotechnics. *New J. Chem.* **2020**, *44*, 1278–1284. https://doi.org/10.1039/C9NJ03577J.
464. Huang, S.; Tian, J.; Qi, X.; Wang, K.; Zhang, Q. Synthesis of *gem*-dinitromethylated and fluorodinitromethylated derivatives of 5,5′-dinitro-bis-1,2,4-triazole as promising high-energy-density materials. *Chem. Eur. J.* **2017**, *23*, 12787–12794. https://doi.org/10.1002/chem.201702451.
465. Izsák, D.; Klapötke, T.M.; Reuter, S.; Rösener, T. Silver salt and derivatives of 5-azido-1*H*-1,2,4-triazole-3-carbonitrile. *Z. Anorg. Allg. Chem.* **2013**, *639*, 899–905. https://doi.org/10.1002/zaac.201300022.
466. Izsák, D.; Klapötke, T.M.; Scharf, R.; Stierstorfer, J. Energetic materials based on the 5-azido-3-nitro-1,2,4-triazolate anion. *Z. Anorg. Allg. Chem.* **2013**, *639*, 1746–1755. https://doi.org/10.1002/zaac.201300203.
467. Wang, Y.; Yang, X.; Hu, J.; Li, H.; Li, Z.; Zhang, T. Insensitive energetic compounds: Alkaline earth metal salts of 5,5′-dinitramino-3,3′-methylene-1*H*-1,2,4-bistriazolate. *New J. Chem.* **2020**, *44*, 19054–19060. https://doi.org/10.1039/D0NJ03773G.
468. Wang, Y.; Yang, X.; Zhang, W.; Li, H.; Li, Z.; Wang, L.; Zhang, T. Energetic transition metal salts of 5,5′-dinitramino-3,3′-methylene-1*H*-1,2,4-bistriazole: Syntheses, structures and properties. *CrystEngComm* **2019**, *21*, 6452–6459. https://doi.org/10.1039/C9CE01158G.

469. Liu, T.; Qi, X.; Wang, K.; Zhang, J.; Zhang, W.; Zhang, Q. Green primary energetic materials based on *N*-(3-nitro-1-(trinitromethyl)-1*H*-1,2,4-triazol-5-yl)nitramide. *New J. Chem.* **2017**, *41*, 9070–9076. https://doi.org/10.1039/C7NJ01917C.
470. Yan, Y.-F.; Wu, H.-F.; Xu, J.-G.; Zheng, F.-K.; Guo, G.-C. A Zn(II) coordination polymer assembled by triazole derivative: Crystal structures and energetic behavior. *Inorg. Chem. Commun.* **2022**, *136*, 109168. https://doi.org/10.1016/j.inoche.2021.109168.
471. Yang, F.; Xu, Y.; Wang, P.; Lin, Q.; Lu, M. Oxygen-enriched metal–organic frameworks based on 1-(trinitromethyl)-1*H*-1,2,4-triazole-3-carboxylic acid and their thermal decomposition and effects on the decomposition of ammonium perchlorate. *ACS Appl. Mater. Interfaces* **2021**, *13*, 21516–21526. https://doi.org/10.1021/acsami.1c03110.
472. Harter, A.G.; Klapötke, T.M.; Krumm, B.; Lechner, J.T.; Riedelsheimer, C. Nitraza-/oxa-propylene- and hydrazonemethylene-bridged 1,2,4-nitraminotriazoles and selected salts. *Eur. J. Org. Chem.* **2023**, *26*, e202300302. https://doi.org/10.1002/ejoc.202300302.
473. Chen, L.; Yang, C.; Hu, H.; Shi, L.; Zhang, C.; Sun, C.; Gao, C.; Du, Y.; Hu, B. Synthesis and characterization of cyclo-pentazolate salts of iron(III) and aluminum(III). *CrystEngComm* **2022**, *24*, 8152–8159. https://doi.org/10.1039/D2CE01270G.
474. Xu, Y.; Wang, Q.; Shen, C.; Lin, Q.; Wang, P.; Lu, M. A series of energetic metal pentazolate hydrates. *Nature* **2017**, *549*, 78–81. https://doi.org/10.1038/nature23662.
475. Zhang, C.; Yang, C.; Hu, B.; Yu, C.; Zheng, Z.; Sun, C. A symmetric $Co(N_5)_2(H_2O)_4{\cdot}4\ H_2O$ high-nitrogen compound formed by cobalt(II) cation trapping of a cyclo-N_5^- anion. *Angew. Chem. Int. Ed.* **2017**, *56*, 4512–4514. https://doi.org/10.1002/anie.201701070.
476. Xu, Y.; Tian, L.; Li, D.; Wang, P.; Lu, M. A series of energetic cyclo-pentazolate salts: Rapid synthesis, characterization, and promising performance. *J. Mater. Chem. A* **2019**, *7*, 12468–12479. https://doi.org/10.1039/C9TA01077G.
477. Xu, Y.; Ding, L.; Yang, F.; Li, D.; Wang, P.; Lin, Q.; Lu, M. LiN_5: A novel pentazolate salt with high nitrogen content. *Chem. Eng. J.* **2022**, *429*, 132399. https://doi.org/10.1016/j.cej.2021.132399.
478. Sun, C.; Zhang, C.; Jiang, C.; Yang, C.; Du, Y.; Zhao, Y.; Hu, B.; Zheng, Z.; Christe, K.O. Synthesis of AgN_5 and its extended 3D energetic framework. *Nat. Commun.* **2018**, *9*, 1269. https://doi.org/10.1038/s41467-018-03678-y.
479. Yuan, Y.; Xu, Y.; Xie, Q.; Li, D.; Lin, Q.; Wang, P.; Lu, M. Pentazolate coordination polymers self-assembled by in situ generated $[Pb_4(OH)_4]^{4+}$ cubic cations trapping cyclo-N_5^-. *Dalton Trans.* **2022**, *51*, 5801–5809. https://doi.org/10.1039/D1DT04392G.
480. Hermann, T.S.; Klapötke, T.M.; Krumm, B.; Stierstorfer, J. Synthesis, characterization, and properties of di- and trinitromethyl-1,2,4-oxadiazoles and salts. *Asian J. Org. Chem.* **2018**, *7*, 739–750. https://doi.org/10.1002/ajoc.201700698.
481. Klapötke, T.M.; Mayr, N.; Stierstorfer, J.; Weyrauther, M. Maximum compaction of ionic organic explosives: Bis(hydroxylammonium)-5,5′-dinitromethyl-3,3′-bis(1,2,4-oxadiazolate) and its derivatives. *Chem. Eur. J.* **2014**, *20*, 1410–1417. https://doi.org/10.1002/chem.201303825.
482. Lu, T.; Wang, C.; Wang, G.; Wang, S.; Song, J.; Yin, H.; Fan, G.; Chen, F.-X. 1,2,4-Oxadiazole-derived polynitro energetic compounds with sensitivity reduced by a methylene bridge. *New J. Chem.* **2019**, *43*, 13330–13333. https://doi.org/10.1039/C9NJ01452G.
483. Yang, F.; Xu, Y.; Wang, P.; Lin, Q.; Lu, M. Novel metal–organic frameworks assembled from the combination of polynitro-pyrazole and 5-nitroamine-1,2,4-oxadiazole: Synthesis, structure and thermal properties. *Dalton Trans.* **2021**, *50*, 12906–12912. https://doi.org/10.1039/D1DT02093E.
484. Du, Y.; Qu, Z.; Wang, H.; Cui, H.; Wang, X. Review on the synthesis and performance for 1,3,4-oxadiazole-based energetic materials. *Propell. Explos. Pyrot.* **2021**, *46*, 860–874. https://doi.org/10.1002/prep.202000318.
485. Liu, T.; Liao, S.; Song, S.; Wang, K.; Jin, Y.; Zhang, Q. Combination of *gem*-dinitromethyl functionality and a 5-amino-1,3,4-oxadiazole framework for zwitterionic energetic materials. *Chem. Commun.* **2020**, *56*, 209–212. https://doi.org/10.1039/C9CC08182H.
486. Hermann, T.S.; Karaghiosoff, K.; Klapötke, T.M.; Stierstorfer, J. Synthesis and characterization of 2,2′-dinitramino-5,5′-bi(1-oxa-3,4-diazole) and derivatives as economic and highly dense energetic materials. *Chem. Eur. J.* **2017**, *23*, 12087–12091. https://doi.org/10.1002/chem.201702191.
487. Li, Z.; Zhang, Y.; Yuan, Y.; Jiao, N.; Liu, L. Nitrogen-rich ligands directed transition metal (Co/Ni/Zn) 3,5-dinitrobenzoic acid energetic complexes: Syntheses, crystal structures and properties. *ChemistrySelect* **2018**, *3*, 10298–10304. https://doi.org/10.1002/slct.201802583.
488. Singla, P.; Singh, A.; Sahoo, S.C.; Soni, P.K.; Kishore, P. Synthesis, characterization and reaction kinetics of an energetic copper(II) complex based on 3,5-dinitrobenzoic acid and 2,2′-bipyridine. *Chem. Pap.* **2022**, *76*, 2153–2165. https://doi.org/10.1007/s11696-021-02000-3.
489. Chen, X.; Guo, Z.; Zhang, C.; Gao, R.; Zhang, J.; Ma, H. Constructing a 3D-layered energetic metal–organic framework with the strong stacking interactions of hydrogen-bridged rings: The way to an insensitive high energy complex. *CrystEngComm* **2020**, *22*, 5436–5446. https://doi.org/10.1039/D0CE00643B.

490. Dong, Y.; Peng, P.; Hu, B.; Su, H.; Li, S.; Pang, S. High-density energetic metal–organic frameworks based on the 5,5′-dinitro-2*H*,2′*H*-3,3′-bi-1,2,4-triazole. *Molecules* **2017**, *22*, e1068. https://doi.org/10.3390/molecules22071068.
491. Su, H.; Yan, S.; Zhao, C.; Peng, P.; Jin, L.; Li, S.; Pang, S. One-step fabrication of high-performance energetic metal-organic framework $[Cu(atrz)_3[NO_3]_2]_n$ films and its tunable crystal structure. *Propell. Explos. Pyrot.* **2021**, *46*, 1746–1753. https://doi.org/10.1002/prep.202100054.
492. Zong, Z.; Zhou, F.; Chang, Q.; Shen, J.; Wang, S.; Liang, L. Construction of highly energetic metal–organic frameworks with a nitrobenzene derivative. *CrystEngComm* **2022**, *24*, 3953–3961. https://doi.org/10.1039/D2CE00464J.
493. Wu, R.-F.; Zhu, Y.-J.; Jin, W. Assembly of nickel(II) and manganese(II) coordination polymers based on a energetic aromatic dicarboxylic acid ligand. *Z. Anorg. Allg. Chem.* **2013**, *639*, 2290–2294. https://doi.org/10.1002/zaac.201300209.
494. Dufter, A.M.W.; Klapötke, T.M.; Rusan, M.; Stierstorfer, J. The lithium salts of bis(azolyl)borates as strontium- and chlorine-free red pyrotechnic colorants. *Z. Anorg. Allg. Chem.* **2020**, *646*, 580–585. https://doi.org/10.1002/zaac.201900132.
495. Dufter, A.M.W.; Klapötke, T.M.; Rusan, M.; Schweiger, A.; Stierstorfer, J. Comparison of functionalized lithium dihydrobis(azolyl)borates with their corresponding azolates as environmentally friendly red pyrotechnic coloring agents. *ChemPlusChem* **2020**, *85*, 2044–2050. https://doi.org/10.1002/cplu.202000427.
496. Glück, J.; Klapötke, T.M.; Rusan, M.; Stierstorfer, J. Green colorants based on energetic azole borates. *Chem. Eur. J.* **2014**, *20*, 15947–15960. https://doi.org/10.1002/chem.201403451.
497. Klapötke, T.M.; Rusan, M.; Stierstorfer, J. Green pyrotechnic formulations based on metal-free and nitrogen-rich tetrazolylborate salts. *J. Pyrotech.* **2014**, *33*, 24–38.
498. Singh, J.; Chinnam, A.K.; Staples, R.J.; Shreeve, J.M. Energetic salts of sensitive *N*,*N*′-(3,5-dinitropyrazine-2,6-diyl)dinitramide stabilized through three-dimensional intermolecular interactions. *Inorg. Chem.* **2022**, *61*, 16493–16500. https://doi.org/10.1021/acs.inorgchem.2c02800.
499. Shem-Tov, D.; Petrutik, N.; Wurzenberger, M.H.H.; Meincke, M.; Flaxer, E.; Tumanskii, B.; Zhang, L.; Dobrovetsky, R.; Fleischer, S.; Klapötke, T.M.; et al. Low-power laser ignition of an antenna-type secondary energetic copper complex: Synthesis, characterization, evaluation, and ignition mechanism studies. *Inorg. Chem.* **2021**, *60*, 10909–10922. https://doi.org/10.1021/acs.inorgchem.1c00358.
500. Bian, C.; Dong, X.; Zhang, X.; Zhou, Z.; Zhang, M.; Li, C. The unique synthesis and energetic properties of a novel fused heterocycle: 7-nitro-4-oxo-4,8-dihydro-[1,2,4]triazolo [5,1-*d*][1,2,3,5]tetrazine 2-oxide and its energetic salts. *J. Mater. Chem. A* **2015**, *3*, 3594–3601. https://doi.org/10.1039/C4TA06139J.
501. Zhao, B.-J.; Wang, P.; Fu, W.; Li, C.; Zhou, Z.-M. High density of a new fused heterocycle: 7,8-dinitro-4-oxo-4,6-dihydropyrazolo [5,1-*d*][1, 2,3,5]tetrazine 2-oxide and its energetic salts. *ChemistrySelect* **2018**, *3*, 4797–4803. https://doi.org/10.1002/slct.201800005.
502. Chen, S.; Jin, Y.; Xia, H.; Wang, K.; Liu, Y.; Zhang, Q. Synthesis of fused tetrazolo [1,5-*b*]pyridazine-based energetic compounds. *Energ. Mat. Front.* **2020**, *1*, 16–25. https://doi.org/10.1016/j.enmf.2020.05.001.
503. Yang, D.; Mo, W.; Zhang, S.; Li, B.; Hu, D.; Chen, S. A graphene oxide functionalized energetic coordination polymer possesses good thermostability, heat release and combustion catalytic performance for ammonium perchlorate. *Dalton Trans.* **2020**, *49*, 1582–1590. https://doi.org/10.1039/C9DT03491A.
504. Liu, J.; Lei, T.; Xue, Y.; Wang, X.; Yan, Q.-l.; Fu, X.; Ma, H.; Guo, Z. Modulation of crystal growth of an energetic metal–organic framework on the surfaces of graphene derivatives for improved detonation performance. *Langmuir* **2022**, *38*, 14959–14968. https://doi.org/10.1021/acs.langmuir.2c02743.
505. Yin, P.; Zhang, Q.; Shreeve, J.M. Dancing with energetic nitrogen atoms: Versatile *N*-functionalization strategies for *N*-heterocyclic frameworks in high energy density materials. *Acc. Chem. Res.* **2016**, *49*, 4–16. https://doi.org/10.1021/acs.accounts.5b00477.
506. Gao, H.; Zhang, Q.; Shreeve, J.M. Fused heterocycle-based energetic materials (2012–2019). *J. Mater. Chem. A* **2020**, *8*, 4193–4216. https://doi.org/10.1039/C9TA12704F.
507. Zhang, S.; Gao, Z.; Lan, D.; Jia, Q.; Liu, N.; Zhang, J.; Kou, K. Recent advances in synthesis and properties of nitrated-pyrazoles based energetic compounds. *Molecules* **2020**, *25*, e3475. https://doi.org/10.3390/molecules25153475.
508. Xie, C.; Pei, L.; Cai, J.; Yin, P.; Pang, S. Imidazole-based energetic materials: A promising family of *N*-heterocyclic framework. *Chem. Asian J.* **2022**, *17*, e202200829. https://doi.org/10.1002/asia.202200829.
509. Wang, P.; Xu, Y.; Lin, Q.; Lu, M. Recent advances in the syntheses and properties of polynitrogen pentazolate anion cyclo-N_5^- and its derivatives. *Chem. Soc. Rev.* **2018**, *47*, 7522–7538. https://doi.org/10.1039/C8CS00372F.
510. She, W.; Xu, Z.; Zhai, L.; Zhang, J.; Huang, J.; Pang, W.; Wang, B. Synthetic methods towards energetic heterocyclic *N*-oxides via several cyclization reactions. *Crystals* **2022**, *12*, e1354. https://doi.org/10.3390/cryst12101354.
511. Bu, R.; Jiao, F.; Liu, G.; Zhao, J.; Zhang, C. Categorizing and understanding energetic crystals. *Cryst. Growth Des.* **2021**, *21*, 3–15. https://doi.org/10.1021/acs.cgd.0c01300.

512. Liu, G.; Bu, R.; Huang, X.; Zhong, K.; Jiao, F.; Wei, S.-H.; Li, H.; Zhang, C. Energetic cocrystallization as the most significant crystal engineering way to create new energetic materials. *Cryst. Growth Des.* **2022**, *22*, 954–970. https://doi.org/10.1021/acs.cgd.1c01090.
513. Tariq, Q.-u.-N.; Manzoor, S.; Tariq, M.-u.-N.; Cao, W.-L.; Dong, W.-S.; Arshad, F.; Zhang, J.-G. Synthesis and energetic properties of trending metal-free potential green primary explosives: A review. *ChemistrySelect* **2022**, *7*, e202200017. https://doi.org/10.1002/slct.202200017.
514. Zhou, J.; Zhang, J.; Wang, B.; Qiu, L.; Xu, R.; Sheremetev, A.B. Recent synthetic efforts towards high energy density materials: How to design high-performance energetic structures? *FirePhysChem* **2022**, *2*, 83–139. https://doi.org/10.1016/j.fpc.2021.09.005.
515. Xu, L.; Qiao, J.; Xu, S.; Zhao, X.; Gong, W.; Huang, T. Constructing strategies and applications of nitrogen-rich energetic metal–organic framework materials. *Catalysts* **2020**, *10*, e690. https://doi.org/10.3390/catal10060690.
516. Zhang, S.; Yang, Q.; Liu, X.; Qu, X.; Wei, Q.; Xie, G.; Chen, S.; Gao, S. High-energy metal–organic frameworks (HE-MOFs): Synthesis, structure and energetic performance. *Coord. Chem. Rev.* **2016**, *307*, 292–312. https://doi.org/10.1016/j.ccr.2015.08.006.
517. Rećko, J. Explosive Coordination Materials: Acyclic Ligands—Review. *Cent. Eur. J. Energ. Mater.* **2022**, *19*, 365–378. https://doi.org/10.22211/cejem/158429.
518. Wang, Y.; Zhang, J.; Su, H.; Li, S.; Zhang, S.; Pang, S. A simple method for the prediction of the detonation performances of metal-containing explosives. *J. Phys. Chem. A* **2014**, *118*, 4575–4581. https://doi.org/10.1021/jp502857d.
519. Klapötke, T.M. *Energetic Materials Encyclopedia*; de Gruyter: Berlin, Germany, 2018.
520. Patnaik, P. A comprehensive Guide to the Hazardous Properties of Chemical Substances, 3rd ed.; Wiley: New Jersey, 2007.
521. Keshavarz, M.H.; Klapötke, T.M. *Energetic Compounds*; De Gruyter: Berlin, Germany; Boston, MA, USA, 2020.
522. Klapötke, T.M. *Chemistry of High-Energy Materials*; De Gruyter: Berlin, Germany; Boston, MA, USA, 2022.
523. Teipel, U. Energetic Materials: Particle Processing and Characterization; Wiley: Weinheim, Germany, 2005.
524. Meyer, R.; Köhler, J.; Homburg, A. *Explosives*, 7th ed.; Wiley: Weinheim, Germany, 2015.
525. Shteinberg, A.S. Fast Reactions in Energetic Materials: High-Temperature Decomposition of Rocket Propellants and Explosives; Springer: Berlin, Germany, 2008.
526. Agrawal, S.P. High Energy Materials: Propellants, Explosives and Pyrotechnics; Wiley: Weinheim, Germany, 2010.
527. Clark, J.D. Ignition! An Informal History of Liquid Rocket Propellants; Rutgers University Press: New Brunswick, NJ, USA, 2017.
528. Liu, J. *Liquid Explosives*; Springer: Heidelberg, Germany, 2015.
529. Agrawal, S.P.; Hodgson, R.D. *Organic Chemistry of Explosives*; Wiley: Southern Gate, CA, USA, 2007.
530. Kubota, N. Propellants and Explosives: Thermochemical Aspects of Combustion, 2nd ed.; Wiley: Weinheim, Germany, 2007.
531. Otłowski, T.; Zalas, M.; Gierczyk, B. Forensic analytical aspects of homemade explosives containing grocery powders and hydrogen peroxide. *Sci. Rep.* **2024**, *14*, e750. https://doi.org/10.1038/s41598-024-51335-w.
532. Ru, C.; Chen, L.; Zhang, H.; Wang, H.; Xu, H.; Chi, Z.; Zhang, Y. Exploration of the reactivities of homemade binary pyrotechnics. *Sci. Rep.* **2024**, *14*, e27555. https://doi.org/10.1038/s41598-024-79212-6.
533. Vanderheyden, N.; Verhoeven, E.; Vermeuler, S.; Bekaert, B. Survival of forensic trace evidence on improvised explosive devices: Perspectives on individualisation. *Sci. Rep.* **2020**, *10*, e12813. https://doi.org/10.1038/s41598-020-69385-1.
534. Michel, P.; Boudenne, J.-L.; Robert-Peillard, F.; Coulomb, B. Analysis of homemade peroxide-based explosives in water: A review. *Trends Anal. Chem.* **2023**, *158*, e116884. https://doi.org/10.1016/j.trac.2022.116884.
535. Schultz, S. It's Elementary: A Review of Forensic Analysis Techniques of Inorganic Components of Explosives. *Chem. Biochem. Student Projects* **2019**, e16. Available on-line: https://pillars.taylor.edu/chemistry-student/16 (Accesed on: 24 November 2024).
536. Olsson, F. Trace Amount Analysis of Common Explosives in Bodies of Water Using UHPLC-HRMS Orbitrap. Bachelor's Thesis, Linköping University, Linköping, Sweden, 2019.
537. Ostrinskaya, A.; Kunz, R.R.; Clark, M.; Kingsborough, R.P.; Ong, T.-H.; Deneaut, S. Rapid quantitative analysis of multiple explosive compound classes on a single instrument via flow-injection analysis tandem mass spectrometry. *J. Forensic Sci.* **2018**, *64*, 223–230. https://doi.org/10.1111/1556-4029.13827.
538. Wiss, J.; Fleury, C.; Heuberger, C.; Onken, U.; Glor, M. Explosion and decomposition characteristics of hydrazoic acid in the gas phase. *Org. Process Res. Dev.* **2007**, *11*, 1096–1103. https://doi.org/10.1021/op7000645.
539. Curtius, T.; Rissom, J. Neue Untersuchungen über den Stickstoffwasserstoff N_3H. *J. Prakt. Chem.* **1898**, *58*, 261–309. https://doi.org/10.1002/prac.18980580113.
540. Fox, P.G. The explosive sensitivity of the metal azides to impact. *J. Solid State Chem.* **1970**, *2*, 491–502. https://doi.org/10.1016/0022-4596(70)90043-5.

541. Schulz, A.; Villinger, A. Binary polyazides of cadmium and mercury. *Chem. Eur. J.* **2015**, *21*, 3649–3663. https://doi.org/10.1002/chem.201406023.
542. Schulz, A.; Villinger, A. Binary zinc azides. *Chem. Eur. J.* **2016**, *22*, 2032–2038. https://doi.org/10.1002/chem.201504524.
543. Straumanis, M.; Cīrulis, A. Das Kupfer(II)-azid. Darstellungsmethoden, Bildung und Eigenschaften. *Z. Anorg. Allg. Chem.* **1943**, *251*, 315–331. https://doi.org/10.1002/zaac.19432510402.
544. Singh, K. Sensitivity of cuprous azide towards heat and impact. *Trans. Faraday Soc.* **1959**, *55*, 124–129. https://doi.org/10.1039/TF9595500124.
545. Cīrulis, A.; Straumanis, M. Die basischen Kupfer(II)-azide. *Z. Anorg. Allg. Chem.* **1943**, *251*, 332–334. https://doi.org/10.1002/zaac.19432510403.
546. Straumanis, M.; Cīrulis, A. Komplexverbindungen des Kupfer(II)-azids. I. Die Einlagerungsverbindungen. *Z. Anorg. Allg. Chem.* **1943**, *251*, 335–340. https://doi.org/10.1002/zaac.19432510404.
547. Cīrulis, A.; Straumanis, M. Komplexverbindungen des Kupfer(II)-azids. II. Nichtelektrolyte. *Z. Anorg. Allg. Chem.* **1943**, *251*, 341–354. https://doi.org/10.1002/zaac.19432510405.
548. Gruhne, M.S.; Lommel, M.; Wurzenberger, M.H.H.; Szimhardt, N.; Klapötke, T.M.; Stierstorfer, J. OZM Ball Drop Impact Tester (BIT-132) vs. BAM standard method—A comparative investigation. *Propell. Explos. Pyrot.* **2020**, *45*, 147–153. https://doi.org/10.1002/prep.201900286.
549. Miles, F.D. The formation and characteristics of crystals of lead azide and of some other initiating explosives. *J. Chem. Soc.* **1931**, 2532–2542. https://doi.org/10.1039/JR9310002532.
550. Wurzenberger, M.H.H.; Gruhne, M.S.; Lommel, M.; Szimhardt, N.; Stierstorfer, J. Advancement and stabilization of copper(II) azide by the use of triazole- and tetrazole ligands—Enhanced primary explosives. *Mat. Adv.* **2022**, *3*, 579–591. https://doi.org/10.1039/D1MA00588J.
551. Liu, X.; Stoffel, R.; Dronskowski, R. Syntheses, crystal structures, and vibrational properties of two lead azide halides PbN_3X (X = Cl, Br). *Z. Anorg. Allg. Chem.* **2020**, *646*, 1525–1530. https://doi.org/10.1002/zaac.202000064.
552. Yang, L.; Pei, Q.; Zhang, T.; Zhang, J.; Cao, Y. Solubilities and enthalpies of solution of picric acid and picrates at 298.15K in DMF, EtOH and acetic acid. *Thermochim. Acta* **2007**, *463*, 13–14. https://doi.org/10.1016/j.tca.2007.04.013.
553. Hopper, J.D. Explosive characteristics of certain metallic picrates. *J. Franklin Inst.* **1938**, *225*, 219–225. https://doi.org/10.1016/S0016-0032(38)90327-8.
554. Matsukawa, M.; Matsunaga, T.; Yoshida, M.; Fujiwara, S. Synthesis and properties of alkali metal picrates. *Sci. Technol. Energetic Mater.* **2003**, *64*, 183–191.
555. Tucholski, T. Analiza termiczna związków wybuchowych. *Acta Phys. Pol.* **1932**, *1*, 351–355.
556. Tucholski, T. Analiza termiczna pikrynianów. I. Odwodnienie, temperatury topnienia, inicjujące i przedwybuchowe pikrynianów Li, Na, K, Rb, Cs, Cu i Ag. *Rocz. Chem.* **1933**, *13*, 435–447.
557. Dinegar, R.H. The ignition and deflagration of potassium picrte (KP) and KP/explosives mixtures: Low-voltage, nonprimary detonators. In Proceedings of the 6th International Pyrotechnics Seminar, Estes Park, CO, USA, 17–21 July 1978; Denver Research Institute: Denver, CO, USA, 1978; pp. 119–134.
558. Tucholski, T. Analiza termiczna pikrynianów. IV. Odwodnienie, temperatury topnienia, temperatury inicjujące i przedwybuchowe pikrynianów Al, Sc, Y, La, Ga, In, i Tl. *Rocz. Chem.* **1934**, *14*, 430–450.
559. Tucholski, T. Analiza termiczna pikrynianów. II. Odwodnienie, temperatury topnienia, inicjujące i przedwybuchowe pikrynianów Be, Mg, Zn, Cd, Hg, Ca, Sr i Ba. *Rocz. Chem.* **1934**, *14*, 125–140.
560. Matsukawa, M.; Matsunaga, T.; Yoshida, M.; Fujiwara, S. Synthesis of alkaline-earth metal picrates. *Sci. Technol. Energetic Mater.* **2003**, *64*, 227–235.
561. Tucholski, T. Analiza termiczna pikrynianów. III. Odwodnienie, temperatury topnienia, inicjujące i przedwybuchowe pikrynianów Cr, Mn, Fe, Co i Ni. *Rocz. Chem.* **1934**, *14*, 259–267.
562. Akiyoshi, M.; Okada, K.; Matsunaga, T. Thermal hazard of iron picrate. *J. Hazard. Mater.* **2006**, *132*, 141–147. https://doi.org/10.1016/j.jhazmat.2005.07.088.
563. Agarwal, J.P.; Agrawal, S.P. A comparative study on some explosive properties of cobalt picrate and cobalt picramate. *Propell. Explos. Pyrot.* **1981**, *6*, 112–116. https://doi.org/10.1002/prep.19810060407.
564. Orbovic, N.; Codoceo, C.L. Production of exploding materials for detonators: Control of crystal growth of lead and barium 2,4,6-trinitroresorcinate. *Propell. Explos. Pyrot.* **2008**, *33*, 459–466. https://doi.org/10.1002/prep.200700254.
565. Finch, A.; Gardner, P.J.; Head, A.J.; Majdi, H.S. The standard enthalpy of formation of silver acetylide. *Thermochim. Acta* **1991**, *180*, 325–330. https://doi.org/10.1016/0040-6031(91)80402-5.
566. Plimpton, R.T.; Travers, M.W. Metallic derivatives of acetylene. I. Mercuric acetylide. *J. Chem. Soc. Trans.* **1894**, *65*, 264–269. https://doi.org/10.1039/CT8946500264.
567. Keiser, E.H. The metallic derivatives of acetylene. *Am. Chem. J.* **1893**, *15*, 535–539.
568. Burkard, E.; Travers, M.W. The action of acetylene on the acetates of mercury. *J. Chem. Soc. Trans.* **1902**, *81*, 1270–1272. https://doi.org/10.1039/CT9028101270.

569. Mathews, J.A.; Watters, L.L. The carbide of gold. *J. Am. Chem. Soc.* **1900**, *22*, 108–111. https://doi.org/10.1021/ja02040a010.
570. Mahdavi, M.; Ghani, K.; Nosratzadegan, K. Synthesis, Structural and energetic properties of copper(II) perchlorate complex with aminoguanidine. *Z. Anorg. Allg. Chem.* **2017**, *643*, 1771–1775. https://doi.org/10.1002/zaac.201700311.
571. Stierstorfer, J.; Klapötke, T.M.; Hammerl, A.; Chapman, R.D. 5-Azido-1*H*-tetrazole—Improved synthesis, crystal structure and sensitivity data. *Z. Anorg. Allg. Chem.* **2008**, *634*, 1051–1057. https://doi.org/10.1002/zaac.200800003.
572. Klapötke, T.M.; Miró Sabaté, C.; Rasp, M. Synthesis and properties of 5-nitrotetrazole derivatives as new energetic materials. *J. Mater. Chem.* **2009**, *19*, 2240–2252. https://doi.org/10.1039/B818925K.
573. Dippold, A.A.; Klapötke, T.M. Synthesis and characterization of 5-(1,2,4-triazol-3-yl)tetrazoles with various energetic functionalities. *Chem. Asian J.* **2013**, *8*, 1463–1471. https://doi.org/10.1002/asia.201300063.
574. Dippold, A.A.; Izsák, D.; Klapötke, T.M. A study of 5-(1,2,4-triazol-*C*-yl)tetrazol-1-ols: Combining the benefits of different heterocycles for the design of energetic materials. *Chem. Eur. J.* **2013**, *19*, 12042–12051. https://doi.org/10.1002/chem.201301339.
575. Klapötke, T.M.; Leroux, M.; Schmid, P.C.; Stierstorfer, J. Energetic materials based on 5,5′-diamino-4,4′-dinitramino-3,3′-bi-1,2,4-triazole. *Chem. Asian J.* **2016**, *11*, 844–851. https://doi.org/10.1002/asia.201500701.
